# Translational genomics of osteoarthritis in 1,962,069 individuals

**DOI:** 10.1038/s41586-025-08771-z

**Published:** 2025-04-09

**Authors:** Konstantinos Hatzikotoulas, Lorraine Southam, Lilja Stefansdottir, Cindy G. Boer, Merry-Lynn McDonald, J. Patrick Pett, Young-Chan Park, Margo Tuerlings, Rick Mulders, Andrei Barysenka, Ana Luiza Arruda, Vinicius Tragante, Alison Rocco, Norbert Bittner, Shibo Chen, Susanne Horn, Vinodh Srinivasasainagendra, Ken To, Georgia Katsoula, Peter Kreitmaier, Amabel M. M. Tenghe, Arthur Gilly, Liubov Arbeeva, Lane G. Chen, Agathe M. de Pins, Daniel Dochtermann, Cecilie Henkel, Jonas Höijer, Shuji Ito, Penelope A. Lind, Bitota Lukusa-Sawalena, Aye Ko Ko Minn, Marina Mola-Caminal, Akira Narita, Chelsea Nguyen, Ene Reimann, Micah D. Silberstein, Anne-Heidi Skogholt, Hemant K. Tiwari, Michelle S. Yau, Ming Yue, Wei Zhao, Jin J. Zhou, George Alexiadis, Karina Banasik, Søren Brunak, Archie Campbell, Jackson T. S. Cheung, Joseph Dowsett, Tariq Faquih, Jessica D. Faul, Lijiang Fei, Anne Marie Fenstad, Takamitsu Funayama, Maiken E. Gabrielsen, Chinatsu Gocho, Kirill Gromov, Thomas Hansen, Georgi Hudjashov, Thorvaldur Ingvarsson, Jessica S. Johnson, Helgi Jonsson, Saori Kakehi, Juha Karjalainen, Elisa Kasbohm, Susanna Lemmelä, Kuang Lin, Xiaoxi Liu, Marieke Loef, Massimo Mangino, Daniel McCartney, Iona Y. Millwood, Joshua Richman, Mary B. Roberts, Kathleen A. Ryan, Dino Samartzis, Manu Shivakumar, Søren T. Skou, Sachiyo Sugimoto, Ken Suzuki, Hiroshi Takuwa, Maris Teder-Laving, Laurent Thomas, Kohei Tomizuka, Constance Turman, Stefan Weiss, Tian T. Wu, Eleni Zengini, Yanfei Zhang, arcOGEN Consortium, Lorraine Southam, Ana M. Valdes, J. Mark Wilkinson, Eleftheria Zeggini, ARGO Consortium, Konstantinos Hatzikotoulas, George Alexiadis, Eleni Zengini, George Babis, Aspasia Tsezou, J. Mark Wilkinson, Eleftheria Zeggini, DBDS Genomic Consortium, Cecilie Henkel, Karina Banasik, Søren Brunak, Joseph Dowsett, Kirill Gromov, Thomas Hansen, Søren T. Skou, Christian Erikstrup, Sisse Rye Ostrowski, Ole Birger Pedersen, Erik Sørensen, Anders Troelsen, Henrik Ullum, Kari Stefansson, Estonian Biobank Research Team, Ene Reimann, Georgi Hudjashov, Maris Teder-Laving, Reedik Mägi, FinnGen, Juha Karjalainen, Susanna Lemmelä, Mark Daly, Aarno Palotie, Genes & Health Research Team, David A. van Heel, Robin Lerner, HUNT All-In Pain, Anne-Heidi Skogholt, Laurent Thomas, Bendik Winsvold, Maiken Gabrielsen, Kristian Hveem, John-Anker Zwart, Million Veteran Program, Merry-Lynn McDonald, Alison Rocco, Vinodh Srinivasasainagendra, Daniel Dochtermann, Bitota Lukusa-Sawalena, Chelsea Nguyen, Hemant K. Tiwari, Jin J. Zhou, Joshua Richman, Jasvinder A. Singh, Regeneron Genetics Center, Arthur Gilly, Manuel Allen Revez Ferreira, Aris Baras, Marcus Jones, Luca Lotta, Manuel Allen Revez Ferreira, George Babis, Aris Baras, Tyler Barker, David J. Carey, Kathryn S. E. Cheah, Zhengming Chen, Jason Pui-Yin Cheung, Mark Daly, Renée de Mutsert, Charles B. Eaton, Christian Erikstrup, Ove Nord Furnes, Yvonne M. Golightly, Daniel F. Gudbjartsson, Nils P. Hailer, Caroline Hayward, Marc C. Hochberg, Georg Homuth, Laura M. Huckins, Kristian Hveem, Shiro Ikegawa, Muneaki Ishijima, Minoru Isomura, Marcus Jones, Jae H. Kang, Sharon L. R. Kardia, Margreet Kloppenburg, Peter Kraft, Nobuyuki Kumahashi, Suguru Kuwata, Ming Ta Michael Lee, Phil H. Lee, Robin Lerner, Liming Li, Steve A. Lietman, Luca Lotta, Michelle K. Lupton, Reedik Mägi, Nicholas G. Martin, Timothy E. McAlindon, Sarah E. Medland, Karl Michaëlsson, Braxton D. Mitchell, Dennis O. Mook-Kanamori, Andrew P. Morris, Toru Nabika, Fuji Nagami, Amanda E. Nelson, Sisse Rye Ostrowski, Aarno Palotie, Ole Birger Pedersen, Frits R. Rosendaal, Mika Sakurai-Yageta, Carsten Oliver Schmidt, Pak Chung Sham, Jasvinder A. Singh, Diane T. Smelser, Jennifer A. Smith, You-qiang Song, Erik Sørensen, Gen Tamiya, Yoshifumi Tamura, Chikashi Terao, Gudmar Thorleifsson, Anders Troelsen, Aspasia Tsezou, Yuji Uchio, A. G. Uitterlinden, Henrik Ullum, Ana M. Valdes, David A. van Heel, Robin G. Walters, David R. Weir, J. Mark Wilkinson, Bendik S. Winsvold, Masayuki Yamamoto, John-Anker Zwart, Kari Stefansson, Ingrid Meulenbelt, Sarah A. Teichmann, Joyce B. J. van Meurs, Unnur Styrkarsdottir, Eleftheria Zeggini

**Affiliations:** 1 Institute of Translational Genomics, Helmholtz Zentrum München, German Research Center for Environmental Health, Neuherberg, Germany; 2 deCODE Genetics/Amgen, Reykjavik, Iceland; 3 Department of Internal Medicine, Erasmus MC Medical Center, Rotterdam, The Netherlands; 4 Birmingham Veterans Affairs Healthcare System (BVAHS), Birmingham, AL USA; 5 Division of Pulmonary, Allergy and Critical Care Medicine, Department of Medicine, School of Medicine, University of Alabama at Birmingham (UAB), Birmingham, AL USA; 6 Department of Epidemiology, School of Public Health, UAB, Birmingham, AL USA; 7 Department of Genetics, School of Medicine, UAB, Birmingham, AL USA; 8 Wellcome Sanger Institute, Cellular Genetics Programme, Wellcome Genome Campus, Cambridge, UK; 9 Department of Biomedical Data Sciences, Section Molecular Epidemiology, Leiden University Medical Center, Leiden, The Netherlands; 10 Department of Biostatistics, School of Public Health, UAB, Birmingham, AL USA; 11 Cambridge Stem Cell Institute, University of Cambridge, Jeffrey Cheah Biomedical Centre, Cambridge Biomedical Campus, Cambridge, UK; 12 Department of Surgery, University of Cambridge, Cambridge, UK; 13 Centre for Genetic Epidemiology, Institute for Clinical Epidemiology and Applied Biometry, University of Tubingen, Tübingen, Germany; 14 Regeneron Genetics Center, Tarrytown, NY USA; 15 Thurston Arthritis Research Center, University of North Carolina at Chapel Hill, Chapel Hill, NC USA; 16 Department of Psychiatry, The University of Hong Kong, Pok Fu Lam, Hong Kong; 17 Icahn School of Medicine at Mount Sinai, New York, NY USA; 18 Clinical Orthopaedic Research Hvidovre (CORH), Department of Orthopaedic Surgery, Copenhagen University Hospital Hvidovre, Hvidovre, Denmark; 19 Medical Epidemiology, Department of Surgical Sciences, Uppsala University, Uppsala, Sweden; 20 Department of Orthopedic Surgery, Shimane University Faculty of Medicine, Izumo, Japan; 21 Laboratory for Bone and Joint Diseases, RIKEN Center for Integrative Medical Sciences, Tokyo, Japan; 22 Laboratory for Statistical and Translational Genetics, RIKEN Center for Integrative Medical Sciences, Kanagawa, Japan; 23 Psychiatric Genetics, Brain and Mental Health Research Program, QIMR Berghofer Medical Research Institute, Brisbane, Queensland Australia; 24 School of Biomedical Sciences, Faculty of Medicine, University of Queensland, Brisbane, Queensland Australia; 25 School of Biomedical Sciences, Queensland University of Technology, Brisbane, Queensland Australia; 26 Tohoku University Graduate School of Medicine, Sendai, Japan; 27 Tohoku Medical Megabank Organization, Tohoku University, Sendai, Japan; 28 Estonian Genome Center, Institute of Genomics, University of Tartu, Tartu, Estonia; 29 Center for Genomic Medicine, Massachusetts General Hospital, Boston, MA USA; 30 HUNT Center for Molecular and Clinical Epidemiology, Department of Public Health and Nursing, Faculty of Medicine and Health Sciences, Norwegian University of Science and Technology, Trondheim, Norway; 31 Hinda and Arthur Marcus Institute for Aging Research, Hebrew SeniorLife, Harvard Medical School, Boston, MA USA; 32 School of Biomedical Sciences, The University of Hong Kong, Pok Fu Lam, Hong Kong; 33 Survey Research Center, Institute for Social Research, University of Michigan, Ann Arbor, MI USA; 34 Department of Epidemiology, School of Public Health, University of Michigan, Ann Arbor, MI USA; 35 Department of Biostatistics, UCLA Fielding School of Public Health, Los Angeles, CA USA; 36 1st Department of Orthopaedics, KAT General Hospital, Athens, Greece; 37 Novo Nordisk Foundation Center for Protein Research, Faculty of Health and Medical Sciences, University of Copenhagen, Copenhagen, Denmark; 38 Centre for Genomic and Experimental Medicine, Institute of Genetics & Cancer, University of Edinburgh, Edinburgh, UK; 39 Faculty of Medical Sciences, University College London, London, UK; 40 Department of Clinical Immunology, Copenhagen University Hospital—Rigshospitalet, Copenhagen, Denmark; 41 Department of Clinical Epidemiology, Leiden University Medical Center, Leiden, The Netherlands; 42 Division of Sleep and Circadian Disorders, Brigham and Women’s Hospital and Harvard Medical School, Boston, MA USA; 43 The Norwegian Arthroplasty Register, Department of Orthopaedic Surgery, Haukeland University Hospital, Bergen, Norway; 44 Danish Headache Center, Department of Neurology, Copenhagen University Hospital, Rigshospitalet – Glostrup, Glostrup, Denmark; 45 Department of Orthopedic Surgery, Akureyri Hospital, Akureyri, Iceland; 46 Faculty of Medicine, University of Iceland, Reykjavik, Iceland; 47 Pamela Sklar Division of Psychiatric Genomics, Department of Genetics and Genomic Sciences, Icahn School of Medicine at Mount Sinai, New York, NY USA; 48 Department of Psychiatry, School of Medicine, University of North Carolina at Chapel Hill, Chapel Hill, NC USA; 49 Department of Medicine, Landspitali The National University Hospital of Iceland, Reykjavik, Iceland; 50 Department of Metabolism & Endocrinology, Sportology Center, Juntendo University Graduate School of Medicine, Juntendo University, Tokyo, Japan; 51 Institute for Molecular Medicine Finland (FIMM), Helsinki Institute of Life Science (HiLIFE), University of Helsinki, Helsinki, Finland; 52 Program in Medical and Population Genetics, Broad Institute of Harvard and MIT, Cambridge, MA USA; 53 Stanley Center for Psychiatric Research, Broad Institute of Harvard and MIT, Cambridge, MA USA; 54 Analytic and Translational Genetics Unit, Massachusetts General Hospital, Boston, MA USA; 55 Institute for Community Medicine, SHIP-KEF, University Medicine Greifswald, Greifswald, Germany; 56 Finnish Institute for Health and Welfare (THL), Helsinki, Finland; 57 Nuffield Department of Population Health, University of Oxford, Oxford, UK; 58 Department of Rheumatology, Leiden University Medical Center, Leiden, The Netherlands; 59 Department of Twin Research and Genetic Epidemiology, Kings College London, London, UK; 60 Department of Surgery, School of Medicine, UAB, Birmingham, AL USA; 61 Center for Primary Care & Prevention, Brown University, Pawtucket, RI USA; 62 Department of Medicine, University of Maryland School of Medicine, Baltimore, MD USA; 63 Department of Orthopaedics and Traumatology, The University of Hong Kong, Hong Kong SAR, China; 64 Department of Orthopaedic Surgery, Rush University Medical Center, Chicago, IL USA; 65 Department of Biostatistics, Epidemiology and Informatics, Perelman School of Medicine, University of Pennsylvania, Philadelphia, PA USA; 66 Research Unit for Musculoskeletal Function and Physiotherapy, Department of Sports Science and Clinical Biomechanics, University of Southern Denmark, Odense, Denmark; 67 The Research and Implementation Unit PROgrez, Department of Physiotherapy and Occupational Therapy, Næstved-Slagelse-Ringsted Hospitals, Slagelse, Denmark; 68 Centre for Genetics and Genomics Versus Arthritis, Centre for Musculoskeletal Research, Division of Musculoskeletal and Dermatological Sciences, University of Manchester, Manchester, UK; 69 Department of Diabetes and Metabolic Diseases, Graduate School of Medicine, University of Tokyo, Tokyo, Japan; 70 Department of Statistical Genetics, Osaka University Graduate School of Medicine, Suita, Japan; 71 Department of Clinical and Molecular Medicine, Norwegian University of Science and Technology, Trondheim, Norway; 72 Department of Epidemiology, Harvard T.H. Chan School of Public Health, Boston, MA USA; 73 Department of Functional Genomics, Interfaculty Institute for Genetics and Functional Genomics, University Medicine Greifswald, Greifswald, Germany; 74 4th Psychiatric Department, Dromokaiteio Psychiatric Hospital, Haidari, Athens, Greece; 75 Genomic Medicine Institute, Geisinger Health System, Danville, PA USA; 76 2nd Department of Orthopaedics, National and Kapodistrian University of Athens, Medical School, ‘Konstantopouleio’ Hospital, Athens, Greece; 77 Sports Medicine Research Institute, The Ohio State University Wexner Medical Center, Columbus, OH USA; 78 Intermountain Healthcare, Precision Genomics, Salt Lake City, UT USA; 79 Department of Orthopaedics, University of Utah, Salt Lake City, UT USA; 80 Department of Genomic Health, Geisinger, Danville, PA USA; 81 Department of Family Medicine, Warren Alpert Medical School of Brown University, Providence, RI USA; 82 Department of Epidemiology, School of Public Health, Brown University, Providence, RI USA; 83 Department of Clinical Immunology, Aarhus University Hospital, Aarhus, Denmark; 84 Department of Clinical Medicine, Aarhus University, Aarhus, Denmark; 85 Department of Clinical Medicine, University of Bergen, Bergen, Norway; 86 University of Nebraska Medical Center, Omaha, NE USA; 87 School of Engineering and Natural Sciences, University of Iceland, Reykjavik, Iceland; 88 Orthopedics, Department of Surgical Sciences, Uppsala University, Uppsala, Sweden; 89 MRC Human Genetics Unit IGC, University of Edinburgh, Edinburgh, UK; 90 Department of Epidemiology and Public Health, Department of Medicine, University of Maryland School of Medicine, Baltimore, MD USA; 91 Department of Psychiatry, Yale School of Medicine, Yale University, New Haven, CT USA; 92 HUNT Research Center, Department of Public Health and Nursing, Faculty of Medicine and Health Sciences, Norwegian University of Science and Technology, Levanger, Norway; 93 Levanger Hospital, Nord-Trøndelag Hospital Trust, Levanger, Norway; 94 Department of Medicine for Orthopaedics and Motor Organ, Sportology Center, Juntendo University Graduate School of Medicine, Tokyo, Japan; 95 Faculty of Human Sciences, Shimane University, Matsue, Japan; 96 Channing Division of Network Medicine, Department of Medicine, Brigham and Women’s Hospital, Boston, MA USA; 97 Department of Orthopedic Surgery, Matsue Red Cross Hospital, Matsue, Japan; 98 Department of Psychiatry, Harvard Medical School, Boston, USA; 99 Blizard Institute, Queen Mary University of London, London, UK; 100 Department of Epidemiology & Biostatistics, School of Public Health, Peking University, Beijing, China; 101 Peking University Center for Public Health and Epidemic Preparedness & Response, Beijing, China; 102 Musculoskeletal Institute, Geisinger, Danville, PA USA; 103 Neurogenetics and Dementia, Brain and Mental Health Research Program, QIMR Berghofer Medical Research Institute, Brisbane, Queensland Australia; 104 School of Biomedical Sciences, Faculty of Health, Queensland University of Technology, Brisbane, Queensland Australia; 105 Genetic Epidemiology, Brain and Mental Health Research Program, QIMR Berghofer Medical Research Institute, Brisbane, Queensland Australia; 106 Division of Rheumatology, Allergy, & Immunology, Tufts Medical Center, Boston, MA USA; 107 School of Psychology, University of Queensland, Brisbane, Queensland Australia; 108 Faculty of Medicine, University of Queensland, Brisbane, Queensland Australia; 109 Department of Public Health, Leiden University Medical Center, Leiden, The Netherlands; 110 Department of Functional Pathology, Shimane University School of Medicine, Izumo, Japan; 111 Department of Clinical Medicine, University of Copenhagen, Copenhagen, Denmark; 112 Department of Clinical Immunology, Zealand University Hospital – Køge, Køge, Denmark; 113 Li Ka Shing Faculty of Medicine, The University of Hong Kong, Pok Fu Lam, Hong Kong; 114 Division of Rheumatology and Clinical Immunology, Department of Medicine at the School of Medicine, UAB, Birmingham, AL USA; 115 Medicine Service, Michale E. DeBakey VA Medical Center, Houston, TX USA; 116 Department of Medicine, Baylor College of Medicine, Houston, TX USA; 117 Statistical Genetics Team, Center for Advanced Intelligence Project, RIKEN, Tokyo, Japan; 118 Department of Metabolism & Endocrinology, Sportology Center, Juntendo University Graduate School of Medicine, Tokyo, Japan; 119 Clinical Research Center, Shizuoka General Hospital, Shizuoka, Japan; 120 The Department of Applied Genetics, The School of Pharmaceutical Sciences, University of Shizuoka, Shizuoka, Japan; 121 Clinical Academic Group: Research OsteoArthritis Denmark (CAG ROAD), Department of Orthopaedic Surgery, Copenhagen University Hospital Hvidovre, Hvidovre, Denmark; 122 Laboratory of Cytogenetics and Molecular Genetics, Faculty of Medicine, University of Thessaly, Larissa, Greece; 123 Statens Serum Institute, Copenhagen, Denmark; 124 Faculty of Medicine & Health Sciences, School of Medicine, University of Nottingham, Nottingham, UK; 125 School of Medicine and Population Health, The University of Sheffield, Sheffield, UK; 126 Department of Research, Innovation and Education, Division of Clinical Neuroscience, Oslo University Hospital and University of Oslo, Oslo, Norway; 127 Department of Neurology, Oslo University Hospital, Oslo, Norway; 128 Institute of Clinical Medicine, Faculty of Medicine, University of Oslo, Oslo, Norway; 129 Department of Medicine & Cambridge Stem Cell Institute, University of Cambridge, Cambridge, UK; 130 TUM School of Medicine and Health, Technical University of Munich and Klinikum Rechts der Isar, Munich, Germany

**Keywords:** Osteoarthritis, Genome-wide association studies, Genomics, Functional genomics, Translational research

## Abstract

Osteoarthritis is the third most rapidly growing health condition associated with disability, after dementia and diabetes^1^. By 2050, the total number of patients with osteoarthritis is estimated to reach 1 billion worldwide^2^. As no disease-modifying treatments exist for osteoarthritis, a better understanding of disease aetiopathology is urgently needed. Here we perform a genome-wide association study meta-analyses across up to 489,975 cases and 1,472,094 controls, establishing 962 independent associations, 513 of which have not been previously reported. Using single-cell multiomics data, we identify signal enrichment in embryonic skeletal development pathways. We integrate orthogonal lines of evidence, including transcriptome, proteome and epigenome profiles of primary joint tissues, and implicate 700 effector genes. Within these, we find rare coding-variant burden associations with effect sizes that are consistently higher than common frequency variant associations. We highlight eight biological processes in which we find convergent involvement of multiple effector genes, including the circadian clock, glial-cell-related processes and pathways with an established role in osteoarthritis (TGFβ, FGF, WNT, BMP and retinoic acid signalling, and extracellular matrix organization). We find that 10% of the effector genes express a protein that is the target of approved drugs, offering repurposing opportunities, which can accelerate translation.

## Abstract (web summary)

A genome-wide association study meta-analysis combined with multiomics data of osteoarthritis identifies 700 effector genes as well as biological processes with a convergent involvement of multiple effector genes; 10% of these genes express the target of approved drugs.

## Main

Osteoarthritis is one of the most rapidly increasing health conditions globally, and among the leading causes of disability and pain^1^. The global burden of osteoarthritis has reached a staggering 595 million individuals, representing a notable 132% increase in prevalence since 1990^2^. The total number of patients with osteoarthritis has been estimated to reach 1 billion worldwide by 2050^2^. Despite the enormous societal and public health burden of osteoarthritis, no effective disease-modifying treatments exist. It is therefore imperative to enhance our understanding of the biological processes leading to disease development to accelerate translation.

Osteoarthritis is a complex disease, caused by an interplay between environmental and genetic risk factors. Previous genome-wide association studies (GWASs) have led to the identification of around 150 risk variants, mediated through effector genes involved in various pathways^3^. Here we conducted a large-scale GWAS meta-analysis across 1,962,069 individuals, achieving a 2.64-fold increase in effective sample size compared with the next largest GWAS^3^. We combine the genetic findings with functional genomics evidence from osteoarthritis-relevant tissues and identify effector genes that converge on key biological processes underpinning disease development, generating insights into targets for focused therapeutic interventions.

## Study overview

We have performed a large multi-ancestry GWAS meta-analysis for osteoarthritis, combining 87 datasets across 489,975 cases and 1,472,094 controls, with an effective sample size of 1,470,467 individuals (Methods and Supplementary Table 1). It includes 87.31% individuals of European (EUR) ancestry, 7.09% East Asian (EAS) ancestry, 3.08% African American (AFR) ancestry, 1.09% South Asian (SAS) ancestry, 0.91% Hispanic (HIS) ancestry and 0.53% with mixed ancestry (ADM) (Supplementary Tables 1 and 2). In addition to osteoarthritis at any joint as an overarching disease phenotype, we performed joint-specific GWAS meta-analyses on the basis of the joint affected (Methods).

## Genetic architecture of osteoarthritis

**Fig. 1:**
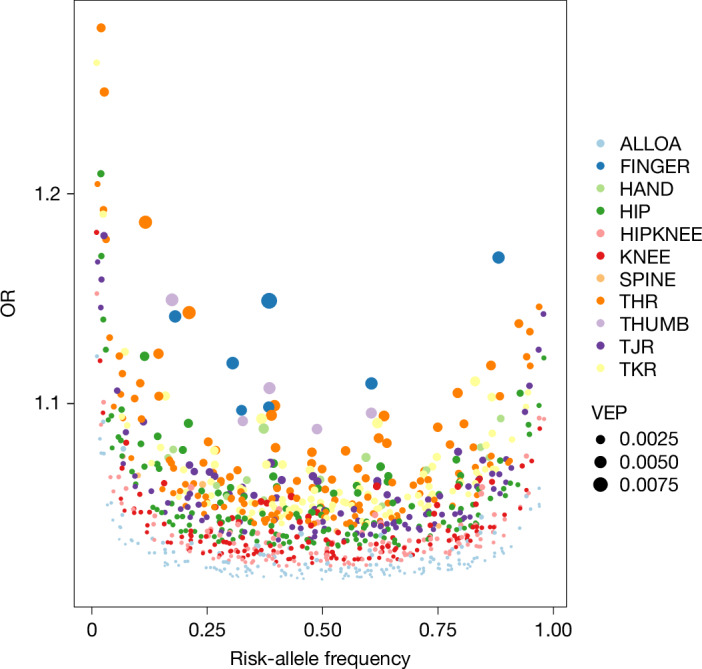
The genetic architecture of osteoarthritis. Meta-analysis-based odds ratios of the 962 index variants as a function of their risk-allele frequency, and phenotypic variance explained (VEP) for each variant indicated by the size of each circle. Each colour corresponds to an osteoarthritis phenotype: osteoarthritis at any site (ALLOA), hip osteoarthritis (HIP), knee osteoarthritis (KNEE), hip and/or knee osteoarthritis (HIPKNEE), spine osteoarthritis (SPINE), hand osteoarthritis (HAND), finger osteoarthritis (FINGER), thumb osteoarthritis (THUMB), total hip replacement (THR), total knee replacement (TKR) and total hip and/or knee replacement (total joint replacement, TJR).

We identified 962 independent osteoarthritis associations at the study-wide significance threshold of *P* ≤ 1.3 × 10^−8^ (175 for osteoarthritis at any site, 151 for hip osteoarthritis, 146 for knee osteoarthritis, 131 for hip and/or knee osteoarthritis, 4 for spine osteoarthritis, 14 for hand osteoarthritis, 7 for finger osteoarthritis, 5 for thumb osteoarthritis, 136 for total hip replacement, 92 for total knee replacement and 101 for total joint replacement) (Fig. 1, Supplementary Figs. 1–3 and Supplementary Table 3), some of which overlap across phenotypes. The majority of these (513 out of 962) are conditionally independent of any previously reported risk variant for any osteoarthritis phenotype (Supplementary Tables 3 and 4). Of the 962 variants, 339 are unique and conditionally independent across all osteoarthritis phenotypes (236 newly reported here) (Methods).

The 962 independently associated variants map to 286 genomic loci (176 newly reported here). Of the 110 previously reported loci, 44 have a newly reported, independent osteoarthritis-associated variant (Methods and Supplementary Tables 3 and 4). Most loci (86%) contain a single independent signal, with the remainder encompassing between 2 and 5 independent signals per locus. 95% of the associated variants have a minor allele frequency (MAF) of ≥5% with small to modest effects (odds ratios (OR), 1.016–1.186). Forty-nine signals are driven by low-frequency variants (MAF, 1–5%; OR, 1.044–1.279) (Fig. 1 and Supplementary Fig. 1). We performed GWAS meta-analysis within four ancestry groups (EAS, AFR, SAS, HIS) and did not detect ancestry-specific study-wide significant associations. We also did not find any additional signals when restricting the GWAS meta-analysis to studies in which osteoarthritis had been defined based on imaging. We find high correlation between associations when comparing GWAS with and without the inclusion of self-reported osteoarthritis (Methods, Supplementary Figs. 4–6 and Supplementary Note).

In addition to the 339 unique signals from the main analyses, we find 3 newly reported female-specific associations and 1 male-specific association with significant differences in effect size between sexes (*P*_het_ < 0.0125) that did not reach genome-wide significance in the combined sex analysis (Methods and Supplementary Tables 5 and 6).

We evaluated the predictive potential of genetic risk scores (GRSs) in independent datasets (Methods). For the osteoarthritis phenotypes tested, no analysis reached an area under the receiver operating characteristic curve (AUC) over 80%. The best-performing GRS was obtained for hip osteoarthritis (AUC, 58.6%) (Supplementary Table 7). We found that including body–mass index (BMI) in the GRS model led to improvements in prediction (for example, hip osteoarthritis including BMI AUC, 66%).

## Signal enrichment in skeletal cell types

**Fig. 2:**
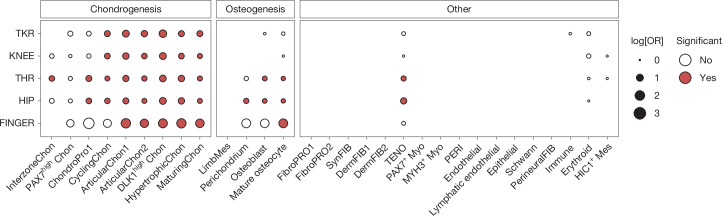
Signal enrichment in cell types associated with skeletal development. fGWAS enrichment for osteoarthritis in 30 cell states of the skeletal development atlas. Significance (FDR < 0.1) and effect size (log-transformed OR, log[OR]) are indicated by colour and dot size, respectively. InterzoneChon, interzone chondrocytes; PAX7^high^ Chon, PAX7-expressing chondrocytes; ChondroPro1, chondrocyte progenitors; CyclingChon, chondrocytes with high cell cycle activity; ArticularChon1, articular chondrocytes with high TRPV4 and VEGFA expression; ArticularChon2, articular chondrocytes with high EPYC and low SOX9 expression; DLK1^high^ Chon, DLK1-expressing chondrocytes; HypertrophicChon, hypertrophic chondrocytes; MaturingChon, maturing chondrocytes; LimbMes, early limb mesenchyme cells; Perichondrium, perichondrial osteoblast progenitors; MatureOsteocyte, osteocytes; FibroPRO1/2, fibroblast progenitors; SynFIB, synovial fibroblasts; DermFIB1/2, dermal fibroblasts; TENO, tenocytes; PAX7^+^ Myo, PAX7-expressing myocytes; MYH3^+^ Myo, MYH3 expressing myocytes; PERI, pericytes; PerineuralFIB, perineural fibroblasts; HIC1^+^ Mes, HIC1-expressing mesenchymal cells.

To determine whether early development of skeletal tissues contributes to the risk of osteoarthritis later in life, we investigated the enrichment of GWAS signals in cell types associated with skeletal development through functional GWAS (fGWAS). We performed the analysis for 30 different cell types using single-cell multiomics data (ATAC and RNA-seq) from the human skeletal development atlas^4^, spanning 5–11 weeks after conception (Fig. 2, Methods and Supplementary Table 8).

In the chondrogenesis lineage, we observed significant enrichment (false discovery rate (FDR) < 0.1) for mature, hypertrophic, articular and DLK1-expressing chondrocytes for all of the tested osteoarthritis phenotypes, consistent with cartilage being the primary affected tissue. Chondrocytes with high cell cycle activity were also enriched for all phenotypes except for finger osteoarthritis. Moreover, more immature cell types including chondrocyte progenitors and early *GDF5* expressing interzone chondrocytes were enriched for total hip replacement, and chondrocyte progenitors were also enriched for hip osteoarthritis. In the osteogenesis lineages, we observed significant enrichment for mature osteocytes (total hip replacement, hip osteoarthritis and finger osteoarthritis), osteoblast (total hip replacement and hip osteoarthritis) and perichondrium (hip osteoarthritis).

The osteoblast enrichment associated with hip and finger osteoarthritis may be linked to bone morphology, as structural abnormalities in femoral head formation can lead to irregular joint surfaces or improper joint congruity, increasing the risk of mechanical overloading, and contributing to osteoarthritis development. Geometric parameters of the hip are known to be associated with osteoarthritis^5,6^, and developmental dysplasia of the hip often leads to osteoarthritis, with research showing shared genetic risk factors between the two conditions, including associations with *GDF5* and *COL11A1*^7,8^. Finger-length patterns in combination with elevated androgen levels during development have also been linked with osteoarthritis^9^. The fGWAS results therefore suggest a role of bone development in the pathogenesis of hip and finger osteoarthritis manifesting in later stages of life and implicates particular transcriptomic and epigenetic cell states.

We find enrichment in total hip replacement and hip osteoarthritis genetic associations with tenocytes. Tendons are vital to the transmission of force and stabilization of the musculoskeletal system. Hip tendon samples from patients with osteoarthritis demonstrate a greater degree of fibrosis, non-collagenous change and calcium deposition in the extracellular matrix (ECM) compared with samples from patients with femoral neck fractures^10^, consistent with periarticular tendinopathy. Similar tendinopathy is found at other osteoarthritis-susceptible joints^11,12^. Our findings indicate that tendon development is also associated with hip osteoarthritis and is more likely related to late-stage osteoarthritis, suggesting that the developmental biology of secondary stabilizers of the joint contributes to the causal pathway in osteoarthritis.

## Fine mapping of causal variants

To identify potential causal variants at the associated loci, we created, at each signal, a set of variants that are predicted with 95% probability to include a causal variant, called credible sets (Methods and Supplementary Note). The number of variants in a credible set ranged from 1 to 247 (mean 23 variants) with 75 credible sets containing a single variant and 149 credible sets containing less than 3 variants (Supplementary Tables 9 and 10). A total of 328 credible sets mapped entirely within the transcript of a single gene, strongly indicating that gene as causal. Most credible-set variants were predicted to be non-coding (57% were intronic and 17% intergenic). In total, 81 coding credible-set variants were missense, 1 was a stop gain variant (in *VIT*) and 1 was a splice acceptor variant. On the basis of 3D chromatin interaction data that we generated in primary osteoarthritis chondrocytes (Methods), 187 credible-set variants overlap promoters, 2,149 overlap enhancers and 814 reside within an enhancer that loops to a promoter. We performed transcription factor enrichment analysis (Methods) and identified 1,585 credible-set variants that both reside within gene regulatory regions and affect a transcription-factor-binding motif in osteoblast or chondrogenic cells (344 unique transcription factors; Supplementary Tables 11 and 12, Supplementary Fig. 7 and Supplementary Note).

## Identification of effector genes

**Extended Data Fig. 1:**
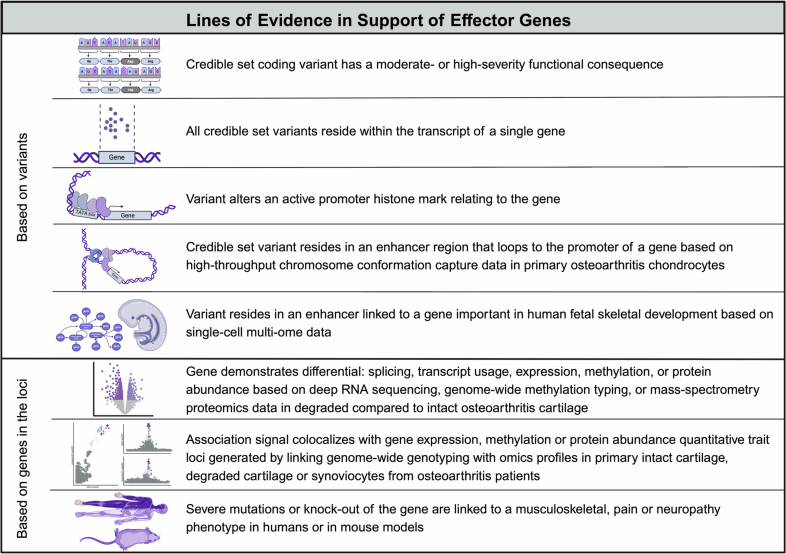
Lines of evidence used to identify effector genes. Created in BioRender. Southam, L. (2025) https://BioRender.com/d58k400.

To identify genes that are very likely to be causal for osteoarthritis (effector genes), we integrated data across 24 orthogonal lines of evidence to score each of the 8,785 genes residing within the 286 genomic risk loci (Methods, Extended Data Fig. 1 and Supplementary Tables 13–19). We identified 700 unique effector genes with a score of ≥3, mapping to over 88% of loci (Supplementary Table 20). We find that 70 loci contain a single effector gene, while the majority (70%) contain more than one gene with at least three orthogonal lines of evidence pointing to its involvement. The highest-scoring effector gene, with 11 lines of evidence in support of its involvement, is *ALDH1A*2, a gene previously implicated in osteoarthritis^13^.

**Extended Data Fig. 2:**
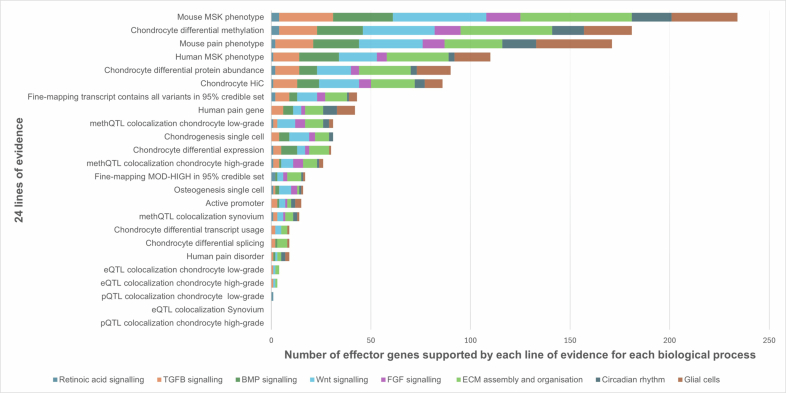
Contribution of the 24 lines of evidence to each biological process. Each bar represents the cumulative absolute number of effector genes identified in the 24 lines of evidence, supporting each of the 8 highlighted biological processes, as indicated by the colour coding. The lines of evidence are ranked, with those contributing the most displayed at the top. MSK: musculoskeletal. MOD: moderate.

We found that mouse and human musculoskeletal and pain phenotypes, chondrocyte HiC and differential chondrocyte methylation are the lines of evidence with relatively higher information contributions (Methods, Supplementary Tables 13, 21 and 22, Supplementary Fig. 8, Extended Data Fig. 2 and Supplementary Note).

## Deleterious rare variant burdens

We assessed the association between loss of function (LOF) variants in the 700 effector genes and osteoarthritis using gene burden tests. To this end, we aggregated the association of all rare LOF variants in these genes (<2% frequency total) (Methods and Supplementary Tables 23–25) and identified nine study-wide significant associations (*P* < 7.1 × 10^−5^) with 5 genes (*ADAMTSL3*, *VIT*, *COL27A1*, *IL11* and *PMVK*), of which the burdens of *ADAMTSL3* and *VIT* on hip osteoarthritis and total hip replacement are genome-wide significant (*P* < 2.5 × 10^−6^). The risk of disease was increased for LOF variants in these genes. When we incorporated missense (MIS) in addition to LOF variants (LOF + MIS) in the burden tests, we identified *ADAMTSL3*, *VIT*, *IL11*, *THBS3*, *ADAMTS6*, *SPRY2* and *COLGALT2* associated with osteoarthritis, of which association of *ADAMTSL3* with hip osteoarthritis and *IL11* with total hip replacement are genome-wide significant. LOF + MIS variants in *ADAMTS6*, *SPRY2* and *COLGALT2* are protective against osteoarthritis, whereas aggregation of these variants in *ADAMTSL3*, *VIT*, *IL11* and *THBS3* confer risk of osteoarthritis. The direction of effects was consistent in both models for all effector genes. Common non-coding sequence variants associated with osteoarthritis phenotypes present concordant directions of effect with gene-burden association results of genes in their vicinity, with the exception of variants near *THBS3* and *PMVK*; these two genes are at the same locus (around 300 kb apart). Notably, none of the above burden associations are driven by a single variant in any of the cohorts (Supplementary Table 25).

We found LOF burdens for genes at the same loci as those identified in the common variant analysis for the same phenotypes and for different phenotypes (for example, *ADAMTSL3* and total hip replacement, *PMVK* and knee osteoarthritis, and *SPRY2* and hand osteoarthritis). We also detected LOF burdens for different genes at the same locus (*PMVK* and *THBS3*). For the same phenotype, the effect sizes in the LOF burden analysis are consistently larger compared with those identified in the common variant analysis, except for *VIT*, for which they are the same.

## Biological Insights

**Extended Data Fig. 3:**
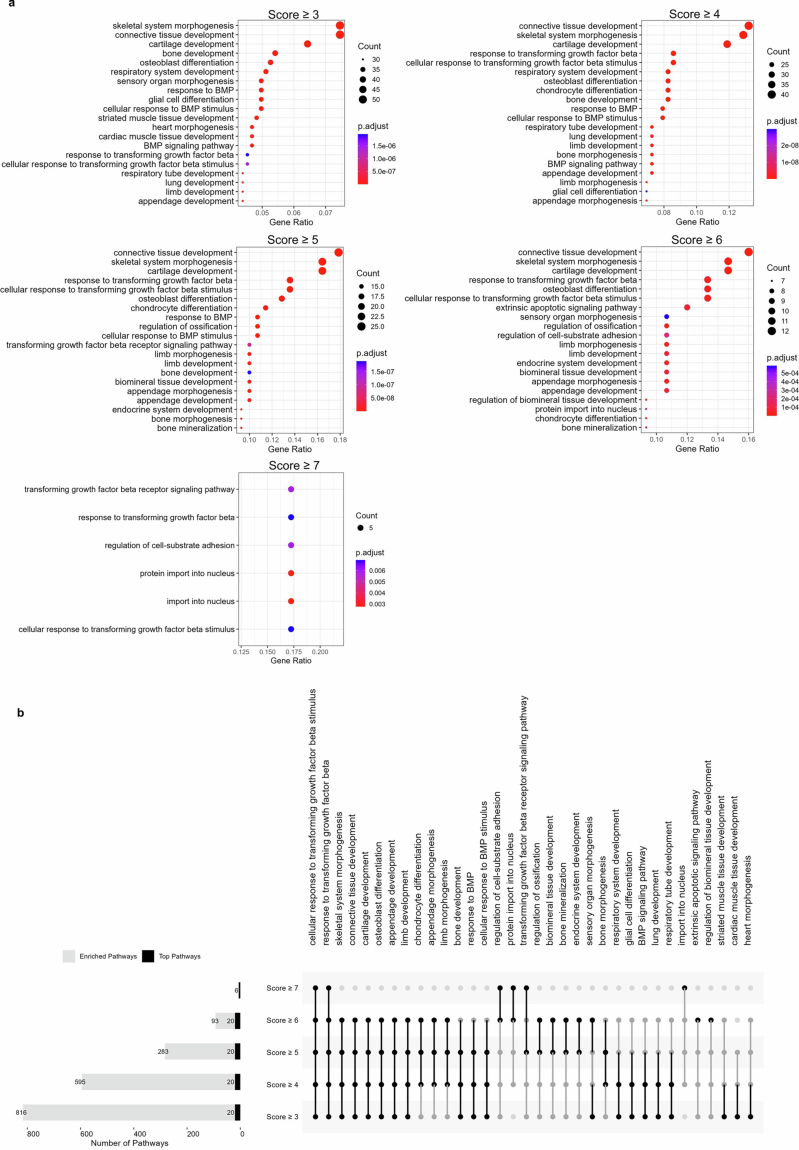
Gene Ontology over-represented pathways involved in osteoarthritis pathogenesis. **(a)** Top 20 overrepresented pathways enriched by effector genes with scores across 3 to 6 and all over-represented pathways enriched by effector genes with score 7 (P.adjust was calculated by two-tailed hypergeometric test and corrected by multiple testing of Benjamini-Hochberg). The colour scale represents scaled adjusted P value. The exact adjusted P values are provided in Supplementary Table 26. The bubble size was scaled based on the counts of genes enriched for each pathway. Top pathways were ranked based on the gene ratio which was calculated by counts of enriched genes divided by the total number of genes that can be found in the background gene set within each score. **(b)** Upset plot illustrating the connection of top 20 pathways across gene sets with scores ≥3 to ≥7. Black dots and lines represent inclusion in the top 20 pathways and dark grey dots and lines represent over-represented but not in the top 20 rank, pathways were ranked by gene ratio. The bar plot displays the total number of over-represented pathways and the fraction of top 20 pathways within each gene score set.

We identify eight interconnected biological pathways that are enriched for effector genes, the majority of which are newly reported here (Table 1, Methods, Supplementary Note, Supplementary Tables 13 and 26–29 and Extended Data Fig. 3; a detailed description of these pathways and the role of the effector genes is provided in the Supplementary Note). We find that the biological processes with the highest number of effector genes, such as ECM and WNT signalling, show higher levels of osteoarthritis heritability explained (Supplementary Fig. 9).

**Table 1:** Distribution of effector genes across the eight highlighted pathways

Pathway	*n* effector genes	*n* novel effector genes	*n* effector genes targeted by approved drugs	Median *n* of risk loci carried by patients in the UKBB/MVP		
				ALLOA	KNEE	HIP
Retinoic acid signalling	4	3	0	4/4	1/1	1/1
TGFβ signalling	28	19	6	21/22	9/11	16/15
BMP signalling	33	28	3	32/31	12/13	15/14
WNT signalling	57	49	5	44/43	15/16	24/24
FGF signalling	20	15	3	17/17	8/8	11/11
ECM assembly and organization	61	48	18	39/38	18/18	21/21
Circadian rhythm	20	18	6	14/14	7/7	5/5
Glial-cell-related pathways	39	35	10	31/31	12/13	17/17

UKBB, UK Biobank; MVP, Million Veteran Program.

### Retinoic acid signalling

**Extended Data Fig. 4:**
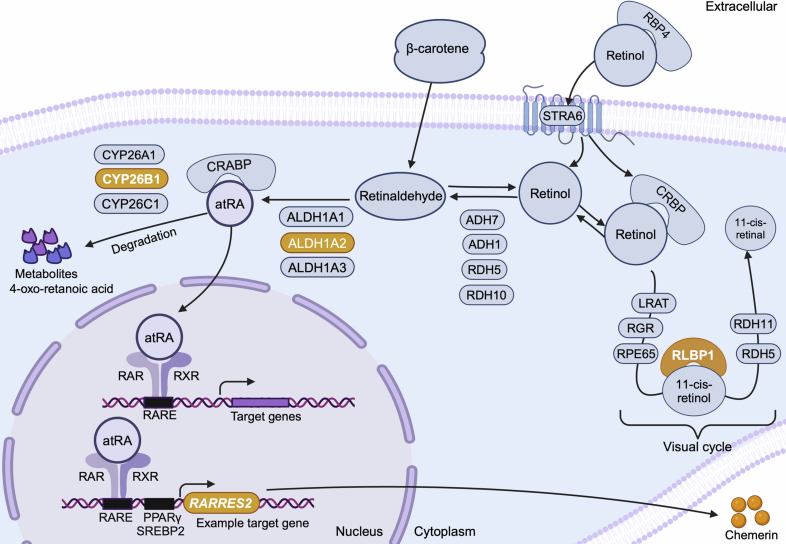
Effector genes associated with the retinoic acid pathway. Effector genes are highlighted in orange with white text, bold indicates a newly-discovered effector gene. Created in BioRender. Southam, L. (2025) https://BioRender.com/z16x054.

The retinoic acid signalling pathway (Extended Data Fig. 4) is associated with the highest-scoring effector gene, *ALDH1A2*. ALDH1A2 catalyses the synthesis of all-trans retinoic acid (ATRA), which then interacts with retinoic acid and retinoid acid receptors, regulating the expression of multiple genes with fundamental roles in skeletal patterning and differentiation^14,15^, as well as organ and limb development^16,17^. *CYP26B1* is involved in the degradation of ATRA, thereby controlling its availability. The balance of synthesis and degradation of ATRA is important for receptor interactions, and depletion or excess of ATRA can result in developmental abnormalities^18^.

### TGFβ signalling

**Extended Data Fig. 5:**
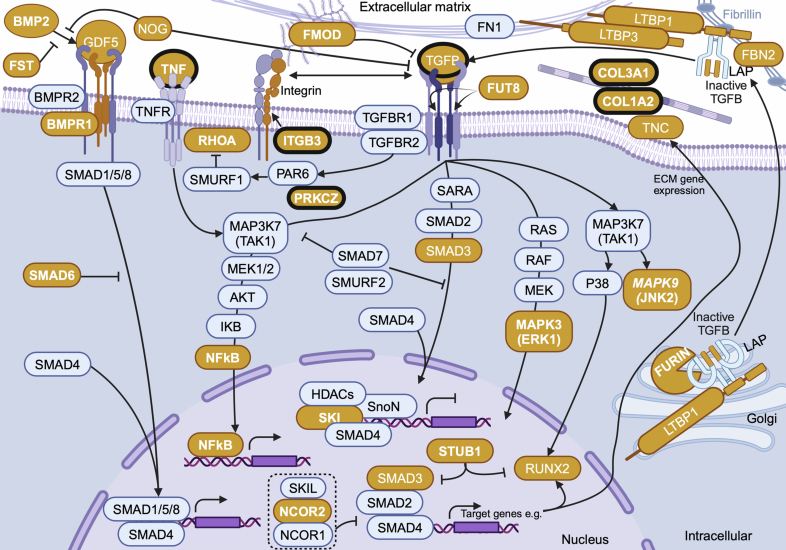
Effector genes associated with TGFB signalling. Effector genes are highlighted in orange with white text, bold indicates a newly-discovered effector gene. A bold outline indicates an effector gene whose protein is the target of an approved drug. Created in BioRender. Southam, L. (2025) https://BioRender.com/x47k064.

TGFβ signalling (Extended Data Fig. 5) is intricately involved in the pathogenesis of osteoarthritis through its effects on chondrocyte and osteoblast differentiation, skeletal development, cartilage and bone formation, inflammation, ECM remodelling, osteophyte and synovial tissue changes, and interactions with other signalling pathways, such as BMP. The identified effector genes traverse all aspects of TFGβ signalling (Extended Data Fig. 5). We find that *TGFB1* and *SMAD6* demonstrate allelic imbalance in subchondral bone (Methods, Supplementary Table 27 and Supplementary Fig. 10) and that the osteoarthritis risk allele of rs146652543 is associated with decreased expression of *TGFB1*. We also identify decreased protein abundance of TGFβ1 in degraded compared with intact osteoarthritis cartilage (Supplementary Table 13). The hip osteoarthritis risk-increasing allele of rs2469081 is associated with decreased expression of *SMAD6*, a newly identified signal. Furin plasma protein quantitative trait loci (pQTLs) colocalize with osteoarthritis signals on chromosome 15 (rs1894401) (Methods and Supplementary Table 28).

### BMP signalling

BMP signalling has an important role in many organs and tissues during early embryogenesis (dorsoventral and anteroposterior axis formation), and in postnatal homeostasis. The role of BMP signalling in skeletal development and maintenance is well established, with a lack or excess of BMP signalling giving rise to skeletal abnormalities. Mutations and/or deletion of the effector genes *BMP2*, *BMP6*, *BMPR1B*, *GDF6* and *GDF5* have been associated with brachydactyly (*BMP2*, *BMPR1B* and *GDF5*)^19–21^, joint deformities and osteoarthritis (*GDF5*)^22^, reduction in long bone size (*BMP6*)^23^, joint defects (*GDF5* and *GDF6*) and severe chondrodysplasia (*BMP2*)^24^. The mechanisms of involvement of BMP signalling with osteoarthritis pathology are complex, ranging from embryonic and developmental changes to those that occur throughout life, such as cartilage homeostasis, osteophyte formation and subchondral bone changes.

### WNT signalling

WNT signalling has an important function in bone and cartilage metabolism and a well-established role in osteoarthritis^25^. Two of the effector genes involved in this pathway are WNT family members (*WNT3* and *WNT5a*), both newly reported here, and the remaining genes are involved in modulation of the WNT signalling pathway. WNT signalling has an essential role in embryonic development and homeostasis of bone and cartilage. Dysregulated WNT signalling can contribute to various aspects of osteoarthritis pathology, including cartilage degradation, subchondral bone changes, synovial inflammation and osteophyte formation. We find that the hip osteoarthritis risk allele of rs77601616 is associated with increased expression of *SFRP4*, located at a locus newly discovered here, in subchondral bone (Methods, Supplementary Table 27 and Supplementary Fig. 10).

### Fibroblast growth factor signalling

Members of the fibroblast growth factor (FGF) pathway have been implicated in the pathogenesis of osteoarthritis through skeletal development, bone and cartilage homeostasis, and also through inflammation and angiogenesis. Five of the effector genes involved in FGF signalling are key FGF pathway members (*FGF1*, *FGF18*, *FGFR3*, *FGFR4* and *FGFRL1*). FGFs have an important role in tissue regeneration and repair and are integral to cell differentiation, proliferation, apoptosis, metabolism, morphogenesis and tissue healing. Two FGF-related pathways involve a further 18 effector genes: FGFR3 signalling in chondrocyte proliferation and terminal differentiation (10 effector genes), and osteoarthritic chondrocyte hypertrophy (16 effector genes) (Supplementary Table 29 and Supplementary Fig. 11). Mutations in *FGFR3* are known to give rise to achondroplasias^26^. Osteoarthritic chondrocyte hypertrophy is associated with dysregulation of FGF, hypoxia and angiogenesis^27^.

### ECM

Among the 61 effector genes associated with ECM assembly and organization, 14 are collagens, 3 are proteoglycans, 12 are glycoproteins, 6 are ECM secreted factors, 7 are ECM regulators and 1 is an ECM-affiliated protein. The majority of the ECM in healthy articular cartilage is composed of aggrecan, encoded by *ACAN*, and collagen type II, encoded by *COL2A1*, both newly reported effector genes. Mutations in both *COL2A1* and *ACAN* give rise to types of spondyloepiphyseal dysplasia characterized by premature osteoarthritis^28^. During osteoarthritis progression, the balance between the aggrecan content (which provides the ability to withstand compression and absorb shocks) and collagen content (which provides tensile strength) is critical. Changes in ECM content can give rise to reduced mechanical strength, lack of elasticity and increased susceptibility to damage. We find further support for the involvement of *COL2A1* for the association signal at rs11168351, which colocalizes with COL2A1 plasma pQTLs (Supplementary Table 28). The pericellular matrix, which surrounds the chondrocyte and modulates the environment, is enriched for collagen type VI (*COL6*) and perlecan (*HSPG2*). COL6 is encoded by six genes, two of which are effector genes (*COL6A1* and *COL6A2*). Mutations in *COL6A1/2* are associated with various myopathies^29^. Mutations in *HSPG2*, which is also an effector gene, give rise to Schwartz–Jampel syndrome type 1, characterized by myotonia and chondrodysplasia^30^. Two genes involved in the ECM also harbour LOF burdens (*COLGALT2* and *COL27A1*). The LOF + MIS burdens in *COLGALT2* are protective against osteoarthritis (Supplementary Table 23). *COLGALT2* encodes an enzyme that is involved in the post-translational glycosylation of collagens and proteins containing collagen domains. Differential allelic expression imbalance between intact and degraded cartilage has shown that lower expression of COLGALT2 is protective for osteoarthritis^31^. In osteoarthritic cartilage, the risk allele of rs11583641 was associated with increased expression of *COLGALT2* mediated through decreased methylation^32^. Mechanistically, over-glycosylation may result in weakened integrity of collagen fibrils and decreased resilience of the cartilage. The risk of disease was increased for LOF variants in *COL27A1*, which is a fibril-forming collagen with a role in the transition of cartilage to bone during skeletogenesis. *COL27A1* has been shown to be regulated by *SOX9* (an effector gene). Mutations in *COL27A1* are associated with Steel syndrome, characterized by short stature, hip dislocation and scoliosis^33,34^.

### Circadian rhythm

**Extended Data Fig. 6:**
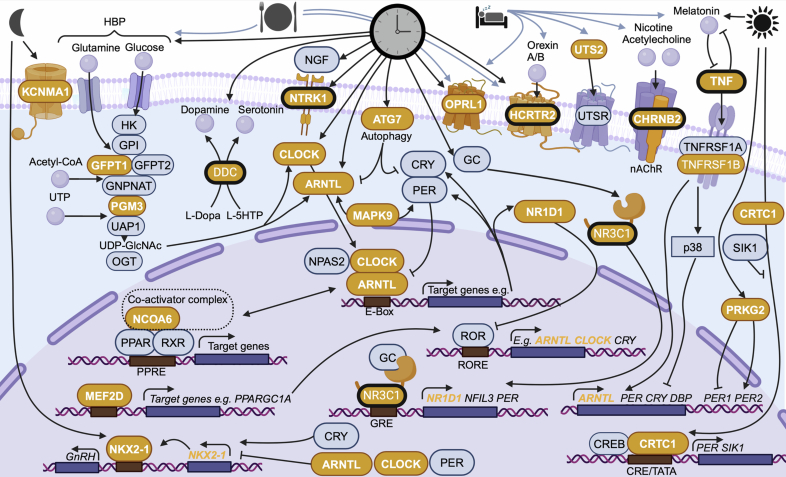
Effector genes associated with the circadian clock. Effector genes are highlighted in orange with white text, bold text indicates a newly-discovered effector gene. A bold outline indicates an effector gene whose protein is the target of an approved drug. Created in BioRender. Southam, L. (2025) https://BioRender.com/h77m147.

The circadian rhythm has not been genomically linked with osteoarthritis, although a few studies have established a role for circadian clocks in articular cartilage in regulating pathways related to tissue ageing, degeneration and osteoarthritis. It has also been demonstrated that chronic circadian misalignment may accelerate tissue ageing and ECM degradation. Furthermore, changes in tissue stiffness, for example during ageing, can impair circadian clock function^35–37^. A subpopulation of chondrocytes has also been shown to have increased expression of circadian-related genes (*PER1* and *SIRT1*)^38^. Disruptions to circadian rhythms may affect the ability of bone and joint tissues to repair and regenerate. Morning joint stiffness can occur due to circadian variations, and age-related changes in sleeping patterns can decrease the amplitude of circadian rhythms. Circadian rhythms can also influence pain perception and sensitivity^39^, and the absorption, distribution and metabolism of drugs. Circadian-related pain perception has been observed in individuals with osteoarthritis of the knee and hand^40,41^. Effector genes implicated in this biological process are core circadian clock components (*CLOCK*, *ARNTL* and *NR1D1*), involved in clock entrainment, orchestration, sleeping patterns, transcription of clock genes, circadian oscillations and/or clock-controlled autophagy in bone metabolism (Extended Data Fig. 6 and Supplementary Fig. 12). We find that *GFPT1*, linked with clock entrainment, demonstrates allelic imbalance in subchondral bone; and that the hip osteoarthritis risk allele of rs6546511 is associated with increased *GFPT1* expression (Methods, Supplementary Table 27 and Supplementary Fig. 10). We also find a decrease in PTGS1 in degraded compared with in intact osteoarthritis-affected chondrocytes (Supplementary Table 13).

### Glial-cell-related pathways

Glial cells provide structural and functional support to neurons, regulate the extracellular environment and have crucial roles in immune defence and repair processes within the nervous system. The 39 effector genes associated with glial cells traverse multiple cellular processes such as cell differentiation, regulation, migration and development. Glial cells may have a multifaceted role in the pathophysiology of osteoarthritis, influencing immune response, neuroinflammation, neuronal plasticity, peripheral and central sensitization. Strategies aimed at modulating glial-mediated mechanisms could provide new therapeutic options for alleviating pain and inflammation associated with osteoarthritis.

## Drug targets

**Extended Data Fig. 7:**
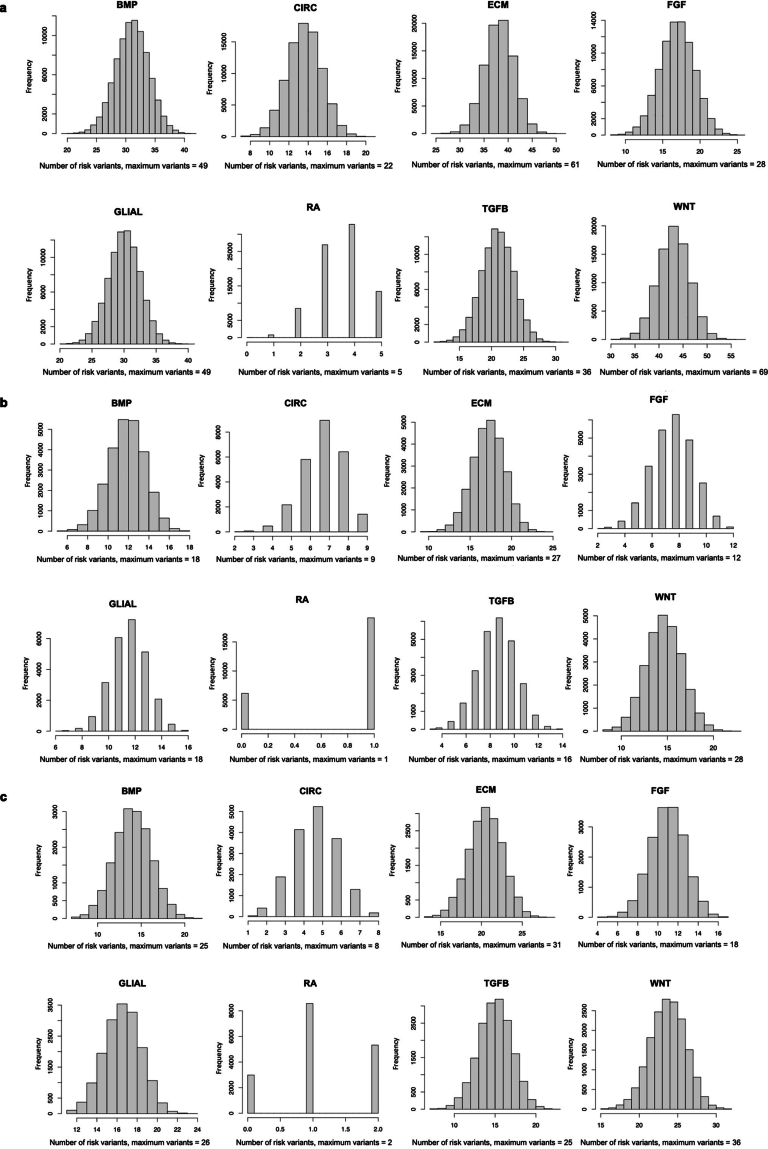
Number of risk alleles carried by UK Biobank osteoarthritis patients in each pathway. Distribution of risk alleles carried by UK Biobank patients with **(a)** osteoarthritis at any site (n = 82,420), **(b)** knee osteoarthritis (n = 25,293) and **(c)** hip osteoarthritis (n = 16,876). The pathways are represented by: RA, retinoic acid signalling; TGFB, TGFB signalling; BMP, BMP signalling; WNT, Wnt signalling; FGF, FGF signalling; ECM, ECM assembly and organization; CIRC, circadian rhythm and GLIAL, glial cell related. On the x-axis the maximum number of variants included in the analysis is provided.

**Extended Data Fig. 8:**
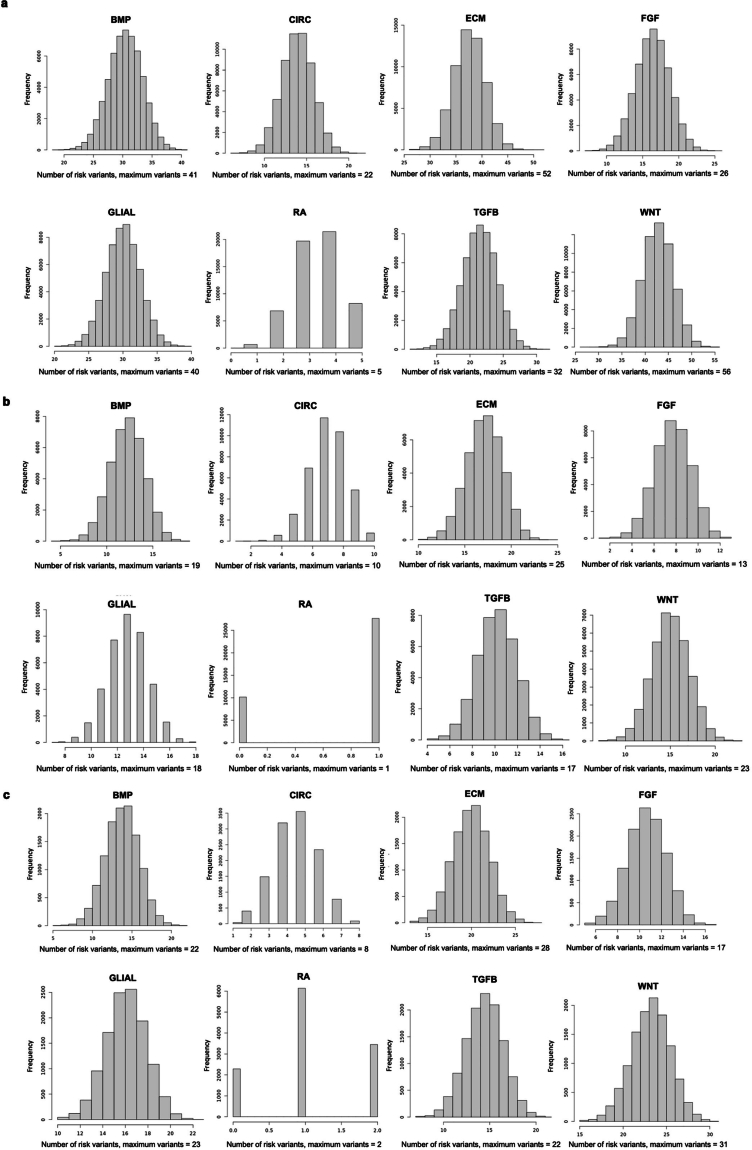
Number of risk alleles carried by Million Veteran Program osteoarthritis patients in each pathway. Distribution of risk alleles carried by the Million Veteran Program patients with (**a**) osteoarthritis at any site (n = 56,848), (**b**) knee osteoarthritis (n = 37,814) and (**c**) hip osteoarthritis (n = 11,873). The pathways are represented by: RA, retinoic acid signalling; TGFB, TGFB signalling; BMP, BMP signalling; WNT, Wnt signalling; FGF, FGF signalling; ECM, ECM assembly and organization; CIRC, circadian rhythm and GLIAL, glial cell related. On the x-axis the maximum number of variants included in the analysis is provided.

We identify 473 approved drugs that target the protein product of 69 effector genes, of which 5 (7.2%) have been previously associated with a pain phenotype (Methods and Supplementary Tables 13, 30 and 31). Over half of these genes (37) are members of one or more of the eight highlighted pathways (Table 1 and Supplementary Note). Genetically informed selection of patients carrying risk alleles mapping to pathways targeted by drugs has the potential to pave the way for personalized medicine and the smart design of clinical trials going forward (Table 1, Supplementary Tables 32 and 33, Extended Data Figs. 7, 8 and Supplementary Note).

*CYP26B1* of the retinoic pathway is involved in the degradation of ATRA, thereby controlling its availability, and is inhibited by taralazole, which is currently undergoing a proof-of-concept trial to treat patients with base of thumb osteoarthritis before surgery (https://www.isrctn.com/ISRCTN16717773).

FGF18, a high-affinity ligand for FGFR3 and a member of the FGF pathway, is currently being investigated in clinical trials for osteoarthritis, in which sprifermin (human recombinant FGF18) injected into joints has shown promising results in terms of improving cartilage thickness and reducing symptoms of osteoarthritis over a 5-year follow-up in patients with knee osteoarthritis^42^.

There are six effector genes that are linked with the TGFβ pathway (*TGFB1*, *COL1A2*, C*OL3A1*, *TNF*, *PRKCZ* and *ITGB3*), for which their protein is the target of at least one approved drug (Supplementary Table 30). These drugs are used to treat a variety of conditions: involving the immune system and inflammation, abnormalities of connective tissue and Dupuytren’s contracture, myocardial infarction and recurrent thrombophlebitis, neoplasms and anaemia.

*SOST* antagonizes both WNT and BMP signalling. Its encoded protein (sclerostin) is inhibited by romosozumab, a monoclonal anti-sclerostin antibody, used to increase bone mass and treat osteoporosis. Four additional WNT signalling genes, *PSMB8*, *TGFB1*, *PSMC3* and *COL6A1*, are targeted by approved drugs, with the latter also a part of the ECM and glial cell pathway.

Eighteen effector genes involved in the ECM have proteins that are the target of approved drugs (Supplementary Table 30). Two approved drugs (ocriplasmin and collagenase clostridium histolyicum) target ten of the collagen effector genes and are licenced for abnormalities of connective tissue, macular degeneration and Dupuytren’s contracture among other indications. For osteoarthritis, the target site here might be the joint capsule or synovium, rather than the articular cartilage.

Agonists of the glucocorticoid receptor, the gene product of *NR3C1*, a member of both the circadian clock and glial cell pathways, are approved for osteoarthritis pain relief, due to its anti-inflammatory ability. Using an individual’s circadian rhythm may improve outcomes by maximizing therapeutic efficacy, decreasing adverse effects and personalizing disease management accordingly. Indeed, the efficacy in the treatment of osteoarthritis pain with the non-steroidal anti-inflammatory drug indomethacin has been shown to be contingent on the timing of drug administration^43^. Indomethacin, among other 44 approved drugs, targets *PTGS1*, also known as *COX1*. Treatment with naproxen (one of the approved drugs) suppressed *PTGS1* expression in synovial tissue, inhibited the migratory and invasive capabilities of osteoarthritis synoviocytes and increased their apoptosis rate^44^.

## Discussion

Osteoarthritis is one of the leading causes of disability and pain worldwide^1^. The societal and public health burden of osteoarthritis is enormous and is accompanied by substantial multimorbidity^45^ and significant cost. For example, in the US alone, the total costs attributed to osteoarthritis averaged US$486.4 billion annually^46^, and in Europe the respective annual costs for knee and hip osteoarthritis are up to €817 billion^47^. No effective disease-modifying treatments exist for osteoarthritis. A better understanding of the biological processes leading to disease development is therefore urgently needed to improve the lives of the staggering number of people with osteoarthritis worldwide.

Here we conducted a GWAS meta-analysis for osteoarthritis with a substantial step-up in sample size and power (2.76-fold increase in the number of patients with osteoarthritis included compared with the next largest GWAS^3^). Although we have achieved an improvement in the genetic diversity of contributing populations (87% European ancestry compared with 97% in the next largest GWAS^3^), there is a clear need to continue efforts in identifying and including cohorts that better reflect genetic diversity globally. In this study, we did not achieve the power required to glean whether non-EUR ancestry-specific signals exist.

Osteoarthritis exhibits discordance between structural changes and symptoms. We find no additional signals when restricting the analyses to imaging-based disease definitions only, although this could be ascribed to relatively lower power. Sensitivity analyses confirm previous reports on the suitability of using self-report in osteoarthritis for genetic studies^48^. We also acknowledge the complexities in differentiating spinal osteoarthritis from other structural abnormalities, such as disc disease and compressive neuropathies. In this work, all four spine osteoarthritis signals demonstrate associations with other osteoarthritis joint phenotypes. Going forward, comparative studies with more precise diagnostic criteria are warranted.

Our findings provide insights into the genetic architecture of disease, with 70% of the unique study-wide significant variants and 62% of loci not having been reported previously. In addition to these, mainly common-frequency, modest-effect variant associations, we identify rare coding-variant burdens with consistently higher effect sizes. Here we have restricted the LOF burden analysis to effector genes at common-variant loci. Moving forward, and with increasing sequencing data availability, it appears likely that we will identify additional loci using LOF burden analysis that are not captured by common variation. Such analyses may identify novel genes and pathways with more profound effects at the protein level for therapeutic targeting.

By generating and integrating molecular profile data in primary osteoarthritis tissue and incorporating additional lines of evidence, coupled with a deep literature dive, we identify 700 effector genes, increasing the number of effector genes for osteoarthritis by an order of magnitude, and provide insights into the biology of disease. Musculoskeletal and pain phenotypes, along with chondrocyte data, are the lines of evidence with relatively higher information contributions. We identify signal enrichment in embryonic skeletal development pathways and highlight eight biological processes in which we find convergence of effector genes. The overlap of genes across multiple biological processes, suggests that these pathways interact to affect osteoarthritis development and progression.

We find that risk allele carriage is pervasive across patients with osteoarthritis for all eight biological processes, potentially facilitating patient selection for clinical trials.

Drug targets supported by human genetics evidence are 2.6 times more likely to progress further in clinical trials and gain approval^49^. We find that approximately 10% of the effector genes express a protein that is the target of approved drugs. Identification of genetic evidence of osteoarthritis risk for targets of already approved drugs opens up an opportunity for repurposing of these drugs for osteoarthritis, which can greatly accelerate the translation pathway. Likewise, prolonged use of some of these drugs may also increase the risk of osteoarthritis, depending on the directionality of effects.

In conclusion, our findings demonstrate the value of integrating large-scale GWAS meta-analysis with functional genomics data across relevant disease tissues to enhance our understanding of complex disease aetiopathology. Going forward, congruent with the aspiration of enhancing genetic diversity in the GWAS meta-analysis, the generation of functional genomics data from global populations across relevant disease tissues is highly warranted^50^. The arising insights can spur clinical translation pathways to achieve an improvement in quality of life for the hundreds of millions of individuals affected by osteoarthritis currently left without anything but symptomatic treatment with modest effect.

## Methods

### Cohorts, phenotypes and genotypes

We conducted a GWAS meta-analysis combining up to 87 GWAS summary statistics in 11 osteoarthritis phenotypes; osteoarthritis at any site, hip osteoarthritis, knee osteoarthritis, hip and/or knee osteoarthritis, spine osteoarthritis, hand osteoarthritis, finger osteoarthritis, thumb osteoarthritis and end-stage osteoarthritis defined by total hip replacement (THR), total knee replacement (TKR) and total hip and/or knee replacement (TJR) (Supplementary Tables 1 and 2 and Supplementary Note).

To evaluate the classification accuracy of self-reported disease status, we performed a sensitivity analysis for osteoarthritis at any site excluding the 27 GWASs that contain self-reported osteoarthritis. We further expanded the analysis by performing the UKBB GWAS for osteoarthritis at any site by excluding individuals with self-reported disease status (Supplementary Figs. 5 and 6 and Supplementary Note).

To investigate the discordance between structural and symptomatic osteoarthritis, we performed a sensitivity meta-analysis restricting to cohorts with phenotypes based only on imaging for osteoarthritis at any site. The sensitivity meta-analysis includes 5 GWASs from the HKDDDPC, RIKEN and Rotterdam studies 1, 2 and 3, totalling a maximum of 6,816 cases and 9,624 controls (Supplementary Fig. 4 and Supplementary Note).

### GWAS summary statistics quality control and meta-analysis

We used a combination of in-house scripts and EasyQC^51^ (https://github.com/hmgu-itg/Genetics-of-Osteoarthritis-2.0; Supplementary Fig. 3 and Supplementary Note) to perform quality control centrally for the GWAS summary statistics in each cohort.

We used a fixed-effect inverse-variance-weighted meta-analysis approach as implemented in METAL^52^ for the 11 osteoarthritis phenotypes, by including a maximum of 87 GWAS summary statistics from 42 different cohorts, encompassing 5 major ancestry groups. We included genomic control correction unless this was already performed. After meta-analysis, we excluded any variant that was only observed in a single GWAS and/or had MAF < 0.01, which resulted in 14.7 to 24.3 million variants depending on the phenotype (Supplementary Note).

### Genome-wide significance threshold

We used *P* ≤ 1.3 × 10^−8^ to declare genome-wide significance, as previously described^3^, to account for the effective number of independent phenotypic traits. In brief, we first estimated the genetic correlation matrix between the 11 osteoarthritis traits by using bivariate LD score regression^53^ with genome-wide meta-analysis summary statistics. This method produces reasonably robust estimates of genetic correlation when the sample size of unrelated individuals is high^54^ by aiming to overcome the limitations of the analysis, including (1) the tendency to be higher than phenotypic correlations; and (2) the potential for inflated estimates when heritability estimates are low. We then calculated the effective number of independent traits (*P*_eff_) from the eigenvalues *λ*_*i*_ of the correlation matrix^55^. For the *P* = 11 osteoarthritis phenotypes in this study, *P*_eff_ = 4.6565.

$${P}_{{\rm{eff}}}=P-\mathop{\sum }\limits_{i=1}^{P}[I({\lambda }_{i} > 1)({\lambda }_{i}-1)]$$

### Defining independent signals and loci

To define independent signals, within and across phenotypes, we used a three-step approach; detailed are available at GitHub (https://github.com/hmgu-itg/Genetics-of-Osteoarthritis-2.0). (1) For each phenotype, we performed clumping using PLINK^56^ together with a significance threshold of *P* ≤ 1.3 × 10^−8^, 2 Mb window around each index variants and linkage disequilibrium (LD) threshold of 0.1. For the LD calculations, we used UK Biobank (v.3) for all ancestries (https://www.ukbiobank.ac.uk). (2) For each index variant in a given clump, we performed an approximate stepwise model-selection procedure implemented by COJO in GCTA^57^ to establish whether index variants were independent (Supplementary Note). (3) To define independent signals across phenotypes, we included index variants from all independent signals across all phenotypes if they were within 1 Mb of each other. We performed reciprocal approximate conditional analyses, implemented by COJO in GCTA^57^. We considered signals independent if either signal conditioned on the other had *P* ≤ 1.3 × 10^−8^. For each independent signal, we selected a lead variant as the variant with the most significant *P* value across all phenotypes.

To determine whether a signal was newly reported or previously known, we included all independent signals and all previously reported variants (Supplementary Table 4 and Supplementary Note) and we performed reciprocal approximate conditional analyses, implemented by COJO in GCTA^57^. We considered signals to be newly reported if either the signal or previously reported variant conditioned on the other had *P* ≤ 1.3 × 10^−8^. After COJO analysis, we also required that each genome-wide significant independent signal should be internally validated in at least one osteoarthritis phenotype. Internal validation was defined as at least two GWASs having the same direction of risk effect and nominally significant (*P* < 0.05). We defined a locus as follows: (1) index variants separated by <1 Mb were grouped together in the same locus; (2) we added 500 kb upstream and downstream of index variants to define the final region of each locus. The loci that contained more than one index variants have been extended out to 500 kb beyond edge variants. If a locus contained a variant that was previously reported for osteoarthritis, the locus was considered to be known.

### Genetic architecture

#### Phenotypic variance explained

We estimated the phenotypic variance explained by the 962 independently associated variants as a function of the effect size and the risk-allele frequency (Fig. 1 and Supplementary Fig. 1). The phenotypic variance explained by a variant is ln(OR)^2^ × 2 × RAF × (1 − RAF), where ln(OR) is the natural logarithm of the OR of the variant in the meta-analysis, and RAF is its weighted risk-allele frequency across all cohorts.

#### Chromosome X meta-analysis

For the chromosome X non-pseudoautosomal region, we performed the GWAS in men and women separately. Moreover, for those cohorts without their own reference panel that imputed to the Haplotype Reference Consortium (HRC), we applied an additional level of quality control to ensure only good-quality genotypes were included (Supplementary Note).

#### Sex-differentiated meta-analysis

We carried out a sex-differentiated analysis to identify any sex-specific variants in addition to the variants identified in the sex-combined meta-analysis, potentially missed due to differences in effects between male and female individuals (magnitude and/or direction). We used GWAMA^58,59^ (https://genomics.ut.ee/en/tools), which provides four different *P* values: single-sex, combined, heterogeneity (*P*_het_), and differentiated (*P*_diff_). In the sex-differentiated analysis, male and female individuals are analysed separately in each GWAS. The male- and female-specific allelic effect estimates are obtained by a fixed-effects meta-analysis, and tested for association with the trait, allowing for sex-differentiation using ${X}_{Dj}^{2}$. By contrast, in the sex-combined analysis, male and female individuals are analysed combined in each GWAS, ambivalent to sex. Combined allelic effect estimates are obtained from a fixed-effects meta-analysis, weighted by the inverse variance, and tested for association with the trait. We defined a significant sex-differentiated association on the basis of the following criteria, all of which must be satisfied: a significant association with one osteoarthritis phenotype in at least one single sex (*P* ≤ 1.3 × 10^−8^) and a significant sex-differentiated *P* value (*P*_diff_ ≤ 1.3 × 10^−8^) and a significant heterogeneity *P* value (*P*_het_ ≤ 0.0125). If the direction of effect between male and female individuals is opposite, we additionally required the association to be present in one sex and at least nominally significant in the opposite direction in the other sex, to ensure that the observed difference in effect is not due to chance or power differences. We defined the independent signals using the three-step approach in COJO and required that they be internally validated (as defined above). The *P*_het_ significance was determined according to the number of newly identified sex-specific variants (*n* = 4), which are independent of the previously reported variants and the main analysis variants (Supplementary Table 5). To identify potential effector genes for the sex-specific signals, we performed fine-mapping and produced 95% credible sets for all 4 signals; each set contained the lead variant (Supplementary Table 6 and Supplementary Note).

#### Non-European-ancestry-specific signals

We performed a fixed-effect inverse-variance-weighted meta-analysis using METAL in five ancestry groups separately (European, African, Hispanic, East Asian and South Asian), and for sensitivity analysis, we also performed meta-analysis of these data using Han and Eskin’s random-effects model (RE2)^60^ implemented in METASOFT (http://genetics.cs.ucla.edu/meta_jemdoc/). None of the variants in the non-European-ancestry-specific meta-analysis reached study-wide significance (*P* ≤ 1.3 × 10^−8^).

#### Genetic risk score analyses

We derived GRSs for osteoarthritis of the knee, hip, hip and/or knee, hand, finger, thumb, THR, TKR, and TJR and performed validation in the Million Veteran Program (MVP) (Supplementary Note and Supplementary Table 7). The MVP did not contribute to the joint-specific meta-analysis and is therefore an independent validation set for the GRS.

#### Signal enrichment in cell types associated with skeletal development

Functional GWAS analysis^61^ was applied to identify disease-relevant cell types as described in detail previously^62^ (https://github.com/natsuhiko/PHM). In brief, the association statistics (log[OR] and standard errors) were converted into approximate Bayes factors using the Wakefield approach^63^. After defining a *cis*-regulatory region of 1 Mb centred at the transcription start site (TSS) for each gene, the Bayes factors of variants existing in each *cis* region were weighted and averaged by a prior probability (an exponential function of TSS proximity), which was estimated from the distance distribution of regulatory interactions^64^. Finally, the likelihood of an fGWAS model was given by the averaged Bayes factors across all genes multiplied by the feature-level prior probability. The latter was obtained from a linear combination of cell-type-specific expression and the averaged expression across all cell types as a baseline. The maximum-likelihood estimator of the effect size for the cell-type-specific expression was used to compute the enrichment of each cell type.

Full summary statistics from the GWAS were used to test knee osteoarthritis and TKR GWAS signals against single-cell knee tissue data, hip osteoarthritis and THR against hip tissue data, and finger osteoarthritis against data from all appendicular tissues. For results presentation, the 30 cell types from single-cell multiome data were grouped into three different categories: those involved in chondrogenesis (9 cell types), osteogenesis (4 cell types) and all other cell lineages^4^ (17 cell types) (Fig. 2 and Supplementary Table 8).

#### Fine-mapping

For each independent signal and each phenotype, we included all variants within 1 Mb around the lead variant. GWAS summary statistics quality control was performed using kriging_rss from susieR package^65^ (v.0.12.27, R v.4.2.1^66^); we used this function to calculate, based on the observed *Z* scores, the expected *Z* score and its variance; we then detected possible outliers using standardized differences between the observed *Z* score and the expected value, at the significance level 0.05, corrected for multiple testing using the Bonferroni method. Fine-mapping of the GWAS summary statistics was performed using susie_rss function from the susieR package^65^ (v.0.12.27, R v.4.2.1^66^). For the fine-mapping, we set the maximum number of causal variants to 10 and a purity threshold of 0.1 to determine 95% credible sets of potentially causal variants. External LD matrices were computed using PLINK (v.1.9) on the imputed genotypes from UK Biobank data (v.3) of all ancestries. Out of a total of 962 independent variants, 913 were assigned a credible set, of which 855 contained the lead variant (Supplementary Table 9).

### Biological insights

#### Identification of effector genes and variants

The main challenge here and in any GWAS is to pinpoint the likely causal variants and the biological effects and mechanisms through which they have a role in disease. To this end, we integrated multiple orthogonal statistical and functional methods to identify effector genes. We considered 24 supporting lines of information, including variant information, functional genomics and database searches (Extended Data Fig. 1 and Supplementary Note). To assess whether certain lines of evidence are more informative than others, we conducted sensitivity analyses at both the variant and gene levels, along with heritability analyses (Supplementary Tables 13, 21 and 22, Supplementary Fig. 8, Extended Data Fig. 2 and Supplementary Note). For the additional four sex-specific signals, we considered variant consequence, fine-mapping within a gene transcript, active promoter, human and mouse musculoskeletal and pain/neuronal phenotype searches as the rest of the supporting lines were performed with males and females combined. We consider newly reported effector genes to be those that were not identified previously^3^. We use the term identify in reference to effector genes to indicate that these genes are implicated as having a role in osteoarthritis.

#### Pathway analysis

We carried out pathway over-representation analysis with the 700 effector genes. We performed pathway analyses using different thresholds as inclusion criteria for genes from scores of 3 and above, up to scores of 7 and upwards (Supplementary Table 26, Extended Data Fig. 3 and Supplementary Note).

#### Subchondral bone allelic imbalance

Allelic expression imbalance was determined using RNA-sequencing data of macroscopically preserved subchondral bone of 24 patients who underwent total joint replacement surgery due to osteoarthritis (RAAK-study, granted by the medical ethics committee of Leiden University Medical Center, P08.239/P19.013) (Supplementary Note, Supplementary Table 27 and Supplementary Fig. 10).

#### Colocalization with plasma pQTL

We performed colocalization of the osteoarthritis associations with associations with variations in protein levels in plasma (plasma pQTL) using the coloc software package implemented in R^67^. For plasma pQTL analysis, we used the dataset described previously^68^, which tested for the association of 58 million sequence variants with levels of 2,941 proteins, measured by Olink Explore 3072, in plasma samples from 46,218 individuals of British or Irish ancestry included in the UK Biobank dataset. Using summary statistics for the osteoarthritis phenotypes (excluding the UK Biobank datasets) and the plasma pQTL, that is, effects and *P* values, we calculated Bayes factors for each of the variants in the associated regions tor the two traits and used coloc to calculate posterior probability for two hypotheses: (1) that the association with osteoarthritis phenotypes and plasma pQTL are independent signals (PP3); and (2) that the association with osteoarthritis phenotypes and plasma pQTL are due to a shared signal (PP4) (Supplementary Table 28 and Supplementary Note).

#### LOF burden analysis

We used the variant effect predictor (VEP)^69^ to predict the consequences of the variants sequenced in each dataset. We classified as high-impact variants those predicted as start-lost, stop-gain, stop-lost, splice donor, splice acceptor or frameshift, collectively called LOF variants. We filtered out LOF variants predicted by the Loss-Of-Function Transcript Effect Estimator^70^ (LOFTEE; https://github.com/konradjk/loftee) not to be likely to be truly LOF (for example, near the end of the transcript) and used only high-confidence LOF variants.

We classified as moderate-impact variants (MIS) those missense variants predicted with LOF by at least one of the following prediction methods: MetaSVM, MetaLR^71^ or CADD^72^ (combined annotation dependent depletion) with a phred score of ≥25, using variants available in dbNSFP (v.4.1c)^73^. We further included indels of moderate impact without any filtering.

We used logistic regression under an additive model to test for association between (1) LOF or (2) LOF + MIS gene burdens and phenotypes, in which disease status was the dependent variable and genotype counts as the independent variable, using a likelihood ratio test to compute two-sided *P* values. Individuals were coded 1 if they carried any of the LOF variants (LOF/LOF + MIS) with MAF < 2% and 0 otherwise. For the analyses, we used software developed at deCODE Genetics^74^. We analysed these gene burden models in whole-genome sequencing (WGS) data and then imputed data for 211,690 patients with osteoarthritis (osteoarthritis at any site), of which 54,513 had WGS, and 719,856 controls, of which 148,488 had WGS, in the UK Biobank, Icelandic, Danish and US Intermountain datasets^75^, and the FinnGen dataset for the LOF model, and meta-analysed the results. For Iceland, we included county of birth, age, age squared, sex and an indicator function for the overlap of the lifetime of the individual with the time span of phenotype collection as covariates to account for differences between cases and controls. We used county of birth as a proxy covariate for the first principal components (PCs) in our analysis because county of birth has been shown to be in concordance with the first PC in Iceland^76^. The UK, Danish and US associations were adjusted for sex, age and the first 20, 12 and 4 PCs, respectively. We used LD score regression intercepts^53^ to adjust the *χ*^2^ statistics and avoid inflation due to cryptic relatedness and stratification, using a set of 1.1 million variants. *P* values were calculated from the adjusted *χ*^2^ results.

Meta-analysis was performed on the summary results from Iceland, the UK, Denmark and the USA, when available, using a fixed-effects inverse-variance-weighted method^77^, in which the datasets were allowed to have different population frequencies for alleles and genotypes but were assumed to have a common OR and weighted with the inverse of the variance of the effect estimate derived from the logistic regression. The FinnGen dataset was also included in the LOF model for the *VIT* gene, no LOF variants were identified in the other genes. We set a study-wise significance threshold at *P* < 7.1 × 10^−5^, accounting for the 700 unique genes tested, whereas a genome-wide significance threshold is considered for burden *P* < 2.5 × 10^−6^, accounting for the approximately 20,000 genes in the genome.

#### Transcription factor enrichment

To determine whether any of the variants in the credible set were localized in gene regulatory regions, we used the ROADMAP ChromHMM data^78^, predicting gene regulatory regions (enhancers and promoters) in human mesenchymal stem-cell-derived chondrocytes (E049) and primary osteoblasts (E127). We used the ROADMAP-generated core 15-state chromatin state model, where the following states were considered as gene regulatory: active TSS, flanking active TSS, enhancers, genic enhancers, bivalent/poised TSS, flanking bivalent/poised TSS/enhancer and bivalent enhancer. Variants that localized in one of these gene regulatory regions were also assessed if they affected a possible transcription-factor-binding motif as predicted by Haploreg (v.4.2)^79,80^ (Supplementary Note and Supplementary Tables 11, 12 and 14).

#### Drug repurposing opportunities

To identify potential drug-repurposing options from the effector gene list, we queried around 17,000 drug molecules and 21,087 protein targets (with UniProt and Ensembl identifiers) from Open Targets^81^ (https://platform.opentargets.org/downloads). This dataset comprises 1,543 genes, of which the protein products are the target of at least 1 drug, and 4,930 drugs that target at least 1 gene product. For the 700 effector genes, there were 652 approved drugs that target the protein of 70 unique genes. After filtering out drugs that were withdrawn and that were not listed with an indication, there are 473 drugs that target the protein of 69 unique effector genes (Supplementary Table 30). Finally, we also investigated the similarities and differences between these effector genes and those in large pain datasets (Supplementary Table 31 and Supplementary Note).

#### Biological insights

With the increase in sample size, we detected 39 loci with >1 independent signal (13.5% of the loci have ≥1 additional signal) (Supplementary Table 3). The additional signals may well exert their effects through the same or different effector gene as many loci have ≥1 effector genes we consider all effector genes as having a potential role in osteoarthritis pathology. With the effector genes as a foundation, our objective was to establish connections among the genes by using multiple sources to identify pathways, networks and common themes that link the effector genes, that could be used for drug targeting. We ranked the 700 effector genes according to their score. We performed literature searches to glean information regarding functionality and associations between the effector genes (Supplementary Note). Finally, we conducted genetic heritability analysis for each of the eight biological processes identified with LDAK v.6 software^82^ (https://www.ldak.org) by using summary statistics from the main meta-analysis of the 11 osteoarthritis phenotypes (Supplementary Fig. 9).

#### Ethics statement

Study-level ethics statements are provided in the Supplementary Note.

### Reporting summary

Further information on research design is available in the Nature Portfolio Reporting Summary linked to this article.

## Online content

Any methods, additional references, Nature Portfolio reporting summaries, source data, extended data, supplementary information, acknowledgements, peer review information; details of author contributions and competing interests; and statements of data and code availability are available at 10.1038/s41586-025-08771-z.

## Supplementary information

Supplementary Information Supplementary Notes, Supplementary Methods, Cohort Descriptions, Consortia Information, Acknowledgements and Funding, Ethics and Study approval and Supplementary Figs. 1–12.

Reporting Summary

Supplementary Tables Supplementary Tables 1–33.

## Data Availability

The data from the genome-wide summary statistics for each meta-analysis generated in this study are publicly available at the downloads page of the Musculoskeletal Knowledge Portal (https://msk.hugeamp.org/downloads.html). Individual-level data can be requested directly from contributing studies, listed in Supplementary Table 2.

## Code availability

Analyses were conducted using publicly available software: BCFtools v.1.13 (https://samtools.github.io/bcftools/bcftools.html), CrossMap v.0.5.4 (https://crossmap.readthedocs.io/en/latest/), EasyQC v.23.8, 5 June 2020 (https://www.uni-regensburg.de/medizin/epidemiologie-praeventivmedizin/genetische-epidemiologie/softwssare), GWAMA v.2.2.2 (https://genomics.ut.ee/en/tools), METAL version released on 25 March 2011 (https://genome.sph.umich.edu/wiki/METAL_Documentation), METASOFT v.2.0.0 (15 February 2012; http://genetics.cs.ucla.edu/meta_jemdoc/), PLINK v.1.9 (https://www.cog-genomics.org/plink/1.9/), PLINK v.2.09 (https://www.cog-genomics.org/plink/2.0/), R (https://www.R-project.org/), COJO in GCTA v.1.93.0beta (https://yanglab.westlake.edu.cn/software/gcta/#COJO), Functional GWAS analysis (https://github.com/natsuhiko/PHM), BGEN bgenix v.1.1.7, revision (https://www.biorxiv.org/content/10.1101/308296v2), HiCLift (https://github.com/XiaoTaoWang/HiCLift), GRNBoost2 algorithm in Scenic+ software (https://github.com/aertslab/scenicplus), dbNFSFP v.4.1c (https://www.dbnsfp.org/home), CADD (https://cadd.gs.washington.edu/), LDAK v.6 (https://www.ldak.org). Analysis also included the following R packages: coloc v.5.2.2 (https://rdrr.io/cran/coloc/man/coloc.abf.html), ClusterProfiler64 v.4.8.2 (https://bioconductor.org/packages/release/bioc/html/clusterProfiler.html), susieR package62 v.0.12.27, R v.4.2.163 (https://cran.r-project.org/web/packages/susieR/index.html). A collection of scripts that were used in this study are available at GitHub (https://github.com/hmgu-itg/Genetics-of-Osteoarthritis-2.0). Online resources, databases, datasets used were as follows: Ensembl BioMart (http://grch37.ensembl.org/biomart/martview/, release 110, GRCh37), UCSC liftOver tool (https://genome.ucsc.edu/cgi-bin/hgLiftOver), ROADMAP ChromHMM (https://egg2.wustl.edu/roadmap/web_portal/index.html, https://egg2.wustl.edu/roadmap/web_portal/chr_state_learning.html), Ensembl REST API (http://grch37.rest.ensembl.org), University of California Santa Cruz (UCSC) Genome Browser (https://genome.ucsc.edu), Ensembl (http://grch37.ensembl.org/Tools/VEP), HaploReg (https://pubs.broadinstitute.org/mammals/haploreg/haploreg.php, v.4.2), Mouse Genome Informatics database (www.informatics.jax.org), International Mouse Phenotyping Consortium (http://www.mousephenotype.org/), Online Mendelian Inheritance in Man (OMIM) database (https://www.omim.org/), Human Pain Genetics Database (https://humanpaingeneticsdb.ca/hpgdb/), Enrichr (https://maayanlab.cloud/Enrichr/), Gene Ontology (https://geneontology.org/), Reactome (https://reactome.org/), Wikipathways (https://www.wikipathways.org/), Open Targets (https://platform.opentargets.org/downloads, v.23.09) and BioRender (https://www.biorender.com/).
