# Supplementary material for: Translational genomics of osteoarthritis in 1,962,069 individuals
Source: Nature. 2025 Apr 9;641(8065):1217–24. doi: 10.1038/s41586-025-08771-z (PMC12119359; doi:10.1038/s41586-025-08771-z)
Supplement: Supplementary file 1 — Supplementary Notes, Supplementary Methods, Cohort Descriptions, Consortia Information, Acknowledgements and Funding, Ethics and Study approval and Supplementary Figs. 1–12. [file 41586_2025_8771_MOESM1_ESM.pdf]

nature portfolio

https://doi.org/10.1038/s41586-025-08771-z

Supplementary information

Translational genomics of osteoarthritis in 1,962,069 individuals

In the format provided by the authors and unedited

Genome-wide analysis in 1,962,069 individuals identifies 962 risk variants and translational opportunities for osteoarthritis

Supplementary Note

Supplementary Text **7**

Genetic architecture of osteoarthritis 7

Replication of previously-reported osteoarthritis signals 7

Accuracy of self-reported data 7

Symptomatic versus structural only osteoarthritis 8

Causal variant fine-mapping 8

Transcription factor enrichment 8

Lines of evidence assessment 9

Biological Insights 9

Pathway analysis 9

Subchondral bone allelic imbalance 9

Colocalization with plasma pQTLs 10

Retinoic acid signalling 10

Transforming growth factor signalling 11

Bone morphogenetic protein signalling 14

Wnt signalling 14

Fibroblast growth factor signalling 15

Extracellular matrix 16

Circadian rhythm 17

Glial cell-related 20

Effector genes traversing biological processes 22

Quantitative comparisons between the biological processes 22

Drug Targets 22

Similarities and differences between the effector genes identified in this study, and those in large pain datasets. 23

Supplementary Methods **23**

Cohorts, phenotypes and genotypes 23

GWAS summary statistics quality control and meta-analysis 24

Defining independent signals and loci 24

Chromosome X meta-analysis 24

Sex-differentiated meta-analysis 25

Genetic risk score analyses 25

Effector gene identification by combining 24 lines of evidence 25

Determination of genes in osteoarthritis-associated loci 25

Variant annotation and fine-mapping 25

Active promoter 26
Chondrocyte Hi-C loop overlap 26
Genes involved in gene regulatory networks important in fetal development 27
Multi-omics in primary osteoarthritis cartilage 27
Colocalization with molecular QTLs in osteoarthritis relevant tissues 27
Human and mouse musculoskeletal and pain/neuronal phenotypes 28
Lines of evidence assessment 28
Biological insights additional analysis 29
Pathway analysis 29
Subchondral bone allelic imbalance 29
Carriage of risk alleles by pathway 29
Transcription factor enrichment 30
Biological Insights 30
Cohort Descriptions 31
arcOGEN 31
ARGO-Athens 31
ARGO-Larissa 31
BioMe BioBank Program 31
Bunkyo Health Study 32
China Kadoorie Biobank 32
deCODE 32
CHB+DBDS 32
eMERGEIII 32
Estonian Biobank 33
FinnGen 33
GeisingerMyCode_F60k and GeisingerMyCode_New30k 33
Generation Scotland 34
Genes & Health 34
Health and Retirement Study 34
Hong Kong Degenerative Disc Disease Population Cohort (HKDDDPC) 34
HUNT study 35
INTERMOUNTAIN 36
JoCoOA 36
LifeLines 36
Mass General Brigham Biobank 36
Million Veteran Program 36
NHS: Nurses Health Study and Nurses Health Study II 37
NIHRBioResource 37
Norwegian Arthroplasty Register (NAR) 37

The Osteoarthritis Initiative (OAI) 37
QIMR [Over 50's (Aged) and Osteoarthritis (OA) Studies] 38
QIMR - PISA [Prospective Imaging Study of Ageing: Genes, Brain and Behaviour] 38
QIMR - GBP [Australian Genetics of Bipolar Disorder Study] 39
QIMR - AGDS [Australian Genetics of Depression Study] 39
RAAK study 39
RIKEN Study 39
The Rotterdam Study 39
Shimane CoHRE Study 39
SHIP START and SHIP TREND 39
SIMPLER-SIMPLER, SIMPLER-COSMC and SIMPLER-SMCC 40
Tohoku Medical Megabank Organisation 40
TwinsUK 40
UK Biobank 40
UK Household Longitudinal Study (UKHLS) 41
Consortia Information 41
The arcOGEN Consortium 41
The ARGO Consortium 41
Regeneron Genetics Center 41
Estonian Biobank Research Team 42
DBDS Genomic Consortium 42
Genes & Health Research Team 42
HUNT All-In Pain 43
FinnGen 43
Million Veteran Program 44
Acknowledgements and Funding 48
arcOGEN 48
BioMe BioBank Program 48
Bunkyo Health Study 48
China Kadoorie Biobank 48
deCODE 49
CHB+DBDS 49
eMERGEIII 49
Estonian Biobank 49
FinnGen 49
GeisingerMyCode_F60k and GeisingerMyCode_New30k 50
Generation Scotland 50
Genes & Health 50
Health and Retirement Study 51

Hong Kong Degenerative Disc Disease Population Cohort (HKDDDPC)....51
HUNT study....51
INTERMOUNTAIN....51
J. Patrick Pett....51
JoCoOA....51
Joyce van Meurs and Cindy G. Boer....51
LifeLines....52
Mass General Brigham Biobank....52
Million Veteran Program....52
NHS: Nurses Health Study and Nurses Health Study II....52
NIHRBioResource....52
Norwegian Arthroplasty Register (NAR)....52
The Osteoarthritis Initiative (OAI)....53
QIMR [Over 50's (Aged) and Osteoarthritis (OA) Studies]....53
QIMR - PISA [Prospective Imaging Study of Ageing: Genes, Brain and Behaviour]....53
QIMR - GBP [Australian Genetics of Bipolar Disorder Study]....53
QIMR - AGDS [Australian Genetics of Depression Study]....53
RAAK study....53
RIKEN Study....53
The Rotterdam Study....54
Shimane CoHRE Study....54
SHIP START and SHIP TREND....54
SIMPLER-SIMPLER, SIMPLER-COSMC and SIMPLER-SMCC....54
Tohoku Medical Megabank Organisation....54
TwinsUK....54
UK Biobank....55
UK Household Longitudinal Study (UKHLS)....55
Ethics and study approval....55
arcOGEN....55
ARGO-Athens....55
ARGO-Larissa....55
BioMe BioBank Program....55
Bunkyo Health Study....55
China Kadoorie Biobank....55
deCODE....55
CHB+DBDS....56
eMERGEIII....56
Estonian Biobank....56
FinnGen....56

GeisingerMyCode_F60k and GeisingerMyCode_New30k 57
Generation Scotland 57
Genes and Health 57
Health and Retirement Study 57
Hong Kong Degenerative Disc Disease Population Cohort (HKDDDPC) 57
HUNT study 57
INTERMOUNTAIN 57
JoCoOA 57
LifeLines 57
Mass General Brigham Biobank 57
Million Veteran Program 57
NHS: Nurses Health Study and Nurses Health Study II 58
NIHRBioResource 58
Norwegian Arthroplasty Register (NAR) 58
The Osteoarthritis Initiative (OAI) 58
QIMR [Over 50's (Aged) and Osteoarthritis (OA) Studies] 58
QIMR - PISA [Prospective Imaging Study of Ageing: Genes, Brain and Behaviour] 58
QIMR - GBP [Australian Genetics of Bipolar Disorder Study] 58
QIMR - AGDS [Australian Genetics of Depression Study] 58
RAAK study 58
RIKEN Study 58
The Rotterdam Study 59
Shimane CoHRE Study 59
SHIP START and SHIP TREND 59
SIMPLER-SIMPLER, SIMPLER-COSMC and SIMPLER-SMCC 59
Tohoku Medical Megabank Organisation 59
TwinsUK 59
UK Biobank 59
UK Household Longitudinal Study (UKHLS) 59
Supplementary Figures 60
Supplementary Figure 1. Joint-specific genetic architecture of osteoarthritis. 61
Supplementary Figure 2. Manhattan and quantile-quantile plots for the main analysis, non-European ancestry and sex-stratified meta-analysis. 63
Supplementary Figure 3. Example of the quality control plots for GWAS datafiles prior to EasyQC 77
Supplementary Figure 4. Sensitivity analysis for osteoarthritis at any site for imaging-only datasets (n=6,816 cases and 9,624 controls). Beta and -$\log_{10}(P)$ comparisons for osteoarthritis at any site between the imaging-only meta-analysis and main meta-analysis. 79

Supplementary Figure 5. Sensitivity analysis for osteoarthritis at any site for datasets without any self-reported definitions included (n=309,125 cases and 793,538 controls). Manhattan and quantile-quantile plots for osteoarthritis at any site for the meta-analysis without self-reported osteoarthritis. Beta and -log10(P) comparisons for osteoarthritis at any site between the meta-analysis without self-reported osteoarthritis and the main meta-analysis. 81

Supplementary Figure 6. Osteoarthritis at any site GWAS in UK Biobank (UKBB): Sensitivity analysis comparing the main UKBB GWAS analysis to UKBB without self-reported definition included. 82

Supplementary Figure 7. Significant enrichment altered Transcription Factor binding Motifs. 83

Supplementary Figure 8. Bar plot illustrating the sensitivity of the 24 lines evidence for known osteoarthritis genes pre and post Boer, Hatzikotoulas, Southam *et al*. 2021. 84

Supplementary Figure 9. Genetic heritability for each of the 8 biological processes. 85

Supplementary Figure 10. Allelic imbalance in subchondral bone....86

Supplementary Figure 11. Additional pathways related to FGF signalling that have relevance to osteoarthritis....87

Supplementary Figure 12. Additional Wikipathways (WP5205) for genes involved in clock-controlled autophagy. 88

References 89

Supplementary Text

Genetic architecture of osteoarthritis

Replication of previously-reported osteoarthritis signals

We determined if previously reported signals are replicated in this meta-analysis, at the nominal significance level (*P* value <0.05) and in the same direction of effect. We find that among the 435 previously-reported osteoarthritis-associated variants (some of which overlap across phenotypes), 420 show association at nominal significance (*P* value < 0.05), and 249 reach study-wide significance (*P* value $\leq 1.3 \times 10^{-8}$) at the exact same variant, in at least one osteoarthritis phenotype with the same direction of effect as previously reported (Supplementary Table 4). Fifteen variants were not replicated in this study: 4 did not meet our quality control criteria and didn't proceed to meta-analysis; 1 was first identified in African American ancestry individuals and is nominally significant here in the African only hip and/or knee osteoarthritis dataset, but with a different direction of effect; 3 are rare variants with loss of power mainly due to variants not present in the updated deCODE cohort; 4 variants had been reported for self-reported osteoarthritis and early-onset osteoarthritis, which have not been investigated here; 2 variants had been reported in build 36 of the human genome, which might indicate a mapping issue; and 1 deletion variant (5:67817769). The 4 previously-reported female-specific variants replicated in the female analyses but not in the main analysis, and one variant replicated in the European ancestry analysis only for osteoarthritis at any site, rather than in the main analysis here.

Accuracy of self-reported data

To evaluate the classification accuracy of self-reported disease status, we performed a sensitivity analysis for osteoarthritis at any site by excluding the 27 GWAS (30% of the total number of datasets) that contain self-reported osteoarthritis (Methods). The remaining 60 datasets contained up to a maximum of 309,125 cases and 793,538 controls. We compared the results of this meta-analysis with the main analysis. In summary, there were 1977 variants in the without self-reported osteoarthritis at any site meta-analysis that reach genome-wide significance (*P* value < 5×10^{-8}) and 100% had the same direction of effect as the main osteoarthritis at any site meta-analysis. 1888 (95%) of these variants are also study-wide significant in the main meta-analysis and the remaining 89 variants range from *P* value = 5.1×10^{-5} to P value = 1.32×10^{-8}. If all overlapping variants are included, we find that 17,129,725 (83%) have the same direction of effect (Supplementary Figure 5). We observed no additional signals after removal of self-reported osteoarthritis patients. In summary, these results indicate a strong correlation between the osteoarthritis at any joint site with and without the inclusion of GWAS datasets that include self-reported osteoarthritis cases. This confirms previous studies, which have reported that self-reported osteoarthritis definition is a powerful tool for genetic association studies[1-3].

To further validate these results, we have conducted a GWAS using the UKBB dataset, the largest dataset in the main meta-analysis, by excluding individuals with self-reported osteoarthritis. The number of cases was 4.14 times lower (N=19885) than the main UKBB GWAS (N=82420), where the number of controls remained the same (N=323032). In accordance with the above results, we find high correlation between the UKBB GWAS with and without the inclusion of self-reported osteoarthritis individuals (Supplementary Figure 6). In addition, the UKBB GWAS without self-reported patients, did not reveal any new signals compared to the main meta-analysis, highlighting the accuracy of phenotype definition.

Symptomatic versus structural only osteoarthritis

We have performed a sensitivity meta-analysis for osteoarthritis at any site restricting to those cohorts with phenotypes based on imaging-only (Methods). We observed no additional genome-wide significant signals from the imaging-only meta-analysis. This is not surprising given the lower power. In order to look further into the differences/similarities of effects across the main and sensitivity meta-analysis we have extracted variants based on 4 different criteria (Supplementary Figure 4): (1) Significant/suggestive in both meta-analyses: We included variants that are study-wide significant (*P* value $<1.3 \times 10^{-8}$) in the main meta-analysis and nominally significant (*P* value <0.05) in the imaging-only meta-analysis. This resulted in 1172 variants, and 1165 (99%) have concordant directions of effect. (2) Significant in main meta-analysis: We included variants that are study-wide significant in the main meta-analysis, and this resulted in 7803 variants, with 6033 (77%) having concordant directions of effect. (3) Suggestive in the imaging-only meta-analysis: We included all variants that are nominally significant (*P* value <0.05) in the imaging-only meta-analysis which resulted in 446,469 variants, and 282,488 (63%) have concordant direction of effect. (4) All overlapping variants: If we include all overlapping variants there are 8,906,420 variants, and 4,839,084 (54%) have concordant direction of effect. We also compared the -log10(*P* value) for each scenario and the Pearson correlations ranged from 0.04 (scenario 4) to 0.2 for scenario 1. In conclusion, we see concordant directions of effect between the main and imaging-only meta-analysis. We do not observe additional signals in the imaging-only meta-analysis, but this could be ascribed to relatively limited power.

Causal variant fine-mapping

Statistical fine-mapping on the regions underlying the 962 independently-associated osteoarthritis signals generated 855 95% credible sets containing potentially causal variants (Methods, Supplementary Table 9). The mean distance spanned by the variants in the credible sets was 92 kb. For the 107 regions which did not report credible sets, or the lead variant was not in the credible set, we report no credible set and include the lead variant only for the purposes of effector gene scoring (Supplementary Table 9).

Transcription factor enrichment

Variants can affect their target gene by altering gene regulation. We find that 57 of the affected transcription factor-binding motifs are significantly enriched for osteoarthritis-associated variants (P value <9.17x10-8). We also find that 15 of the enriched transcription factor motifs are known to be involved in transcription initiation and general gene regulation (Supplementary Table 12, Supplementary Figure 7). Sixteen are linked to cell development and differentiation, and 14 to neuro/endocrine development and function. There are also motifs linked with involvement of immune response and development (n=11), cellular apoptosis (n=2) and cellular stress response (n=1).

We examined if any of the proteins binding to transcription factor motifs enriched for genetic signal are produced by effector genes (Supplementary Table 12, Supplementary Figure 7). For 12 of the enriched transcription factor binding motifs, the gene expressing the relevant transcription factor is also an osteoarthritis effector gene. We find that 22 of these transcription factors are an effector gene (Supplementary Table 14). The ERalpha-1 motif and *ESR1* are of particular interest, as there is evidence of increased prevalence of osteoarthritis in women compared to men[4,5] and hormones have been suggested as a possible explanation for this disparity[6]. ESR1 is the target of 28 approved drugs for indications such as menopause, atrophy, postmenopausal osteoporosis and breast cancer (Supplementary Table 30).

Lines of evidence assessment

We find that the major lines of evidence in support of known osteoarthritis genes are mouse and human musculoskeletal and pain phenotypes, chondrocyte HiC, differential chondrocyte methylation and protein abundance, transcript contains all variants in the 95% credible set, and a 95% credible set variant has a moderate-high impact (Methods, Supplementary Table12, Supplementary Figure 8). Some lines of evidence overlap between this study and the Boer, Hatzikotoulas, Southam *et al.* study, and this may skew the results of this analysis. Notably these are human musculoskeletal phenotype, mouse neuronal phenotype, human pain disorder, human pain gene, mouse musculoskeletal and the fine-mapping to transcript and variant impact. For known osteoarthritis genes prior to Boer, Hatzikotoulas, Southam et al., a similar ranking between the lines of evidence is seen, albeit the human musculoskeletal and chondrogenesis single cell lines rank higher in terms of sensitivity in this subset of known genes. This may reflect previous approaches used for the selection of osteoarthritis candidate genes to functionally investigate, in which genes with a human musculoskeletal phenotype or developmental roles may have been prioritised for investigation. We also examined the number of effector genes supported by each line of evidence within each biological process. The same lines of evidence consistently contribute more to the identification of effector genes in each process (Extended Data Fig. 2). Since many of these major lines of evidence are specific to chondrocytes, there may be a greater likelihood of detecting pathway enrichment related to chondrocyte function. In support of these results, genetic heritability analysis, revealed that the same lines of evidence exhibit the highest variance explained estimates (Supplementary Table 22). In contrast, no particular line of evidence appeared to be more informative than others from a GWAS variant perspective (Supplementary Tables 13, 21 and 22).

Biological Insights

To understand the role played by the effector genes and the biological mechanisms at each locus, we performed: a deep dive into the literature, pathways analysis (Methods, Supplementary Table 26, Extended Data Fig. 3), allelic imbalance analysis in subchondral bone (Methods, Supplementary Table 27, Supplementary Figure 10), and colocalization with plasma pQTLs (Methods, Supplementary Table 28).

Pathway analysis

We identified significant (FDR < 0.05) enrichment of genetic association signal for biological pathways (Supplementary Table 26, Extended Data Fig. 3). The 8 highlighted pathways have related Gene Ontology pathways that show enrichment for genetic signal: Retinoic acid signalling (response to retinoic acid, $P_{FDR} = 9.17 \times 10^{-5}$), TGFB signalling (cellular response to transforming growth factor beta stimulus, $P_{FDR} = 3.50 \times 10^{-11}$), Circadian rhythm (regulation of circadian rhythm, $P_{FDR} = 2.75 \times 10^{-2}$), glial cell related pathways (glial cell differentiation, $P_{FDR} = 1.09 \times 10^{-9}$), FGF signalling (fibroblast growth factor receptor signalling pathway, $P_{FDR} = 3.456 \times 10^{-6}$), Wnt signalling (positive regulation of Wnt signalling pathway, $P_{FDR} = 6.62 \times 10^{-6}$), BMP signalling (response to BMP, $P_{FDR} = 3.20 \times 10^{-13}$), ECM assembly and organisation (regulation of extracellular matrix organization, $P_{FDR} = 7.98 \times 10^{-7}$).

Subchondral bone allelic imbalance

We calculated the allelic expression imbalance (AEI) in individuals heterozygous for each of the 262 variants (Methods) and identified 15 unique coding variants located in 11 unique genes showing significant (FDR<0.05) AEI in subchondral bone (Supplementary Table 27, Supplementary Figure 10).

Colocalization with plasma pQTLs

Ten plasma pQTLs colocalize with osteoarthritis GWAS signals. Six colocalizing plasma pQTLs are effector genes *COL2A1*, *FURIN*, *FES*, *APOE*, *ADM* and *MAX*. *COL2A1*, is the joint highest ranking effector gene in the locus (scoring 6) and a major component of the ECM. Due to cartilage degradation and remodelling during osteoarthritis, COL2A1 and associated degradation products have been investigated as potential disease biomarkers[7]. No *COL2A1* molecular QTLs (methylation, expression or protein) in primary tissues colocalized with osteoarthritis GWAS signals (Supplementary Table 13). *FES* (scoring 7) and *FURIN* (scoring 5) are located at the same, newly-identified, locus, with a single independent signal affecting osteoarthritis at any site, hip and/or knee osteoarthritis, hip osteoarthritis, THR, TKR and TJR. Osteoarthritis GWAS signals colocalize with *FURIN* methylation QTLs in cartilage (degraded and intact) and synovium, and *FES* methylation QTLs colocalized in cartilage (degraded and intact). Three of the 10 pQTLs, which colocalize with GWAS signals (Methods, Supplementary Table 28), are associated with effector genes in highlighted pathways; *FURIN* (TGFB related, extracellular matrix (ECM) related), *COL2A1* (BMP and ECM related) and *APOE* (Wnt and ECM related). *COL2A1* and *APOE* are previously reported effector genes and COL2A1 is the target of 2 approved drugs (Supplementary Table 30).

Retinoic acid signalling

The highest scoring effector gene is *ALDH1A2*, with a score of 11, which encodes RALDH2, an enzyme that irreversibly catalyses the synthesis of all trans-retinoic acid (atRA) from retinaldehyde. atRA is a derivative of vitamin A (retinol) and a hormonal signalling molecule that has an essential role in embryonic development and in the maintenance of adult tissues including cartilage and bone. Tretinoin, the drug version of atRA, is used for the treatment of acne and acute promyelocytic leukaemia[8,9]. This locus was the first genome-wide association identified for hand osteoarthritis, discovered in individuals from Iceland and the Netherlands[10]. In this study, 2 variants (rs3204689, in the 3′ UTR of the *ALDH1A2* transcript; and rs4238326, intronic in *ALDH1A2*) were also associated with allelic imbalance in intact and degraded cartilage. Both variants are present in the finger and thumb osteoarthritis 95% credible sets. Functional characterisation studies determined that osteoarthritis risk is associated with a decreased expression of *ALDH1A2* dependent upon rs12915901[11] in joint tissues (rs12915901 is present in the finger osteoarthritis credible set). Subsequent analysis found that the rs3204689 and rs4238326 risk variants were associated with low expression of *ALDH1A2* in individuals with hand osteoarthritis[12]. They also demonstrate that administration of Taralozole, a Retinoic Acid Metabolism Blocking Agent (RAMBA) decreased mechano-inflammatory genes in chondrocytes and decreased osteophyte formation in mice. Taralazole is a drug that inhibits CYP26A-C1 (Extended Data Fig. 4). CYP26A-C1 are monooxygenases that essentially eliminate atRA, and inhibition of these proteins results in increased cytosolic RA, which has an anti-inflammatory role. *CYP26B1* is the highest scoring effector gene associated with 2 independent signals for hip osteoarthritis and hand osteoarthritis. For the hand osteoarthritis signal, 5 variants make up the credible set; a missense variant, an intronic variant and 3 variants in the 3'UTR of *CYP26B1*. The missense variant, rs2241057, is internally validated in 9 European cohorts (nominally significant with the same direction of effect). According to SIFT, PolyPhen, CADD and AlphaMissense, rs2241057 is not predicted to be deleterious. A group investigating atherosclerosis and inflammation have shown that the hand osteoarthritis risk allele possessed enhanced retinoid catabolism associated with increased local inflammation[13]. The hip osteoarthritis signal at the same locus contains 24 variants in the credible set, all intergenic and residing between *CYP26B1* and *DYSF* (also a high-scoring effector gene with a score of 5). One of the hip credible set variants, rs6751566, is located in an enhancer that loops to a promoter site at the 5′ end of *CYP26B1*. The enhancer site appears to be cell-type specific, only present in

the ENCODE MSC-chondrocyte cell line according to HaploReg version 4.2[14]. RARRES2, an effector gene related to this pathway, was initially discovered in skin graft cultures where it was observed that RARRES2 was found to be expressed at lower levels in psoriatic versus non-lesional psoriatic skin graft cultures. Following topical application with Tazarotene, an increased expression of RARRES2 in the psoriatic cultures was observed[15]. The gene product of RARRES2 is chemerin, an adipokine, for which levels are positively correlated with obesity, metabolic syndrome and type 2 diabetes, and are affected by diet and time of day[16-18]. Chemerin has also been detected in synovial fluid and positively correlated with severity of knee osteoarthritis[19]. RARRES2 has decreased expression and protein abundance in high-grade compared to low-grade chondrocytes (Supplementary Table 13). This is contrary to the observation in synovium and knee osteoarthritis severity. The mechanistic link between chemerin levels and disease have not been established and tissue differences have been observed[20], which may account for the differences in observations. Chemerin is a regulator of adipogenesis, is elevated in obesity, is associated with inflammation and vascular dysfunction, and is an attractive drug target and biomarker for various metabolic conditions[21]. A further effector gene associated with the retinaldehyde pathway, *RLBP1*, is expressed in retinal pigment epithelial and functions in the visual cycle as a carrier of 11-cis-retinol and 11-cis-retinal and has a role in dark adaptation. Mutations in *RLBP1* are associated with autosomal recessive retinitis pigmentosa[22,23]. It has been shown that osteoarthritis and age-related macular degeneration are chronic comorbidities shared by older adults[24-26].

Transforming growth factor signalling

TGFB signalling plays major roles in numerous biological processes throughout embryonic and neonatal development, and adult homeostasis. *LTBP1*, with a score of 10, is the only effector gene in a locus which contains a single independent signal associated with multiple osteoarthritis phenotypes (osteoarthritis at any site, hip osteoarthritis, hip and/or knee osteoarthritis, knee osteoarthritis, TJR and TKR). *LTBP3* is also an effector gene and the highest scoring in its locus. LTBP1 and LTBP3 are structural and signalling extracellular matrix proteins that associate with fibrillin microfibrils and control the bioavailability of TGFB 1-3[27,28]. TGFB is involved in a plethora of cellular processes, including remodelling of the extracellular matrix (ECM), differentiation, proliferation, migration and apoptosis. In turn, these can have an impact on inflammation, wound healing, fibrosis and tumour progression. The TGFB pathway has an established role in osteoarthritis and 28 effector genes are linked to the TGFB pathway, including many genes that have previously been reported as effector genes: *LTBP1, LTBP3*, *TGFB1, SMAD3*, *GDF5*, *RUNX2*, *NOG, FBN2* and *TNC* (Supplementary Tables 13 and 29). Below we describe the 28 effector genes that are associated with the TGFB pathway.

One gene is involved in the processing of TGFB; *FURIN* is the highest scoring effector gene in the respective locus, with a single independent signal associated with multiple osteoarthritis phenotypes (osteoarthritis at any site, hip osteoarthritis, hip and/or knee osteoarthritis, THR, TJR). *FURIN* is a protease that is involved with the processing of TGFB1 by cleavage of the pro-peptide (LAP), in the golgi apparatus, an essential step in the activation of TGFB1[29,30]. FURIN plasma pQTLs also colocalized with an osteoarthritis locus associated with THR and osteoarthritis at any site (Supplementary Table 28); the osteoarthritis risk allele is associated with decreased plasma FURIN.

Multiple genes are involved in the regulation of the TGFB1 pathway either by interacting with receptors or signalling: *FUT8* is the highest scoring effector gene in a newly identified locus associated with knee osteoarthritis. FUT8 has decreased protein abundance in high-

grade compared to low-grade chondrocytes (Supplementary Table 13). FUT8 is involved in core fucosylation, adding fucosyl moieties to glycoproteins. FUT8 can regulate TGFB signalling pathway by core fucosylation of the TGFB receptor and it has been demonstrated in multiple tissues that inhibition of FUT8 and suppression of core fucosylation attenuates TGFB signalling[31-34]. Altered glycosylation patterns have been observed in osteoarthritis chondrocytes[35]. Chondrocyte core fucosylation by FUT8 has been linked with extracellular matrix resilience in osteoarthritis[36]. ITGB3 is a new effector gene at a known locus with 2 independent signals, one of which is newly-identified here and associated with osteoarthritis at any site. Integrins are cell surface receptors made up of an alpha and beta chain. ITGB3 is the beta chain and is involved in multiple processes depending upon cell type and alpha-beta chain combination. Integrins can modulate signalling by TGFB and, conversely, TGFB can alter the transcription of integrins and their ligands. The bi-directional interaction between integrin and TGFB is complex and important in fibrosis, cancer and wound repair. ITGB3 has been shown to accelerate the onset of senescence in human primary fibroblasts by activating the TGFB pathway, and has been identified as a potential therapeutic target during early carcinogenesis and aging[37]. *SKI* is the highest scoring effector gene in a newly-identified locus with a single independent signal associated with osteoarthritis at any site. SKI is a negative regulator of TGFB signalling by interacting with SMAD members. SMADs are signal transducers and transcriptional modulators[38]. Accelerated chondrocyte differentiation was observed in chick chondrocytes following inhibition of TGFB1 by SKI[39]. SMAD3 and newly-identified SMAD6 are both effector genes in a locus associated with 3 independent signals and multiple osteoarthritis phenotypes: a previously-reported signal associated with osteoarthritis at any site, hip osteoarthritis, hip and/or knee osteoarthritis, THR, TJR, TKR and spine osteoarthritis; and 2 new signals, one associated with hip osteoarthritis and the other associated with osteoarthritis at any site and knee osteoarthritis. SMAD6 inhibits BMP signalling of the TGFB pathway[40]. In Xenopus embryos and in mammalian cells, it was shown that Smad6 interacts with Smad1 and alters the formation of the Smad1 and Smad4 complex[41]. In mice, loss of Smad6 results in skeletal defects, with Smad6 required to limit BMP signalling during endochondral bone formation[42]. *STUB1* is associated with a single independent signal with TKR, it is among 6 effector genes identified for this locus and ranks the second highest. STUB1 is an E3 ligase involved in a multitude of biological processes by regulating the stability of proteins. It has many roles in cancer progression and immunity[43] and has been demonstrated to be a pluripotency regulator in embryonic stem cells[44]. STRUB1 has been demonstrated to negatively regulate osteoblast differentiation by interacting with Runx2[45] (*RUNX2* is another effector gene) and involved in bone remodelling[46,47]. It has been shown to interact with Smad1/4 to block BMP signalling[48] and to modulate TGFB signalling via ubiquitin-mediated degradation of Smad3[49]. *NCOR2* is one of eight effector genes associated with 2 signals at a known locus associated with osteoarthritis at any site, hip osteoarthritis, hip and/or knee osteoarthritis, knee osteoarthritis and TJR, THR and TKR. NCOR2 is recruited by SKIL to repress TGFB signalling via SMAD2 and SMAD4[50].

A number of genes affect the TGFB pathway via BMP signalling: *FST* is associated with a single independent signal with osteoarthritis at any site, hip osteoarthritis, hip and/or knee osteoarthritis, knee osteoarthritis, THR and TJR, and is a joint top effector gene for this locus along with *MOCS2*. *FST* demonstrated a decrease in expression and protein abundance in high-grade compared to low-grade chondrocytes (Supplementary Table 13). FST is a myostatin- and activin-binding protein. FST deficient mice have skeletal defects and reduced muscle mass, and die shortly after birth[51]. FST is an antagonist of some bone morphogenetic proteins[52,53] (BMP-2, BMP-4, and BMP-7). FST has been investigated as a potential therapeutic agent for injury-induced osteoarthritis and metabolic inflammation in obesity[54].

BMP2 is associated with a single independent signal for osteoarthritis at any site, hip osteoarthritis, hip and/or knee osteoarthritis, THR and TJR, and is the only effector gene in this locus. There are controversial findings regarding BMP2 levels and osteoarthritis with some findings supporting a role of BMP2 in disease and others in repair processes[55,56]. BMP2 may have a role protecting cartilage from destruction during osteoarthritis[57]. This has led to a number of tissue engineering and stem cell therapy studies with promising results investigating effective treatment methods with BMP2 for joint degeneration, such as traumatic cartilage injury and cartilage defects associated with osteoarthritis[58,59] and also in bone defects[60]. In a proof-of-concept trial in an *in vivo* murine model, the sustained release of BMP2 and BMP7 demonstrated promising results by promoting chondrogenesis and improving cartilage repair[58]. Recombinant BMP2 was approved in the US for local bone formation[61]. BMPR1B is associated with a single independent signal for knee osteoarthritis and is the highest scoring effector gene in the locus. It demonstrated differential methylation and an increased expression in high-grade compared to low-grade chondrocytes (Supplementary Table 13). BMPR1B is a receptor for ligands of the TGFB superfamily including BMP-2 and GDF5, both effector genes that have affinity for BMPR1B[62]. Expression analysis in murine knees indicate distinct roles of *Gdf5* and *Bmp2* in knee joint biology[63]. Homozygous mutations in *BMPR1B* have been linked to Hunter-Thomas type disease, which gives rise to skeletal defects in limbs[64].

Several effector genes are involved in non-canonical TGFB signalling and/or interconnected pathways. *TNF* is associated with knee osteoarthritis. TNF is a proinflammatory cytokine and a ligand for the tumour necrosis factor receptor superfamily, involved in multiple processes including immune response, haematopoiesis and morphogenesis[65]. TNF has a known role in inflammatory bone resorption and rheumatoid arthritis[66], and it has been shown that TGFB can regulate a TNF-dependent inflammatory osteoclastogenic pathway, which opens up further translational options for treatment of inflammatory osteolysis[67]. Signalling of TNF activates other pathways including NFκB1. *NFκB1* is one of 3 effector genes associated with a single independent signal for osteoarthritis at any site, hip osteoarthritis, hip and/or knee osteoarthritis, knee osteoarthritis, THR and TJR in a known locus. NFκB1 is a Rel family member that acts as a homo or heterodimer in transcriptional regulation, and is involved in many cellular processes including immune and inflammatory response[68]. The NFκB pathway has been linked to osteoarthritis pathology via cartilage catabolism, making NFκB pathway members attractive translational targets[69]. There is cross talk between the NFκB pathway to the TGFB pathway via SMAD7 and TAK1[70]. *MAPK3* is an effector gene associated with osteoarthritis at any site and knee osteoarthritis. MAPK3 demonstrated decreased protein abundance in high-grade compared to low-grade cartilage (Supplementary Table 13). MAPK3 is an extracellular signal-regulated kinase (ERK), also known as ERK1, is involved in differentiation and cell proliferation and is rapidly activated via non-canonical TGFB signalling. *MAPK9* is associated with osteoarthritis at any site, hip osteoarthritis, hip and/or knee osteoarthritis, and demonstrated decreased protein abundance in high-grade compared to low-grade cartilage (Supplementary Table 13). *MAPK9* is a c-Jun N-terminal kinase member (JNKs), also known as JNK2, and is activated by the TGFB non-canonical pathway. It is involved in multiple processes, including cell proliferation, differentiation, survival, apoptosis, and inflammation. PRKCZ is one of 4 effector genes associated with a single independent signal for osteoarthritis at any site. PRKCZ plays a role in various cellular processes, including cell polarity, survival, proliferation and neuronal function[71]. TGFB has a role in cell polarity and the regulation of epithelial-to-mesenchymal transition, in which PRKCZ forms a complex with PARD6 via SMURF1 and RHOA, which is also an effector gene associated with osteoarthritis at any site. This leads to the loss of tight junctions, thereby controlling epithelial morphology[72-74].

Multiple effector genes have links with TGFB signalling in connection with ECM composition, which has an important role in osteoarthritis pathology[75]. TGFB signalling is important in regulating the production of extracellular matrix components such as *COL1A2* and *COL3A1*[76]. *COL1A2* is the highest-scoring effector gene in a locus associated with TKR. *COL1A2* demonstrated increased expression and protein abundance and differential splicing in high-grade compared to low-grade chondrocytes (Supplementary Table 13). *COL3A1* is associated with hip osteoarthritis and THR, and showed increased gene expression and protein abundance in high-grade compared to low-grade chondrocytes, in addition to differential methylation (Supplementary Table 13). *FMOD* is joint top scoring gene in a locus associated with a single independent association with hip osteoarthritis. FMOD is a small leucine-rich proteoglycan (SLURP) and an important regulator of matrix component assembly. SLURPs have been linked to the pathology of osteoarthritis via multiple mechanisms including changes in the ECM and modulation of TGFB members[77]. FMOD can bind and potentially inhibit TGFB activity by sequestering it in the ECM[78].

Bone morphogenetic protein signalling

BMP signalling exerts diverse effects on joint tissues and contributes to various aspects of osteoarthritis pathology. Here, we focus on the effector genes that are BMP family members, receptors and BMP antagonists. There are 4 effector genes that are BMP subfamily members, 3 of which are newly reported (*BMP2*, *BMP6*, *GDF6*, plus previously reported *GDF5*). A number of studies have investigated BMP family members from a regenerative standpoint: BMP2 and BMP6 have osteogenic activities[79] and have been identified as having important roles in cartilage and bone regeneration and repair[80-82]. BMP2 may have a role protecting cartilage from destruction during osteoarthritis[57]. In a trial using an autologous bone graft substitute with recombinant BMP6 in patients undergoing surgery to delay medial osteoarthritis, accelerated bone healing was observed compared to the placebo[83]. GDF5 is also involved in cartilage homeostasis. In chondrocytes, GDF5 predominantly signals via BMPR1B, another effector gene[84]. GDF5 and was shown to also act as an inhibitor of BMP2 signalling by an unknown mechanism, possibly by a new co-receptor[85], and is associated with joint repair; it is highly expressed after injury and up-regulated during the early stages of osteoarthritis[86]. GDF6 has been investigated in regenerative medicine, in which differentiating mesenchymal stem cells were derived from human embryonic stem cells for cell-mediated bone regeneration[87], and has shown promising effects in intervertebral disc regeneration therapies[88]. Five effector genes are key BMP antagonists[89]: *CHRD* is an antagonist of BMP2/4/7[89] and *CHRDL2* which was shown to promote osteosarcoma proliferation and metastasis via BMP9[90]. NOG also inhibits BMP2 and TGFB1 expression, and prevented cartilage destruction[91,92]. SOST (described in main text and below) and FST (described above). Three effector genes encode receptors (*BMPR1B*, *SORL1* and *KDR*). KDR is described below. In addition, *BMPR1B* was shown to act as a cartilage stabilizer during joint morphogenesis by preventing chondrocyte hypertrophy[93]. *SORL1* (also known as *LR11*) is an intracellular receptor that sorts cargo proteins, including kinases, phosphatases, and signalling receptors, to their destined subcellular location[94]. In mice, Lr11 was shown to negatively regulate thermogenesis by binding to BMPRs and inhibiting BMP/TGFB signalling to suppress activation of thermogenic genes in adipocytes[95]. The remaining BMP-related effector genes are involved in modulating or response of BMP signalling.

Wnt signalling

The Wingless-related integration site family members are encoded by 19 genes, 2 of which are effector genes (*WNT3* and *WNT5a*) (Supplementary Table 29). WNT3 is essential for

primary axis formation during vertebrate embryogenesis in the mouse[96]. In humans, homozygous nonsense mutations in WNT3 cause tetra-amelia, in which all 4 limbs are missing, indicating that WNT3 is necessary during the initial phases of human limb formation[97]. Wnt signalling can induce a wide variety of intracellular pathways and has both catabolic and anabolic effects on cartilage and bone, depending on the context and signalling pathway involved[98-100]. WNT5a has been shown to promote early chondrocyte differentiation and to repress chondrocyte hypertrophy[101], and has an important role in regulating longitudinal growth of the developing long bones[102]. The expression of WNT5a has been shown to be positively correlated with osteoarthritis[103] and also demonstrates increased expression in degraded compared to intact cartilage[104] (Supplementary Table 13). Most effector genes are involved with the modulation of the Wnt signalling pathway and for those effector genes with GO terms related to Wnt signalling half are associated with negative regulation and half with positive regulation of the Wnt signalling pathway (according to The Gene Ontology (GO) knowledgebase[105,106]; http://geneontology.org). Moderate Wnt activity is essential for chondrocyte proliferation and maintenance, whilst excessive activation or suppression of the Wnt-β-catenin pathway results in cartilage degradation. *WLS*, a transmembrane protein located in the endoplasmic reticulum, is essential for the intracellular transport and secretion of Wnt proteins for the initiation of Wnt signalling[107,108]. *WLS* has been shown to be differentially methylated between degraded and intact articular cartilage samples[109,110]. DOT1L methylates lysine 79 on histone 3 (H3K79), resulting in a histone modification and in gene expression changes. DOT1L was shown to be protective against osteoarthritis by preventing Wnt hyper-activation through negative modulation of SIRT1 Wnt signalling[111]. Variants in the *DOT1L* gene have been associated with cartilage thickness[112] (joint space width). SFRP4, located at a newly-discovered locus, mostly functions as a Wnt antagonist[113]. In bone, SFRP4 affects osteoblast and osteoclast functioning during development and remodelling, with a loss of SFRP4 preventing age-related bone loss in the trabecular bone[114]. Mutations in SFRP4 are associated with Pyle disease, which is characterised by increased trabecular bone, cortical bone thinning and increased fracture risk due to fragility[115]. Five of the effector genes in this pathway express proteins that are the targets of approved drugs (*TGFB1*, SOS*T*, *PSMB8*, *PSMC3* and *COL6A1*). SOST is primarily expressed by osteocytes, although it is also expressed in chondrocytes. Mutations in *SOST* are associated with sclerosteosis and Van Buchem disease, in which increased bone formation causes bone thickening[116]. Inhibition of SOST inhibits bone resorption and promotes bone formation[117]. Two more effector genes may be associated with an antiosteoporotic therapeutic: WNT5a and *NFATC1*. *NFATC1* has a role in chondrogenesis and cartilage growth[118], and has been shown to regulate bone mass in osteoblasts and osteoclasts[119,120]. Strontium ranelate (SrRan) is an anti-osteoporotic treatment that increases bone formation and decreases bone resorption[121,122]. SrRan activates NFATC1 signalling in osteoblasts, and the canonical and non-canonical Wnt signalling pathways are involved in the induced osteoblastogenesis. In addition, SrRan was shown to increase the expression of *WNT5a*, further supporting a role of WNT5a in osteoblastogenesis[123]. Targeting the Wnt signalling may hold therapeutic potential for managing osteoarthritis and mitigating its progression.

Fibroblast growth factor signalling

Here we highlight the involvement of 5 prominent fibroblast growth factor (FGF) pathway effector genes (FGF and FGFRs) and their involvement in osteoarthritis. There are 22 FGFs, 2 of which are effector genes (*FGF1*, *FGF18*), both belonging to a paracrine subfamily. FGF18 and FGF1 share some overlapping functions, but they can exert distinct effects on different aspects of osteoarthritis pathogenesis. FGF1 is similar in structure to FGF2, for which a lot of research has been carried out, linking it with rheumatoid arthritis[124,125]. FGF1 has a

pathological role in osteoarthritis progression[126,127]. In rat cranium, FGF1 was shown to enhance bone regeneration due to angiogenic properties[128]. Angiogenesis has been observed in early-stage osteoarthritis, with serum FGF1 concentrations being positively correlated with radiographic early-stage knee osteoarthritis[129]. FGF1 levels were also shown to be significantly increased in late-stage osteoarthritis in synovial fibroblasts[130]. FGF18 demonstrates anti-osteoarthritis effects in chondrocytes via the PI3K-AKT signalling pathway and by augmenting mitochondrial fusion and fission processes[131]. FGF18 has been implicated in promoting cartilage repair and regeneration processes[132], and has shown significant healing of articular cartilage after surgical repair in a number of *in vivo* preclinical animal models involving Sprifermin, which is recombinant human FGF18[133,134] (also described in the main text). Functionally it has been shown that FGF18 regulates chondrocyte proliferation via binding to FGFR3, another effector gene, and regulates osteogenesis via binding to other FGFRs[135,136]. FGFs signal through 5 FGFRs (FGFR1-4, FGFRL1), and 3 of these are effector genes (*FGFR3*, *FGFR4*, *FGFRL1*). FGF signalling has a critical role in chondrocyte maturation and skeletal development[137]. It has been shown that dynamic balance of receptors can play an important role in osteoarthritis pathology. For example, FGF2 exerts catabolic and anti-anabolic effects in human articular chondrocytes primarily through FGFR1, whereas the beneficial effects are through FGFR3[138]. Mutations in *FGFR3* give rise to multiple skeletal disorders, such as thanatophoric dysplasia, achondroplasia, and hypochondroplasia[139]. In the growth plate, in mature chondrocytes and *in vitro* in primary chondrocytes, FGF signalling via FGFR3 can lead to attenuation of chondrocyte growth and bone formation[137,140-142]. The involvement of FGFR4 in osteoarthritis pathology is unclear; mice without *Fgfr4* develop normally. However, mice with double homozygote mutations in *Fgfr3* and *Fgfr4* demonstrate growth defects (much smaller than sibling controls) and exhibit shorter life spans; the underlying mechanism for the slow growth has not been established[143]. In neonatal mouse calvaria, the expression patterns of *Fgfr4* suggest that it may be an important regulator of osteogenesis[144]. FGFRL1 is different to the other FGFRs, in that it lacks the intracellular protein tyrosine kinase domain. It is the latest FGFR to be discovered and has been found to be expressed in skeletal tissues and postulated to be a decoy receptor[145]. In pancreatic islet beta cells, it has been shown that FGFRL1 enhances FGF signalling via SHP-1 and via MAPK/ERK signalling[146]. *MAP3K* (aka *ERK*1) is also an effector gene. The FGF pathway leads to the activation of multiple downstream pathways including, Ras/Raf/mitogen-activated protein kinase kinase/extracellular signal-regulated kinase (MAPK/ERK pathway), Phosphoinositide 3-kinase (PI3K/AKT), Phospholipase C gamma/diacylglycerol/protein kinase C (PLCγ/DAG/PKC) and signal transducer and activator of transcription (STAT) pathways. The remaining 14 effector genes are involved with the modulation of upstream and downstream signalling. For example, PIK3R1 encodes a regulatory subunit of the phosphoinositide 3-kinase enzyme, part of the PI3K/AKT signalling pathway; and SPRY2 is a negative regulator of the FGF pathway[147].

Extracellular matrix

Constituting ~90% of cartilage, the extracellular matrix (ECM) plays an important role in mechanosensing and mechanoadaption to the environment[148]. The disruption of ECM organization compromises cartilage integrity and leads to cartilage degeneration. Inflammation-triggered alterations in the ECM can disrupt cellular processes necessary for cartilage self-regeneration[149]. Degradation of the pericellular matrix may initiate changes associated with osteoarthritis[150]. Sixty-one effector genes are associated with ECM assembly. GWAS associations clustered near the *ACAN* gene have been identified for a number of phenotypes, including height[151], but none previously for osteoarthritis. The newly-identified osteoarthritis associated locus is linked to 4 osteoarthritis phenotypes, all at weight-bearing

joints (hip osteoarthritis, hip and/or knee osteoarthritis, THR and TJR). The effector gene evidence included differential splicing, methylation and protein abundance, with lower protein abundance of ACAN in degraded compared to intact cartilage. There are 2 variants in the respective 95% fine-mapping credible sets: these are rs1879529 (intronic in *ACAN*) and rs3817428 (missense in *ACAN*), which has the highest posterior probability of inclusion ranging from 0.793-0.954. rs3817428 has mixed predictions for functional impact; deleterious in SIFT and pathogenic in AlphaMissense[152], whilst tolerated in Polyphen, CADD, REVEL and METALR (http://grch37.ensembl.org). In a study investigating ACAN and lumbar back pain, rs3817428 was among 3 variants predicted to be pathogenic by *in-silico* functional analysis[153]. rs3817428 overlaps 4 *ACAN* transcripts (ENST00000352105, ENST00000439576, ENST00000559004 and ENST00000561243). In human osteoarthritis degraded and intact chondrocytes, the most abundant transcripts of *ACAN* are ENST00000558604 (40% of all transcripts) and ENST00000439576 (~30% of all transcripts) (information provided by the author of [104]). ENST00000558604 is a retained intron, whilst in ENST00000439576 rs3817428 resides in exon 15, in the G3 region of the protein which is thought to be essential for normal trafficking of aggrecan within the chondrocyte and for its secretion into the ECM[154]. This region appears to be degraded over time due to aging[155].

Circadian rhythm

The basic circadian molecular clock has a period of ∼24 hours and is intrinsic to most cells and tissues. Circadian clocks influence the timing of cellular processes, including those involved in tissue repair and maintenance. Here, we discuss the circadian relevance of the 20 effector genes related to this pathway (Supplementary Table 29, Extended Data Fig. 6). Three effector genes are key clock components. The basic core components include *CLOCK*, *ARNTL* (also known as *BMAL1*), *PER1-3* and *CRY1-2* and *NR1D1* (also known as *REV-ERBα*). *CLOCK* and *ARNTL* are both newly-identified effector genes, which form heterodimers that bind to enhancer-box (E-Box) elements and activate the expression of *PER* and *CRY*. PER and CRY proteins accumulate, dimerise and act as repressors for CLOCK/ARNTL, thereby regulating their own expression. The third effector core clock gene is *NR1D1* which is activated via E-box binding by CLOCK/ARNTL. NR1D1 is a is a nuclear hormone receptor that competes with ROR for binding to a shared DNA binding element, RORE, to regulate the expression of *ARNTL* (repression by ARNTL1 and activation by ROR) and thereby controlling the period length of rhythms in gene expression[156,157]. These 3 effector genes all have links to osteoarthritis: *ARNT*L expression was progressively reduced as osteoarthritis severity increased in human cartilage and as age increased in murine cartilage. In addition, in a chondrocyte-specific Bmal1-knockout mouse, a progressive degeneration and severity of lesions in articular cartilage of the knee (but not hip) was observed over time, indicating that *Bmal1* expression is essential in chondrocytes for preserving the structural integrity of cartilage tissue in the knee[158]. In clock mutant mice (Clock$^{\Delta 19}$), cartilage degradation and inflammation are observed[159]. The circadian rhythm pathway was shown to be significantly dysregulated in osteoarthritis-affected cartilage from human donors, with decreased expression of *NR1D1* and *ARNTL* observed in human osteoarthritis affected cartilage and also in aged cartilage compared to young cartilage in mice. It was shown that reduced expression of *NR1D*1 and *ARNTL* in chondrocytes leads to deleterious changes in TGFB signalling, which has a key role in cartilage homeostasis[160].

Six genes are involved in entrainment of the circadian clock. *NR3C1* which encodes the glucocorticoid receptor, can act as a transcriptional regulator of transcription factors or as a transcription factor binding to glucocorticoid response elements (GREs). GREs are found in the promoters of circadian clock genes[161-164] and the production of glucocorticoids are under circadian control[165,166]. The entrainment of extra-suprachiasmatic nucleus (SCN) clocks in

multiple tissues have been shown to be dependent upon the rhythmic secretion of glucocorticoids in tissues that also express *NR3C1*, and evidence suggests that misalignment between central and peripheral clocks has detrimental effects[167].

CRTC1 has been associated with major depression, obesity and circadian and sleep dysregulation[168,169]. *CRTC1* is preferentially expressed in brain tissue and regulates the transcription of cAMP response element binding protein (CREB) target genes following neuronal activity[170]. Following light stimulation, CRTC1 and CREB activate the expression of *PER* and *SIK1*. SIK1 acts in a negative feedback manner by phosphorylating and deactivating CRTC1, and suppressing the impact of light on the circadian clock[171]. CRTC1 appears to be a major player in bone osteogenesis; it has been shown that BMP2 reduces SIK1 expression resulting in the activation of CRTC1-CREB and the transcription of osteogenic genes[172].

GFPT1 is the initial rate-limiting enzyme of the hexosamine biosynthetic pathway (HBP), which is driven by nutrient availability (glucose, glutamine, acetyl-coenzyme A and uridine-5′-triphosphate) from carbohydrate, amino acid, lipid and nucleotide metabolism, respectively. The HBP is involved in the synthesis of uridine diphosphate -N-acetylglucosamine (UDP-GlcNAc), which is the substrate for N- or O-linked glycosylation and O-GlcNAcylation[173]. The activity of HBP enzymes, and thus the protein O-GlcNAcylation rhythm, is regulated by both the circadian clock and feeding-fasting[174]. In mice, it was demonstrated that ARNTL and CLOCK are rhythmically O-GlcNAcylated, which prevents their degradation, thereby altering the amplitude and phase of the clock oscillation and conveying nutritional signals to the core circadian timing system[175]. *PGM3*, a further effector gene, is also part of the HBP. HBP-related processes have been investigated as potential therapeutic targets following cartilage trauma[176], although the therapeutic efficacy of glucosamines for osteoarthritis remains controversial[177].

DDH encodes an enzyme responsible for the synthesis of 2 neurotransmitters (dopamine from L-DOPA and serotonin from L-5-hydroxytryptophan) and also synthesises tryptamine from L-tryptophan. DDC has an indirect involvement with the circadian clock via the synthesis of both neurotransmitters, which are known to modulate the circadian clock and can affect the main SCN clock[178-180]. For example, in the retina, dopamine mediates the effects of light in many circadian processes[181]. Serotonin is implicated in various processes including sleep-wake cycles[182].

MAPK9 is a member of the MAP kinase family and involved in multiple cellular processes. It is closely related to MAPK8. MAPK8 and MAPK9 (or JNKs), capable of transmitting light signals to the ARNTL–CLOCK complex[183]. They regulate the speed of oscillation and the phase response of the master clock by phosphorylating clock proteins in both positive and negative branches, affecting ARNTL protein stability and PER2 phosphorylation[184].

PRKG2 encodes a serine/threonine protein kinase that can bind to and inhibit the activation of several receptor tyrosine kinases. Mice deficient in cGKII (also known as PRKG2) are unable to reset their circadian clocks according to light, and the mechanism appears to be via activation of *Per2* and attenuation of *Per1*[185].

One effector gene, *KCNMA1*, is involved in circadian rhythm orchestration. *KCNMA1* encodes the alpha subunits of calcium-activated BK (big potassium) channels, which form the pore. KCNMA1 is an important regulator of neuronal activity in the SCN and plays a vital role in orchestrating circadian rhythms. It has been demonstrated that the expression of *KCNMA1* varies over the circadian cycle with increased expression during the night[186] which suppress

neuronal activity in the SCN during the evenings in mice[186,187]. In addition, knocking-out *Kcnma1* in mice or blocking BK channels, results in increased spontaneous firing of the SCN neurons at night, demonstrating a critical role of BK channels in regulating rhythmicity in the SCN[188-190].

Four effector genes are involved in sleeping patterns. *HCRTR2* is a high-affinity receptor for the neuropeptides orexin-A and orexin-B, and functions in sleep-awake cycles and feeding behaviour[191]. It has been associated with narcolepsy[192] and cluster headaches, which are the most severe of the primary headache syndromes typified by recurring headaches occurring in a circannual rhythm[193,194].

UTS2 is a potent vasoconstrictor[195], also expressed in the central nervous system, and plays a role in modulating rapid eye movement (REM) sleep via its receptor, which is expressed in the cholinergic neurons of the pedunculopontine tegmental nuclei, an area with a known involvement in sleep-awake patterns and important for the on- and offset of REM sleep[196-198].

CHRNB2 gene encodes a subunit of the nicotinic acetylcholine receptor. CHRNB2 mutations have been associated with autosomal dominant nocturnal frontal lobe epilepsy[199], characterised by clustered epileptic attacks during sleep. In mice, it was demonstrated that the expression of *Chrnb2* showed odor-evoked circadian rhythm under the control of BMAL1[200].

OPRL1 is an opioid receptor that binds nociception and has several roles including pain, learning and memory, emotional states, neuroendocrine control, food intake and motor control[201]. OPRL1 has a role in circadian rhythm regulation and sleep-wake control. In a study examining two regions of the prefrontal cortex in human post-mortem brain samples, *OPRL1* was one of the most rhythmic genes identified[202]. OPRL1 agonism has also been associated with sleep-wake control, increased non-REM sleep and suppression of REM sleep[203]. OPRL1 is also associated with re-entrainment of the clock following alteration in the environmental light/dark cycle by down-regulation of PER2[204]. Curcumin and curcumin-Boswellia act as nociceptin receptor antagonists and selectively down-regulate OPRL1 expression, which is associated with pain relief[205]. Their combination has been investigated for improved pain treatment in knee osteoarthritis[206].

Four effector genes are transcription factors involved in the regulation of circadian-related genes. MEF2D is involved in muscle and neuronal development and differentiation[207,208]. MEF2D binds as a homo or heterodimer to consensus sequences when activated by p38 mitogen-activated protein (MAP) kinase signalling[209]. A target gene of MEF2D, PPARGC1A, is activated in response to cytokines and exercise[210,211], and plays a role in the circadian clock response to energy, by inducing mitochondrial oxidative metabolism. Ppargc1a also stimulates the expression of Bmal1, Clock, Per2, and Rev-erbα through coactivation of ROR[212,213]. Mef2 (the MEF2D equivalent in Drosophila) has been linked to normal circadian behaviour in *Drosophila*[214] and *Mef2d*$^{-/-}$ mice demonstrate lengthened circadian free-running periods and altered behavioural activity patterns[215].

NCOA6, previously known as PRIP, is a coactivator that interacts with various transcription factors and enhances the ability of the nuclear receptors to activate the transcription of target genes. For example, NCOA6 interacts with PPARs and RXR, retinoic acid receptor, thyroid hormone receptors, glucocorticoid receptor, liver X receptor, vitamin D receptor, and androgen receptor as part of a coactivator complex that can influence the expression of

circadian clock genes. In addition, there is cross-talk between the PPARs/RXR-regulated system and the CLOCK/BMAL1-regulated system[216-218].

NKX2-1 (aka TTF1) is a transcription factor vital for the development and differentiation of various tissues, including the thyroid, lung, and ventral forebrain. In the preoptic area of the hypothalamus of rodents, the upregulation of NKX2-1 expression coincides with darkness and precedes the transcriptional oscillations of gonadotropin-releasing hormone. ARNTL1, CLOCK, and PER1 suppressed Ttf1 promoter activity and CRY1 was found to activate Ttf1 transcription. Furthermore, Ttf1 was observed to repress the transcription of *Nr1d1*[219] and Ttf1 and was shown to interact with Per2 in SCN[220].

TNF is a pleiotropic proinflammatory cytokine activating a number of signalling pathways and thus involved in the regulation of a wide range of biological processes. TNF signals through 2 main receptors; TNFRSF1A and TNFRSF1B, the latter also an effector gene. Expression of *TNF* shows a circadian pattern and there are numerous connections of TNF to the circadian clock[221]. Evidence suggests that there is a reciprocal inhibitory effect between melatonin and TNF[222]. Melatonin plays a pivotal role in circadian control. The expression levels of melatonin peak during the evening (as expression is inhibited by light). In inflammatory conditions, resulting in increased TNF, the nocturnal synthesis of melatonin can be disrupted for short periods of time[223]. In addition, TNF can activate and repress the expression of clock-related genes due to the activation of p38 Mitogen-activated protein kinases[224] and/or accumulation of calcium[225], and via TGFB and inhibition CIRBP[226], resulting in both inhibition and activation of *Per* and *Cry*, as well as activation of Rev-erbα (*NR1D1*) and *Bmal1 (ARNTL)*. Multiple studies have investigated the therapeutic effect of TNF inhibitors in cartilage repair processes[227].

Two effector genes demonstrate circadian oscillations. *ATG7* encodes an enzyme that is a key regulator in the autophagic process, essential for the conjugation of autophagy-related proteins and the formation of autophagosomes. Many autophagy genes have been shown to be regulated in a circadian-dependent manner, and *ATG7* expression and activity exhibit circadian rhythmicity. In the liver and heart, the expression of ATG7 peaks during specific times of the day, aligning with the circadian regulation of autophagy[228,229]. In the liver, CRY1 is degraded by autophagy[228] and evidence suggests that autophagic degradation of ARNTL promotes ferroptosis[230], a type of cell death shown to be associated with osteoarthritis pathogenicity[231]. Fifteen effector genes are involved in the clock-controlled autophagy in bone metabolism pathway (Wikipathways WP5205) (Supplementary Figure 12).

NTRK1 is a neurotrophic tyrosine kinase receptor, for which the main ligand is NGF. In mice, it was demonstrated that Ngf and TrkA RNA (which is the mouse homologue of the nerve growth factor receptor) oscillate in a circadian manner in the SCN, in phase with p75NTR and Per1[232] and tyrosine kinases. Additionally, upregulated expression levels of *NGF* and *NTRK1* were observed in human chondrocytes affected by osteoarthritis[233]. NGF can also promote neuronal sprouting and pain, and has been linked with neuronal plasticity[234] and contribution to peripheral inflammatory pain[235]. In addition to circadian effects on bone and joint, the severity of inflammation in the joints may vary over the course of the day due to circadian fluctuations in immune system and cytokine activity[236].

Glial cell-related

Glial cells include oligodendrocytes, astrocytes, and microglia in the central nervous system (CNS); and satellite cells and Schwann cells in the peripheral nervous system. The 39 effector genes associated with glial cells traverse multiple cellular processes, including glial cell

differentiation, regulation, migration, and development. Here, we provide a few examples: ACAN is a core ECM component also expressed by developing astrocytes and has an important role in the control of glial cell maturation during brain development[237]. *DAG1* is ubiquitously expressed, including in neuroepithelial cells during early development[238], as well as in adult glial cells[239]. DAG1 is associated with a number of neuromuscular diseases and has multiple roles in the nervous system[240], including peripheral nerve myelination[241]. Two examples of effector genes involved with astrocytes are *CLCF1* and *CDK6*. CLCF1 (also known as CLC) has the ability to differentiate neural stem cells into astrocytes within the developing brain and is associated with neuro-regeneration[242]. YAP regulation of CDK6 signalling prevents premature senescence of astrocytes, resulting in delayed brain aging and aging-related neurodegenerative diseases[243]. Further examples of effector genes associated with oligodendrocytes include *NKX2.1* and *TPPP*. Loss of Nkx2.1 function in mice causes loss of the pallidal structure and loss of GABAergic interneurons and oligodendrocytes in the dorsal telencephalic region[244]. Additionally, Nkx2.1 controls embryonic spatiotemporal astroglial production[245]. *TPPP* is specifically expressed in myelinating oligodendrocytes[246] and plays an important role in cytoskeletal rearrangement during the process elongation of oligodendrocytes[247]. Loss of RhoA-dependent immune regulation in microglia causes brain inflammation, synapse loss, neurotoxicity and memory problems. Additionally, during neuroinflammatory conditions the deficiency of RhoA can further worsen microglial necrosis and trigger apoptosis, leading to increased microglial cell death[248]. The process in which glial cells may contribute to osteoarthritis pathology and pain is complex. Here, we outline a simplified model to illustrate the different processes involved and provide osteoarthritis-relevant examples. Within the osteoarthritic joint, injury can cause the activation of immune and glial cells causing neurogenic inflammation[249] and the release of neuropeptides from sensory nerve fibres leading to local vasodilation, plasma extravasation and the recruitment of immune cells to the joint[250,251]. Glial cells can also interact with sensory nerves to regulate neurogenic inflammation and the release of inflammatory mediators (e.g. IL1, IL-6, TN, chemokines, and PGE2) that contribute to tissue inflammation and pain. In osteoarthritis, glial cell activation may enhance neurogenic inflammation in the joint, exacerbating tissue damage and pain[252]. This sustained neurogenic inflammation can further sensitise nociceptive neurons, which contributes to the maintenance of peripheral sensitisation and further amplifies the pain signalling from the joint to the central nervous system (CNS). Neuroinflammation is characterised by activation of glial cells in the peripheral nervous system (PNS) and CNS which can further drive peripheral sensitisation. Activated glial cells release pro-inflammatory cytokines and chemokines, contributing to neuroinflammation within the CNS and central sensitisation[253]. Proinflammatory responses and neuroinflammation in adult and aged mice with osteoarthritis has been found to lead to increased expression of cytokines and glia markers. In addition, the impact of pain on physical disabilities in the aged mice was found to be more pronounced[254]. In a rat model of osteoarthritis, the onset of pain-related behaviour coincided with increased sensory and sympathetic innervation of the joints and alterations in glial cells within the dorsal horn of the spinal cord[255]. In the PNS, satellite glial cells have also been implicated in the programming of dorsal root ganglia macrophages into a pro-algesic phenotype that maintains pain in osteoarthritis[256]. Glial activation and the release of pro-inflammatory cytokines and chemokines can enhance pain signalling in osteoarthritis, leading to chronic pain and hyperalgesia[257]. In rodents with induced experimental osteoarthritis, behavioural pain responses were associated with the presence of activated microglia and astrocytes in the spinal cord, indicating that osteoarthritis joint pain is associated with central sensitisation[258]. Chronic pain can manifest due to neural plasticity and due to dysfunction of glial cells[259]. Although the role of glial cells in osteoarthritis-associated chronic plain has not been fully established, glial cells have a role in central sensitisation and chronic pain

associated osteoarthritis[249,258]. The correlation of pain associated with articular joint damage in osteoarthritis is variable, and a number of clinical features have been assessed with some studies showing a correlation and others not[260,261]. In knee osteoarthritis, different markers associated with inflammation showed a weak to moderate correlation with pain[262]. Some of these observed inconsistencies may be due to differences in neuroplastic changes in the peripheral and central nervous system, which can result in a reduction of the pain threshold, allodynia and hyperalgesia in certain individuals[263,264]. Osteoarthritis can have a significant impact on an individual's mental health and well-being due to chronic pain, concerns about worsening symptoms, stress caused by coping strategies and disrupted sleeping patterns. A number of studies show a positive correlation between osteoarthritis and mental health conditions; including dementia, Alzheimer's disease, anxiety and depression[265-267]. Depression, anxiety and stress also have links with glial cell involvement.

Effector genes traversing biological processes

A total of 263 genes are implicated across the eight biological processes, with 190 being unique. Of these, 52 genes overlap between different processes. The majority of overlapping genes (38 out of 52) are shared between two processes, 8 genes are shared between three, 5 between four, and 1 gene is shared across five processes. Specifically, 28 genes are involved in the TGFB signalling pathway, with 24 of them being shared across two or more biological processes. For the other pathways, the numbers are as follows: 21 out of 33 for BMP signalling, 21 out of 57 for Wnt signalling, 7 out of 20 for FGF signalling, 29 out of 61 for ECM assembly and organisation, 5 out of 21 for Circadian rhythm, 18 out of 39 for Glial cell related pathways, and none for retinoic acid signalling (0 out of 4) (Supplementary Table 29).

Quantitative comparisons between the biological processes

We have conducted variant heritability analysis and find that the processes with the highest number of effector genes such as ECM and Wnt signalling show higher levels of osteoarthritis heritability explained, consistently across the 11 phenotypes (Supplementary Figure 9). The higher heritability attributed to these processes can be largely explained by the greater number of effector genes identified within them, but this might also reflect the fundamental roles that processes like cartilage development and remodelling play in osteoarthritis pathogenesis.

Drug Targets

Of the 473 identified approved drugs, 33 are approved for pain or inflammation in osteoarthritis, and an additional 46 drugs are approved for pain relief in other indications, and/or for other musculoskeletal diseases. The 33 drugs prescribed for osteoarthritis target just 2 genes: *NR3C1*, and PTGS1. NR3C1 is the target of 65 approved drugs, 15 of which have indications for osteoarthritis due to their anti-inflammatory action. PTGS1 is targeted by 45 drugs, including 18 nonsteroidal anti-inflammatory drugs that provide pain relief in osteoarthritis. We find a decrease of PTGS1 in degraded compared to intact osteoarthritis-affected chondrocytes (Supplementary Table 13). In humans and mouse models, *PTGS1* expression was significantly elevated in damaged cartilage and osteoarthritis-affected synovial cells compared to non-osteoarthritis-affected cells. Treatment with naproxen (one of the approved drugs) suppressed *PTGS1* expression in synovial tissue, inhibited the migratory and invasive capabilities of osteoarthritis synoviocytes and increased their apoptosis rate[268].

Ten of the effector genes associated with glial cells have proteins that are the target of approved drugs: 5 genes with approved indications for cancer treatment (*ERBB2*, *MAP2K1*, *CDK6*, *CSF1R* and *RAF1*), 2 genes for eye disease (*LAMB2 and COL6A1* (also indicated for

collagen build-up treatment)), 2 genes for autoimmune conditions (*TNF*, *NR3C1*), and *TGFB1*, which is indicated for anaemia (Supplementary Tables 29 and 30).

Three effector genes linked to BMP signalling encode proteins that are the targets of approved drugs (Supplementary Table 30) (*COL2A1*, *SOST* (described below and in the main text) and *KDR*). KDR is the target of 19 approved drugs, all of which act as inhibitors for treatment of various indications, including fibrosis. KDR (also known as VEGFR2) responds to VEGF and is involved in endothelial migration and proliferation. During hypoxia, BMP2 (via Delta like Canonical Notch Ligand 4) and BMP6 (via TAZ-Hippo) have been shown to regulate VEGF signalling by differentially modifying the availability of VEGFR2 and thereby inducing angiogenesis, suggesting that BMP2/6 may be potential targets for anti-/pro-angiogenic therapy[269].

Six proteins encoded by effector genes that are linked with the TGFB pathway are the target of at least one approved drug (Supplementary Table 30). TNF is the target of 5 drugs, all inhibitors, to treat conditions involving the immune system and inflammation, with indications for use in rheumatoid arthritis, spondylitis, ankylosing spondylitis and psoriatic arthritis. COL1A2 and COL3A1 are both targeted by 2 drugs indicated in abnormalities of connective tissue and Dupuytren's contracture. ITGB3 is inhibited by 4 drugs and has indications in myocardial infarction and recurrent thrombophlebitis. PRKCZ is the target of a drug with cancer-related indications, and TGFB1 is also targeted by a single drug, which has indications for anaemia (Supplementary Table 30).

Targeting the Wnt signalling may hold therapeutic potential for osteoarthritis. There are 5 effector genes, for which the protein is the target of an approved drug (*TGFB1*, SOS*T*, *PSMB8*, *PSMC3* and *COL6A1*). SOST antagonises Wnt signalling via binding to LRP co-receptors and also antagonises BMP-signalling[270]. Romosozumab, a monoclonal anti-sclerostin antibody, has been licenced for use in osteogenesis imperfecta, osteopenia and prevention of fractures in postmenopausal women and men with osteoporosis with promising results, although reports also suggest a higher risk of cardiovascular events[271-273].

Similarities and differences between the effector genes identified in this study, and those in large pain datasets.

We have compiled a comprehensive set of 562 variants previously reported to be associated with various pain phenotypes at genome-wide significance, derived from 33 GWAS studies (Supplementary Table 31). These variants have been mapped to 445 genes. Of the 700 osteoarthritis effector genes in our study, 41 genes (5.3%) have been classified as pain-related genes. Five out of 69 (7.2%) of the effector genes encoding proteins targeted by approved drugs have also been identified as pain-related genes. These genes are mainly associated with multisite chronic pain and pain intensity.

Supplementary Methods

Cohorts, phenotypes and genotypes

Ancestry groups were assigned according to genetic similarity with 1000 Genomes Project groupings[274] using 6 broad ancestry groups (individuals of European ancestry (EUR), East Asian (EAS) ancestry, African American (AFR) ancestry, South Asian (SAS) ancestry, Hispanic (HIS) ancestry and mixed ancestry (ADM)). Each study imputed genotypes to the most suitable reference panel available for them, which was either the Haplotype Reference Consortium panel[275] (HRC), 1000 Genomes Project[274], Trans-Omics for Precision Medicine[276] (TOPMed) or cohort-specific reference panels (Supplementary Table 2).

GWAS summary statistics quality control and meta-analysis

To check for file naming discrepancies, we used this script https://github.com/hmgu-itg/Genetics-of-Osteoarthritis-2.0/filename_checker.py. Followed by https://github.com/hmgu-itg/Genetics-of-Osteoarthritis-2.0/preQC to check that the column headers were correct, calculated the effect allele frequency, converted any odds ratios (OR) to beta and standard error (SE), excluded missing or nonsense data (e.g., *P* values > 1, infinite betas, mono-allelic variants). If applicable, we converted the linear regression coefficients to log-OR. To do this, the beta and SE were multiplied by 1/(pi*(1-pi)) where pi is the proportion of the sampled individuals that are cases[277]. Diagnostic plots were visualized, including: chromosome span, number of variants per chromosome, Manhattan plot, uniform qq-plot for *P* values, normal qq-plot for beta and SE, beta vs EAF plot and a histogram of the beta. Most of the GWAS provided were imputed to the HRC[275] (http://www.haplotype-reference-consortium.org) or 1000 Genomes Project[274] and were analysed using GRCh37/hg19. A minority of GWAS (6 cohorts) were imputed using their own reference panel or TOPMed[276] (https://topmed.nhlbi.nih.gov) using GRCh38/hg38. To harmonise across GWAS and account for differences in the reference genome build and insertion-deletion allele nomenclature, we used the following script https://github.com/hmgu-itg/Genetics-of-Osteoarthritis-2.0/runliftnorm, which implements BCFtools[278] (https://samtools.github.io/bcftools/bcftools.html) and CrossMap[279] and creates a standardized variant identifier across all datasets in GRCh37/hg19 coordinates with left-aligned insertion-deletion variants with the format chromosome:position_reference allele_alternate allele. Finally, we used Easy QC[280] to exclude variants with poor imputation quality (R^2<0.3), effective sample size <20, minor allele count <6. For some cohorts, in which the number of cases was small and there was case:control imbalance, we additionally excluded variants with effective allele count (EAC)<20 and imputation accuracy (INFO)<0.8. We checked that the *P* value matched the corresponding beta value. We checked the allele frequency against the HRC imputation reference (http://www.haplotype-reference-consortium.org/) or 1000Genomes (https://www.internationalgenome.org) to determine if there were allele coding errors. Variants with frequency below 1% were not included in the meta-analyses.

We performed sensitivity analyses to establish if including cohort summary statistics, in which the number of cases was very low, introduced errors. To do this, we performed the meta-analysis for each phenotype including only GWAS summary statistics, in which the number of cases was >1,000, >100, and across all datasets. For each phenotype, the correlation was high between the meta-analyses regardless of the number of cases. We did not observe any spurious additional signals due to inclusion of all datasets regardless of the number of cases.

Defining independent signals and loci

The stepwise conditional regression model as implemented in COJO-GCTA, uses the LD between the index variants to correct β and *P* values of neighboring variants and then runs through the same process in a stepwise manner to identify the number of independent variants in a region (Methods). Independence was declared if the variant remained significant (*P* value $\leq 1.3 \times 10^{-8}$).

Chromosome X meta-analysis

In some of the contributing datasets, the scaffold and imputation quality was lower on chromosome X compared to the autosomes. Where this was the case, each cohort converted the imputed genotype probabilities to hard calls using a threshold of 0.9 in PLINK[281]. Each cohort excluded any variant that was heterozygous in males or had a Hardy

Weinberg Equilibrium *P* value <0.0001 in females. For each set of chromosome X GWAS summary statistics, we carried out the same central quality control steps as described above as for the autosomal data. We performed a 2 step meta-analysis; in step 1, we analyzed female and male cohorts separately using METAL[282], using the inverse variance weighted approach for females and a Z-score analysis in males, to control for potential differences in allele coding between different analysis software. In step 2, we meta-analyzed the males and females using Z-scores in METAL. We observed no study-wide significant associations (*P* value $\leq 1.3 \times 10^{-8}$) on the X chromosome.

Sex-differentiated meta-analysis

The sex-differentiated analysis resulted in 468 study-wide significant variants with the majority being intergenic or intronic (57% and 29% respectively). We considered 8 lines of evidence to define effector genes, as the other lines of evidence in the effector gene scoring are based on mixed sex data and therefore not appropriate for inclusion here. Four genes scored 2, and 10 scored 1 point (Supplementary Table 6).

Genetic risk score analyses

GRS was derived using PLINK version 2.0. Only the lead variants were included in the analysis (Defining independent signals and loci). The predictive performance of GRS was assessed in multi-ancestry and ancestry-stratified MVP veterans, adjusting for covariates age, sex, and the first 10 PCs of genetic data. We contrasted the performance of the covariates-only model with the covariates plus GRS model. In an effort to disentangle the potential confounder of body mass index (BMI), we generated models with and without BMI included among the covariates (https://arocco.shinyapps.io/go2_grs/). In addition to this, we generated models where PCs were not introduced among the covariates but included in the GRS prediction to measure the overall cumulative effect of genetics on osteoarthritis prediction. We consider only the AUCs that have a significant P (P<0.05) between the individuals in the top 5% highest GRS scores compared to individuals in the lowest 5% GRS scores.
The GRS performed the best at predicting hip osteoarthritis in multi-ancestry veterans (Supplementary Table 7). When including BMI in the model, veterans with the top 5% highest GRS had a 2.4-fold increased risk of osteoarthritis in comparison with the lowest 5% of GRS scores (Supplementary Table 7). When we examined GRS performance in ancestry-stratified MVP veterans, veterans in the top highest 5% GRS scores were 2.5-fold more likely to have hip osteoarthritis compared to the lowest 5% of GRS scores, whereas African American and Admixed American veterans in the top 5% of the GRS were 1.5 and 2.2. fold more likely to have hip osteoarthritis than those in with the lowest GRS, respectively (Supplementary Table 7).

Effector gene identification by combining 24 lines of evidence

Determination of genes in osteoarthritis-associated loci

We produced a list of all of the genes in each locus using the Ensembl REST API (http://grch37.rest.ensembl.org; Methods) using overlap/region/human. As different databases can give slightly different results and in order to be more comprehensive, we also performed the same search using University of California Santa Cruz (UCSC) Genome Browser[283] and added additional genes with ENSG identifiers to the list for each locus. For the 286 loci, we extracted 8,785 unique genes, based on ENSG identifier.

Variant annotation and fine-mapping

For each variant in the credible set, we added all annotations available using Ensembl[284] (http://grch37.ensembl.org/Tools/VEP), for each locus with no credible set, we included the

lead variant only. We extracted the most severe consequence for each variant along with the associated gene and impact rating (https://www.ensembl.org/info/genome/variation/prediction/predicted_data.html) (Supplementary Table 9). For effector gene scoring, we used an impact of moderate to high. A variant annotation with an impact of moderate to high corresponds to the following high Sequence Ontology (SO) terms: transcript_ablation, splice_acceptor_variant, splice_donor_variant, stop_gained, frameshift_variant, stop_lost, start_lost, transcript_amplification, feature_elongation, feature_truncation and the following moderate SO terms: inframe_insertion, inframe_deletion, missense_variant, protein_altering_variant. In addition, we scored a gene as being potentially important if all of the variants in the credible set reside within the canonical transcript of the gene. For the 84 moderate to high impact variants, we identified the gene affected and gave it a score of 1 point for the effector gene ranking. We identified 142 genes, in which all variants in the credible set reside within the transcript (Supplementary Tables 9 and 13).

Active promoter

To glean as much information as possible from the non-coding credible set variants, we performed an *in-silico* search to identify putative transcription factor binding motifs that are altered by the variant. We used the HaploReg version 4.2[14] database from the ENCODE project[14,285] to identify potential promoters that are altered by the variant. We included all credible set variants, and for each locus with no credible set, we included the lead variant only. We identified 34 variants situated in active histone marks related to a promoter and predicted TATA-BOX motif and annotated these manually to the closest gene. For 5 variants, there was no gene nearby (the nearest gene was > 3kb away). We scored 23 unique genes a score of 1 point for the effector gene ranking (Supplementary Tables 13 and 15).

Chondrocyte Hi-C loop overlap

To consider the 3-dimensional spatial organization of the genome in chondrocytes and search for long-range regulatory elements, we integrated high-throughput chromosome conformation capture (Hi-C) data generated in patient-derived primary chondrocyte cells[286]. We utilised the ROADMAP epigenome Chromatin Hidden Markov Models(HMM) 15-state model[287], which identifies the following: Active Transcription Start Site (1_TssA), Flanking Active Transcription Start Site (2_TssAFnk), Transcription at gene 3' and 5' (3_Tx_Flnk)., Genic Enhancers (6_EnhG), Enhancer (7_Enh),Bivalent/Poised Transcription Start Site (10_TssBiv), Flanking Bivalent/Poised Transcription Start Site (11_BivFlnk) and Bivalent Enhancer (12_EnhBiv). We used Mesenchymal stem cell-derived chondrocytes as a model cell type (E049)[287]. The HiC contact file was lifted to GRCh37 with HiCLift[288]. We overlayed the credible set variants with loop anchor regions identified with 4 different loop calling algorithms using *P* value or FDR ≤ 0.1. To determine if any of these reside in an enhancer region, we checked if those variants residing in a loop anchor region overlap with any of the ChromHMM states 3,6,7,11 or 12. To further check if the loop anchor containing a variant in an enhancer is in contact with a loop anchor containing a promoter, we checked if the contact loop anchors contain regions with ChromHMM states 1,2 or 10 (Supplementary Table 16). To link the promoter to a gene, we used -1,500 bp and +500 bp relative to the transcriptional start site (TSS) using Ensembl BioMart[289] (release 110, GRCh37). We used the ENSG identifier for the mapping to the gene list, and for each locus with no credible set, we included the lead variant only (Supplementary Table 17). We score 1 point to all genes in the list linked to an enhancer-promoter variant (Supplementary Table 13). We identified 10,078 variants residing in a loop region, 836 residing in an enhancer and, of these, 814 variants looped to a promoter, for 1155 unique genes. Fifty-two percent of the 1,155 unique genes (n = 599) were among the 8,785 genes in the GWAS associated loci; 493 genes were in the

same locus as the looped enhancer variant, 89 genes were in a different locus and 17 genes were linked to looped enhancer-variants in the same and different loci.

Genes involved in gene regulatory networks important in fetal development

To identify if any of the credible set variants were located in an enhancer that is linked to a gene involved in gene regulatory networks important in foetal development, we interrogated data from the development atlas. Single cell multi-ome data (scRNA-seq and scATAC-seq) were generated in hip, knee and shoulder tissue to investigate osteogenesis and chondrogenesis (personal communication). The data were analysed using the GRNBoost2 algorithm in Scenic+ software[290], which quantifies co-expression and accessibility. We used 2 metrics associated with the enhancer-gene links; the importance and the correlation coefficient to filter the data. These 2 values are used for the purpose of matching TF with bound regions and target genes. Initially, the links were filtered by rho (|rho| > 0.03) and then sorted by importance score[290], we selected the top (most important) 3 regions per gene and investigated if any of the credible set variants reside in these regions. In order to map the variants, we firstly lifted the region coordinates from GRCh38 to GRCh37 using the UCSC Genome Browser[291] (http://genome.ucsc.edu) and scored the gene if variants in the credible set reside in this window. For each locus with no credible set, we included the lead variant only. We find 146 unique genes in which the credible set variants are located in an enhancer-gene linked region involved in gene regulatory networks important in the development of the fetal skeleton. Ninety-eight are associated with chondrogenesis, 81 with osteogenesis and 33 associated with both developmental processes (Supplementary Tables 13 and 18).

Multi-omics in primary osteoarthritis cartilage

We performed a look-up for all genes located in the 286 osteoarthritis loci using the largest functional datasets available in chondrocyte osteoarthritis tissue to determine if any of the genes in the loci were differentially expressed. We investigated differences between paired low-grade (intact) cartilage and high-grade (degraded) cartilage from individuals who had undergone joint replacement due to osteoarthritis. We examined differences in terms of gene expression (n=124 individuals), protein abundance (n=99 individuals) and methylation (n=90 individuals)[104,109,292]. We used the published definitions for declaring differential status. For the gene expression and protein abundance, Ensembl gene name and ENSG identifier were used to perform the search. For differential methylation, we included methylation sites annotated to at least one gene name based on the Illumina manifest file for the 450k array. For methylation, the Ensembl gene name and aliases were used for the search and mapping for the effector gene scoring. Aliases were identified using HUGO Gene Nomenclature Committee (HGNC) Biomart (https://biomart.genenames.org). Seventy-six genes were differentially expressed, 31 had lower expression and 45 had increased expression in degraded compared to intact cartilage. Seventeen genes and 18 transcripts demonstrated differential usage; 8 transcripts had increased usage and 10 transcripts had decreased usage in degraded compared to intact cartilage. Thirty-nine genes were differentially spliced in degraded compared to intact cartilage. We identified 431 genes associated with differential protein abundance, 241 with decreased abundance and 190 with increased abundance in degraded compared to intact cartilage. There were 1,297 genes associated with at least differentially methylated site between degraded and intact cartilage (Supplementary Table 13).

Colocalization with molecular QTLs in osteoarthritis relevant tissues

We performed statistical colocalization analyses in primary osteoarthritis high-grade (degraded) cartilage, low-grade (intact) cartilage and synovium. For synovium, we used gene

expression (n=77 individuals)[292] and methylation (n=78 individuals) QTL data from osteoarthritis patients[109]. Cartilage data from osteoarthritis patients were collected from degraded and intact tissue and included gene expression (degraded: n=87 individuals, intact: n=95 individuals), protein abundance (n=99)[292] and methylation (degraded: n=90 individuals, intact: n=98 individuals) QTL data[109]. For the loci that included more than one independent variant, we performed conditional analyses with COJO[293] to generate adjusted summary statistics for each index variant by conditioning all variants in the locus on the other independent index variants. For each signal and each QTL dataset, we included all variants in the respective window. Colocalization was conducted for each respective molecular feature (for instance gene, protein or CpG sites) that contained at least one QTL (using a threshold of <5% false discovery rate). In addition, for the methylation data, colocalization was conducted only if the CpG site was annotated to a gene, based on the Illumina manifest file for the 450k array. We used the *coloc.abf* function from the *coloc* R package (version 5.2.2)[294]. We used the default prior probabilities of the *coloc* R package. We considered evidence for suggestive colocalization if the posterior probability of H4 (PP4) was > 0.8. For each suggestive colocalizing signal, we calculated a 95% credible set for the causal variant by taking the cumulative sum of the variants' posterior probabilities to be causal, conditional on H4 being true. We considered robust evidence of a shared signal if the 95% credible set of the colocalization overlapped at least by one variant with the 95% credible set of the GWAS fine-mapping or the lead GWAS risk variant (Supplementary Table 19). If a methylation site was associated with >1 gene, these genes were both scored (Supplementary Table 19).

Human and mouse musculoskeletal and pain/neuronal phenotypes

We performed bioinformatics searches to identify genes in the loci involved in mouse or human musculoskeletal phenotype or linked to a pain or neuropathy disorder. Genes associated with mouse musculoskeletal phenotypes (code MP:0005390 (skeleton phenotype), MP:0005369 (muscle phenotype) and MP:0005387 (immune system)) or neuronal phenotype (code MP:0003631 (nervous system phenotype) were retrieved from the Mouse Genome Informatics (MGI) database (www.informatics.jax.org) and International Mouse Phenotyping Consortium (IMPC) (http://www.mousephenotype.org/). Genes associated with human monogenic musculoskeletal phenotypes (MeSH code C05) were identified from Online Mendelian Inheritance in Man (OMIM) database (https://www.omim.org/). For human genes linked to monogenic pain disorders, we extracted any genes containing phenotypes with the key words pain and sensory neuropathy in the OMIM database. Additionally, we identified genes associated with human musculoskeletal phenotypes according to the Nosology and classification of genetic skeletal disorders[295]. We also searched the Butterfield *et al* publication[296] for genes identified as having a role in abnormal joint formation in mice, in data generated by the Origins of Bone and Cartilage Disease Project. Additionally, we downloaded the gene lists which have been linked to pain from the curated Human Pain Genetics Database (https://humanpaingeneticsdb.ca/hpgdb/). We found 204 genes to be linked to a human musculoskeletal phenotype, 57 to a monogenic human pain disorder and 96 genes to be associated with pain in the curated Human Pain Genetics Database (Methods). In the mouse searches, 1,358 genes were linked to a musculoskeletal phenotype and 763 to a pain phenotype.

Lines of evidence assessment

To investigate if certain lines of evidence are more informative than others, we extracted 77 known osteoarthritis unique effector genes from Boer, Hatzikotoulas, Southam *et al.*[297]. We identified which of these were from loci previously reported (n=48). *LMX1B* is present at 2 different loci (1 known and 1 novel) we considered this gene as previously reported. There

are 18/77 genes that are not included in this study, this is likely due to locus definition differences between Boer, Hatzikotoulas, Southam *et al.* and this study as 11/18 reside within 500kb of loci in this study. There were 2 genes *TACC3* and *ENHO* that are not considered as effector genes in this study because they score <3, the difference is due to updated or different datasets utilised in this study compared to that in Boer, Hatzikotoulas, Southam *et al* for the colocalisation and differential analysis respectively. For the analysis we used 57 known osteoarthritis genes from Boer, Hatzikotoulas, Southam *et al* and we also counted the lines of evidence separately for the 36 of these that were previously reported (Supplementary Table 13). To establish the sensitivity, we divided the counts for each line of evidence by 57 and 36 for the known osteoarthritis from Boer, Hatzikotoulas, Southam *et al.* and previously reported osteoarthritis genes, respectively (Supplementary Figure 8). Additionally, we estimated variant heritability of each line of evidence by using LDAK v6 software[298] (https://www.ldak.org). We used summary statistics from the meta-analysis for each of 11 binary traits.

To further investigate if any line of evidence is more informative from a GWAS variant perspective, we mapped the implicated genes from each line of evidence to their corresponding GWAS loci, selecting the lead variant for each locus. The effect sizes and posterior probabilities of these lead variants were then compared using one-way ANOVA and Tukey's Honestly Significant Difference post-hoc test (Supplementary Tables 13, 21 and 22).

Biological insights additional analysis

Pathway analysis

We included all human genes as the background, with the Gene Ontology biological processes as the reference. The thresholds used for the minimum and maximum number of genes in each pathway were 10 and 300, respectively. Over-represented gene sets required a minimum of 5 genes to overlap with the examined gene set. The significance threshold was set at FDR < 0.05. Pathway analyses were performed using the R package ClusterProfiler[299] (version 4.8.2) (Supplementary Table 26, Extended Data Fig. 3, Supplementary Note).

Subchondral bone allelic imbalance

Details on RNA isolation, RNA-sequencing, alignment, and quality control are provided elsewhere[300]. The approach to determine AEI was based on two previous studies[301,302]. In short, variants located in the coding region in high LD ($R2>0.6$) with the fine mapped variants in the credible set were identified, for each locus with no credible set we included the lead variant only. In total, we used 262 variants. The LD reference panel used was 1000 Genomes CEU. Subsequently, the count fraction of the alternative allele among the alternative and reference allele together (φ) was calculated for each heterozygote. Then, a meta-φ and FDR were generated by performing a meta-analysis. An FDR<0.05 was considered a significantly different allelic expressed imbalance compared to the null hypothesis genomic median allelic imbalance: φ=0.49 (Supplementary Note, Supplementary Table 27, Supplementary Figure 10).

Carriage of risk alleles by pathway

Treatment and/or clinical trial success may be enhanced by targeting patients who harbor risk alleles associated with effector genes that are involved in a pathway related to the mode of action/target of the drug. We investigated how many osteoarthritis patients carry risk alleles associated with the effector genes for each of the 8 pathways. We investigated 3 osteoarthritis phenotypes: osteoarthritis at any site, knee osteoarthritis and hip osteoarthritis, and restricted the analyses to individuals of European ancestry, due to sample

size considerations, from the UK Biobank (82,420 with osteoarthritis at any site; 25,293 with knee osteoarthritis; and 16,867 with hip osteoarthritis) and MVP (56,848 with osteoarthritis at any site, 37,814 with knee osteoarthritis and 11,783 with hip osteoarthritis). For each of the 8 pathways (retinoic acid signalling, TGFB signalling, BMP signalling, Wnt signalling, FGF signalling, ECM assembly and organization, circadian rhythm, and glial cell related), we mapped each of the pathway-associated effector genes to their respective locus (Supplementary Table 33). For each variant, we counted 1 if a case included at least 1 risk allele and 0 otherwise. For UKBB: BGEN bgenix was used to extract the variants[303]. PLINK[281] was used to extract the cases and genotypes, and R version 4.3.2 (2023-10-31)[304] was used to count the number of risk alleles. Three variants were not included in the UK Biobank genotyping data (Supplementary Table 32, Extended Data Fig. 7). For MVP: PLINK2 was used to extract the cases and genotypes, and R version 4.0.3 was used to count the number of risk alleles (Extended Data Fig. 8).

Transcription factor enrichment

There were 1,585 unique variants located in a gene regulatory region that significantly altered a regulatory transcription factor (TF) binding motif. These variants affected in total 344 unique transcription factors. We performed enrichment analysis after generating TF motif background counts. The enrichment was established using a propensity test in R (*prop.test* function), using the generated background counts as the background input for the enrichment calculation. The significance threshold (Bonferroni correction) was set to the number of variants tested (1,585) and number of TF factors tested (344), and we report 57 significantly enriched TFs (*P* value $<9.17 \times 10^{-8}$) (Supplementary Table 12, Supplementary Figure 7). The HaploReg resource[14] constructed a library of position weight matrices (PWMs) based on motif-finding analysis of the ENCODE project and scored if variants had an effect on the regulatory motif using the PWMs[14,285]. Only variants that significantly (P value $<4 \times 10^{-8}$) changed the PWM, and significantly altered the transcription factor binding motif, were reported. Transcription factor binding motif naming convention was harmonized to identify the number of unique transcription factors (example: TATA_known01 and TATA_known02 were harmonized to TATA).

To establish a random background set, we used a previously generated list of independent variants associated with unique GWAS phenotypes, as previously described[305]. Briefly, only phenotype-associated variants with genome-wide significant associations (P value $<5 \times 10^{-8}$) and variants that were present in populations of European descent were selected. To ensure independence, variants with a $r^2>0.1$ and <100kb away from a more strongly associated GWAS lead variant were removed. This resulted in a set of 56,182 LD-pruned variants scattered throughout the genome[305]. Using this list of independent phenotype-associated variants, we performed the same analysis as we have done for all our identified osteoarthritis associated variants. We selected at random 285 variants and their LD-block (variants in high LD ($r^2 \geq 0.8$)). From this list, we selected all variants that overlapped with gene regulatory regions in human mesenchymal stem cell-derived chondrocytes and altered a transcription factor binding motif. We repeated this analysis for 1000 permutations. Finally, we combined all data to produce the average occurrence of TF motifs for use as a background set for the enrichment analysis, which consisted of a propensity test in R (prop.test function).

Biological Insights

We used information from the pathways analysis and Enrichr[306,307] to check the pathway membership across different sources. We combined gene set information from Gene

Ontology[105,106], Reactome[308], Wikipathways[309] and manual curation based on literature to interconnect the genes.

Cohort Descriptions

arcOGEN

Arthritis Research UK Osteoarthritis Genetics (arcOGEN) is a collection of 7410 unrelated, UK-based individuals of European ancestry with knee and/or hip osteoarthritis from the arcOGEN Consortium. Samples were collected in 2 stages from 10 United Kingdom locations (London, Nottingham, Oxford, Sheffield, Southampton, Edinburgh, Newcastle-Upon-Tyne, Sheffield, Wansbeck, and Worcester). The majority of cases had primary osteoarthritis requiring joint replacement of the hip or knee while a smaller number were ascertained by radiographic evidence of disease (Kellgren-Lawrence (KL) grade ≥2). The exclusion criteria included the need for joint replacement due to fracture, secondary osteoarthritis of any cause, and developmental, vascular, or infective causes of joint disease.
https://www.arcogen.org.uk United Kingdom

ARGO-Athens

The ARGO study was set up to investigate the genetic architecture of hip and knee osteoarthritis in a Greek population. More than 1,500 patients with severe osteoarthritis, undergoing hip and/or knee total joint replacement were recruited from the cities of Athens and Larissa. The ARGO collection was conducted in three public hospitals (Attikon University General Hospital of Athens, Nea Ionia General Hospital Konstantopouleio, and KAT Hospital) and one private hospital (Lefkos Stavros General Hospital) in the city of Athens, Greece between February of 2015 and March 2017. All studies were approved by the relevant hospital Institutional Review Board and conducted in accordance with the principles set out in the Declaration of Helsinki. All patients provided written informed consent prior to participation.

ARGO-Larissa

The ARGO-Larissa study was set up to investigate the genetic architecture of knee osteoarthritis in a Greek population. It included individuals with primary knee osteoarthritis undergoing total knee arthroplasty. The osteoarthritis participants' recruitment was conducted in the city of Larissa, central Greece. Verbal informed consent was given by all research participants prior to the collection of blood samples for the research. The research participant recruitment, consent process, and study protocol were approved by the Institutional Review Board of the University Hospital of Larissa and conform to the ethical principles set out in the Declaration of Helsinki (1975).

BioMe BioBank Program

The BioMe BioBank Program is dedicated to advancing the application of human blood-derived biospecimen and clinical data to life science research to accelerate the development of personalized healthcare and medical solutions. From September 2007, over 65,000 Mount Sinai Health System patients have enrolled in the Electronic Medical Record-linked Bio Me Biobank Program. The program is designed to generate a large collection of DNA and plasma samples, phenotypic (questionnaire-based and EMR-linked) and genomic data, that are stored in a way that protects the patient's privacy. The three major self-reported racial/ethnic populations include 33% EA (European Ancestry), 22% AA (African Ancestry, 35% HA (Hispanic Ancestry). The biobank enables research to be performed on de-identified, comprehensive, electronic clinical information extracted from the Mount Sinai Data Warehouse (MSDW). More than 31,900 of the BioMe participants have genotype data available for genetic analyses. Data include whole exome sequencing, genome-wide genotyping array (GSA), and for a subset of participants (N~12,000) whole genome sequencing data is available.

Bunkyo Health Study

Bunkyo Health Study is a prospective cohort study of over 10 years, which recruited older subjects aged 65–84 years living in Bunkyo-Ku, an urban area in Tokyo, Japan. Among the 68 communities in Bunkyo-Ku, we selected 13 communities based on probability proportionate to size sampling. We obtained the name and address of all residents aged 65-84 years in the selected communities from residential registries. The exclusion criteria were to have a pacemaker or defibrillator placement and diabetes mellitus requiring insulin therapy.

China Kadoorie Biobank

CKB is a prospective cohort study of 512,713 adults aged 30-79 years, recruited between 2004 and 2008 from 5 urban and 5 rural areas across China. Baseline information included a laptop-based questionnaire, physical measurements, and blood sample collection. Incident osteoarthritis outcomes were identified from long-term follow up through electronic linkage of each participant's unique national identification number to the Chinese national health insurance system.

deCODE

The deCODE genetics osteoarthritis study is an ongoing population based study in Iceland that was initiated in 1997. The study includes information on all subjects who have undergone total joint replacement in Iceland, and on osteoarthritis status from the Landspitali University Hospital electronic health records. Information on hand osteoarthritis patients is derived from a database of hand osteoarthritis patients that was initiated in 1972. Subjects have given blood or buccal samples to deCODE genetics biobank, which has gathered genotypic and medical data from more than 160,000 volunteer participants. https://www.decode.com/.

The details of osteoarthritis definition and analyses have been described previously[10,310].

CHB+DBDS

The Danish cohort is based on material from 2 separate studies, 1) Genetics of Pain and Degenerative Musculoskeletal Diseases – a Genome-Wide Association study on repository samples from Copenhagen Hospital Biobank (CHB) and 2) the Danish Blood Donor Study (DBDS). Both cohorts are supplemented with data from high-quality National Danish Health Registers, including the Danish National Patient Register, the Central Person Registry, the Danish Knee Arthroplasty Register, and the Danish Hip Arthroplasty Register. CHB is a regional biobank in which leftover EDTA whole blood samples from type and screen analyses are stored for patients admitted to hospitals in the Capital Region of Denmark[311]. CHB contains samples from >450,000 patients, and in this study, genome-wide genotype data (Illumina Infinium Global Screening Array) is available for >250,000 patients under the present study (approval number: NVK-1803812, P-2019-51). DBDS is a prospective cohort that includes >140,000 Danish Blood Donors[312]. Whole blood and plasma samples are consecutively collected for all DBDS donors, along with phenotypic data from questionnaires and National Danish Health Registers. Additionally, DBDS participants have been genotyped using Illumina Infinium Global Screening Array. Eligibility for inclusion in DBDS is equivalent to eligibility for blood donations, which includes being 17-70 years old, weighing >50 kg, and having overall good health (full, updated list of eligibility criteria is available at https://bloddonor.dk/). Blood donation and participation in DBDS is voluntary and unpaid. DBDS data are included under approval number NVK-1700407, P-2019-99).

eMERGEIII

The Electronic Medical Records and Genomics (eMERGE) network is a network of medical centers with electronic medical records linked to existing biorepository samples for genomic discovery and genomic medicine research. The eMERGE phase III cohort includes 105,108 individuals recruited from 12 contributing medical centers with deidentified demographics, diagnosis information based on the International Classification of Diseases (ICD) codes, and genotyping data. Genetic data from 83 Illumina and Affymetrix genotype array bathes were

unified and imputed to HRC1.1 panel using minimac 3 algorithm via Michigan Imputation Server[313].

Estonian Biobank

The Estonian Biobank cohort is a volunteer-based sample of the Estonian resident adult population (aged ≥18 years). Estonians represent 83%, Russians 14%, and other nationalities 3% of all participants. The current number of participants is > 205,000 and represents a large proportion, >15%, of the Estonian adult population, making it ideally suited to population-based studies. General practitioners (GPs) and medical personnel in the special recruitment offices have recruited participants throughout the country. At baseline, the GPs performed a standardised health examination of the participants, who also donated blood samples for DNA, white blood cells and plasma tests and filled out a 16-module questionnaire on health-related topics such as lifestyle, diet and clinical diagnoses described in WHO ICD-10. A significant part of the cohort has whole genome sequencing (3000), whole exome sequencing (2500), genome-wide single nucleotide polymorphism (SNP) array data (200 000) and/or NMR metabolome data (11 000) available. The data are continuously updated through periodical linking to national electronic databases and registries. A part of the cohort has been re-contacted for follow-up purposes and resampling, and targeted invitations are possible for specific purposes, for example people with a specific diagnosis. For the current GWAS data freeze including approximately 205,000 gene donors was applied. Osteoarthritis cases were chosen from ca 205000 participants of Estonian Biobank by using ICD 10 codes. To specify THR, TKR and TJR cases, the codes from NOMESCO Classification of Surgical Procedures were used in addition (https://genomics.ut.ee/en/access-biobank)[314].

FinnGen

The FinnGen study is a large-scale genomics initiative that has analyzed over 500,000 Finnish biobank samples and correlated genetic variation with health data to understand disease mechanisms and predispositions. The project is a collaboration between research organisations and biobanks within Finland and international industry partners.

GeisingerMyCode_F60k and GeisingerMyCode_New30k

Geisinger is an integrated health care provider located in central and northeastern Pennsylvania and New Jersey. Geisinger's electronic health record (EHR) consists of comprehensive longitudinal clinical information including patients' demographic data, diagnoses (including co-morbidities), lab measurements, prescriptions, procedures, vital signs, and, of relevance for this study, surgical procedure logs. The EHR captures a median of 14 years of health data for patients within the MyCode®Community Health Initiative biorepository. Through the Geisinger-Regeneron DiscovEHR collaboration, whole exome sequence and genome wide genotype data are available from more than 92,000 MyCode® participants to date. These high dimensional clinical data linked to genetic data provide opportunities to conduct precision health research at an unprecedented scale that can lead to significant clinical insights. https://www.geisinger.org/precision-health/mycode
The details of MyCode Community Health Initiative have been described previously[315]. Genotyping was performed in two batches on the Illumina Infinium OmniExpress Exome array and GSA-24v1-0 array for Geisinger 60k and Geisinger 30k cohorts, respectively. The Michigan Imputation Server was used to impute genotypes for both cohorts to HRC.r1-1 EUR reference genome (GRCh37 build) separately. Pre-imputation QC included sample call rate and marker call rate >90%, HWE p-value >1e-15, MAF>1%. A/T & G/C SNPs were removed if MAF > 0.4. SNPs with differing alleles, SNPs with > 0.2 allele frequency difference, SNPs not in HRC reference panel were also removed. Variants with imputation info score > 0.3 and MAC >5 were included in the analyses. We used ICD-code based method to define osteoarthritis cases and controls. We adopted a linear mixed model built in BOLT_LMM (N>5000) or GEMMA (N<5000) for the association tests while accounting for the relatedness

and population structure (first 20PCs). PLINK1.9 was used for genetic data quality control and PC calculation.

Generation Scotland

The Generation Scotland: Scottish Family Health Study (GS:SFHS) is a family-based population cohort with DNA, biological samples, socio-demographic, psychological and clinical data from approximately 24,000 adult volunteers across Scotland. Although data collection was cross-sectional, GS:SFHS became a prospective cohort due to of the ability to link to routine Electronic Health Record (EHR) data. Over 20,000 participants were selected for genotyping using a large genome-wide array.

Genes & Health

Genes & Health is a long-term longitudinal population study of target 100,000 volunteers (currently 62,000 recruited) of British-Bangladeshi and British-Pakistani origin, adults aged 16 or over, living in England. Data is linked to NHS health record data (primary care, hospital secondary care, and national NHS England) using NHS number. Oragene saliva DNA was provided at enrollment, and genotyped using Illumina GSAv3EAMD and TOPMED-r2 imputation.

Health and Retirement Study

The Health and Retirement Study (HRS) is a longitudinal survey of a representative sample of approximately 20,000 people in America over the age of 50. The study interviews respondents every two years about income and wealth, health and use of health services, work and retirement, and family connections. DNA was extracted from saliva collected during a face-to-face interview in the respondents' homes. These data represent respondents who provided DNA samples and signed consent forms in 2006, 2008, 2010, and 2012.

Hong Kong Degenerative Disc Disease Population Cohort (HKDDDPC)

Hong Kong Degenerative Disc Disease Population Cohort (HKDDDPC) is a population-based cohort with subjects openly recruited via newspapers advertisement, posters and e-mails, regardless of their social and economic status. The study call was for any participant who agreed to a study on the lumbosacral spine with MRI, clinical questionnaires and follow-up assessments. Participants with prior surgical treatment of the spine, spinal tumours and fractures, and marked spinal deformities were excluded from the study. Subjects selected were not based on the presence or absence of clinical symptoms. All qualified subjects underwent T1-weighted axial MRI and T2-weighted sagittal MRI of the lumbosacral spine (L1-S1) after informed consent was obtained from participants and ethics was approved by a local institutional board. MRI Protocol: 1.5T or 3T MRI machines were used for axial and sagittal imaging at L1-S1. Subjects were oriented in the supine position. For T1-weighted axial scans, the field of view was 21cmx21cm, slice thickness was 4mm, slice spacing was 0.4mm, and imaging matrix was 218x256. For T2-weighted sagittal scans, the field of view was 28cm[illegible]28cm, slice thickness was 5mm, slice spacing was 1mm, and imaging matrix was 448[illegible]336. The repetition time for T1- and T2-weighted MRI were 500ms-800ms and 3320ms respectively, and their echo time was 9.5ms and 85ms. According to the pedicle and disc levels, 11 parallel slices were made at each spinal level with reference to the adjacent endplates. Definition of osteoarthritis on x-rays: For the lumbar lateral radiographs, each disc level (L1-2, L2-3, L3-4, L4-5, L5-S1) was measured for osteophytes and vertebral narrowing. Grade 0 was considered none; grade 1 was mild; grade 2 was moderate; and grade 3 was severe. The L5-S1 disc was narrowed when its height was less than the disc space of L3-4. Diagnosis of osteoarthritis was based on the criteria as described by de Schepper et al[316]. Disc space narrowing was present with grade ≥1 and osteophytes with grade ≥2. Osteoarthritis had either “narrowing”, “osteophytes” or “both”. Narrowing was considered with grade ≥1 narrowing at 2 or more vertebral levels and osteophytes were considered with grade ≥2 at 2 or more vertebral levels. With both narrowing and osteophytes, then “both”

was considered. Conversion of MRI for osteoarthritis diagnosis: Conversion of MRI for osteoarthritis diagnosis was dependent on two MRI ratings: disc bulging and Schneiderman score. Disc bulging was divided into 4 categories: 0 = no disc bulging; 1 = posterior disc bulging (disc displaced beyond a virtual line connecting the posterior edges of two adjacent vertebrae); 2 = disc extrusion (distance between the edge of the protruded disc into the spinal canal was greater than the distance between edges of the base of the disc); 3 = disc sequestration (disc material detached and migrated away from the level of the intervertebral disc)[317-319]. The Schneiderman score[320] was used to describe the disc signal intensity and was evaluated by a 4-point scale: 0 = normal disc height and signal intensity; 1 = speckled pattern or heterogeneous decreased disc signal intensity; 2 = diffuse loss of signal; 3 = signal void. Each lumbar intervertebral disc was rated for disc bulging and Schneidermann score. Lumbar spine osteoarthritis was defined to be present if (1) At least 1 disc with Schneidermann score 3, OR (2) At least 1 disc with Schneidermann score 2 and Bulging score 2, OR (3) At least 2 discs with Schneidermann score 2 AND disc bulging score 1, There were 587 of subjects with X-rays as well. We validated our definition based on MRI by testing and confirming its equivalence with X-ray diagnoses using these subjects. DNA samples were genotyped using the Illumina humanOmniZhongHua-8 v1.2 BeadChip. Quality control (QC) of the genotyped data were conducted based on pipeline provided by Anderson et al[321]. Imputation of single nucleotide polymorphisms (SNPs) was performed using reference panels from the Haplotype Reference Consortium (HRC)[322]. We wish to acknowledge grant support from the Hong Kong Research Grant committee: AoE/M-04/04, T12-708/12-N and C7044-19G.

HUNT study

The Trøndelag Health Study (HUNT) is a large population-based cohort from the county Trøndelag in Norway. All residents in the county, aged 20 years and older, have been invited to participate. Data was collected through three cross-sectional surveys, HUNT1 (1984-1986), HUNT2 (1995-1997) and HUNT3 (2006-2008), and has been described in detail previously[323], with the fourth survey recently completed (HUNT4, 2017-2019). DNA from whole blood was collected from HUNT2 and HUNT3, with genotypes available from 71,860 participants. All genotyped participants have signed a written informed consent regarding the use of data from questionnaires, biological samples and linkage to other registries for research purposes. The current study was approved by the Regional Committee for Medical and Health Research Ethics (REK) 2015/573. https://www.ntnu.edu/hunt. Quality control: In total, DNA from 71,860 HUNT samples was genotyped using one of three different Illumina HumanCoreExome arrays (HumanCoreExome12 v1.0, HumanCoreExome12 v1.1 and UM HUNT Biobank v1.0). Samples which failed to reach a 99% call rate, had contamination > 2.5% as estimated with BAF Regress[324], large chromosomal copy number variants, lower call rate of a technical duplicate pair and twins, gonosomal constellations other than XX and XY, or whose inferred sex contradicted the reported sex, were excluded. Samples that passed quality control were analysed in a second round of genotype calling following the Genome Studio quality control protocol described elsewhere[325]. Genomic position, strand orientation and the reference allele of genotyped variants were determined by aligning their probe sequences against the human genome (Genome Reference Consortium Human genome build 37 and revised Cambridge Reference Sequence of the human mitochondrial DNA; http://genome.ucsc.edu) using BLAT[326]. Variants were excluded if their probe sequences could not be perfectly mapped to the reference genome, cluster separation was < 0.3, Gentrain score was < 0.15, showed deviations from Hardy Weinberg equilibrium in unrelated samples of European ancestry with p-value < 0.0001), their call rate was < 99%, or another assay with higher call rate genotyped the same variant. Imputation was performed on the 69,716 samples of recent European ancestry using Minimac3[275] (v2.0.1, http://genome.sph.umich.edu/wiki/Minimac3) with default settings (2.5 Mb reference

based chunking with 500kb windows) and a customized Haplotype Reference consortium release 1.1 (HRC v1.1) for autosomal variants and HRC v1.1 for chromosome X variants[322]. The customized reference panel represented the merged panel of two reciprocally imputed reference panels: (1) 2,201 low-coverage whole-genome sequences samples from the HUNT study and (2) HRC v1.1 with 1,023 HUNT WGS samples removed before merging. We excluded imputed variants with Rsq < 0.3 resulting in over 24.9 million well-imputed variants.

INTERMOUNTAIN

The HerediGene: Population Study is a collaboration between Intermountain Health and deCODE Genetics targeting the enrolment of 500,000 subjects. The purpose of this study is to link genomic and genetic data to medical records with the intent of improving predictive, prognostic, and diagnostic potential across diseases. Among its population based initiative, this study inherently includes subjects (>/= 18 years of age and United States resident) predisposed to, diagnosed with, and/or who have undergone a procedure associated with osteoarthritis.

JoCoOA

JoCo OA began in 1990 as a community-based prospective cohort of European-ancestry and African American men and women with and without osteoarthritis (OA). JoCo OA was designed to estimate the incidence and progression of knee and hip osteoarthritis among Europeans and African Americans, and the study has expanded to study the hand, lumbar spine, ankles and feet during successive follow-up visits (occurring approximately every 5 years). The sampling methods and study protocol have been previously reported. At baseline, 3187 European-ancestry and African American adults aged 45 years or older (35% men; 32% African American; mean age 61±10 years) were recruited from six townships in Johnston County, North Carolina, and completed a clinical examination visit during 1991–1997. An additional 1019 Johnston County residents 45+ years old were enrolled and attended a clinic visit during 2003–2004. The genotyped sample consists of those participants from baseline who returned for the first follow-up from 1999 to 2003, and the enrichment cohort of new participants in 2003–2004.

LifeLines

Lifelines participants were initially recruited through general practitioners in the three provinces in the northern part of the Netherlands: Groningen, Friesland and Drenthe1). General practitioners invited all their patients between 25 and 50 years old (on Terschelling 18+), resulting in ~81.500 participants at baseline.

These individuals were then asked to invite their family members (parents, partner, children, parents-in-law), resulting in an additional ~64.500 participants at baseline.

~21.500 interested individuals registered directly for baseline participation via the Lifelines website.

Mass General Brigham Biobank

The Mass General Brigham Biobank provides banked samples (plasma, serum, DNA,buffy coats, and, for some of our COVID-19 inpatient cohort, PBMCs) collected from patients who consented to broad-based research. These samples are linked to clinical data from the Electronic Health Record (EHR), quantitative data derived from medical images, and survey data on lifestyle, environment, and family history. The Biobank also provides genotype data and exome sequencing data.

Million Veteran Program

The Million Veteran Program (MVP), initiated by the Department of Veterans Affairs (VA), aims to collect bio-samples with consent from at least one million veterans. Presently, blood samples have been collected from over 800,000 enrolled participants. The size and diversity of the MVP cohort, as well as the availability of extensive VA electronic health records, make

it a promising resource for precision medicine. MVP is conducting array-based genotyping to provide a genome-wide scan of the entire cohort, in parallel with whole-genome sequencing, methylation, and other ‘omics assays. Here, we present the design and performance of the MVP 1.0 custom Axiom array, which was designed and developed as a single assay to be used across the multi-ethnic MVP cohort. A unified genetic quality-control analysis was developed and conducted on an initial tranche of 485,856 individuals, leading to a high-quality dataset of 459,777 unique individuals. 668,418 genetic markers passed quality control and showed high-quality genotypes not only on common variants but also on rare variants. We confirmed that, with non-European individuals making up nearly 30%, MVP's substantial ancestral diversity surpasses that of other large biobanks. We also demonstrated the quality of the MVP dataset by replicating established genetic associations with height in European Americans and African Americans ancestries. This current dataset has been made available to approved MVP researchers for genome-wide association studies and other downstream analyses. Further data releases will be available for analysis as recruitment at the VA continues and the cohort expands both in size and diversity.

The Netherlands Epidemiology of Obesity (NEO) study

The Netherlands Epidemiology of Obesity (NEO) study is a population-based, prospective cohort study, with an oversampling of overweight or obese individuals. In short, men and women between 45 and 65 years with a self-reported body mass index (BMI) ≥ 27 kg/m2 living in the greater area of Leiden (the Netherlands) were eligible to participate. In addition, all inhabitants aged between 45 and 65 years from one municipality (Leiderdorp) were invited to participate irrespective of their BMI, allowing for a reference BMI distribution comparable to the general Dutch population. The collection of data started in September 2008 and was completed at the end of September 2012. In total, 6,671 participants were included in the NEO study. In this study 5706 participants with genotype data were included. The Medical Ethical Committee of the Leiden University Medical Center (LUMC) approved the design of the study. All participants gave their written informed consent.

NHS: Nurses Health Study and Nurses Health Study II

The Nurses' Health Studies are among the largest prospective investigations into the risk factors for major chronic diseases in women (http://nurseshealthstudy.org/). The NHS is a prospective cohort study established in 1976. Blood samples were collected from a subset of participants in 1989-90. DNA was collected from cheek cells from another subset of participants in 2001-2004. The NHS II was established in 1989 to study a population younger than the original NHS cohort. Blood samples were collected on a subset of participants in 1996-1999. DNA from cheek cells was collected in 2006 from another subset of participants. Self-reported cases of total hip replacement from the NHS and NHS2 were analysed for the GO meta-analysis.

NIHRBioResource

The aim of the NIHR BioResource is to further health research within the UK. We help research organisations with volunteer recruitment, specifically by genotype and/or phenotype. The NIHR BioResource is a recallable resource of over 250,000 volunteers, with and without health conditions who have agreed to take part in health-related research.

Norwegian Arthroplasty Register (NAR)

Patients from the NAR were contacted and asked to participate after pre-defined protocol[327]. The cohort was after linked to HUNT.

The Osteoarthritis Initiative (OAI)

The Osteoarthritis Initiative (OAI) is a prospective longitudinal study designed to identify risk factors for the incidence and progression of symptomatic tibiofemoral knee osteoarthritis. A total of 4,796 men and women of any race/ethnicity aged 45 – 79 years were enrolled into pre-defined progression or incidence sub-cohorts[328]. Briefly, the progression sub-cohort included individuals who had symptomatic radiographic knee osteoarthritis while the

incidence sub-cohort included individuals who were considered to be at increased risk for developing symptomatic radiographic knee osteoarthritis based on weight, knee symptoms, history of knee injuries/surgeries, family history of knee replacement and hand osteoarthritis. Participants were recruited at four clinical sites: 1) Brown University (Providence, RI); 2) The Ohio State University (Columbus, OH); 3) University of Maryland and The Johns Hopkins University (Baltimore, MD); and 4) University of Pittsburgh (Pittsburgh, PA). Participants underwent bilateral posteroanterior fixed flexion knee radiographs at baseline and annually up to 96 months, and pelvic and hand radiographs at baseline and 48 months. Genome-wide genotyping is available for 3,302 European ancestry and 709 African ancestry participants[329,330].

QIMR [Over 50's (Aged) and Osteoarthritis (OA) Studies]

The QIMR cohort consists of twins recruited to two studies conducted at QIMR Berghofer Medical Research Institute: The Over 50's (Aged) and Osteoarthritis (OA) Studies. The Aged Study was conducted in 1993 as a multi-wave mailout to 4562 twins (2281 pairs) aged over 50 years old who were registered with the Australian Twin Registry. In total, 3116 individuals (1279 complete pairs and 558 singles) completed and returned the 16-page questionnaire form. Self-report osteoarthritis data was collected in the 'Bones and Joints' section in collaboration with the Australian Arthritis Foundation, and in consultation with Nick Bellamy and David Duffy. Participants were questioned about ever having experienced pain, swelling or stiffness in any joints; prior diagnosis of osteoarthritis or degenerative arthritis, rheumatoid arthritis, and other forms of arthritis or rheumatism; as well as prior bone fracture or joint injury. Self-report of pain and/or swelling in the joints of the hands, hips and knees were used as indicators of potential osteoarthritis, excluding those joints indicated to have sustained prior injury. Genome-wide data are available for 933 participants for osteoarthritis at any site and age of onset for osteoarthritis. The OA Study was designed to investigate how similar identical and non-identical twins are to each other and reach an estimate of the effect of genetic and environmental influences on osteoarthritis in the general population. The study conducted in 1994 where 441 twin pairs answered questions about prior diagnosis of osteoarthritis; presence of pain or swelling in target joints for osteoarthritis; and onset of joint pain/swelling not due to trauma after age 45. In addition, clinical examinations were performed by three Rheumatologists on 159 subjects, where approximately half were considered to be normal. Examinations of the twin's medical records and joint radiographs were conducted, where available. Osteoarthritis diagnosis involved comparing the self-report, clinical examination and radiological assessments. GWAS data is available for 1,073 participants (including 931 of the Aged Study participants) for osteoarthritis at any site, hip osteoarthritis, knee osteoarthritis, hand osteoarthritis and hip-knee osteoarthritis.

QIMR- PISA [Prospective Imaging Study of Ageing: Genes, Brain and Behaviour]

The scientific aims of the Prospective Imaging Study of Ageing: Genes, Brain and Behaviour (PISA Study) are to predict healthy and pathological ageing, quantify behavioural and psychological phenotypes, establish a cohort of patients with pre-clinical AD for future testing of interventions, and to inform novel diagnostic criteria for subtypes of disease (including stages before clinical symptoms occur). All participants in previous studies conducted by the Genetic Epidemiology group at QIMR Berghofer Medical Research Institute who were aged 40-70 years old and had genome-wide genotype data available (N=~15000) were invited to participate in the PISA Study. Self-report osteoarthritis data collected between 2018 and 2019 in the 'Pain and Health' section of the online questionnaire is available for 1,539 PISA participants (note: PISA data collection is ongoing). Osteoarthritis data is available for osteoarthritis at any site (454 cases and 1083 controls), as well as for hip osteoarthritis, knee osteoarthritis, hip-knee osteoarthritis, hand osteoarthritis, spine osteoarthritis, total joint replacement, total knee replacement and total hip replacement[331].

QIMR- GBP [Australian Genetics of Bipolar Disorder Study]

The Australian Genetics of Bipolar Disorder (GBP) Study is a nation-wide cohort of Australian adults aged 18-90 years old with lived experience of bipolar disorder (BD). The study aims to detect the relationships between genetic risk, symptom severity, and the lifetime prevalence of BD, treatment-response and medication side-effects, and patterns and costs of health care usage. Self-report osteoarthritis data (osteoarthritis at any site) was collected between 2018 and 2021 and is available for 3,092 GBP participants with genome-wide genotype data.

QIMR- AGDS [Australian Genetics of Depression Study]

The Australian Genetics of Depression Study (AGDS) was established to recruit a large sample of Australian participants aged 18-90 years old who have lived experienced of depression. Self-report osteoarthritis and genotype data is available for osteoarthritis at any site (1733 cases, 5240 controls), and for hip osteoarthritis, knee osteoarthritis, hip-knee osteoarthritis, hand osteoarthritis, spine osteoarthritis, total joint replacement, total knee replacement and total hip replacement.

RAAK study

The RAAK study is an ongoing collection of blood, articular cartilage and subchondral bone samples of thoroughly phenotyped osteoarthritis patients undergoing joint replacement surgery at Leiden University Medical Center and the Alrijne Hospital[332]. Materials are collected to allow, histology, isolation of RNA and DNA as well as cells (human bone marrow derived stem cells, primary chondrocytes) to employ in-vitro models. High dimensional molecular datasets (transcriptome (mRNA, miRNA and lncRNA) and methylome) were generated from preserved and lesioned OA cartilage and subchondral bone samples[110,300,333,334].

RIKEN Study

The Japanese cohort of knee osteoarthritis GWAS (disease cohort) consists of 900 cases and 3,400 controls. The cases were all symptomatic osteoarthritis. They were diagnosed and recruited by expert orthopedic surgeons based on clinical and radiographic examination. All had clinical records for osteoarthritis and radiographs (standing knee A-P). The controls were obtained from Biobank Japan. The genotyping was done by using Illumina HumanHap550v3 Genotyping BeadChip. After excluding cases with call rate of <0.98, we applied SNP QC (call rate of ≥ 0.99 in both cases and controls and P value of Hardy-Weinberg equilibrium test of $\geq 1.0\times10^{-6}$ in controls). Finally, 459,393 SNPs on autosomal chromosomes passed the QC filters[335].

The Rotterdam Study

The Rotterdam study is a large longitudinal population-based cohort study designed to study the risk factors for all major diseases of the elderly. The study started in 1991, has grown to up to 15000 individuals and has detailed phenotyping for cardiovascular, neurodegenerative, endocrine and locomoter diseases and more. For osteoarthritis, longitudinal X-rays on multiple joints and knee MRI's are available (and scored), as well as information on joint pain. GWAS data is available for all individuals of the Rotterdam Study, as well as additional molecular layers (such as RNA, methylation, microbiome). Genomic studies in the Rotterdam Study are led by the Genetic Laboratory, Department of Internal Medicine of the ErasmusMC in Rotterdam (http://www.epib.nl/research/ergo.htm, http://www.glimdna.org).

Shimane CoHRE Study

Shimane CoHRE Study is a part of the cohort study conducted by the Center for Community-based Healthcare Research and Education in Shimane University. The study is an ongoing health examination for the community-dwelling people in Shimane prefecture, Japan.

SHIP START and SHIP TREND

SHIP (Study of Health in Pomerania): The Study of Health In Pomerania (SHIP) is a prospective longitudinal population-based cohort study in Mecklenburg-Western Pomerania

assessing the prevalence and incidence of common diseases and their risk factors[336,337]. SHIP encompasses the two independent cohorts SHIP-START and SHIP-TREND. Participants aged 20 to 79 with German citizenship and principal residency in the study area were recruited from a random sample of residents living in the three local cities, 12 towns as well as 17 randomly selected smaller towns. Individuals were randomly selected stratified by age and sex in proportion to population size of the city, town or small towns, respectively. A total of 4,308 participants were recruited between 1997 and 2001 in the SHIP-START cohort. Between 2008 and 2012 a total of 4,420 participants were recruited in the SHIP-TREND cohort. Individuals were invited to the SHIP study centre for a computer-assisted personal interviews and extensive physical examinations. The study protocol was approved by the medical ethics committee of the University of Greifswald. Oral and written informed consent was obtained from each of the study participants.

SIMPLER-SIMPLER, SIMPLER-COSMC and SIMPLER-SMCC

The Swedish Infrastructure for Medical Population-based Life-course and Environmental Research - SIMPLER - comprises two population-based longitudinal cohorts: the Swedish Mammography Cohort and the Cohort of Swedish Men. Almost 110 000 participants (born from 1914 through 1952) are included in the two cohorts with health questionnaires, including diet and lifestyle on repeated occasions. The women entered the cohort in 1987-1990 and the men in 1997. Complete follow-up of diseases is accomplished by use of the individual personal registration number provided to all Swedish citizens and different national registers. Participants in two sub-cohorts, SMCC and COSMC, have also visited research clinics in Uppsala and Västerås for biometric measurements and for sampling various types of fasting biological samples. DNA was extracted from whole blood samples in the sub-cohorts and saliva samples for the rest of SIMPLER's participants.

Tohoku Medical Megabank Organisation

The Tohoku Medical Megabank (TMM) Project[338] was launched for the purpose of reconstruction from the Great East Japan Earthquake and establishment of personalized healthcare and medicine. In the TMM Community-Based Cohort Study[339], which is a population-based cohort study as a part of the TMM Project, a total of more than 80,000 residents aged 20 years or older living in Iwate and Miyagi Prefectures, located on the Pacific side of Tohoku (northeastern) region of Honshu (the main island of Japan), were recruited from May 2013 to March 2016. In the study, only the participants in Miyagi Prefecture were used.

TwinsUK

TwinsUK is the UK's largest adult twin registry and the most clinically detailed in the world. Professor Tim Spector from King's College London set up the cohort in 1992 to investigate the incidence of osteoporosis and other rheumatologic diseases in several hundred monozygotic (identical) twins. We now have almost 14000 identical and non-identical twins from across the UK, with ages between sixteen and one hundred and our research has expanded to include multiple diseases and conditions. TwinsUK aims to investigate the genetic and environmental basis of a range of complex diseases and conditions. Current research includes the genetics of metabolic syndrome, cardiovascular disease, the musculoskeletal system, ageing, sight as well as how the microbiome affects human health. The TwinsUK cohort is now probably the most genotyped and phenotyped cohort in the world. TwinsUK data have enabled multiple collaborations with research groups worldwide and the publication of research papers. http://twinsuk.ac.uk/. Details on osteoarthritis definition as in [10] and GWAS, QC imputation as described in [340].

UK Biobank

The UK Biobank study is a large population-based prospective study of >500,000 participants with ages ranging 40–69 years. All participants signed consent to participate in UK Biobank and UK Biobank's scientific protocol and operational procedures were reviewed and

approved by the North West Research Ethics Committee (REC reference number 06/MRE08/65), North West Multicentre Research Ethics Committee (REC reference 11/NW/0382), the National Information Governance Board for Health and Social Care and the Community Health Index Advisory Group. In total, 503,325 participants who registered in the National Health Service were recruited out of 9.2 million mailed invitations between 2006 and 2010 in 22 assessment centres throughout the UK[341]. Most participants visited the centre once, but some individuals visited the centre at up to three times. Baseline data were collected using electronic signed consent, a self-completed touch-screen questionnaire, a brief computer-assisted interview, physical and functional measures, and collection of biological samples and genetic data. The UK Biobank genetic data contains genotypes for 488,377 participants. All detailed genotyping, quality control, and imputation procedures are described at the UK Biobank website (http://biobank.ctsu.ox.ac.uk). Briefly, 50,000 samples were genotyped using the UKBiLEVE array and the remaining samples were genotyped using the UK Biobank Axiom array (Affymetrix) for ~800,000 SNPs. Population structure was captured by principal component analysis on ~500,000 UK Biobank samples using ~100,000 SNPs. After sample and SNP quality control (QC) of the directly-typed genotypes, resulting in 670,739 autosomal markers in 487,442 individuals, data were prephased using SHAPEIT3[342] and imputed using the IMPUTE4 program (https://jmarchini.org/software/). Both analyses were carried out centrally[343] and the full dataset consisted of approximately 96 million variants in 487,411 individuals. https://www.ukbiobank.ac.uk/
This work was based on the third UK Biobank release, which includes the full set of genotypes imputed on the Haplotype Reference Consortium[322] and the merged UK10K and 1000 Genomes phase 3 reference panels1000 Genomes Consortium[344]. Access to UK Biobank genetic and phenotypic data was given through the UK Biobank Resource under application number 9979.

UK Household Longitudinal Study (UKHLS)

UKHLS also known as Understanding Society, is a longitudinal panel survey of 40,000 UK households (England, Scotland, Wales and Northern Ireland) representative of the UK population. Participants are surveyed annually since 2009 and contribute information relating to their socioeconomic circumstances, attitudes, and behaviours via a computer assisted interview. The study includes phenotypical data for a representative sample of participants for a wide range of social and economic indicators as well as a biological sample collection encompassing biometric, physiological, biochemical, and haematological measurements and self-reported medical history and medication use.
https://www.understandingsociety.ac.uk/

Consortia Information

The arcOGEN Consortium

John Loughlin, Nigel Arden, Fraser Birrell, Andrew Carr, Panos Deloukas, Michael Doherty, Andrew W. McCaskie, William E. R. Ollier, Ashok Rai, Stuart H. Ralston, Tim D. Spector, Ana M. Valdes, Gillian A. Wallis, J. Mark Wilkinson, Eleftheria Zeggini

The ARGO Consortium

ARGO-Athens: Eleni Zengini, George Alexiadis, Evangelos Tyrpenou, Athanasios Koukakis, Dimitrios Chytas, Dimitrios Stergios Evangelopoulos, Chronopoulos Efstathios, Spiros Pneumaticos, Vasileios S. Nikolaou, J. Mark Wilkinson, George C. Babis, Konstantinos Hatzikotoulas, Eleftheria Zeggini. ARGO-Larissa: Konstantinos Malizos, Lydia Anastasopoulou, Aspasia Tsezou, Eleni Zengini, J. Mark Wilkinson, Konstantinos Hatzikotoulas, Eleftheria Zeggini

Regeneron Genetics Center

Gonçalo Abecasis, Aris Baras, Aris Economides, Adolfo Ferrando, Michael Cantor, Giovanni Coppola, Andrew Deubler, Katia Karalis, Luca Lotta, John Overton, Jeffrey Reid, Katherine

Siminovitch, Lyndon Mitnaul, Alan Shuldiner, Christina Beechert, Caitlin Forsythe, Erin Brian, Zhenhua Gu, Michael Lattari, Alexander Lopez, John Overton, Maria Sotiropoulos Padilla, Manasi Pradhan, Kia Manoochehri, Ricardo Schiavo, Raymond Reynoso, Kristy Guevara, Laura Cremona, Chenggu Wang, Hang Du, Sarah Wolf, Amelia Averitt, Nilanjana Banerjee, Michael Cantor, Dadong Li, Sameer Malhotra, Deepika Sharma, Justin Mower, Jay Sundaram, Aaron Zhang, Sean Yu, Mudasar Sarwar, Jeffrey Staples, Xiaodong Bai, Lance Zhang, Sean O'Keeffe, Andrew Bunyea, Lukas Habegger, Suganthi Balasubramanian, Suying Bao, Boris Boutkov, Gisu Eom, Lukas Habegger, Alicia Hawes, Olga Krasheninina, Rouel Lanche, Adam Mansfield, Evan Edelstein, Sujit Gokhale, Alexander Gorovits, Evan Maxwell, Ju Guan, George Mitra, Janice Clauer, Mona Nafde, Vrushali Mahajan, Razvan Panea, Koteswararao Makkena, Krishna Pawan Punuru, Benjamin Sultan, Sanjay Sreeram, Tommy Polanco, Ayesha Rasool, Jeffrey Reid, William Salerno, Kathie Sun, Joshua Backman, Anthony Marcketta, Bin Ye, Lauren Gurski, Nan Lin, Gonçalo Abecasis, Jonathan Marchini, Manuel Allen Revez Ferreira, Yuxin Zou, Jack Kosmicki, Jonathan Ross, Joelle Mbatchou, Andrey Ziyatdinov, Kyoko Watanabe, Eli Stahl, Akropravo Ghosh, Lei Chen, Rujin Wang, Adam Locke, Carlo Sidore, Arden Moscati, Lee Dobbyn, Eric Jorgenson, Blair Zhang, Christopher Gillies, Michael Kessler, Maria Suciu, Timothy Thornton, Priyanka Nakka, Sheila Gaynor, Tyler Joseph , Benjamin Geraghty, Anita Pandit, Joseph Herman, Sam Choi, Peter VandeHaar, Liron Ganel, Kuan-Han Wu, Aditeya Pandey, Kathy Burch, Adrian Campos, Scott Vrieze, Sailaja Vedantam, Charles Paulding, Amy Damask, Ariane Ayer, Aysegul Guvenek, George Hindy, Giovanni Coppola, Jan Freudenberg, Jonas Bovijn, Katherine Siminovitch, Luca Lotta, Manav Kapoor, Mary Haas, Moeen Riaz, Niek Verweij, Olukayode Sosina, Parsa Akbari, Priyanka Nakka, Sahar Gelfman, Sujit Gokhale, Tanima De, Veera Rajagopal, Alan Shuldiner, Bin Ye, Gannie Tzoneva, Jin He, Adolfo Ferrando, Silvia Alvarez, Kayode Sosina, Neel Parikshak, Jacqueline Otto, Anna Alkelai, Vijay Kumar, Peter Dombos, Amit Joshi, Sarah Graham, Luanluan Sun, Antoine Baldassari, Jessie Brown, Cristen Willer, Arthur Gilly, Hossein Khiabanian, Brian Hobbs, Billy Palmer, Juan Rodriguez-Flores, Esteban Chen, Jaimee Hernandez, Marcus Jones, Michelle LeBlanc, Jason Mighty, Nirupama Nishtala, Nadia Rana, Jennifer Rico-Varela, Randi Schwartz, Thomas Coleman, Alison Fenney, Jody Hankins, Ruan Cox, Samuel Hart

Estonian Biobank Research Team

Andres Metspalu, Lili Milani, Tõnu Esko, Reedik Mägi, Mari Nelis, Georgi Hudjashov

DBDS Genomic Consortium

Karina Banasik, Jakob Bay, Jens Kjærgaard Boldsen, Thorsten Brodersen, Søren Brunak, Kristoffer Burgdorf, Mona Ameri Chalmer, Maria Didriksen, Khoa Manh Dinh, Joseph Dowsett, Christian Erikstrup, Bjarke Feenstra, Frank Geller, Daniel Gudbjartsson, Thomas Folkmann Hansen, Lotte Hindhede, Henrik Hjalgrim, Rikke Louise Jacobsen, Gregor Jemec, Bitten Aagaard Jensen, Katrine Kaspersen, Bertram Dalskov Kjerulff,Lisette Kogelman, Margit Anita Hørup Larsen, Ioannis Louloudis, Agnete Lundgaard, Susan Mikkelsen, Christina Mikkelsen, Ioanna Nissen, Mette Nyegaard, Sisse Rye Ostrowski, Ole Birger Pedersen, Alexander Pil Henriksen, Palle Duun Rohde, Klaus Rostgaard, Michael Schwinn, Kari Stefansson, Hreinn Stefánsson, Erik Sørensen, Unnur Þorsteinsdóttir, Lise Wegner Thørner, Mie Topholm Bruun, Henrik Ullum, Thomas Werge, David Westergaard

Genes & Health Research Team

Current Genes & Health Research Team: Eamonn Maher, Shabana Chaudhary, Joseph Gafton, Karen A Hunt, Shapna Hussain, Kamrul Islam, Hilary Martin, Mohammed Bodrul Mazid, Elizabeth Owor, Jessry Russell, Nishat Safa, John Solly, Marie Spreckley, David A Van Heel, Jan Whalley, Ishevanhu Zengeya, Emily Mantle, Shaheen Akhtar, Samina Ashraf, Dan Mason, John Wright, Daniel MacArthur, Michael Simpson, Richard C Trembath, Gerome Breen, Raymond Chung, Sang Hyuck Lee, Omar Asgar, Joanne Harvey, Karen Tricker, Caroline Winckley, Hanifa Khatun, Amna Asif, Claudia Langenberg, Grainne Colligan, Ceri Durham, Bill Newman, Ahsan Khan, Teng Heng, Matt Hurles, Vivek Iyer, Georgios Kalantzis, Vladimir

Ovchinnikov, Iaroslav Popov, Klaudia Walter, Panos Deloukas, David Collier, Ana Angel, Saeed Bidi, Fabiola Eto, Sarah Finer, Chris Griffiths, Sam Hodgson, Benjamin M Jacobs, Rohini Mathur, Caroline Morton, Asma Qureshi, Stuart Rison, Annum Salman, Miriam Samuel, Moneeza K Siddiqui, Daniel Stow, Sabina Yasmin, Julia Zöllner, Sheik Dowlut.

HUNT All-In Pain

Amy E Martinsen[1,2,3], Anne Heidi Skogholt[3], Ben M Brumpton[3], Bendik S Winsvold[1,3,4], Egil A Fors[5], Elisabeth Gjefsen[1], Espen S Kristoffersen[6,7,1], Ingrid Heuch[1], Ingunn Mundal[8], John-Anker Zwart[1,2,3], Jonas B Nielsen[3,9], Kjersti Storheim[10,11], Knut Hagen[12], Kristian Bernhard Nilsen[4,12], Kristian Hveem[3,13,14], Lars G Fritsche[15], Laurent F Thomas[3,16,17,18], Linda M Pedersen[1], Maiken E Gabrielsen[3], Sigrid Børte[2,3,10], Synne Ø Stensland[10,19], Wei Zhou[20,21]

1 Department of Research and Innovation, Division of Clinical Neuroscience, Oslo University Hospital, Oslo, Norway
2 Institute of Clinical Medicine, Faculty of Medicine, University of Oslo, Oslo, Norway
3 K. G. Jebsen Center for Genetic Epidemiology, Department of Public Health and Nursing, Faculty of Medicine and Health Sciences, Norwegian University of Science and Technology (NTNU), Trondheim, Norway
4 Department of Neurology, Oslo University Hospital, Oslo, Norway
5 Department of Public Health and Nursing, Faculty of Medicine and Health Sciences, Norwegian University of Science and Technology (NTNU), Trondheim, Norway
6 Department of General Practice, University of Oslo, Oslo, Norway
7 Department of Neurology, Akershus University Hospital, Lørenskog, Norway
8 Department of Health Science, Molde University College, Molde, Norway
9 Department of Internal Medicine, Division of Cardiovascular Medicine, University of Michigan, Ann Arbor, MI, 48109, USA
10 Research and Communication Unit for Musculoskeletal Health (FORMI), Department of Research and Innovation, Division of Clinical Neuroscience, Oslo University Hospital, Oslo, Norway
11 Department of physiotherapy, Faculty of Health Sciences, Oslo Metropolitan University, Oslo, Norway
12 Department of Neuromedicine and Movement Science, Faculty of Medicine and Health Sciences, Norwegian University of Science and Technology (NTNU), Trondheim, Norway
13 HUNT Research Center, Department of Public Health and Nursing, Faculty of Medicine and Health Sciences, Norwegian University of Science and Technology (NTNU), Trondheim, Norway
14 Department of Research, Innovation and Education, St. Olavs Hospital, Trondheim University Hospital, Trondheim, Norway
15 Center for Statistical Genetics, Department of Biostatistics, University of Michigan, Ann Arbor, MI, 48109, USA
16 Department of Clinical and Molecular Medicine, Norwegian University of Science and Technology (NTNU), Trondheim, Norway
17 BioCore - Bioinformatics Core Facility, Norwegian University of Science and Technology (NTNU), Trondheim, Norway
18 Clinic of Laboratory Medicine, St. Olavs Hospital, Trondheim University Hospital, Trondheim, Norway
19 Norwegian Centre for Violence and Traumatic Stress Studies, Oslo, Norway
20 Department of Computational Medicine and Bioinformatics, University of Michigan, Ann Arbor, MI, 48109, USA
21 Analytic and Translational Genetics Unit, Massachusetts General Hospital, Boston, MA, USA

FinnGen

FinnGen is public-private research project. FinnGen Consorotium Partners are: AbbVie Inc., AstraZeneca UK Ltd, Biogen MA Inc., Bristol Myers Squibb (and Celgene Corporation & Celgene International II Sàrl), Genentech Inc., Merck Sharp & Dohme LCC, Pfizer Inc., GlaxoSmithKline Intellectual Property Development Ltd., Sanofi US Services Inc., Maze Therapeutics Inc., Janssen Biotech Inc, Novartis AG, and Boehringer Ingelheim International GmbH, Auria Biobank, University of Turku, THL Biobank, Helsinki Biobank, Biobank Borealis of Northern Finland, Finnish Clinical Biobank Tampere, Biobank of Eastern Finland, Central FinnGen is public-private research project. FinnGen Consorotium Partners are 13 international pharmaceutical companies, 9 Finnish biobanks and their host organizations, and the Finnish Biobank Cooperative (FINBB).
The pharmaceutical company partners are AbbVie Inc., AstraZeneca UK Ltd, Biogen MA Inc., Bristol Myers Squibb (and Celgene Corporation & Celgene International II Sàrl), Genentech

Inc., Merck Sharp & Dohme LCC, Pfizer Inc., GlaxoSmithKline Intellectual Property Development Ltd., Sanofi US Services Inc., Maze Therapeutics Inc., Janssen Biotech Inc, Novartis AG, and Boehringer Ingelheim International GmbH.
The Finnish biobanks and their host organizations are Auria Biobank, Wellbeing Services County of Southwest Finland, University of Turku, THL Biobank, Finnish Institute for Health and Welfare, Helsinki Biobank, Hospital District of Helsinki and Uusimaa (HUS Group), University of Helsinki, Biobank Borealis of Northern Finland, The Wellbeing Services County of North Ostrobothnia, University of Oulu, Finnish Clinical Biobank Tampere, Wellbeing Services County of Pirkanmaa, Tampere University Foundation sr, Biobank of Eastern Finland, Wellbeing Services County of North Savo, University of Eastern Finland, Central Finland Biobank, The Wellbeing Services County of Central Finland, University of Jyväskylä, Finnish Hematology Registry and Clinical Biobank, Finnish Red Cross Blood Service Biobank, The Finnish Red Cross Blood Services.

Million Veteran Program

MVP Executive Committee
- Co-Chair: J. Michael Gaziano, M.D., M.P.H.
VA Boston Healthcare System, 150 S. Huntington Avenue, Boston, MA 02130
- Co-Chair: Sumitra Muralidhar, Ph.D.
US Department of Veterans Affairs, 810 Vermont Avenue NW, Washington, DC 20420
- Rachel Ramoni, D.M.D., Sc.D., Chief VA Research and Development Officer
US Department of Veterans Affairs, 810 Vermont Avenue NW, Washington, DC 20420
- Jean Beckham, Ph.D.
Durham VA Medical Center, 508 Fulton Street, Durham, NC 27705
- Kyong-Mi Chang, M.D
Philadelphia VA Medical Center, 3900 Woodland Avenue, Philadelphia, PA 19104
- Christopher J. O'Donnell, M.D., M.P.H.
VA Boston Healthcare System, 150 S. Huntington Avenue, Boston, MA 02130
- Philip S. Tsao, Ph.D.
VA Palo Alto Health Care System, 3801 Miranda Avenue, Palo Alto, CA 94304
- James Breeling, M.D., Ex-Officio
US Department of Veterans Affairs, 810 Vermont Avenue NW, Washington, DC 20420
- Grant Huang, Ph.D., Ex-Officio
US Department of Veterans Affairs, 810 Vermont Avenue NW, Washington, DC 20420
- Juan P. Casas, M.D., Ph.D., Ex-Officio
VA Boston Healthcare System, 150 S. Huntington Avenue, Boston, MA 02130

MVP Program Office
- Sumitra Muralidhar, Ph.D.
US Department of Veterans Affairs, 810 Vermont Avenue NW, Washington, DC 20420
- Jennifer Moser, Ph.D.
US Department of Veterans Affairs, 810 Vermont Avenue NW, Washington, DC 20420

MVP Recruitment/Enrollment
- Recruitment/Enrollment Director/Deputy Director, Boston – Stacey B. Whitbourne, Ph.D.; Jessica V. Brewer, M.P.H.
VA Boston Healthcare System, 150 S. Huntington Avenue, Boston, MA 02130
- MVP Coordinating Centers
o Clinical Epidemiology Research Center (CERC), West Haven – Mihaela Aslan, Ph.D.
West Haven VA Medical Center, 950 Campbell Avenue, West Haven, CT 06516
o Cooperative Studies Program Clinical Research Pharmacy Coordinating Center, Albuquerque – Todd Connor, Pharm.D.; Dean P. Argyres, B.S., M.S.

New Mexico VA Health Care System, 1501 San Pedro Drive SE, Albuquerque, NM 87108
o Genomics Coordinating Center, Palo Alto – Philip S. Tsao, Ph.D.
VA Palo Alto Health Care System, 3801 Miranda Avenue, Palo Alto, CA 94304
o MVP Boston Coordinating Center, Boston-J. Michael Gaziano, M.D., M.P.H.
VA Boston Healthcare System, 150 S. Huntington Avenue, Boston, MA 02130
o MVP Information Center, Canandaigua – Brady Stephens, M.S.
Canandaigua VA Medical Center, 400 Fort Hill Avenue, Canandaigua, NY 14424
- VA Central Biorepository, Boston – Mary T. Brophy M.D., M.P.H.; Donald E. Humphries, Ph.D.; Luis E. Selva, Ph.D.
VA Boston Healthcare System, 150 S. Huntington Avenue, Boston, MA 02130
- MVP Informatics, Boston – Nhan Do, M.D.; Shahpoor (Alex) Shayan, M.S.
VA Boston Healthcare System, 150 S. Huntington Avenue, Boston, MA 02130
- MVP Data Operations/Analytics, Boston – Kelly Cho, M.P.H., Ph.D.
VA Boston Healthcare System, 150 S. Huntington Avenue, Boston, MA 02130
- Director of Regulatory Affairs – Lori Churby, B.S.
VA Palo Alto Health Care System, 3801 Miranda Avenue, Palo Alto, CA 94304
MVP Science
- Science Operations – Christopher J. O'Donnell, M.D., M.P.H.
VA Boston Healthcare System, 150 S. Huntington Avenue, Boston, MA 02130
- Genomics Core – Christopher J. O'Donnell, M.D., M.P.H.; Saiju Pyarajan Ph.D.
VA Boston Healthcare System, 150 S. Huntington Avenue, Boston, MA 02130
Philip S. Tsao, Ph.D.
VA Palo Alto Health Care System, 3801 Miranda Avenue, Palo Alto, CA 94304
- Data Core – Kelly Cho, M.P.H, Ph.D.
VA Boston Healthcare System, 150 S. Huntington Avenue, Boston, MA 02130
- VA Informatics and Computing Infrastructure (VINCI) – Scott L. DuVall, Ph.D.
VA Salt Lake City Health Care System, 500 Foothill Drive, Salt Lake City, UT 84148
- Data and Computational Sciences – Saiju Pyarajan, Ph.D.
VA Boston Healthcare System, 150 S. Huntington Avenue, Boston, MA 02130
- Statistical Genetics – Elizabeth Hauser, Ph.D.
Durham VA Medical Center, 508 Fulton Street, Durham, NC 27705
Yan Sun, Ph.D.
Atlanta VA Medical Center, 1670 Clairmont Road, Decatur, GA 30033
Hongyu Zhao, Ph.D.
West Haven VA Medical Center, 950 Campbell Avenue, West Haven, CT 06516

Current MVP Local Site Investigators
- Atlanta VA Medical Center (Peter Wilson, M.D.)
1670 Clairmont Road, Decatur, GA 30033
- Bay Pines VA Healthcare System (Rachel McArdle, Ph.D.)
10,000 Bay Pines Blvd Bay Pines, FL 33744
- Birmingham VA Medical Center (Louis Dellitalia, M.D.)
700 S. 19th Street, Birmingham AL 35233
- Central Western Massachusetts Healthcare System (Kristin Mattocks, Ph.D., M.P.H.)
421 North Main Street, Leeds, MA 01053
- Cincinnati VA Medical Center (John Harley, M.D., Ph.D.)
3200 Vine Street, Cincinnati, OH 45220
- Clement J. Zablocki VA Medical Center (Jeffrey Whittle, M.D., M.P.H.)
5000 West National Avenue, Milwaukee, WI 53295
- VA Northeast Ohio Healthcare System (Frank Jacono, M.D.)
10701 East Boulevard, Cleveland, OH 44106

- Durham VA Medical Center (Jean Beckham, Ph.D.)
508 Fulton Street, Durham, NC 27705
- Edith Nourse Rogers Memorial Veterans Hospital (John Wells., Ph.D.)
200 Springs Road, Bedford, MA 01730
- Edward Hines, Jr. VA Medical Center (Salvador Gutierrez, M.D.)
5000 South 5th Avenue, Hines, IL 60141
- Veterans Health Care System of the Ozarks (Gretchen Gibson, D.D.S., M.P.H.)
1100 North College Avenue, Fayetteville, AR 72703
- Fargo VA Health Care System (Kimberly Hammer, Ph.D.)
2101 N. Elm, Fargo, ND 58102
- VA Health Care Upstate New York (Laurence Kaminsky, Ph.D.)
113 Holland Avenue, Albany, NY 12208
- New Mexico VA Health Care System (Gerardo Villareal, M.D.)
1501 San Pedro Drive, S.E. Albuquerque, NM 87108
- VA Boston Healthcare System (Scott Kinlay, M.B.B.S., Ph.D.)
150 S. Huntington Avenue, Boston, MA 02130
- VA Western New York Healthcare System (Junzhe Xu, M.D.)
3495 Bailey Avenue, Buffalo, NY 14215-1199
- Ralph H. Johnson VA Medical Center (Mark Hamner, M.D.)
109 Bee Street, Mental Health Research, Charleston, SC 29401
- Columbia VA Health Care System (Roy Mathew, M.D.)
6439 Garners Ferry Road, Columbia, SC 29209
- VA North Texas Health Care System (Sujata Bhushan, M.D.)
4500 S. Lancaster Road, Dallas, TX 75216
- Hampton VA Medical Center (Pran Iruvanti, D.O., Ph.D.)
100 Emancipation Drive, Hampton, VA 23667
- Richmond VA Medical Center (Michael Godschalk, M.D.)
1201 Broad Rock Blvd., Richmond, VA 23249
- Iowa City VA Health Care System (Zuhair Ballas, M.D.)
601 Highway 6 West, Iowa City, IA 52246-2208
- Eastern Oklahoma VA Health Care System (Douglas Ivins, M.D.)
1011 Honor Heights Drive, Muskogee, OK 74401
- James A. Haley Veterans' Hospital (Stephen Mastorides, M.D.)
13000 Bruce B. Downs Blvd, Tampa, FL 33612
- James H. Quillen VA Medical Center (Jonathan Moorman, M.D., Ph.D.)
Corner of Lamont & Veterans Way, Mountain Home, TN 37684
- John D. Dingell VA Medical Center (Saib Gappy, M.D.)
4646 John R Street, Detroit, MI 48201
- Louisville VA Medical Center (Jon Klein, M.D., Ph.D.)
800 Zorn Avenue, Louisville, KY 40206
- Manchester VA Medical Center (Nora Ratcliffe, M.D.)
718 Smyth Road, Manchester, NH 03104
- Miami VA Health Care System (Hermes Florez, M.D., Ph.D.)
1201 NW 16th Street, 11 GRC, Miami FL 33125
- Michael E. DeBakey VA Medical Center (Olaoluwa Okusaga, M.D.)
2002 Holcombe Blvd, Houston, TX 77030
- Minneapolis VA Health Care System (Maureen Murdoch, M.D., M.P.H.)
One Veterans Drive, Minneapolis, MN 55417
- N. FL/S. GA Veterans Health System (Peruvemba Sriram, M.D.)
1601 SW Archer Road, Gainesville, FL 32608
- Northport VA Medical Center (Shing Shing Yeh, Ph.D., M.D.)

79 Middleville Road, Northport, NY 11768
- Overton Brooks VA Medical Center (Neeraj Tandon, M.D.)
510 East Stoner Ave, Shreveport, LA 71101
- Philadelphia VA Medical Center (Darshana Jhala, M.D.)
3900 Woodland Avenue, Philadelphia, PA 19104
- Phoenix VA Health Care System (Samuel Aguayo, M.D.)
650 E. Indian School Road, Phoenix, AZ 85012
- Portland VA Medical Center (David Cohen, M.D.)
3710 SW U.S. Veterans Hospital Road, Portland, OR 97239
- Providence VA Medical Center (Satish Sharma, M.D.)
830 Chalkstone Avenue, Providence, RI 02908
- Richard Roudebush VA Medical Center (Suthat Liangpunsakul, M.D., M.P.H.)
1481 West 10th Street, Indianapolis, IN 46202
- Salem VA Medical Center (Kris Ann Oursler, M.D.)
1970 Roanoke Blvd, Salem, VA 24153
- San Francisco VA Health Care System (Mary Whooley, M.D.)
4150 Clement Street, San Francisco, CA 94121
- South Texas Veterans Health Care System (Sunil Ahuja, M.D.)
7400 Merton Minter Boulevard, San Antonio, TX 78229
- Southeast Louisiana Veterans Health Care System (Joseph Constans, Ph.D.)
2400 Canal Street, New Orleans, LA 70119
- Southern Arizona VA Health Care System (Paul Meyer, M.D., Ph.D.)
3601 S 6th Avenue, Tucson, AZ 85723
- Sioux Falls VA Health Care System (Jennifer Greco, M.D.)
2501 W 22nd Street, Sioux Falls, SD 57105
- St. Louis VA Health Care System (Michael Rauchman, M.D.)
915 North Grand Blvd, St. Louis, MO 63106
- Syracuse VA Medical Center (Richard Servatius, Ph.D.)
800 Irving Avenue, Syracuse, NY 13210
- VA Eastern Kansas Health Care System (Melinda Gaddy, Ph.D.)
4101 S 4th Street Trafficway, Leavenworth, KS 66048
- VA Greater Los Angeles Health Care System (Agnes Wallbom, M.D., M.S.)
11301 Wilshire Blvd, Los Angeles, CA 90073
- VA Long Beach Healthcare System (Timothy Morgan, M.D.)
5901 East 7th Street Long Beach, CA 90822
- VA Maine Healthcare System (Todd Stapley, D.O.)
1 VA Center, Augusta, ME 04330
- VA New York Harbor Healthcare System (Scott Sherman, M.D., M.P.H.)
423 East 23rd Street, New York, NY 10010
- VA Pacific Islands Health Care System (George Ross, M.D.)
459 Patterson Rd, Honolulu, HI 96819
- VA Palo Alto Health Care System (Philip Tsao, Ph.D.)
3801 Miranda Avenue, Palo Alto, CA 94304-1290
- VA Pittsburgh Health Care System (Patrick Strollo, Jr., M.D.)
University Drive, Pittsburgh, PA 15240
- VA Puget Sound Health Care System (Edward Boyko, M.D.)
1660 S. Columbian Way, Seattle, WA 98108-1597
- VA Salt Lake City Health Care System (Laurence Meyer, M.D., Ph.D.)
500 Foothill Drive, Salt Lake City, UT 84148
- VA San Diego Healthcare System (Samir Gupta, M.D., M.S.C.S.)
3350 La Jolla Village Drive, San Diego, CA 92161

- VA Sierra Nevada Health Care System (Mostaqul Huq, Pharm.D., Ph.D.)
975 Kirman Avenue, Reno, NV 89502
- VA Southern Nevada Healthcare System (Joseph Fayad, M.D.)
6900 North Pecos Road, North Las Vegas, NV 89086
- VA Tennessee Valley Healthcare System (Adriana Hung, M.D., M.P.H.)
1310 24th Avenue, South Nashville, TN 37212
- Washington DC VA Medical Center (Jack Lichy, M.D., Ph.D.)
50 Irving St, Washington, D. C. 20422
- W.G. (Bill) Hefner VA Medical Center (Robin Hurley, M.D.)
1601 Brenner Ave, Salisbury, NC 28144
- White River Junction VA Medical Center (Brooks Robey, M.D.)
163 Veterans Drive, White River Junction, VT 05009
- William S. Middleton Memorial Veterans Hospital (Robert Striker, M.D., Ph.D.)
2500 Overlook Terrace, Madison, WI 53705

Acknowledgements and Funding

arcOGEN

arcOGEN was funded by a special-purpose grant from Arthritis Research UK (grant 18030). The UKHLS was funded by grants from the Economic and Social Research Council (ES/H029745/1) and the Wellcome Trust (WT098051).

BioMe BioBank Program

The Mount Sinai biobank was established in 2007 with a donation from The Andrea and Charles Bronfman Philanthropies.

Bunkyo Health Study

Bunkyo Health Study is supported by the Strategic Research Foundation at Private Universities (S1411006) and Japan Society for the Promotion of Science (JSPS) KAKENHI (18H03184) from the Ministry of Education, Culture, Sports, Science and Technology of Japan, the Mizuno Sports Promotion Foundation, the Mitsui Life Social Welfare Foundation

China Kadoorie Biobank

The most important acknowledgement is to the participants in the study and the members of the survey teams in each of the 10 regional centres, and to the project development and management teams based at Beijing, Oxford and the 10 regional centres. China's National Health Insurance provides electronic linkage to all hospital treatments. The CKB baseline survey and the first re-survey were supported by the Kadoorie Charitable Foundation in Hong Kong. Long-term follow-up was supported by the Wellcome Trust (212946/Z/18/Z, 202922/Z/16/Z, 104085/Z/14/Z, 088158/Z/09/Z), the National Key Research and Development Program of China (2016YFC0900500, 2016YFC0900501, 2016YFC0900504, 2016YFC1303904), and the National Natural Science Foundation of China (81941018, 82192900, 91843302, 91846303). DNA extraction and genotyping was funded by GlaxoSmithKline, and the UK Medical Research Council (MC-PC-13049, MC-PC-14135). The project is supported by core funding from the UK Medical Research Council (MC_UU_00017/1, MC_UU_12026/2, MC_U137686851), Cancer Research UK (C16077/A29186; C500/A16896), and the British Heart Foundation (CH/1996001/9454) to the Clinical Trial Service Unit and Epidemiological Studies Unit and to the MRC Population Health Research Unit at Oxford University. Computation used the Oxford Biomedical Research Computing (BMRC) facility, a joint development between the Wellcome Centre for Human Genetics and the Big Data Institute supported by Health Data Research UK and the NIHR Oxford Biomedical Research Centre; the views expressed are those of the authors and not necessarily those of the NHS, the NIHR, or the Department of Health.

deCODE
We thank the study subjects for their valuable participation.
CHB+DBDS
We thank the Danish Blood Donor Study and Copenhagen Hospital Biobank, including all study participants and staff, for making data available for this research. The Copenhagen Hospital Biobank was supported by the Novo Nordisk Foundation (grant numbers NNF14CC0001 and NNF17OC0027594) and by Department of Clinical Immunology, Copenhagen University Hospital. The establishment and development of the Danish Biobank Register system is supported by the Novo Nordisk Foundation (grant numbers 2010-11-12 and 2009-07-28 to Statens Serum Institut to establish the Danish National Biobank). The initiation of the Danish Blood Donor Study was supported by the Danish Administrative Regions (02/2611) and the Danish Council for Independent Research (09–069412). The study is currently funded by the Danish Administrative Regions and Bio- and Genome Bank Denmark.
eMERGEIII
In eMERGE network (Phase 3 ascertainment), this phase of the eMERGE Network was initiated and funded by the NHGRI through the following grants: U01HG8657 (Kaiser Washington/University of Washington); U01HG8685 (Brigham and Women's Hospital); U01HG8672 (Vanderbilt University Medical Center); U01HG8666 (Cincinnati Children's Hospital Medical Center); U01HG6379 (Mayo Clinic); U01HG8679 (Geisinger Clinic); U01HG8680 (Columbia University Health Sciences); U01HG8684 (Children's Hospital of Philadelphia); U01HG8673 (Northwestern University); U01HG8701 (Vanderbilt University Medical Center serving as the Coordinating Center); U01HG8676 (Partners Healthcare/Broad Institute); and U01HG8664 (Baylor College of Medicine). In eMERGE network (Phase 1 and 2 ascertainment), the eMERGE Network was initiated and funded by NHGRI through the following grants: U01HG006828 (Cincinnati Children's Hospital Medical Center/Boston Children's Hospital); U01HG006830 (Children's Hospital of Philadelphia); U01HG006389 (Essentia Institute of Rural Health, Marshfield Clinic Research Foundation and Pennsylvania State University); U01HG006382 (Geisinger Clinic); U01HG006375 (Group Health Cooperative/University of Washington); U01HG006379 (Mayo Clinic); U01HG006380 (Icahn School of Medicine at Mount Sinai); U01HG006388 (Northwestern University); U01HG006378 (Vanderbilt University Medical Center); and U01HG006385 (Vanderbilt University Medical Center serving as the Coordinating Center) with U01HG004438 (CIDR) and U01HG004424 (the Broad Institute) serving as Genotyping Centers.
Estonian Biobank
The projects leading to this article has received funding from European Union through the European Regional Development Fund Projects No. 2014-2020.4.01.15-0012 GENTRANSMED and MOBEC008, from the European Union's Horizon research and innovation programme under grant agreements No 101017802, No 101016775, No 101095084 and No 810645 and the Estonian Research Council grant PUT (PRG1911). Data analysis was carried out in part in the High-Performance Computing Center of University of Tartu. Views and opinions expressed are however those of the author(s) only and do not necessarily reflect those of the European Union or the European Health and Digital Executive Agency. Neither the European Union nor the granting authority can be held responsible for them.
FinnGen
We want to acknowledge the participants and investigators of FinnGen study. The FinnGen project is funded by two grants from Business Finland (HUS 4685/31/2016 and UH 4386/31/2016) and the following industry partners: AbbVie Inc., AstraZeneca UK Ltd, Biogen MA Inc., Bristol Myers Squibb (and Celgene Corporation & Celgene International II Sàrl), Genentech Inc., Merck Sharp & Dohme LCC, Pfizer Inc., GlaxoSmithKline Intellectual Property Development Ltd., Sanofi US Services Inc., Maze Therapeutics Inc., Janssen Biotech Inc,

Novartis AG, and Boehringer Ingelheim International GmbH. Following biobanks are acknowledged for delivering biobank samples to FinnGen: Auria Biobank (www.auria.fi/biopankki), THL Biobank (www.thl.fi/biobank), Helsinki Biobank (www.helsinginbiopankki.fi), Biobank Borealis of Northern Finland (https://www.ppshp.fi/Tutkimus-ja-opetus/Biopankki/Pages/Biobank-Borealis-briefly-in-English.aspx), Finnish Clinical Biobank Tampere (www.tays.fi/en-US/Research_and_development/Finnish_Clinical_Biobank_Tampere), Biobank of Eastern Finland (www.ita-suomenbiopankki.fi/en), Central Finland Biobank (www.ksshp.fi/fi-FI/Potilaalle/Biopankki), Finnish Red Cross Blood Service Biobank (www.veripalvelu.fi/verenluovutus/biopankkitoiminta), Terveystalo Biobank (www.terveystalo.com/fi/Yritystietoa/Terveystalo-Biopankki/Biopankki/) and Arctic Biobank (https://www.oulu.fi/en/university/faculties-and-units/faculty-medicine/northern-finland-birth-cohorts-and-arctic-biobank). All Finnish Biobanks are members of BBMRI.fi infrastructure (www.bbmri.fi). Finnish Biobank Cooperative -FINBB (https://finbb.fi/) is the coordinator of BBMRI-ERIC operations in Finland. The Finnish biobank data can be accessed through the Fingenious® services (https://site.fingenious.fi/en/) managed by FINBB.

GeisingerMyCode_F60k and GeisingerMyCode_New30k

Regeneron Genetics Center provided funding for the collection of MyCode samples, the generation of genotype data and genotype imputation. Geisinger provided funding for clinical data extraction and genetic association analysis.

Generation Scotland

Generation Scotland received core support from the Chief Scientist Office of the Scottish Government Health Directorates [CZD/16/6] and the Scottish Funding Council [HR03006] and is currently supported by the Wellcome Trust [216767/Z/19/Z]. Genotyping of the GS:SFHS samples was carried out by the Genetics Core Laboratory at the Edinburgh Clinical Research Facility, University of Edinburgh, Scotland and was funded by the Medical Research Council UK and the Wellcome Trust (Wellcome Trust Strategic Award “STratifying Resilience and Depression Longitudinally” (STRADL) Reference 104036/Z/14/Z). Caroline Hayward was supported by an MRC University Unit Programme Grant “QTL in Health and Disease” (U. MC_UU_00007/10). We are grateful to all the families who took part, the general practitioners and the Scottish School of Primary Care for their help in recruiting them, and the whole Generation Scotland team, which includes interviewers, computer and laboratory technicians, clerical workers, research scientists, volunteers, managers, receptionists, healthcare assistants and nurses.

Genes & Health

Genes & Health is/has recently been core-funded by Wellcome (WT102627, WT210561), the Medical Research Council (UK) (M009017, MR/X009777/1, MR/X009920/1), Higher Education Funding Council for England Catalyst, Barts Charity (845/1796), Health Data Research UK (for London substantive site), and research delivery support from the NHS National Institute for Health Research Clinical Research Network (North Thames). Genes & Health is/has recently been funded by Alnylam Pharmaceuticals, Genomics PLC; and a Life Sciences Industry Consortium of Astra Zeneca PLC, Bristol-Myers Squibb Company, GlaxoSmithKline Research and Development Limited, Maze Therapeutics Inc, Merck Sharp & Dohme LLC, Novo Nordisk A/S, Pfizer Inc, Takeda Development Centre Americas Inc.

We thank Social Action for Health, Centre of The Cell, members of our Community Advisory Group, and staff who have recruited and collected data from volunteers. We thank the NIHR National Biosample Centre (UK Biocentre), the Social Genetic & Developmental Psychiatry Centre (King's College London), Wellcome Sanger Institute, and Broad Institute for sample processing, genotyping, sequencing and variant annotation.

This work uses data provided by patients and collected by the NHS as part of their care and support. This research utilised Queen Mary University of London's Apocrita HPC facility, supported by QMUL Research-IT, [345].

We thank: Barts Health NHS Trust, NHS Clinical Commissioning Groups (City and Hackney, Waltham Forest, Tower Hamlets, Newham, Redbridge, Havering, Barking and Dagenham), East London NHS Foundation Trust, Bradford Teaching Hospitals NHS Foundation Trust, Public Health England (especially David Wyllie), Discovery Data Service/Endeavour Health Charitable Trust (especially David Stables), Voror Health Technologies Ltd (especially Sophie Don), NHS England (for what was NHS Digital) - for GDPR-compliant data sharing backed by individual written informed consent.

Most of all we thank all of the volunteers participating in Genes & Health.

Health and Retirement Study

HRS is supported by the National Institute on Aging (NIA U01AG009740). The genotyping was funded separately by the National Institute on Aging (RC2 AG036495, RC4 AG039029). Our genotyping was conducted by the NIH Center for Inherited Disease Research (CIDR) at Johns Hopkins University. Genotyping quality control and final preparation of the data were performed by the Genetics Coordinating Center at the University of Washington and the School of Public Health at the University of Michigan.

Hong Kong Degenerative Disc Disease Population Cohort (HKDDDPC)

Hong Kong Research Grants Council grants: AoE/M-04/04, T12-708/12-N, 776613, C7044-19G. We acknowledge Ken Cheung and Danny Chan for their contributions to the establishment and follow-up of the Hong Kong Degenerative Disc Disease population cohort.

HUNT study

The Trøndelag Health Study (HUNT) is a collaboration between HUNT Research Centre (Faculty of Medicine and Health Sciences, Norwegian University of Science and Technology NTNU), Trøndelag County Council, Central Norway Regional Health Authority, and the Norwegian Institute of Public Health. The genotyping was financed by the National Institute of health (NIH), University of Michigan, The Norwegian Research council, and Central Norway Regional Health Authority and the Faculty of Medicine and Health Sciences, Norwegian University of Science and Technology (NTNU). The genotype quality control and imputation has been conducted by the K.G. Jebsen center for genetic epidemiology, Department of public health and nursing, Faculty of medicine and health sciences, Norwegian University of Science and Technology (NTNU).

INTERMOUNTAIN

We thank all the subjects that participated in this study for the valuable contribution.

J. Patrick Pett

This project has received funding from the European Union’s Horizon 2020 research and innovation programme under the Marie-Skłodowska-Curie grant agreement No. 101026233.

JoCoOA

The Johnston County Osteoarthritis Project (JoCoOA) was funded in part by: Association of Schools of Public Health/Centers for Disease Control and Prevention (CDC) S043, S1734, S3486; CDC U01 DP003206 and U01 DP006266; National Institutes of Health/National Institute of Arthritis and Musculoskeletal and Skin Diseases P60AR30701, P60AR049465, P60AR064166, and P30AR072580.

Joyce van Meurs and Cindy G. Boer

Joyce van Meurs and Cindy G. Boer are funded by by ReumaNederland (Project number: LLP-34).

LifeLines

The LifeLines Cohort Study, and generation and management of GWAS genotype data for the LifeLines Cohort Study is supported by the Netherlands Organization of Scientific Research NWO (grant 175.010.2007.006), the Economic Structure Enhancing Fund (FES) of the Dutch government, the Ministry of Economic Affairs, the Ministry of Education, Culture and Science, the Ministry for Health, Welfare and Sports, the Northern Netherlands Collaboration of Provinces (SNN), the Province of Groningen, University Medical Center Groningen, the University of Groningen, Dutch Kidney Foundation and Dutch Diabetes Research Foundation. The authors are grateful to the study participants, the staff from the LifeLines Cohort Study and the contributing research centers delivering data to LifeLines and the participating general practitioners and pharmacists.

Mass General Brigham Biobank

We thank the participants and staff of the Mass General Brigham Biobank for providing samples, genomic data, and health information data. PHL was supported by NIMH grants R01 MH119243, R01 MH124694, R01 MH116037, R01 GM148494, and R01 MH120219.

Million Veteran Program

Million Veteran Program is a national, voluntary research program funded entirely by the Department of Veterans Affairs Office of Research & Development and is an important partnership between VA and Veterans to learn more about how genes affect health and to improve health care for Veterans. This research is based on data from the Million Veteran Program, Office of Research and Development, Veterans Health Administration, and was supported by award #I01RX002745 (J.A.S.). This publication does not represent the views of the Department of Veteran Affairs or the United States Government.

The Netherlands Epidemiology of Obesity (NEO) study

We thank all individuals who participated in the NEO study, and all participating general practitioners for inviting eligible participants. The authors also thank all P.R. van Beelen and all research nurses for collection of the data, P.J. Noordijk and her team for sample handling and storage, and I. de Jonge for data management of the NEO study.

The NEO study was supported by the participating Departments, the Division and the Board of Directors of the Leiden University Medical Centre, and by the Leiden University, Research Profile Area 'Vascular and Regenerative Medicine'. Assessment of the osteoarthritis status was obtained from the Dutch Arthritis Society and the IMI-APPROACH project.

NHS: Nurses Health Study and Nurses Health Study II

We thank the participants and staff of the Nurses Health Study and Nurses Health Study II for their valuable contributions. The Nurses Health Study was supported by NIH grants UM1 CA186107, P01 CA87969 and R01 CA49449, and the Nurses Health Study II was supported by U01 CA176726 and R01 CA67262.

NIHRBioResource

We thank NIHR BioResource volunteers for their participation, and gratefully acknowledge NIHR BioResource centres, NHS Trusts and staff for their contribution. We thank the National Institute for Health and Care Research, NHS Blood and Transplant, and Health Data Research UK as part of the Digital Innovation Hub Programme. The views expressed are those of the author(s) and not necessarily those of the NHS, the NIHR or the Department of Health and Social Care.

Norwegian Arthroplasty Register (NAR)

We thank the Norwegian surgeons for their contribution by reporting to the NAR and the staff at the NAR for their effort. The NAR is funded by the health trusts Helse Vest RHF and Helse Bergen HF. The NAR cohort and genotyping was funded by Versus Arthritis grants 21163 and 18030, Wellcome grant 098051, and the Western Norway Regional Health Authority.

The Osteoarthritis Initiative (OAI)

The Osteoarthritis Initiative (OAI) is a public-private partnership comprised of five contracts (N01-AR-2–2258; N01-AR-2–2259; N01-AR-2–2260; N01-AR-2–2261; N01-AR-2–2262) funded by the NIH. Analyses were supported by NIH R01AR075356 and NIH P30DK072488.

QIMR [Over 50's (Aged) and Osteoarthritis (OA) Studies]

We thank Fran Boyle and Len Roberts for their work in constructing the Over 50s (Aged Study) questionnaire, Olivia Zheng for administering the mailout, John Pearson for data management and Nirmala Pandeya for data cleaning. Twins from the Aged Study were drawn from the Australian National Health and Medical Research Council (NHMRC) Twin Registry. This work was partly supported by a donation from Mr George Landers, and benefited from funding from NHMRC to Ian B. Hickie (931215-Project Grant, and 953208-Program Grant) and Nicholas G. Martin (941177).

QIMR- PISA [Prospective Imaging Study of Ageing: Genes, Brain and Behaviour]

Data acquired in the preparation of this manuscript was obtained from The Prospective Imaging Study of Ageing (PISA) database. The PISA project was funded by the Australian National Health and Medical Research Council (NHMRC) (Grant ID: APP1095227).

QIMR- GBP [Australian Genetics of Bipolar Disorder Study]

We thank the participants for giving their time and support for this project. We acknowledge and thank M. Steffens for her generous donations in loving memory of J. Banks. Data collection was funded and data analysis was supported by the Australian National Health and Medical Research Council (No. APP1138514) to S.E.M.. S.E.M. is supported by a National Health and Medical Research Council Investigator Grant (No. APP1172917).

QIMR- AGDS [Australian Genetics of Depression Study]

We thank all the participants for giving their time to contribute to this study. We wish to thank all the people who helped in the conception, implementation, beta testing, media campaign and data cleaning. We would specifically like to acknowledge Ken Kendler, Patrick Sullivan, Andrew McIntosh and Cathryn Lewis for input on the questionnaire; Lorelle Nunn, Mary Ferguson, Lucy Winkler and Natalie Garden for data and sample collection; Natalia Zmicerevska, Alissa Nichles and Candace Brennan for participant recruitment support. Jonathan Davies, Luke Lowrey and Valeriano Antonini for support with IT aspects; Vera Morgan and Ken Kirkby for help with the media campaign. The AGDS was primarily funded by National Health and Medical Research Council (NHMRC) of Australia (Grant No. APP1086683) to N.G.M.. This work was further supported by NHMRC grants (No. 1145645, 1078901 and 1087889). N.G.M. is supported by a NHMRC Investigator Grant (No. APP1172990).

RAAK study

We thank all study participants of the RAAK study. The Leiden University Medical Centre have and are supporting the RAAK. We thank Rachid Mahdad (Alrijne), Enrike van der Linden, Robert van der Wal, Peter van Schie, Shaho Hasan, Maartje Meijer, Daisy Latijnhouwers, Anika Rabelink-Hoogenstraaten, and Geert Spierenburg (LUMC) for their contribution to the collection of the joint tissue. Funding to assess RAAK molecular datasets was obtained within the scope of the Medical Delta programs Regenerative Medicine 4D: Generating complex tissues with stem cells and printing technology and Improving Mobility with Technology, the Dutch Scientific Research council (NWO) project OCENW.GROOT (2019.079), ZonMW VICI scheme (91816631/528), NWA-ORC LOAD (1389.20.009), and Dutch Arthritis Society (LPP-32).

RIKEN Study

RIKEN study was supported by Japan Agency for Medical Research and Development (AMED) Grant (Grant number: JP21ek0109555, JP21tm0424220, JP21ck0106642, JP23ek0410114 and JP23tm0424225), Japan Society for the Promotion of Science (JSPS) KAKENHI grant

JP20H00462 and JP22H03207, Takeda Hosho Grants for Research in Medicine, The JCR Grant for Promoting Basic Rheumatology. We would express our sincere gratitude to the study participants, the research and medical staffs in the the BioBank Japan project.

The Rotterdam Study

The Rotterdam Study is funded by Erasmus Medical Center and Erasmus University, Rotterdam, Netherlands Organization for the Health Research and Development (ZonMw), the Research Institute for Diseases in the Elderly (RIDE), the Ministry of Education, Culture and Science, the Ministry for Health, Welfare and Sports, the European Commission (DG XII), and the Municipality of Rotterdam. The Rotterdam Study GWAS datasets are supported by the Netherlands Organisation of Scientific Research NWO Investments (nr. 175.010.2005.011, 911-03-012), the Genetic Laboratory of the Department of Internal Medicine, Erasmus MC, the Research Institute for Diseases in the Elderly (014-93-015; RIDE2), the Netherlands Genomics Initiative (NGI)/Netherlands Organisation for Scientific Research (NWO) Netherlands Consortium for Healthy Aging (NCHA), project nr. 050-060-810.

Shimane CoHRE Study

Shimane CoHRE Study was is supported by Japan Society for the Promotion of Science (JSPS) KAKENHI (Grant number: 19H03996).

SHIP START and SHIP TREND

SHIP (Study of Health in Pomerania): SHIP is part of the Community Medicine Research net of the University of Greifswald, Germany, which is funded by the Federal Ministry of Education and Research (grants no. 01ZZ9603, 01ZZ0103, and 01ZZ0403), the Ministry of Cultural Affairs as well as the Social Ministry of the Federal State of Mecklenburg-West Pomerania, and the network 'Greifswald Approach to Individualized Medicine (GANI_MED)' funded by the Federal Ministry of Education and Research (grant 03IS2061A). Genome-wide data were supported by the Federal Ministry of Education and Research (grant no. 03ZIK012) and a joint grant from Siemens Healthcare, Erlangen, Germany and the Federal State of Mecklenburg- West Pomerania.

SIMPLER-SIMPLER, SIMPLER-COSMC and SIMPLER-SMCC

The study was supported by funding from the Swedish Research Council (https://www.vr.se; grants No. 2015-03257, 2017-00644, 2017-06100, and 2019-01291 to Karl Michaëlsson) and funding from Olle Engkvist Byggmästares stiftelse (SOEB). We acknowledge the national research infrastructure SIMPLER for generation and availability of data and computational facilities and resources. SIMPLER receives funding through the Swedish Research Council under the grant No. 2017-00644 and 2021-00160 (to Uppsala University and Karl Michaëlsson). The computations were performed on resources provided by the Swedish National Infrastructure for Computing's (http://www.snic.se) support for sensitive data SNIC-SENS through the Uppsala Multidisciplinary Center for Advanced Computational Science (UPPMAX) under Project SIMP2021019. SNIC is financially supported by the Swedish Research Council. The funders had no role in study design, data collection and analysis, decision to publish, or preparation of the manuscript.

Tohoku Medical Megabank Organisation

This work was supported by the following programs by the Japan Agency for Medical Research and Development (AMED) and the Ministry of Education, Culture, Sports, Science and Technology (MEXT): the Tohoku Medical Megabank Project [JP20km0105001 and JP20km0105002], and the Facilitation of R&D Platform for the AMED Genome Medicine Support [JP20km0405001] of the Platform Program for Promotion of Genome Medicine (P3GM).

TwinsUK

TwinsUK is funded by the Wellcome Trust, Medical Research Council, European Union, Chronic Disease Research Foundation (CDRF), Zoe Global Ltd and the National Institute for

Health Research (NIHR)-funded BioResource, Clinical Research Facility and Biomedical Research Centre based at Guy's and St Thomas' NHS Foundation Trust in partnership with King's College London.

UK Biobank

This research has been conducted using data from UK Biobank, a major biomedical database (www.ukbiobank.ac.uk) under application number 9979.

UK Household Longitudinal Study (UKHLS)

UKHLS is led by the Institute for Social and Economic Research at the University of Essex. The survey was conducted by NatCen, and the genome-wide scan data were analysed and deposited by the Wellcome Sanger Institute. Information on how to access the data can be found on the Understanding Society website https://www.understandingsociety.ac.uk/.

Ethics and study approval

arcOGEN

The arcOGEN study was ethically approved by appropriate review committees, and the prospective collections were approved by the National Research Ethics Service in the United Kingdom. All subjects in this study provided written, informed consent.

ARGO-Athens

All studies were approved by the relevant hospital Institutional Review Board and conducted in accordance with the principles set out in the Declaration of Helsinki. All patients provided written informed consent prior to participation.

ARGO-Larissa

Verbal informed consent was given by all research participants prior to the collection of blood samples for the research. The research participant recruitment, consent process, and study protocol were approved by the Institutional Review Board of the University Hospital of Larissa and conform to the ethical principles set out in the Declaration of Helsinki (1975)

BioMe BioBank Program

Study participants were recruited from the BioMe Biobank Program of the Charles Bronfman Institute for Personalized Medicine at Mount Sinai Medical Center from 2007 onward. TheBioMe Biobank Program (Institutional Review Board 07-0529) operates under a Mount Sinai Institutional Review Board-approved research protocol. All study participants provided written informed consent.

Bunkyo Health Study

The study protocol was approved by the ethics committee of Juntendo University in November 2015 (Nos. 2015078, 2016138, 2016131, 2017121, and 2019085). This study was carried out in accordance with the principles outlined in the Declaration of Helsinki. All participants gave written informed consent and were informed that they had the right to withdraw from the trial at any time.

China Kadoorie Biobank

Ethical approval for CKB was obtained jointly from the University of Oxford, the Chinese Centre for Disease Control and Prevention (CCDC) and the regional CCDC from the 10 study areas. All participants provided written informed consent. Genotyping data were exported from China to the Oxford CKB International Coordinating Centre under Data Export Approvals 2014-13 and 2015-39 from the Office of Chinese Human Genetic Resource Administration.

deCODE

All participants who donated samples gave informed consent and the National Bioethics Committee of Iceland approved the study (VSN 14-148) which was conducted in agreement

with conditions issued by the Data Protection Authority of Iceland. Personal identities of the participant's data and biological samples were encrypted by a third-party system (Identity Protection System), approved and monitored by the Data Protection Authority.

CHB+DBDS

Genetics of Pain and Degenerative Musculoskeletal Diseases – a Genome-Wide Association study on repository samples from Copenhagen Hospital Biobank" is approved by the Danish National Committee on Health Research Ethics (NVK- 1803812) and the Capital Region Data Protection Office (P-2019-51). The Danish Blood Donor Study is approved by the Capital Region Data Protection Office and the Scientific Ethical Committee system.

eMERGEIII

All 11 sample collection sites consented participants under Institutional Review Board (IRB)-approved protocols and the two sequencing centers had IRB-approved protocols that deferred consent to the participating sites. Protocol numbers are as follows:
Partners Healthcare (2015P000929), Baylor College of Medicine (#H-40455). https://emerge-network.org/phase-iii-consent-forms/

Estonian Biobank

The activities of the EstBB are regulated by the Human Genes Research Act, which was adopted in 2000 specifically for the operations of the EstBB. Individual level data analysis in the EstBB was carried out under ethical approval [1.1-12/624] from the Estonian Committee on Bioethics and Human Research (Estonian Ministry of Social Affairs), using data according to release application [N05] from the Estonian Biobank.

FinnGen

Patients and control subjects in FinnGen provided informed consent for biobank research, based on the Finnish Biobank Act. Alternatively, separate research cohorts, collected prior the Finnish Biobank Act came into effect (in September 2013) and start of FinnGen (August 2017), were collected based on study-specific consents and later transferred to the Finnish biobanks after approval by Fimea (Finnish Medicines Agency), the National Supervisory Authority for Welfare and Health. Recruitment protocols followed the biobank protocols approved by Fimea. The Coordinating Ethics Committee of the Hospital District of Helsinki and Uusimaa (HUS) statement number for the FinnGen study is Nr HUS/990/2017.
The FinnGen study is approved by Finnish Institute for Health and Welfare (permit numbers: THL/2031/6.02.00/2017, THL/1101/5.05.00/2017, THL/341/6.02.00/2018, THL/2222/6.02.00/2018, THL/283/6.02.00/2019, THL/1721/5.05.00/2019 and THL/1524/5.05.00/2020), Digital and population data service agency (permit numbers: VRK43431/2017-3, VRK/6909/2018-3, VRK/4415/2019-3), the Social Insurance Institution (permit numbers: KELA 58/522/2017, KELA 131/522/2018, KELA 70/522/2019, KELA 98/522/2019, KELA 134/522/2019, KELA 138/522/2019, KELA 2/522/2020, KELA 16/522/2020), Findata permit numbers THL/2364/14.02/2020, THL/4055/14.06.00/2020,,THL/3433/14.06.00/2020, THL/4432/14.06/2020, THL/5189/14.06/2020, THL/5894/14.06.00/2020, THL/6619/14.06.00/2020, THL/209/14.06.00/2021, THL/688/14.06.00/2021, THL/1284/14.06.00/2021, THL/1965/14.06.00/2021, THL/5546/14.02.00/2020 and Statistics Finland (permit numbers: TK-53-1041-17 and TK/143/07.03.00/2020 (earlier TK-53-90-20)).
The Biobank Access Decisions for FinnGen samples and data utilized in FinnGen Data Freeze 7 include: THL Biobank BB2017_55, BB2017_111, BB2018_19, BB_2018_34, BB_2018_67, BB2018_71, BB2019_7, BB2019_8, BB2019_26, BB2020_1, Finnish Red Cross Blood Service Biobank 7.12.2017, Helsinki Biobank HUS/359/2017, Auria Biobank AB17-5154 and amendment #1 (August 17 2020), Biobank Borealis of Northern Finland_2017_1013, Biobank of Eastern Finland 1186/2018 and amendment 22 § /2020, Finnish Clinical Biobank Tampere MH0004 and amendments (21.02.2020 & 06.10.2020), Central Finland Biobank 1-2017, and Terveystalo Biobank STB 2018001.

GeisingerMyCode_F60k and GeisingerMyCode_New30k

MyCode Governing Board and an external Ethics Advisory Council approved the study and informed consent was obtained from all subjects as detailed in this reference: [315].

Generation Scotland

Ethical approval for the GS:SFHS study was obtained from the Tayside Committee on Medical Research Ethics (on behalf of the National Health Service).

Genes and Health

East London Genes & Health (ELGH) operates under ethical approval, 14/LO/1240, from London South East NRES Committee of the Health Research Authority, dated 16 September 2014. Stage 1 volunteers complete a brief questionnaire, give consent to lifelong EHR linkage and donate a saliva sample for DNA extraction and genetic tests.
A favourable ethical opinion for the main Genes & Health research study was granted by NRES Committee London - South East (reference 14/LO/1240) on 16 Sept 2014.

Health and Retirement Study

This study was approved by the University of Michigan Institutional Review Board (IRB), and informed consent was obtained from all participants

Hong Kong Degenerative Disc Disease Population Cohort (HKDDDPC)

Informed consent was obtained from participants and ethics was approved by a local institutional board.

HUNT study

All genotyped participants have signed a written informed consent regarding the use of data from questionnaires, biological samples and linkage to other registries for research purposes. The current study was approved by the Regional Committee for Medical and Health Research Ethics (REK) 2015/573.

INTERMOUNTAIN

Subjects (≥ 18 years and U.S. resident) visiting an Intermountain Health facility and willing to provide a blood sample or a blood sample that would otherwise be discarded following a routine standard of care blood draw procedure were recruited for study participation. Subjects were informed of and provided written and verbal consent to the study protocol and procedures. This study was approved by an Institutional Review Board (Intermountain Health, Salt Lake City, UT USA).

JoCoOA

This study was approved by the Institutional Review Boards of the Centers for Disease Control and Prevention and the University of North Carolina at Chapel Hill. Informed consent was obtained from all participants for collection of data and biospecimens including use for genetic studies.

LifeLines

The Lifelines protocol has been approved by the UMCG Medical ethical committee under number 2007/152

Mass General Brigham Biobank

The Mass General Brigham IRB has approved the Mass General Brigham Biobank (Biobank) to distribute data and samples collected via the Biobank protocol to MGB investigators for research use. The PI has used de-identified MGB samples with the approved MGB Biobank Data Use Agreement. All data analysis was done internally at MGB and aligned with the terms of the Biobank Consent form.

Million Veteran Program

The work described in this manuscript received ethical and study protocol approval from the Veterans Affairs Central Institutional Review Board as well as the University of Alabama at Birmingham (UAB) in accordance with the principles outlined in the Declaration of Helsinki.

UKB data were analyzed after approved review of proposal no. 30350 by the UKB Research Ethics and Governance committee.

The Netherlands Epidemiology of Obesity (NEO) study

The study was approved by the medical ethical committee of the Leiden University Medical Center (LUMC).

NHS: Nurses Health Study and Nurses Health Study II

This study was approved by the Institutional Review Boards of the Harvard T. H. Chan School of Public Health and Brigham and Women's Hospital. Informed consent was obtained from all subjects for the collection of biospecimens for genotyping and the use of their genotype and de-identified data for research.

NIHRBioResource

Ethics approval and consent to participate The UK IBD BioResource has been reviewed and approved by Cambridge Central Research Ethics Committee (ref 15/EE/0286). All patients provided written informed consent at initial recruitment. For this nested phase 2 study within in the UK IBD BioResource, Human Research Ethics Committee approval was granted by London – Stanmore Research Ethics Committee (Ref 19/LO/1891). Participants in this study provided consent by progressing from the patient information leaflet to the questionnaire.

Norwegian Arthroplasty Register (NAR)

The current study was approved by the Regional Committee for Medical and Health Research Ethics (REK) 2015/573. All patients in NAR have signed a written informed consent regarding the use of data from and linkage to other registries for research purposes.

The Osteoarthritis Initiative (OAI)

The study was approved by the IRB at each participating clinical center. All participants provided informed consent for collection of data and biospecimens.

QIMR [Over 50's (Aged) and Osteoarthritis (OA) Studies]

Informed consent was obtained from participants. Ethical approval for these studies has been granted by the QIMR Berghofer Medical Research Institute Human Research Ethics Committee (P1204).

QIMR- PISA [Prospective Imaging Study of Ageing: Genes, Brain and Behaviour]

Informed consent was obtained from participants. The components of the PISA study protocol used in this project (P2210) has approval from the Human Research Ethics Committees (HREC) of QIMR Berghofer Medical Research Institute.

QIMR- GBP [Australian Genetics of Bipolar Disorder Study]

Participants who completed the online survey provided written informed consent for the study online. Participants who completed the abridged paper survey completed a paper consent form. Ethics approval for all aspects of the project was obtained from the QIMR Berghofer Medical Research Institute Human Research Ethics Committee for the GBP (P3408) and AGDS (P2118) studies.

QIMR- AGDS [Australian Genetics of Depression Study]

Participants provided written informed consent for the study online. All protocols and questionnaires for the AGDS cohort were approved by the QIMR Berghofer Medical Research Institute Human Research Ethics Committee (P2118).

RAAK study

RAAK-study is granted by the medical ethics committee of Leiden University Medical Center with protocol numbers P08.239/P19.013.

RIKEN Study

The Ethical committee of RIKEN Yokohama Institute approved the study. Informed consent was obtained from all subjects.

The Rotterdam Study

The Rotterdam Study has been approved by the Medical Ethics Committee of the Erasmus MC (registration number MEC 02.1015) and by the Dutch Ministry of Health, Welfare and Sport (Population Screening Act WBO, license number 1071272-159521-PG). The Rotterdam Study has been entered into the Netherlands National Trial Register (NTR; www.trialregister.nl) and into the WHO International Clinical Trials Registry Platform (ICTRP; www.who.int/ictrp/network/primary/en/) under shared catalogue number NTR6831. All participants provided written informed consent to participate in the study and to have their information obtained from treating physicians.

Shimane CoHRE Study

The study protocol was approved by the Ethics Committee of Shimane University School of Medicine. Written informed consent was obtained from all participants.

SHIP START and SHIP TREND

Written informed consents were obtained from all participants according to the principles of the Declaration of Helsinki. The studies were approved by the Ethics Committee at the University Medicine Greifswald, Germany (approval number BB 39/08).

SIMPLER-SIMPLER, SIMPLER-COSMC and SIMPLER-SMCC

Written informed consent was obtained from all participants.

Tohoku Medical Megabank Organisation

The study was approved by the Institutional Review Board of Tohoku Medical Megabank Organization, Tohoku University (2021-4-048), and all the participants gave written informed consents.

TwinsUK

Ethics approval was obtained from the Guy's and St. Thomas' Hospital Ethics Committee. Written informed consent was obtained from every participant.

UK Biobank

All participants signed consent to participate in UK Biobank and UK Biobank's scientific protocol and operational procedures were reviewed and approved by the North West Research Ethics Committee (REC reference number 06/MRE08/65), North West Multicentre Research Ethics Committee (REC reference 11/NW/0382), the National Information Governance Board for Health and Social Care and the Community Health Index Advisory Group.

UK Household Longitudinal Study (UKHLS)

The UKHLS has been approved by the University of Essex Ethics Committee, and informed consent was obtained from every participant.

Supplementary Figures

Supplementary Figure 1. Joint-specific genetic architecture of osteoarthritis.

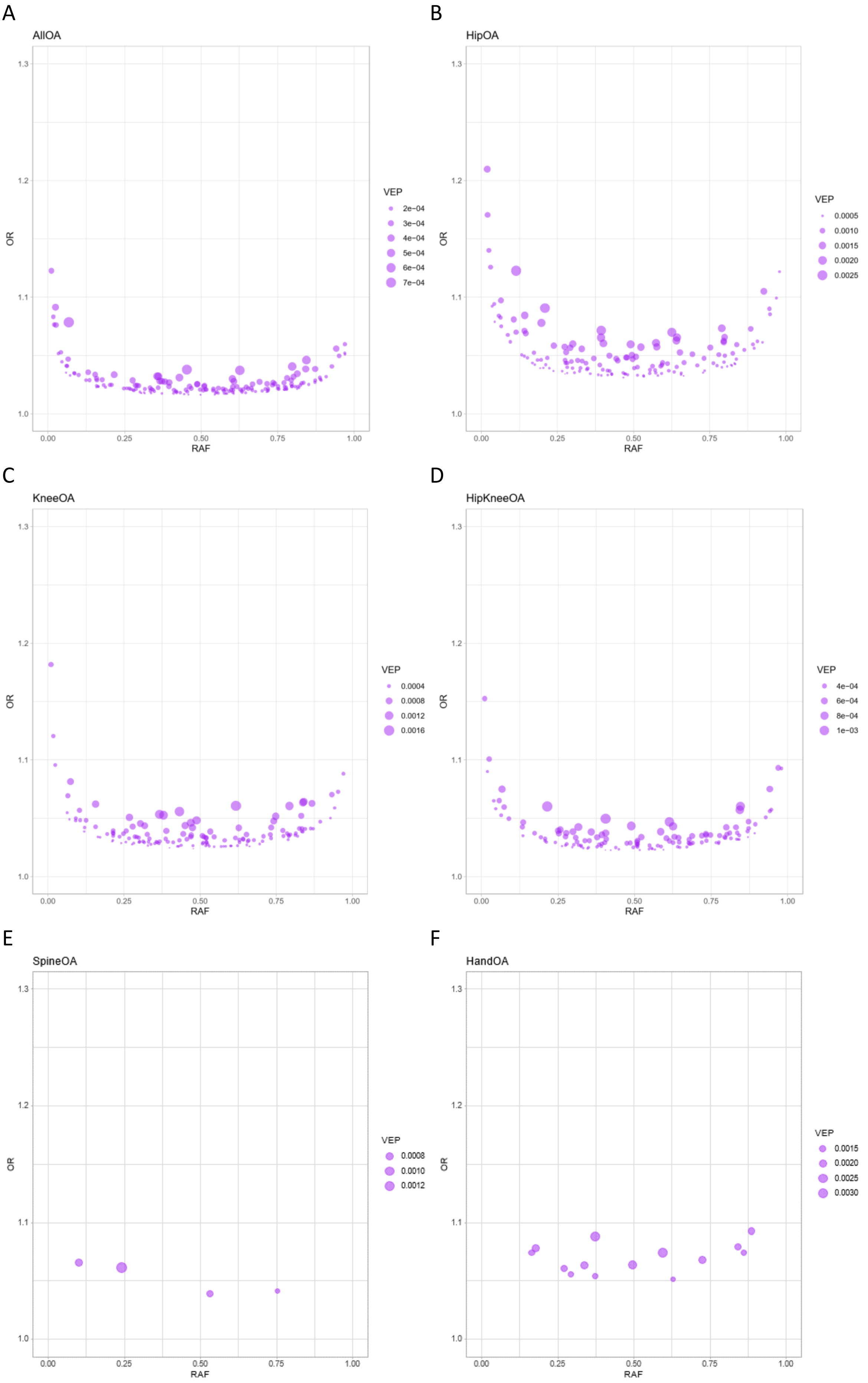

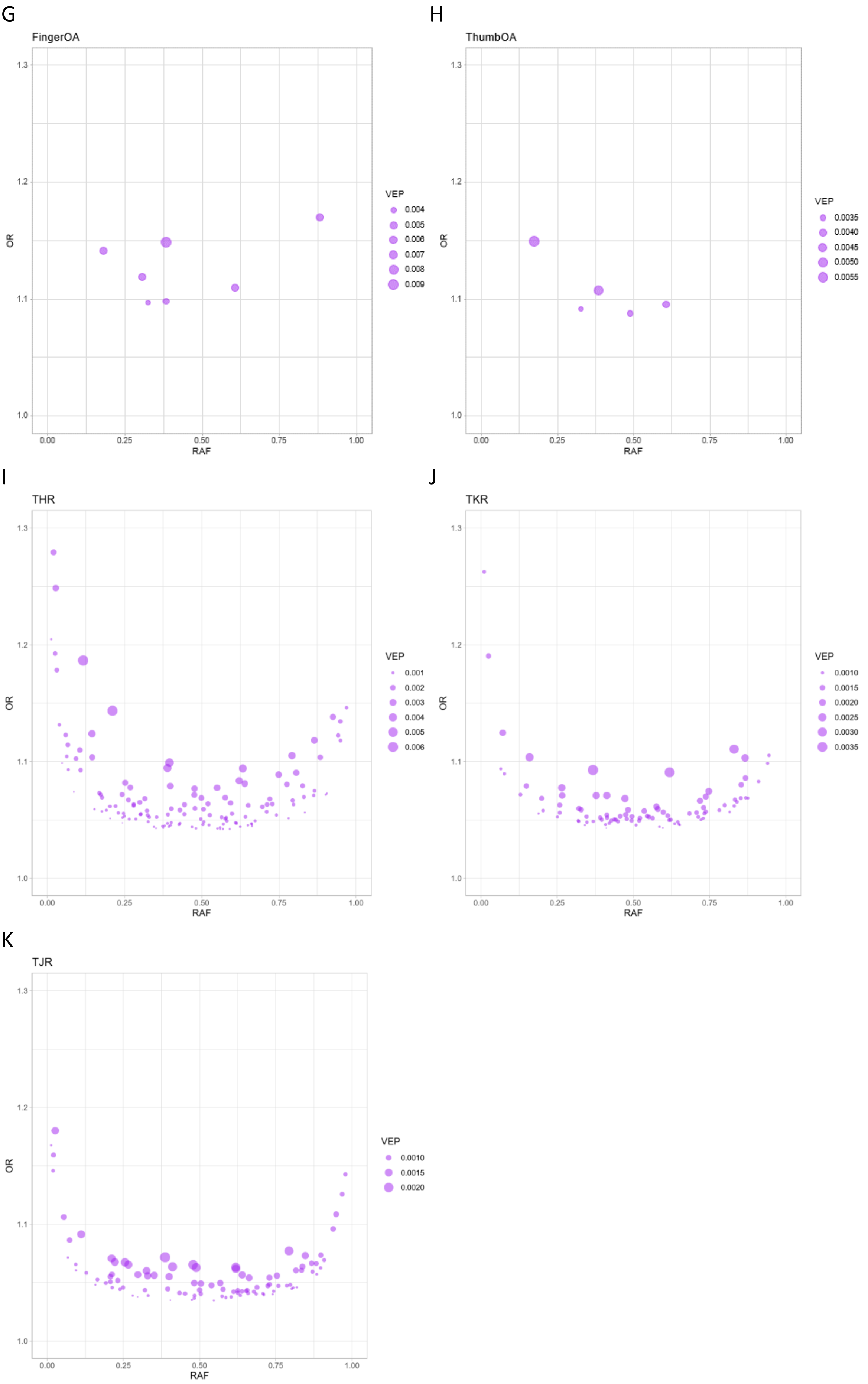

Per phenotype meta-analysis based odd ratios (OR) of the 962 index variants (y-axis) as a function of their risk allele frequency (x-axis; RAF), and phenotypic variance explained (VEP) for each variant indicated by the varying size of each circle. Osteoarthritis at any site (ALLOA) n=175 variants, hip osteoarthritis (HipOA) n=151 variants, knee osteoarthritis (KneeOA) n=146 variants, hip and/or knee osteoarthritis (HipKneeOA) n=131 variants, spine osteoarthritis (SpineOA) n=4 variants, hand osteoarthritis (HandOA) n=14 variants, finger osteoarthritis (FingerOA) n=7 variants, thumb osteoarthritis (ThumbOA) n=5 variants, total hip replacement (THR) n=136 variants, total knee replacement (TKR) n=92 variants and total hip and/or knee replacement (total joint replacement; TJR) n=101 variants.

Supplementary Figure 2. Manhattan and quantile-quantile plots for the main analysis, non-European ancestry and sex-stratified meta-analysis.

Main analysis

Osteoarthritis at any site (n=489,975 cases and 1,472,094 controls)

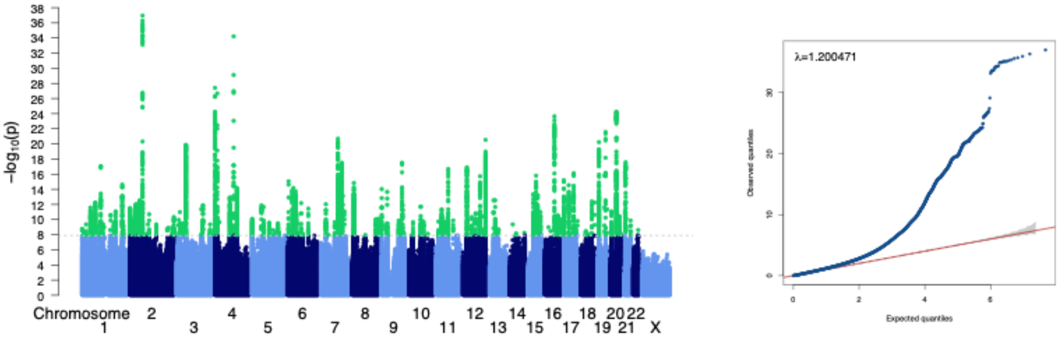

Hip and/or knee osteoarthritis (n=213,839 cases and 1,080,482 controls)

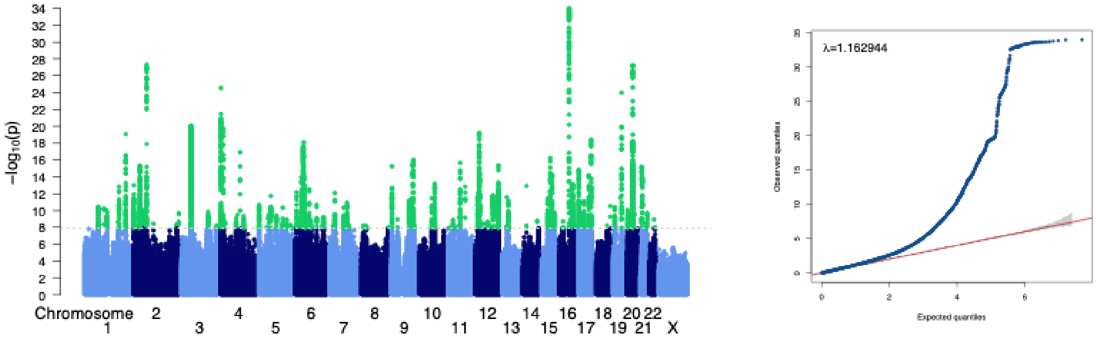

Hip osteoarthritis (n= 97,328 cases and 1,055,379 controls)

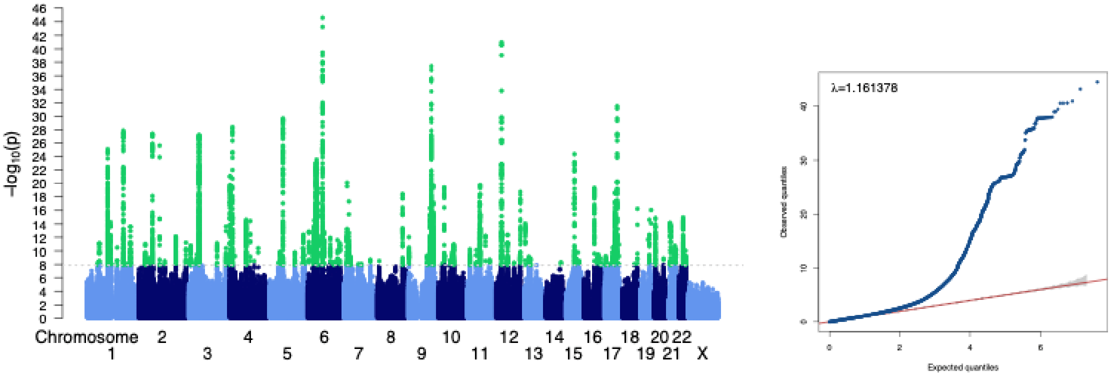

Knee osteoarthritis (n=172,256 cases and 114,4244 controls)

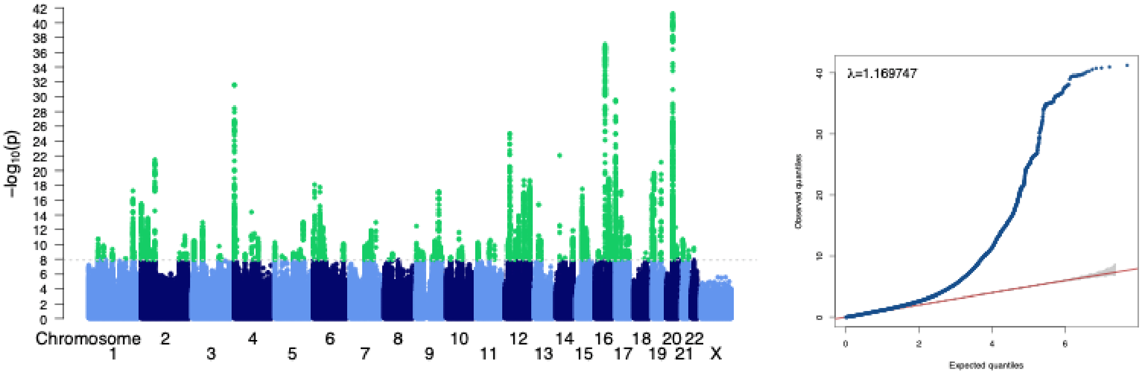

Hand osteoarthritis (n= 40,904 cases and 849,561 controls)

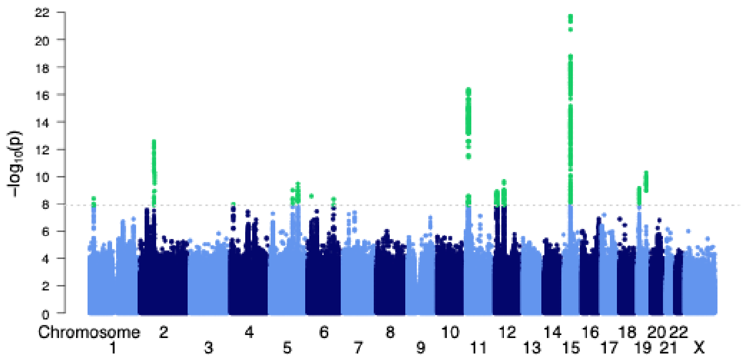

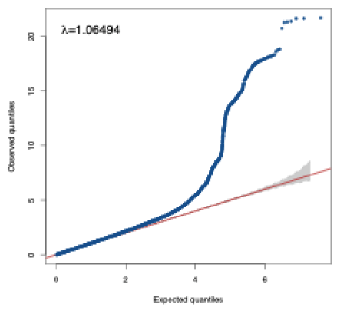

Finger osteoarthritis (n= 16,455 cases and 709,038 controls)

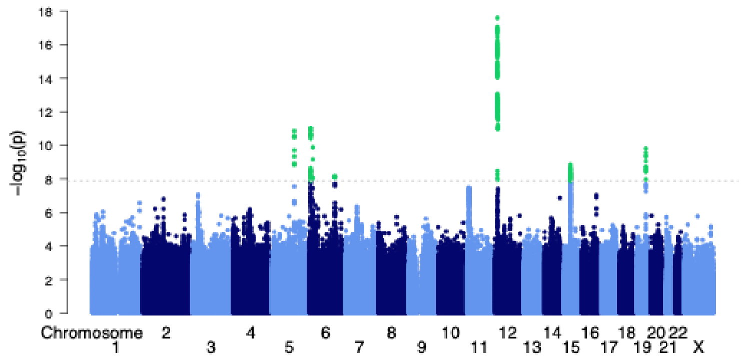

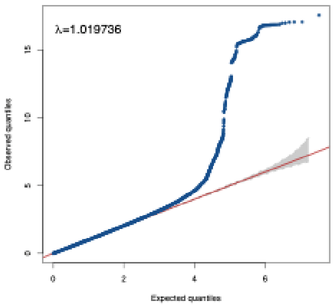

Thumb osteoarthritis (n=16,892 cases and 784,547 controls)

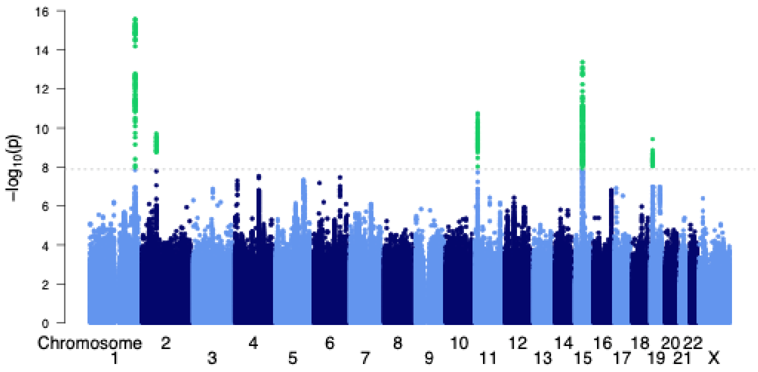

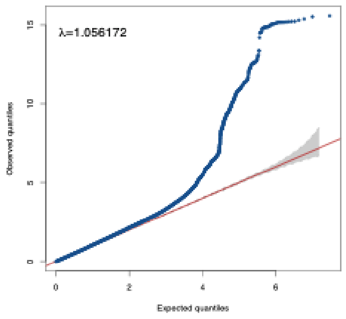

Spine osteoarthritis (n=76,169 cases and 856,428 controls)

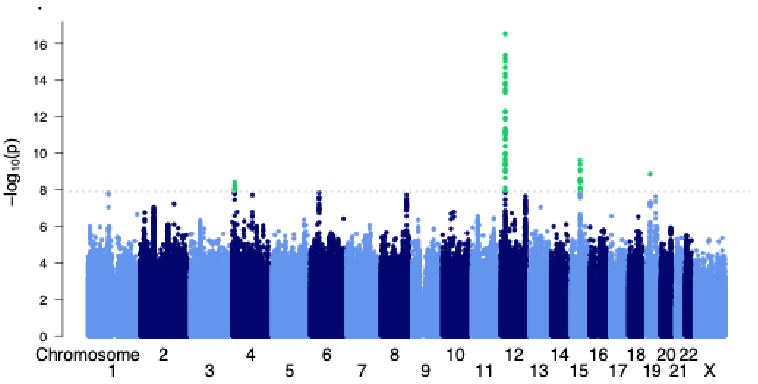

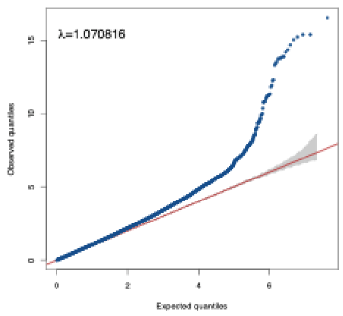

Total joint replacement (n=77,192 cases and 789,225 controls)

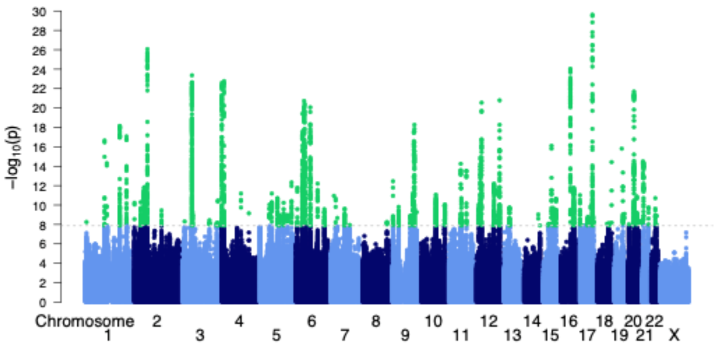

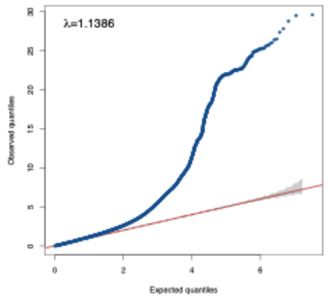

Total hip replacement (n=49,874 cases and 981,172 controls)

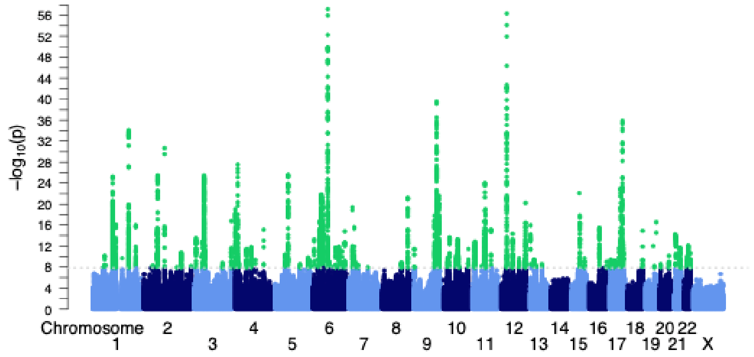

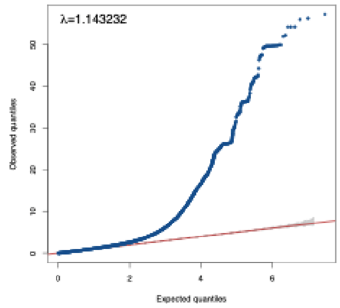

Total knee replacement (n=48,161 cases and 958,463 controls)

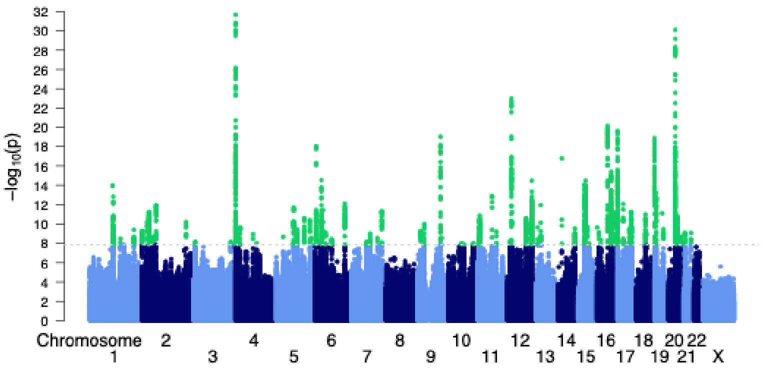

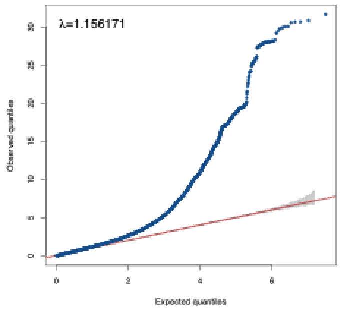

East Asian ancestry analysis

Osteoarthritis at any site (n=10,408 cases and 128,633 controls)

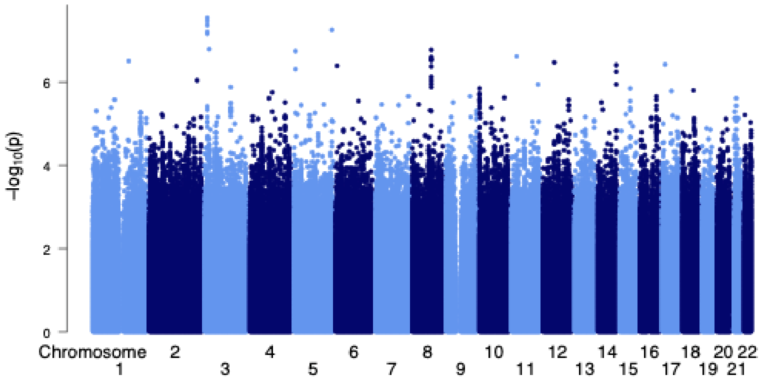

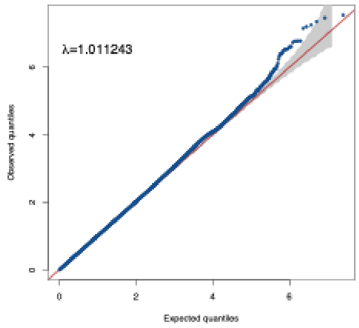

Hip and/or knee osteoarthritis (n=5,506 cases and 126,769 controls)

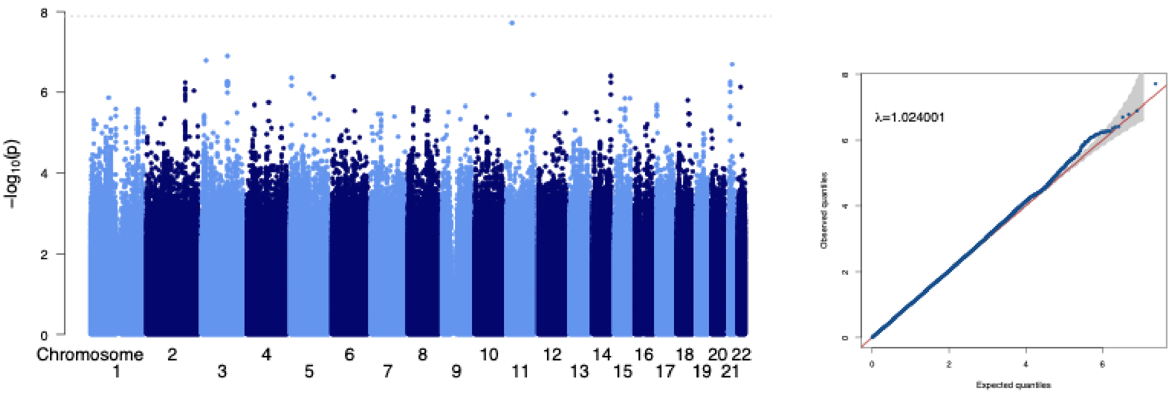

Hip osteoarthritis (n=65 cases and 2,619 controls)

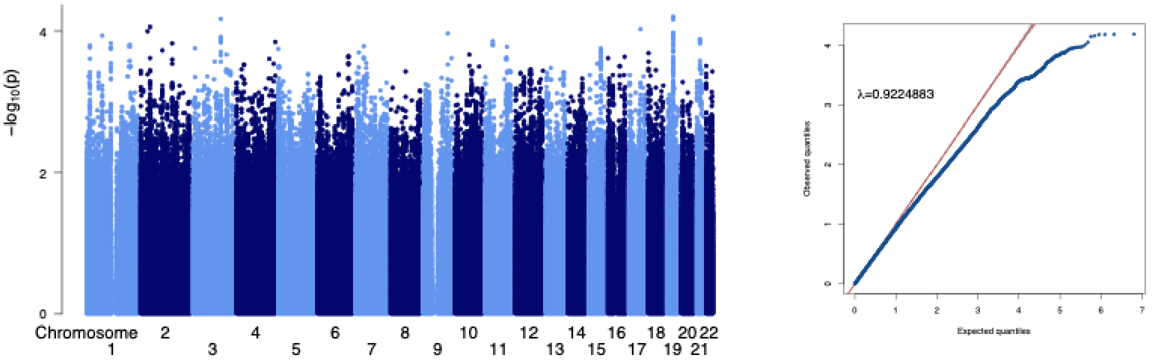

Knee osteoarthritis (n=5,433 cases and 126,769 controls)

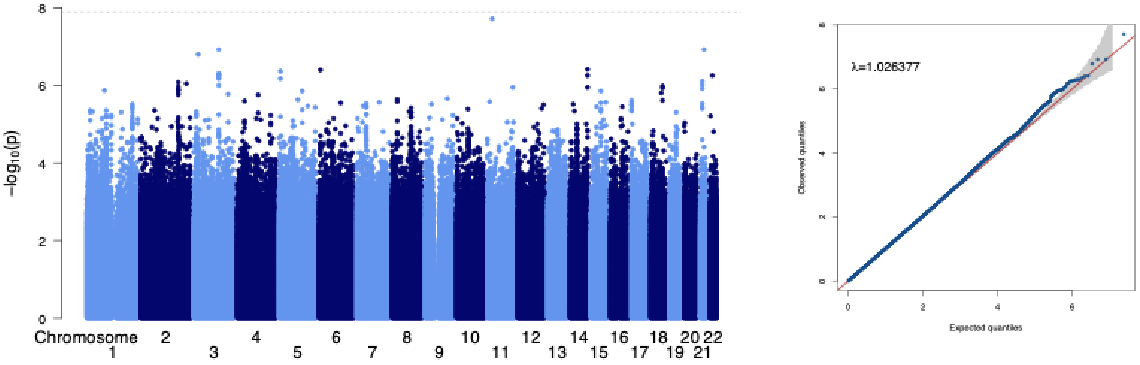

Spine osteoarthritis (n=3,799 cases and 70,581 controls)

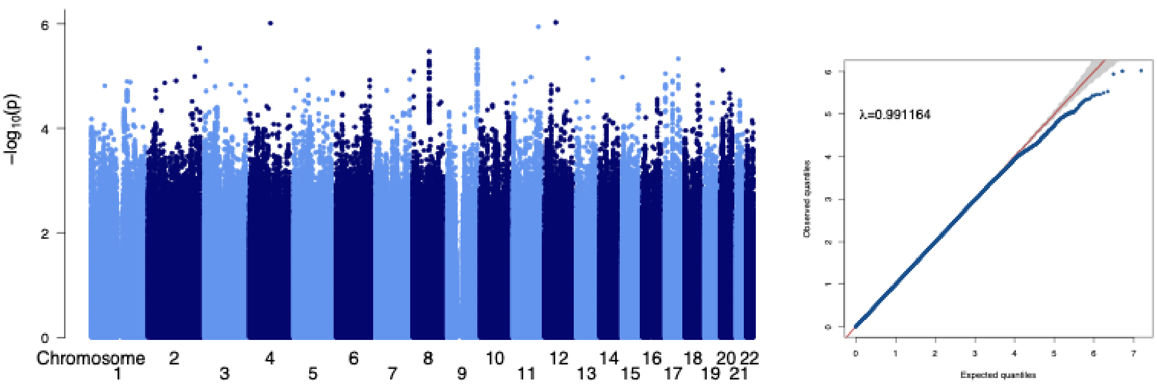

South Asian ancestry analysis

Osteoarthritis at any site (n=2,422 cases and18,894 controls)

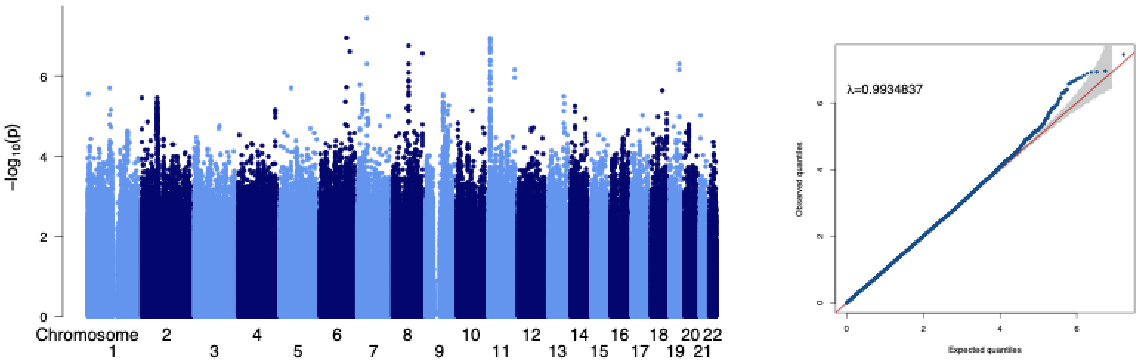

Hip and/or knee osteoarthritis (n=1,087 cases and 18,871 controls)

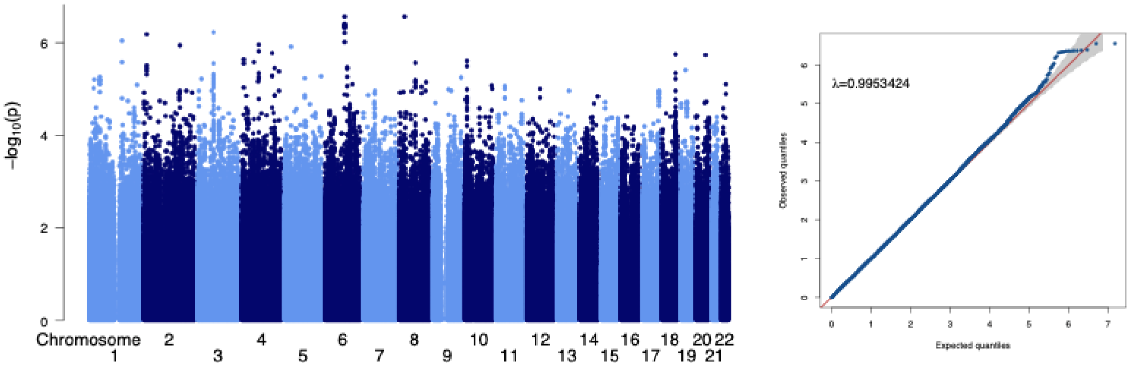

Knee osteoarthritis (n=993 cases and 18,871 controls)

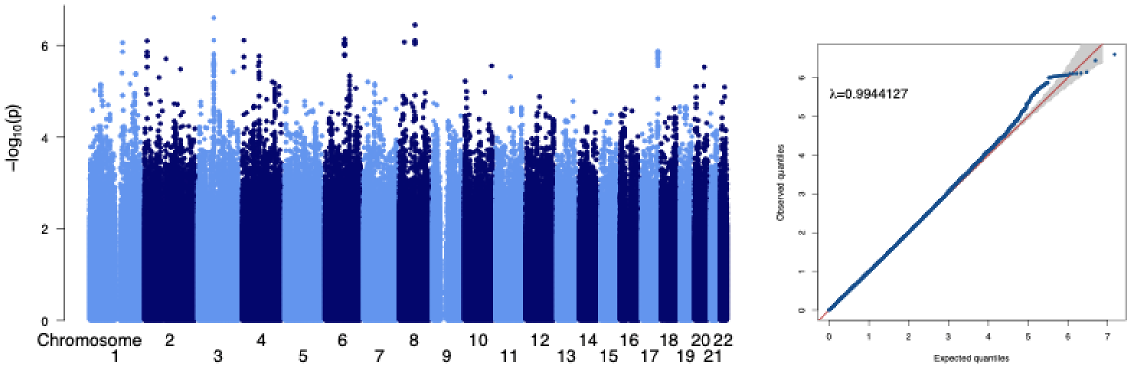

Spine osteoarthritis (n= 506 cases and 18,870 controls)

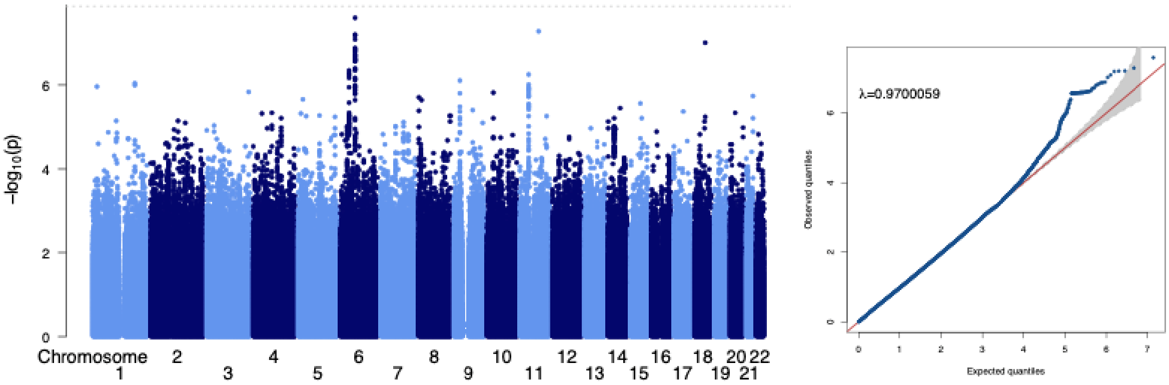

African ancestry analysis

Osteoarthritis at any site (n=25,698 cases and 34,718 controls)

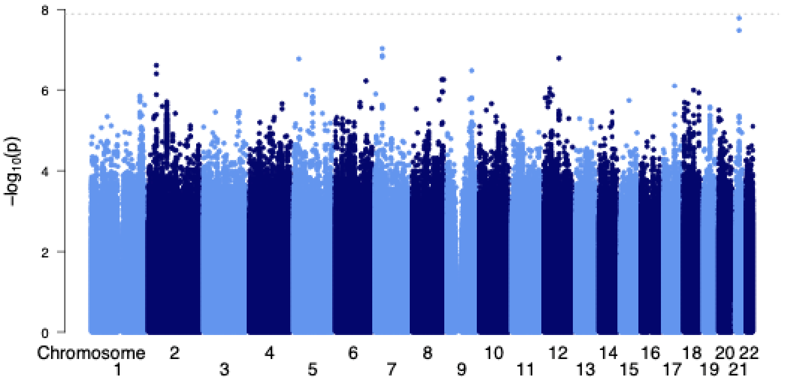

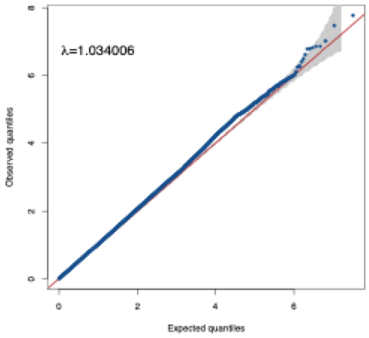

Hip and/or knee osteoarthritis (n=4,149 cases and 21,448 controls)

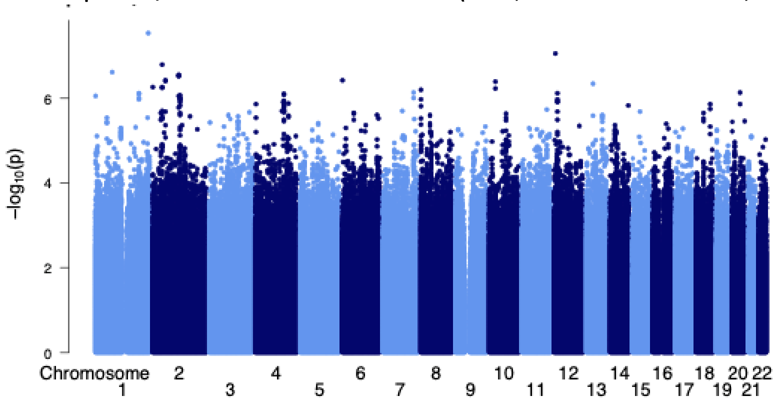

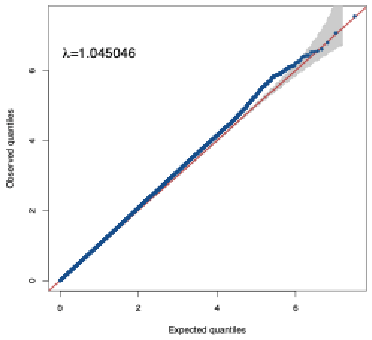

Hip osteoarthritis (n=1,357 cases and 21,394 controls)

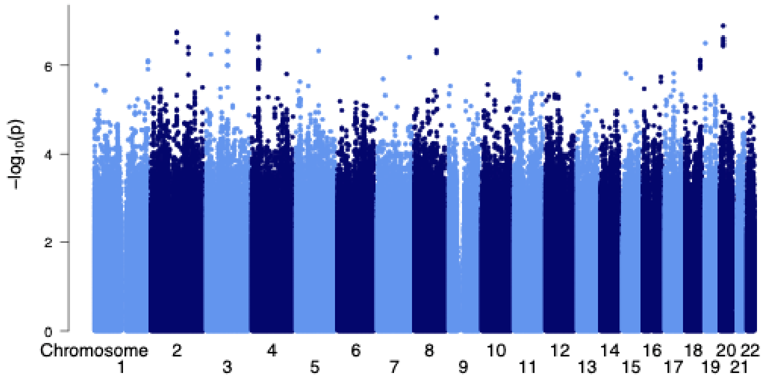

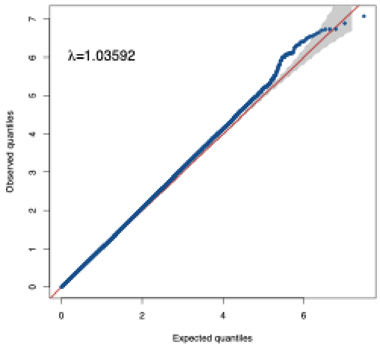

Knee osteoarthritis (n=3,463 cases and 21,448 controls)

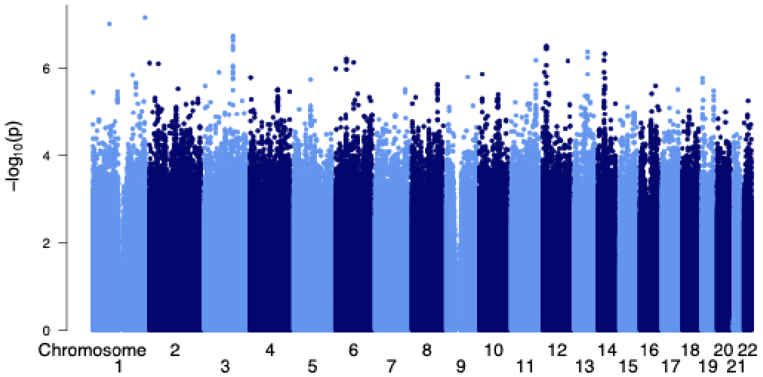

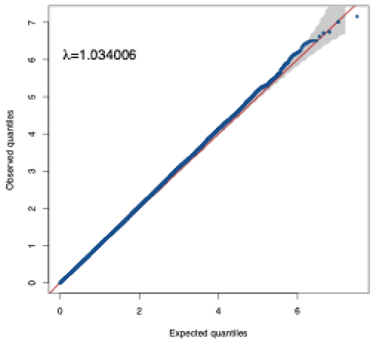

Hand osteoarthritis (n=949 cases and 14,129 controls)

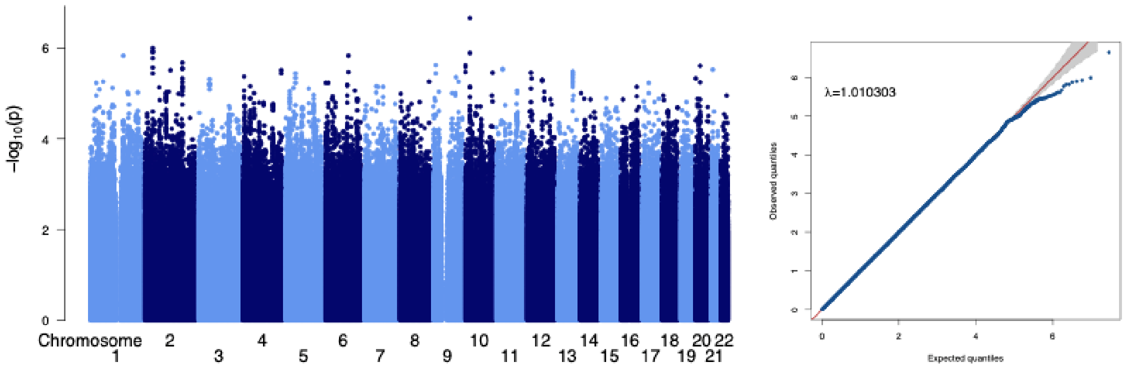

Finger osteoarthritis (n=253 cases and 8,523 controls)

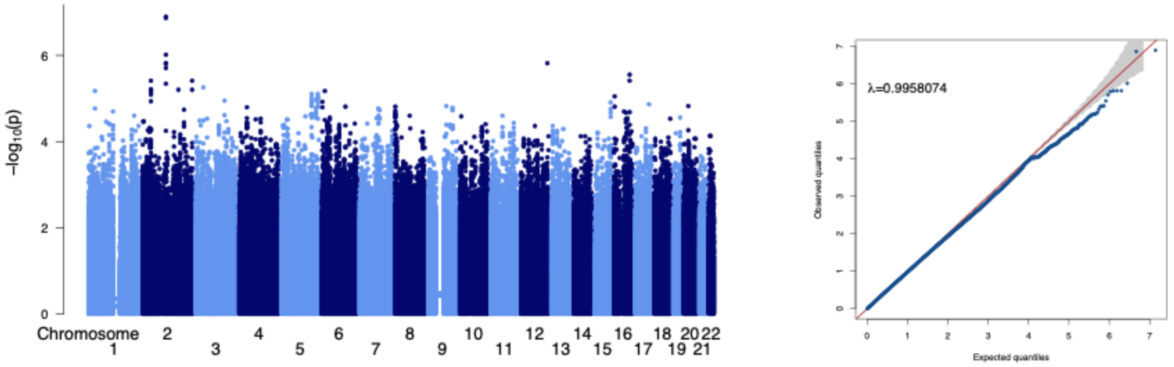

Thumb osteoarthritis (n=166 cases and 8,523 controls)

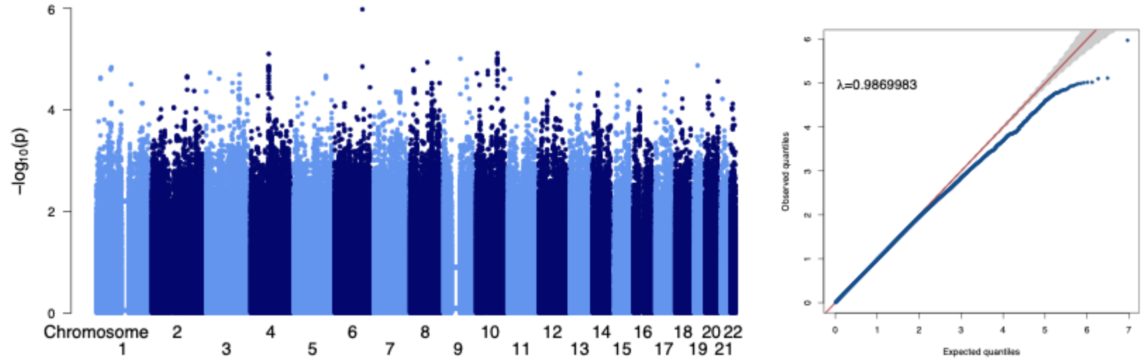

Spine osteoarthritis (n=1,973 cases and 21,394 controls)

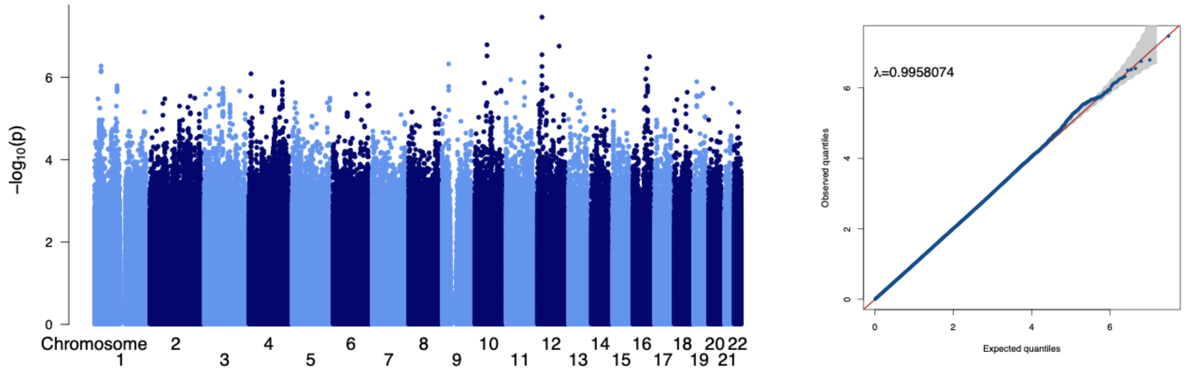

Hispanic ancestry analysis

Osteoarthritis at any site (n=8,128 cases and 9,759 controls)

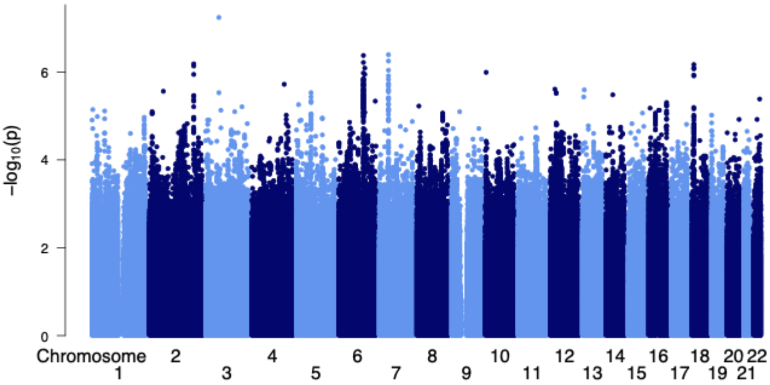

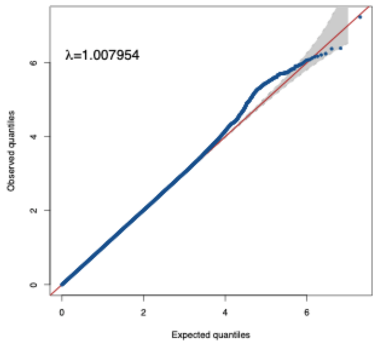

Hip and/or knee osteoarthritis (n=842 cases and 3,489 controls)

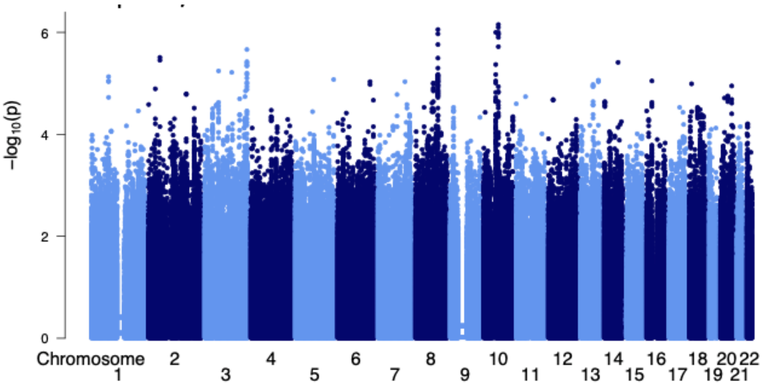

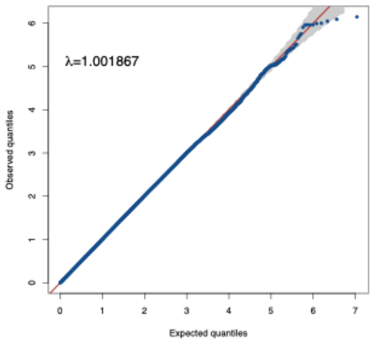

Hip osteoarthritis (n=288 cases and 3,486 controls)

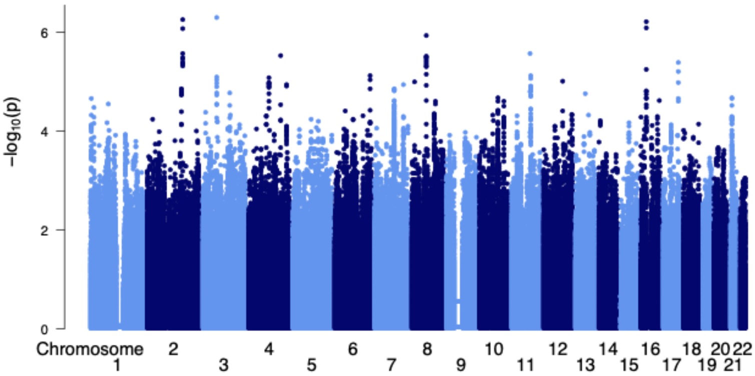

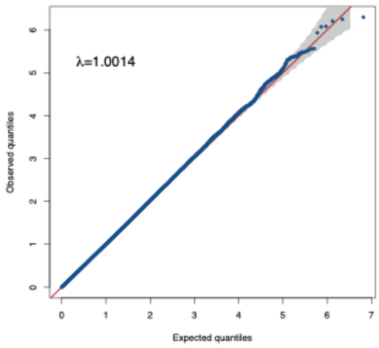

Knee osteoarthritis (n=523 cases and 2,903 controls)

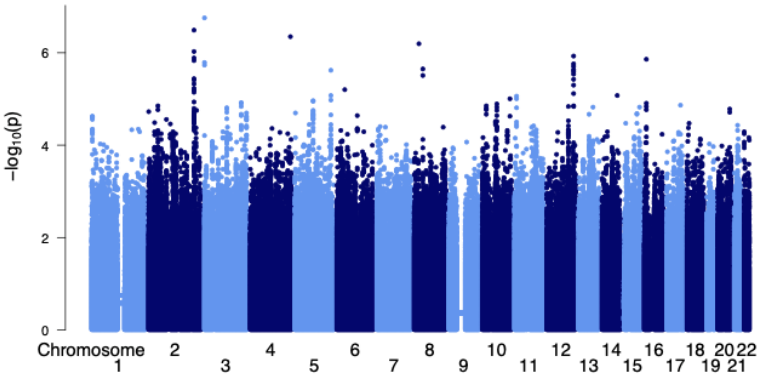

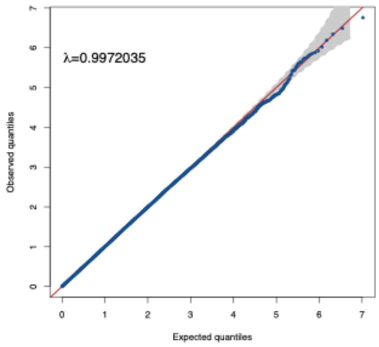

Hand osteoarthritis (n=242 cases and 3,471 controls)

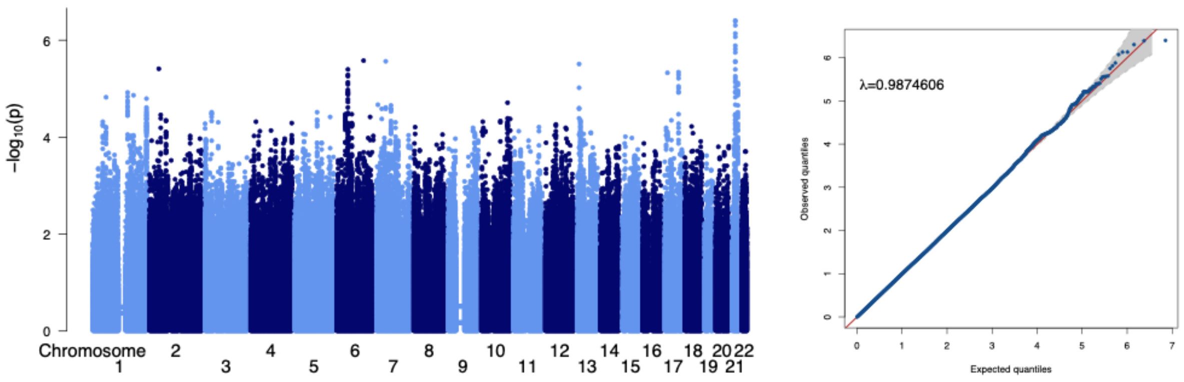

Sex-stratified analysis

Osteoarthritis at any site

Female n=199,234 cases and 588,852 controls

Male n=129,191 cases and 515,493 controls

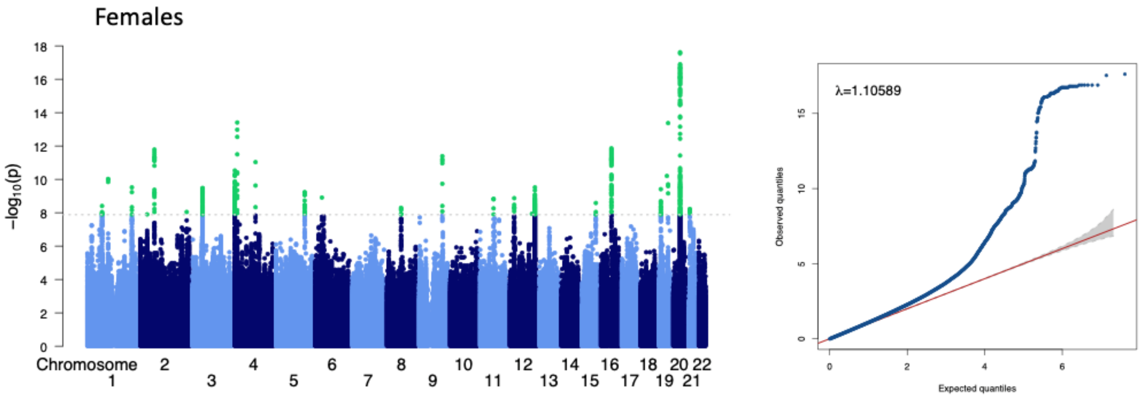

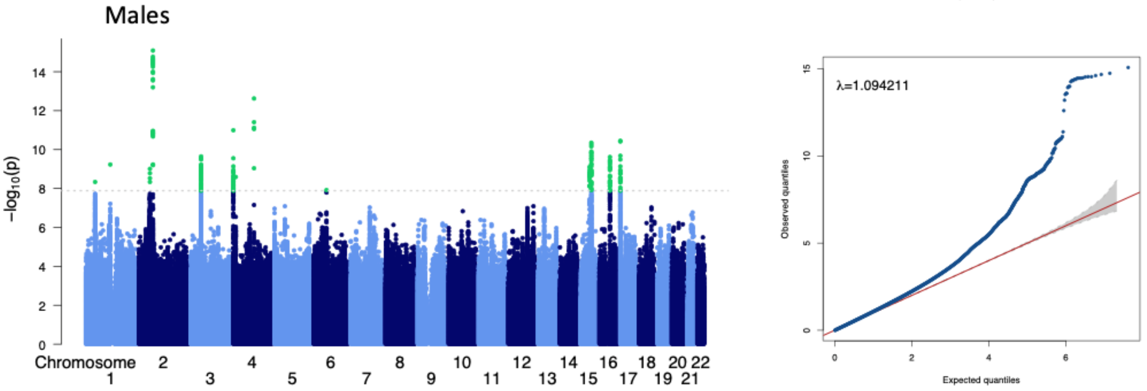

Hip and/or knee osteoarthritis
Female n=107,705 cases and 442,452 controls
Male n=72,281 cases and 380,585 controls

Females

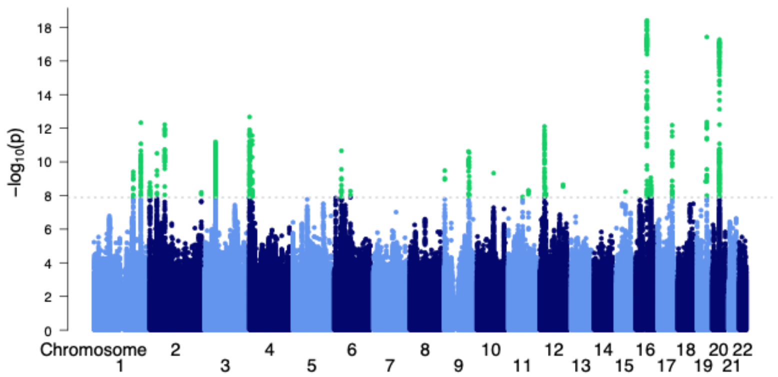

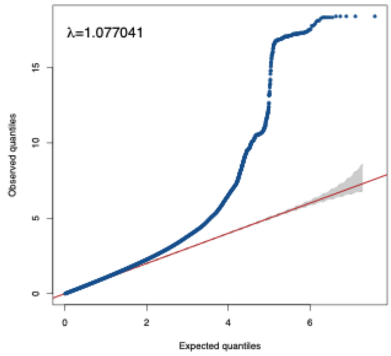

Males

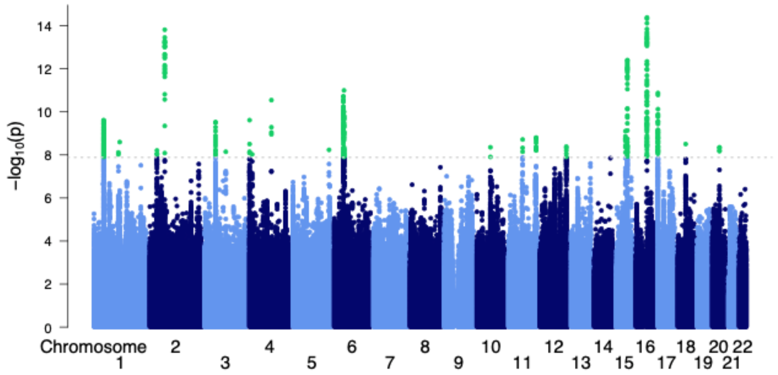

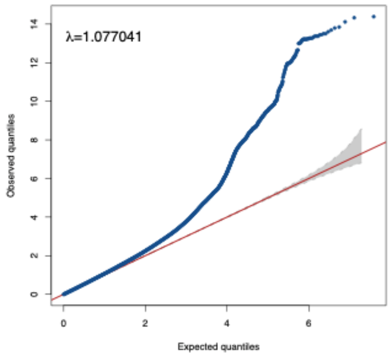

Hip osteoarthritis
Female n=45,562 cases and 413,714 controls
Male n=29,536 cases and 362,133 controls

Females

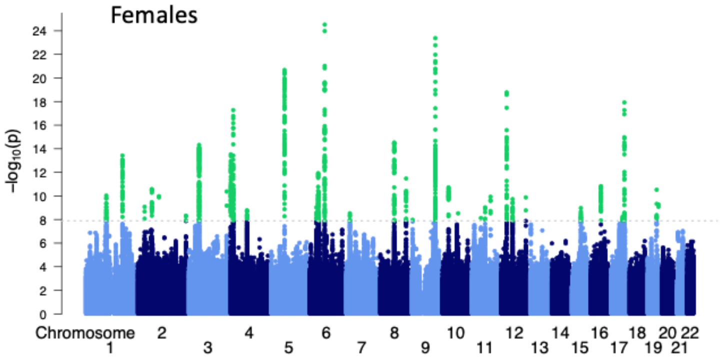

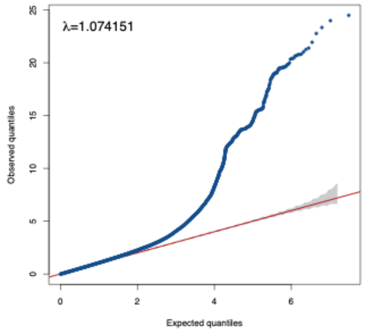

Males

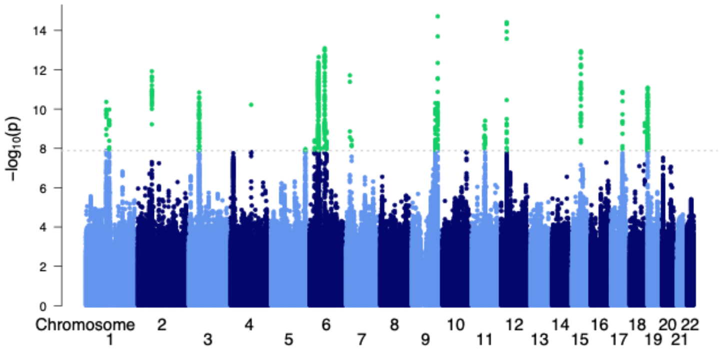

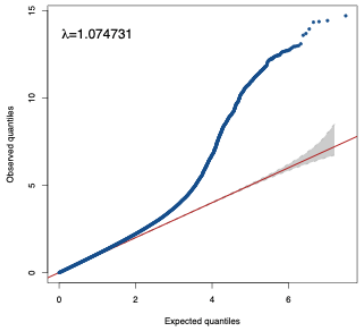

Knee osteoarthritis
Female n=75,727 cases and 410,599 controls
Male n=50,135 cases and 363,279 controls

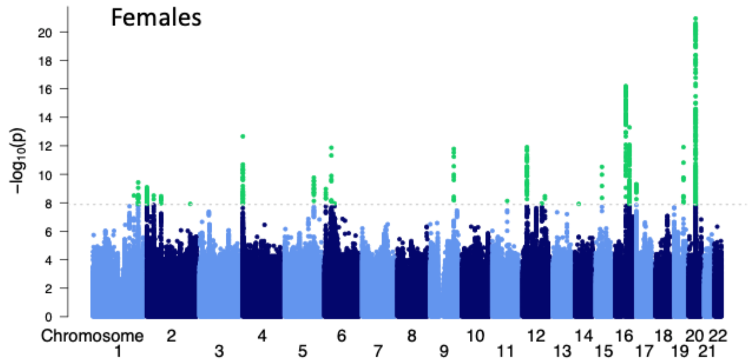

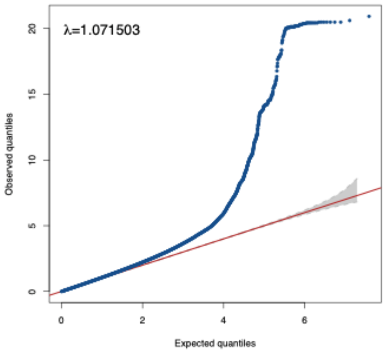

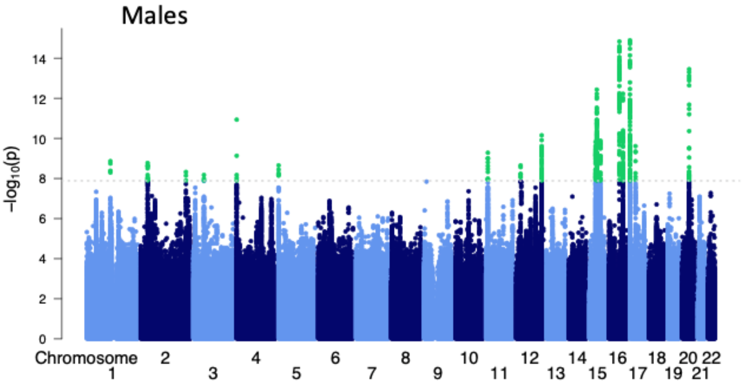

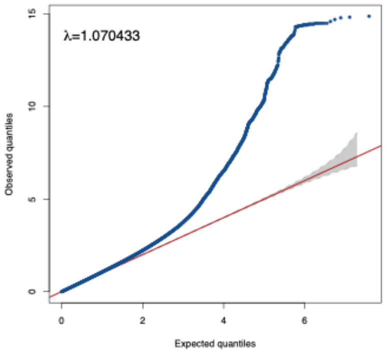

Hand osteoarthritis
Female n=27,339 cases and 401,542 controls
Male n=10,074 cases and 342,593 controls

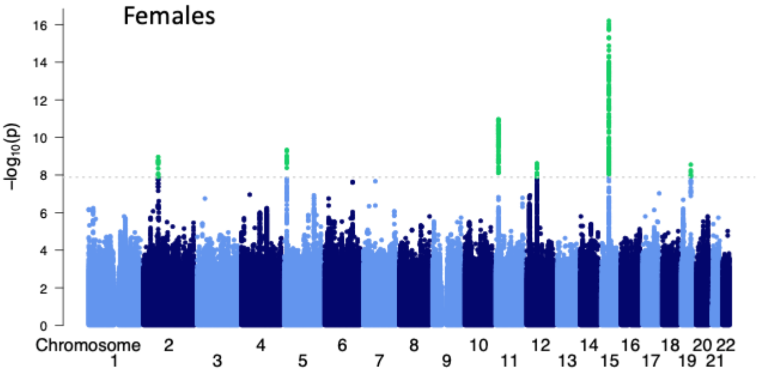

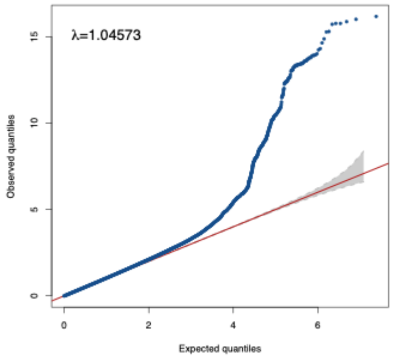

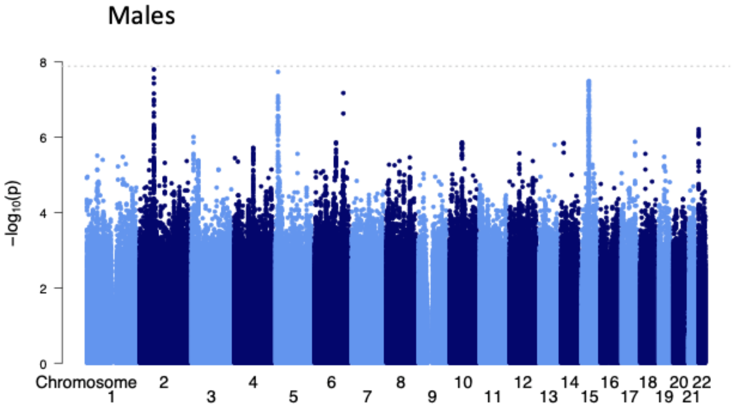

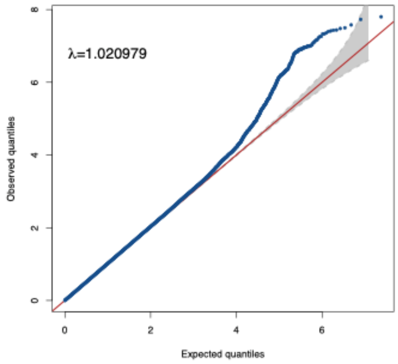

Finger osteoarthritis
Female n=10,655 cases and 338,289 controls
Male n=4,333 cases and 117,585 controls

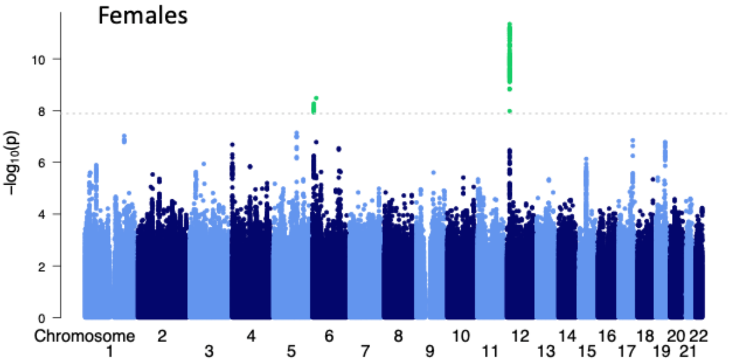

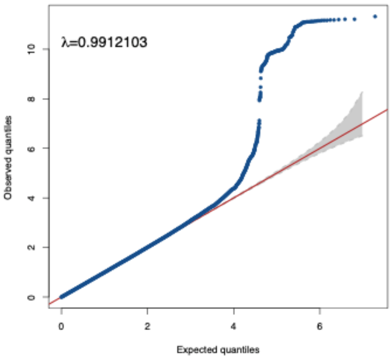

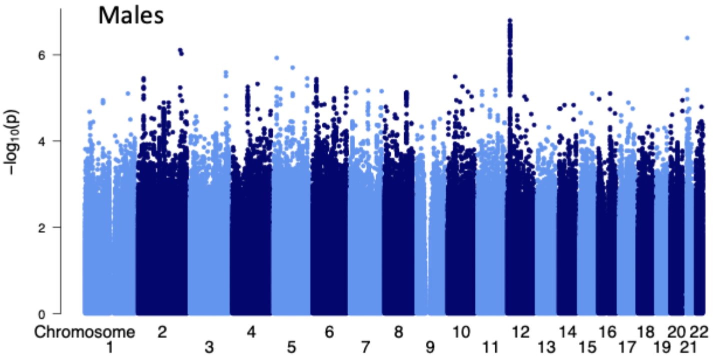

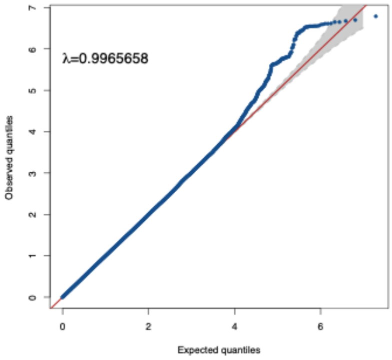

Thumb osteoarthritis
Female n=11,426 cases and 322,724 controls
Male n=3,742 cases and 315,372 controls

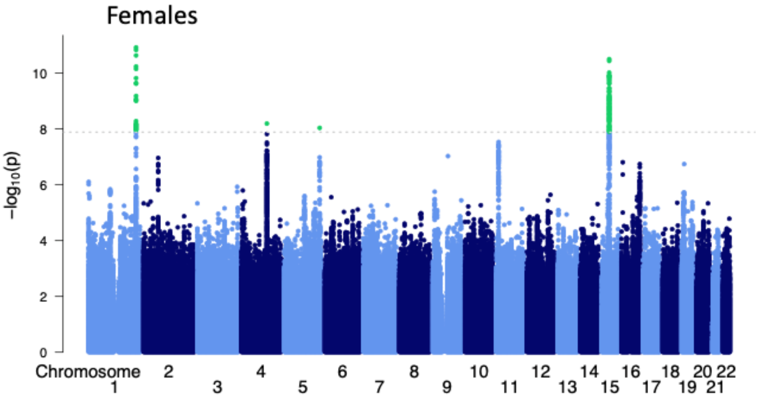

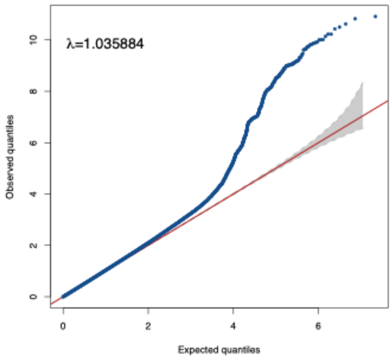

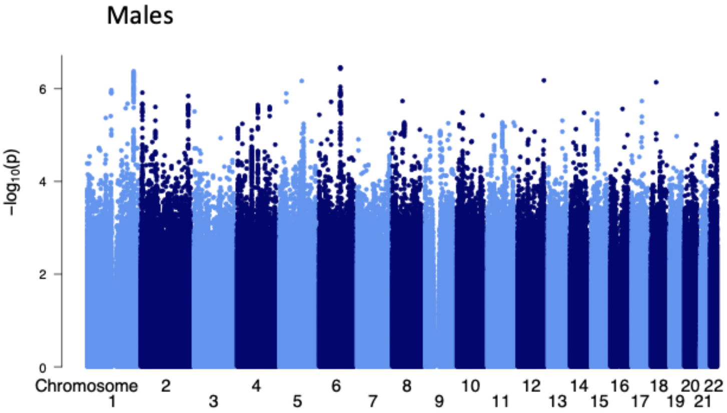

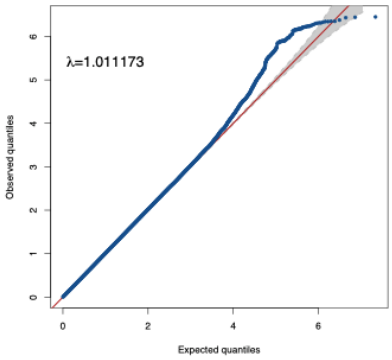

Spine osteoarthritis
Female n=41,963 cases and 423,373 controls
Male n=27,125 cases and 375,475 controls

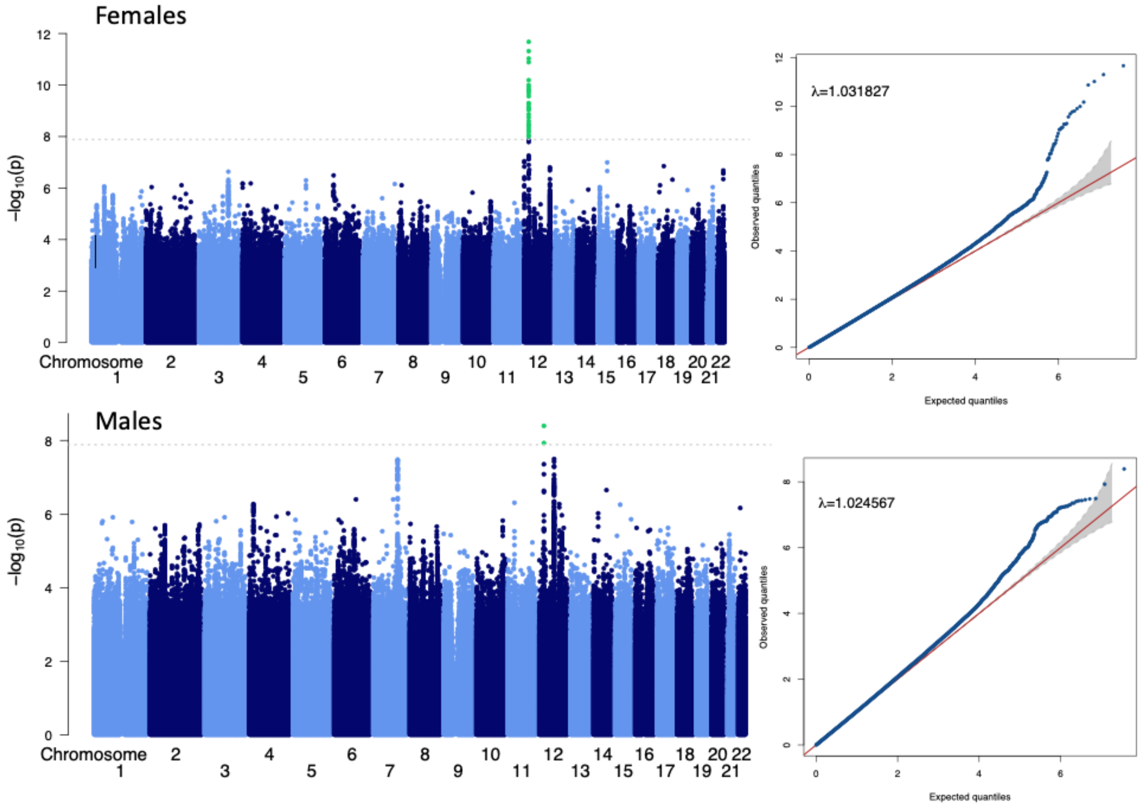

Total joint replacement
Female n=38,898 cases and 362,729 controls
Male n=26,488 cases and 309,220 controls

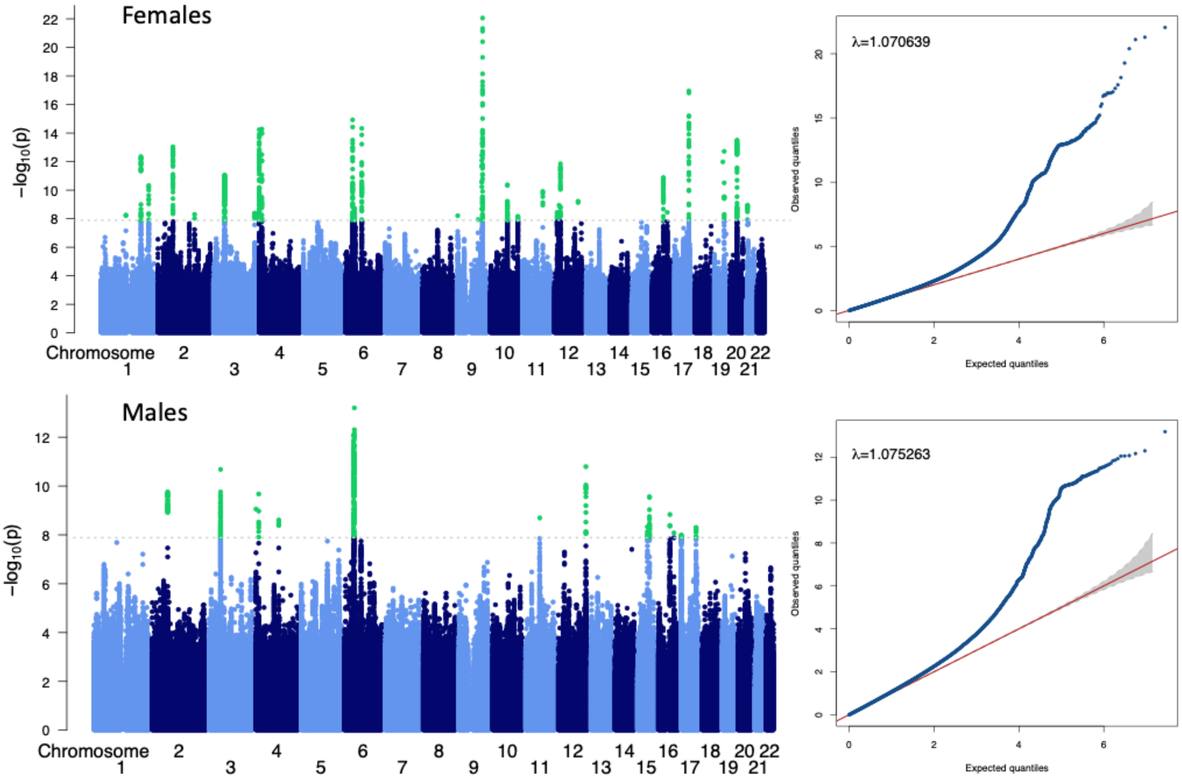

Total hip replacement

Female n=22,269 cases and 358,515 controls

Male n=14,048 cases and 302,489 controls

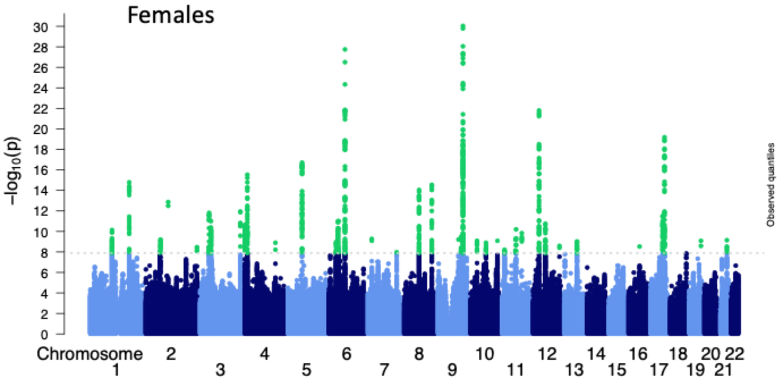

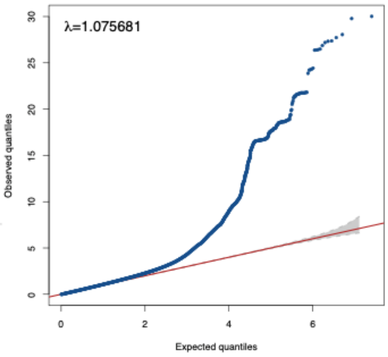

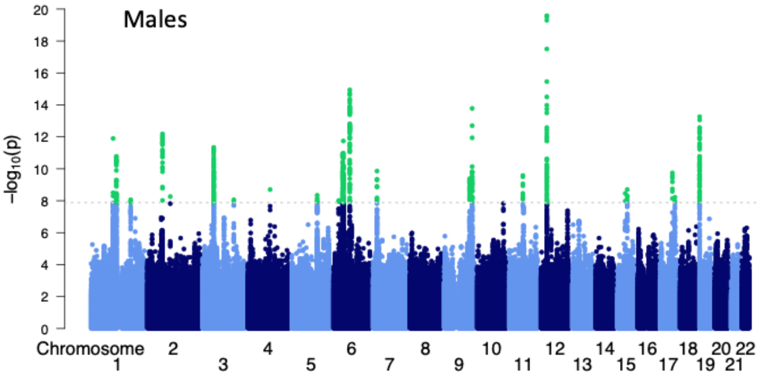

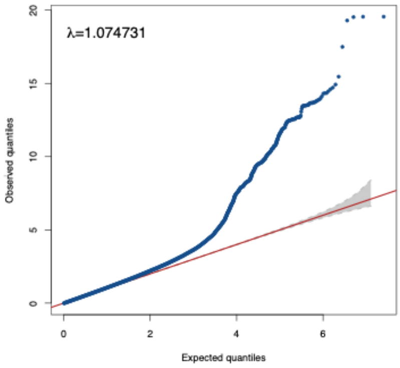

Total knee replacement

Female n=19,140 cases and 34,4258 controls

Male n=13,633 cases and 30,9914 controls

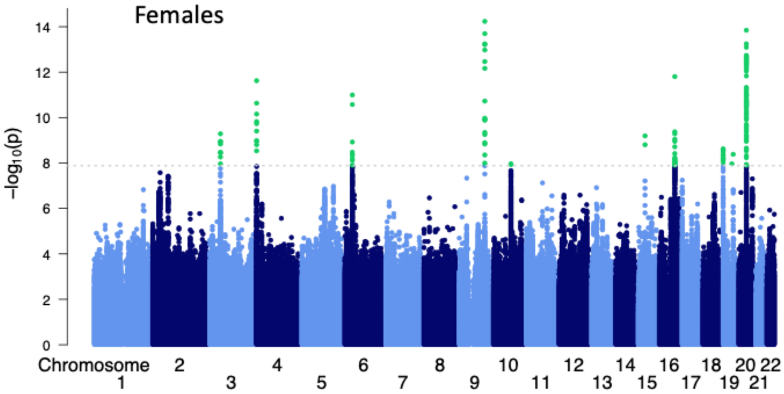

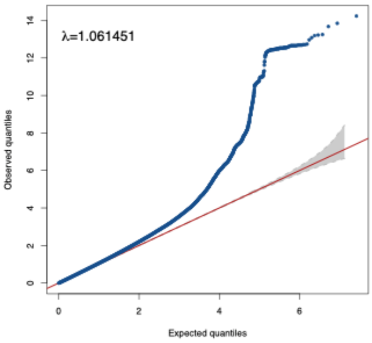

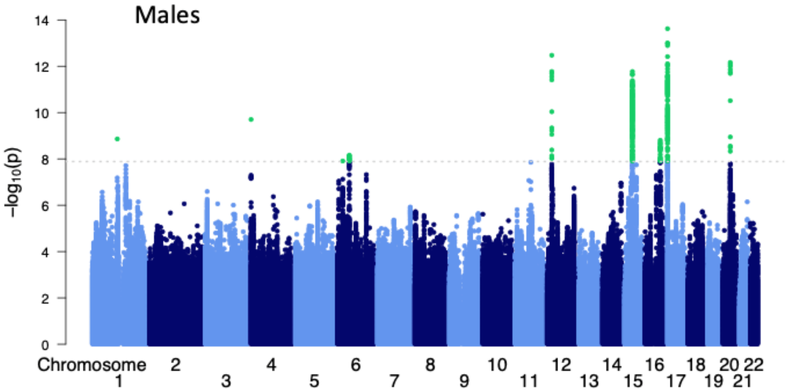

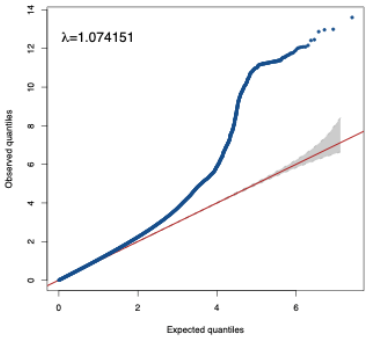

Each point represents a variant passing quality control in the meta-analysis, plotted with their association p-value (on a -log10 scale; y-axis) as a function of genomic position (NCBI build 37; x-axis). The dashed horizontal gray line indicates study-wide significance (-log10(1.3x10^{-8})). Only Manhattan and quantile-quantile plots with sufficient variants that pass all internal quality control are displayed here.

Supplementary Figure 3. Example of the quality control plots for GWAS datafiles prior to EasyQC.

A

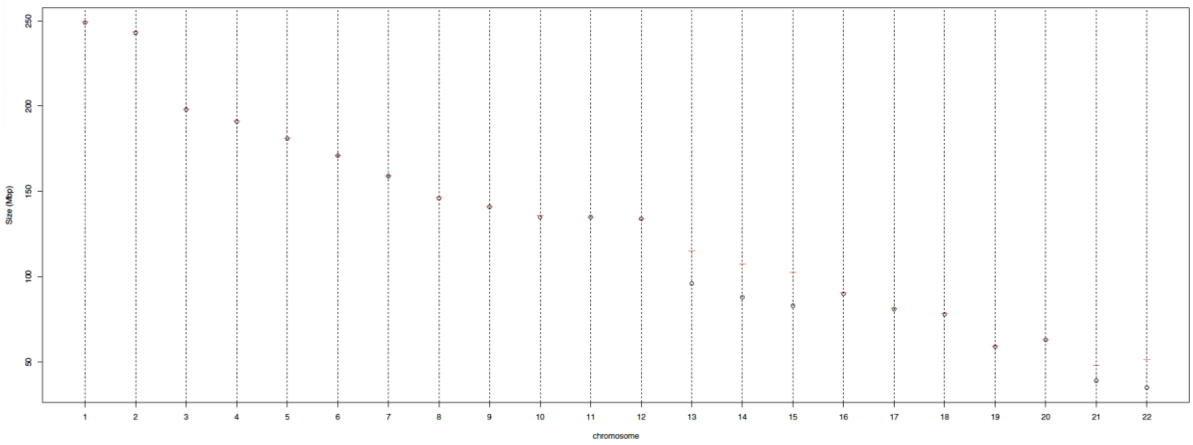

B

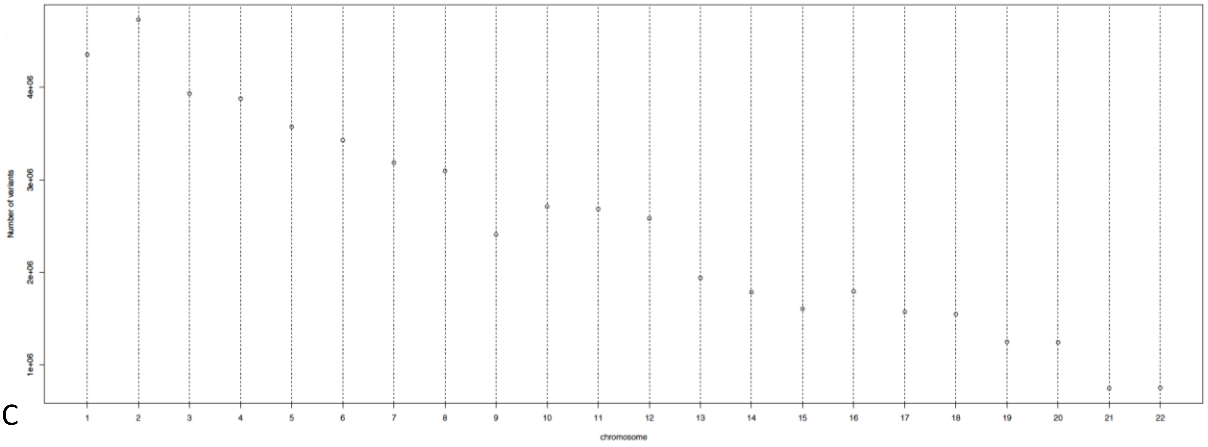

C

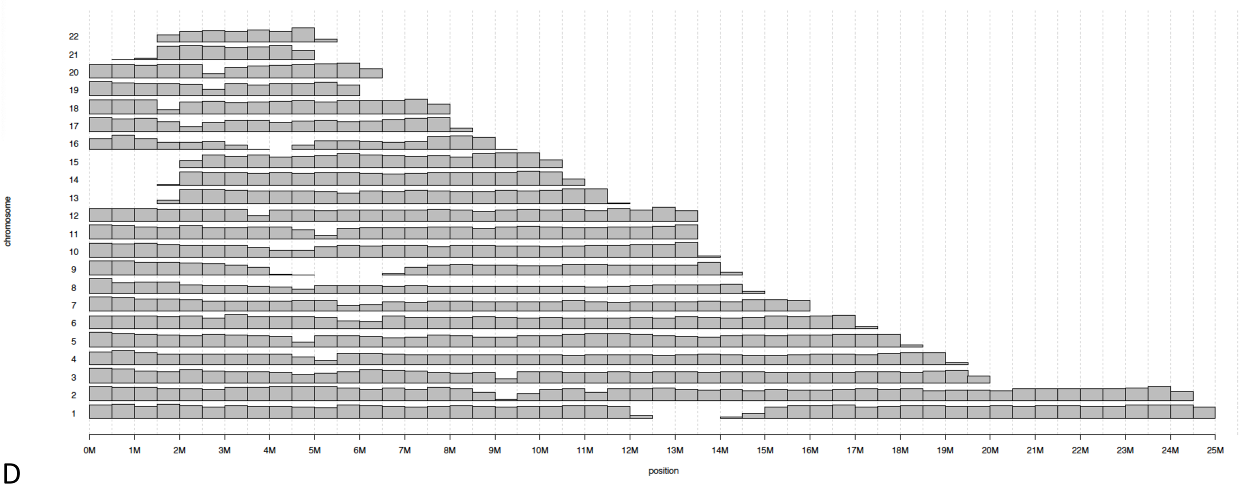

D

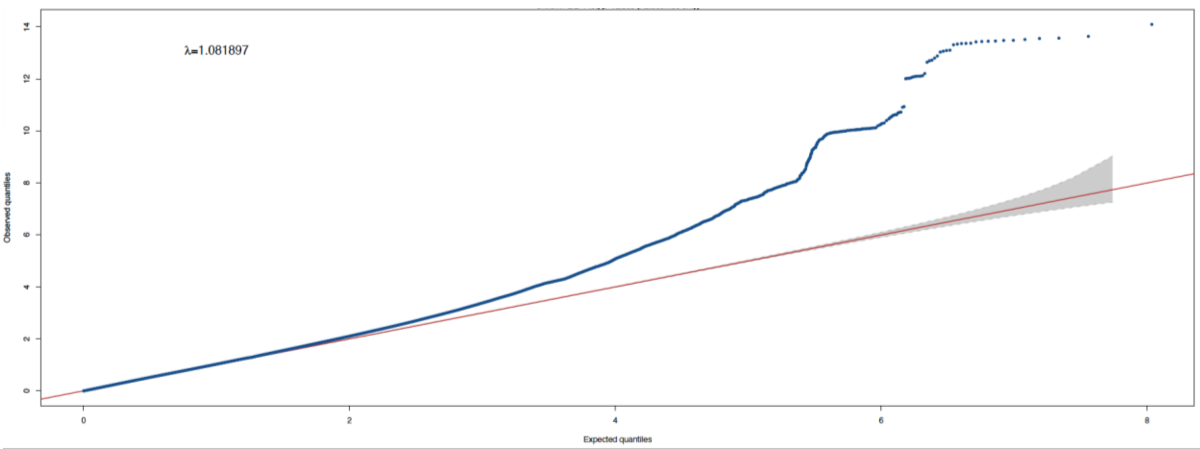

E

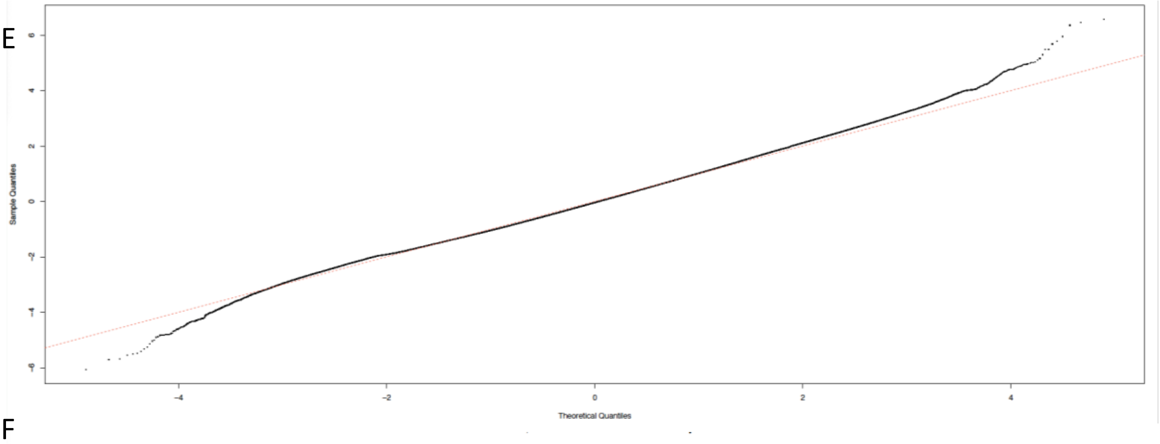

F

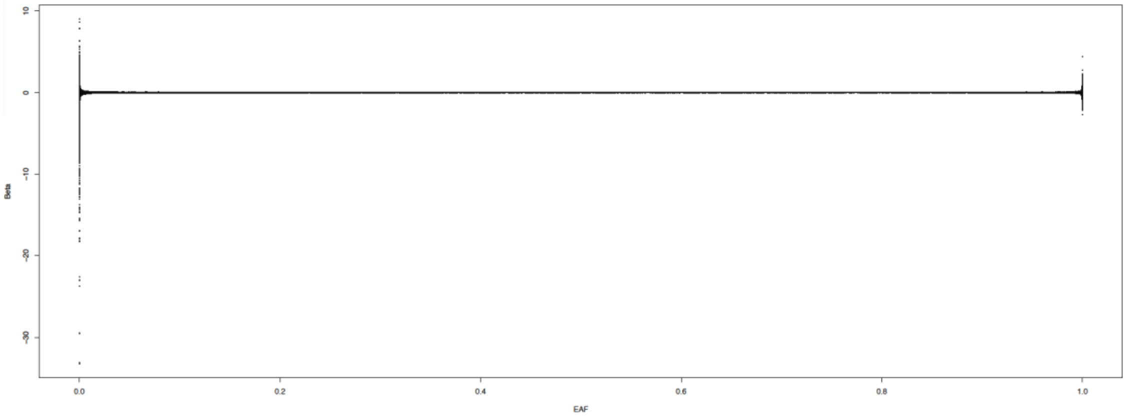

G

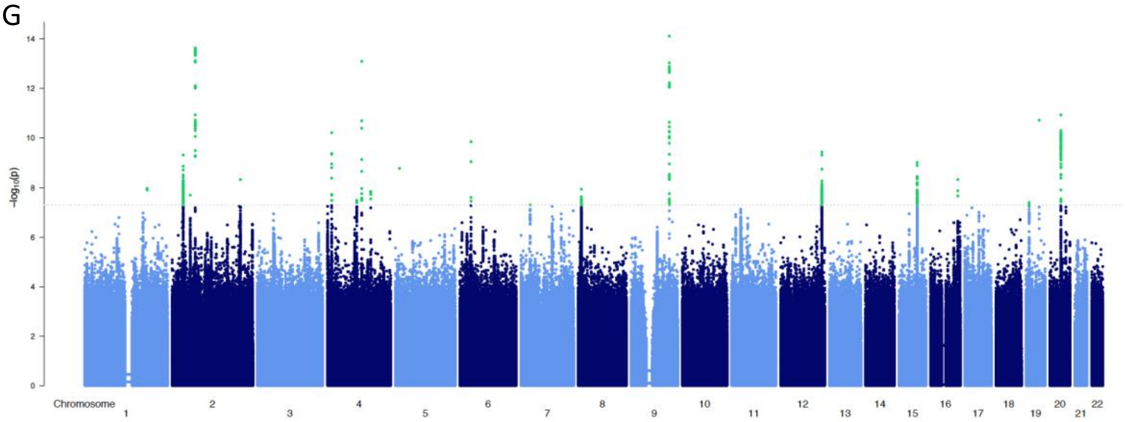

H

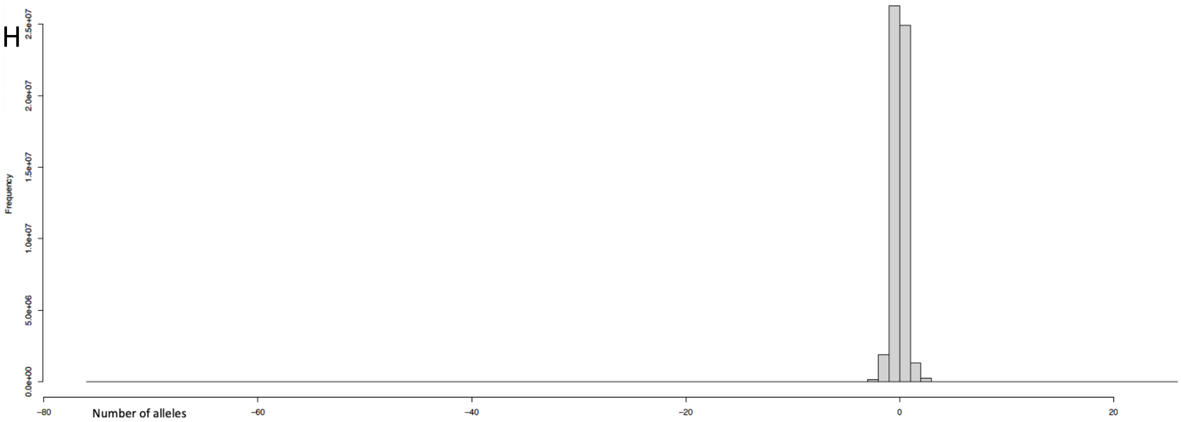

For each GWAS diagnostic plots were visualised using the PreQC script from here: https://github.com/hmgu-itg/go2-scripts. These include the following: (A) The chromosome (x-axis) span (y-axis) in the data, the red line indicates the expected length of each chromosome and the circle the number observed. (B) The number of variants (y-axis) in the file per chromosome (x-axis). (C) A coverage plot across the genome (chromosomes on y-axis and their position on the x-axis). (D) Quantile-quantile (QQ)-plot (uniform) for $-\log_{10}(P)$ with the expected values on the x-axis and the observed ones on the y-axis. (E) QQ-plot (normal) for beta/se. (G) Manhattan plot. The association p-value of the variants (on a -log10 scale) on the y-axis, as a function of genomic position (NCBI build 37; x-axis). (H) Histogram of the variant allele sizes (number of alleles on the x-axis and their frequency on the y-axis).

Supplementary Figure 4. Sensitivity analysis for osteoarthritis at any site for imaging-only datasets (n=6,816 cases and 9,624 controls). Beta and-$\log_{10}(P)$ comparisons for osteoarthritis at any site between the imaging-only meta-analysis and main meta-analysis.

A.

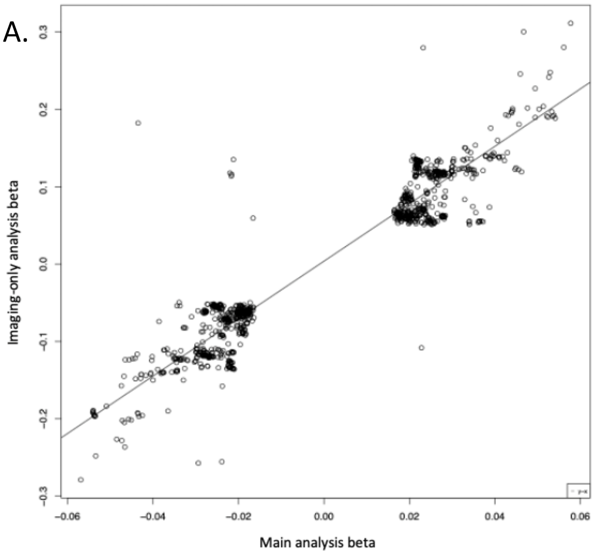

Pearson=0.948605
Spearman=0.865706

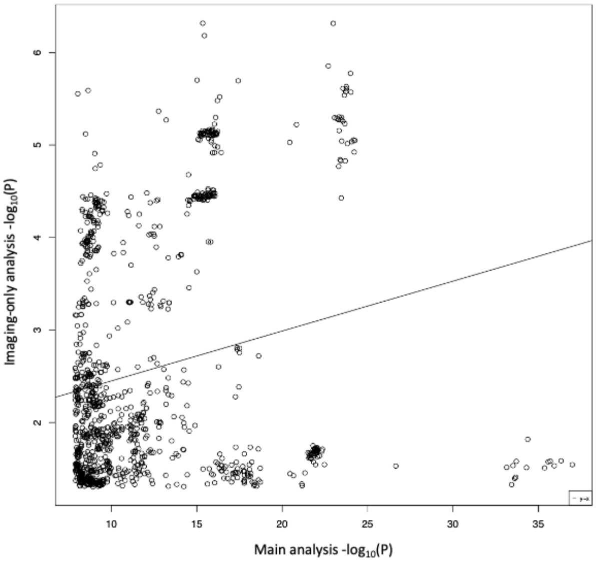

Pearson=0.2057966
Spearman=0.165216

B.

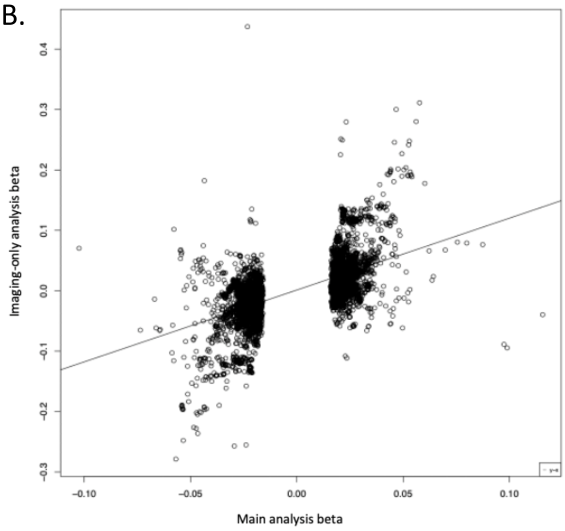

Pearson = 0.5933296
Spearman = 0.5844836

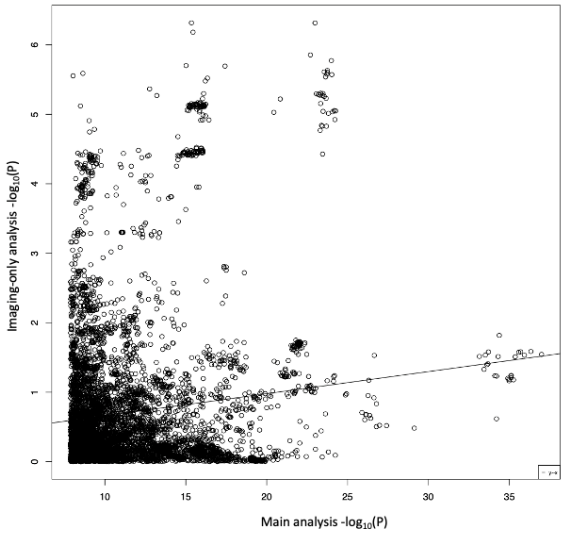

Pearson = 0.1182153
Spearman = -0.03765571

C.

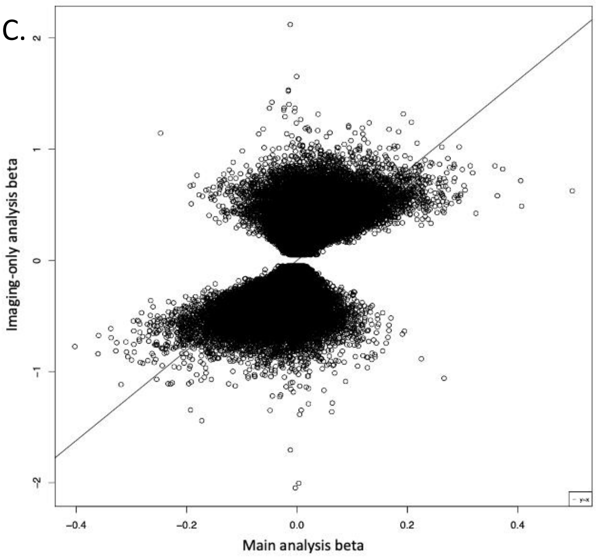

Pearson=0.430422
Spearman=0.3330855

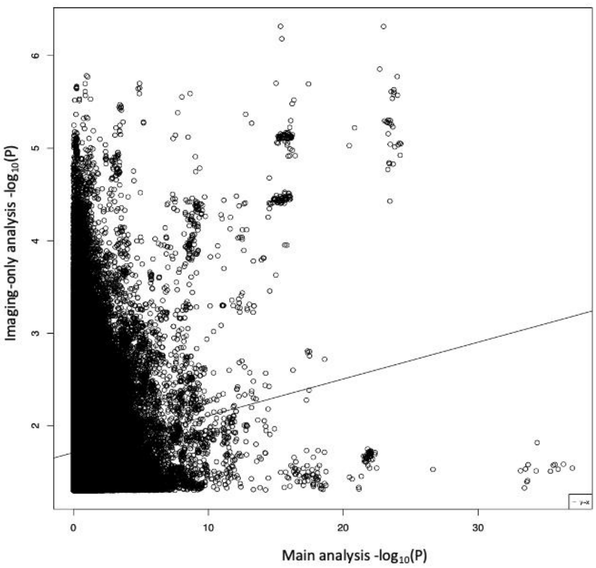

Pearson=0.09123026
Spearman=0.02786036

D.

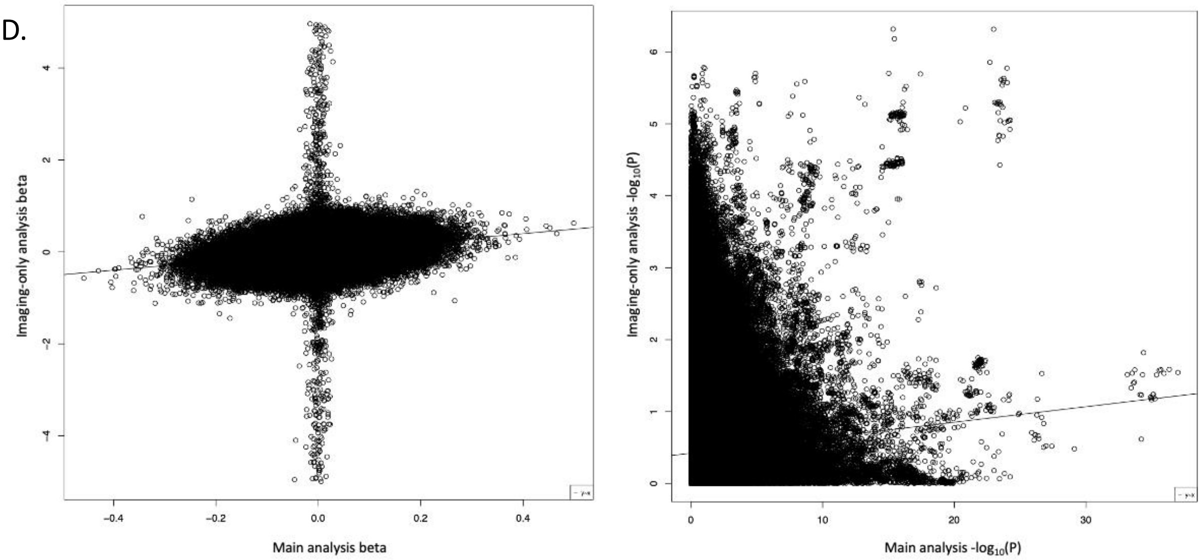

Pearson=0.2033918
Spearman=0.1386296

Pearson=0.03914701
Spearman=0.02253108

(A) Correlation of 1165 variants that are study-wide significant ($P < 1.3 \times 10^{-8}$) in the main meta-analysis and with suggestive significance ($P<0.05$) in the imaging-only meta-analysis. (B) Correlation of 7,803 variants study-wide significant ($P < 1.3 \times 10^{-8}$) in the main meta-analysis. (C) Correlation of 446,469 variants with suggestive significance ($P<0.05$) in the imaging-only meta-analysis. 4 variants are excluded from the imaging-only meta-analysis with beta > 10 or < -10 (for visualisation purposes) (D). Correlation of all 8,906,420 overlapping variants with the exclusion of variants in the imaging-only meta-analysis with beta > 5 or < -5 (for visualisation purposes). The black correlation line represents y~x.

Supplementary Figure 5. Sensitivity analysis for osteoarthritis at any site for datasets without any self-reported definitions included (n=309,125 cases and 793,538 controls). Manhattan and quantile-quantile plots for osteoarthritis at any site for the meta-analysis without self-reported osteoarthritis. Beta and-log10(P) comparisons for osteoarthritis at any site between the meta-analysis without self-reported osteoarthritis and the main meta-analysis.

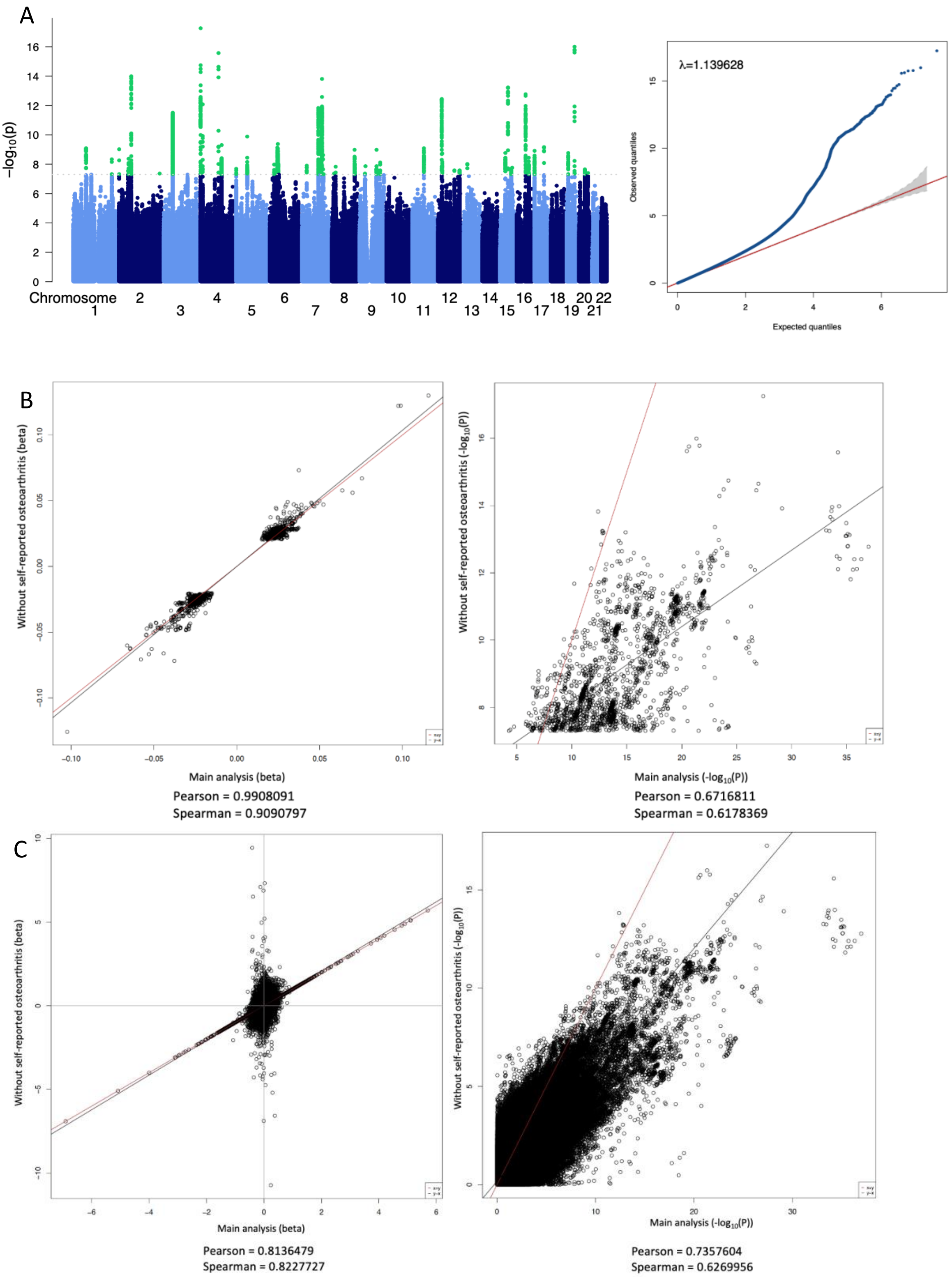

(A) Manhattan and quantile-quantile plots for the osteoarthritis at any site meta-analysis excluding individuals with self-reported osteoarthritis. The horizontal dashed grey line indicates study-wide significance (*P* value ≤ 1.3×10^{-8}). (B) Correlation of 1,977 variants that are genome-wide significant ($P < 5 \times 10^{-8}$) for osteoarthritis at any site between the meta-analysis without self-reported osteoarthritis and the main meta-analysis. (C) Correlation of all 20,571,594 overlapping variants for osteoarthritis at any site between the meta-analysis without self-reported osteoarthritis and the main meta-analysis. In (B) and (C) the Pearson and Spearman correlations are provided below each plot. The red correlation line indicates y=x and the black correlation line indicates y~x.

Supplementary Figure 6. Osteoarthritis at any site GWAS in UK Biobank (UKBB): Sensitivity analysis comparing the main UKBB GWAS analysis to UKBB without self-reported definition included.

A

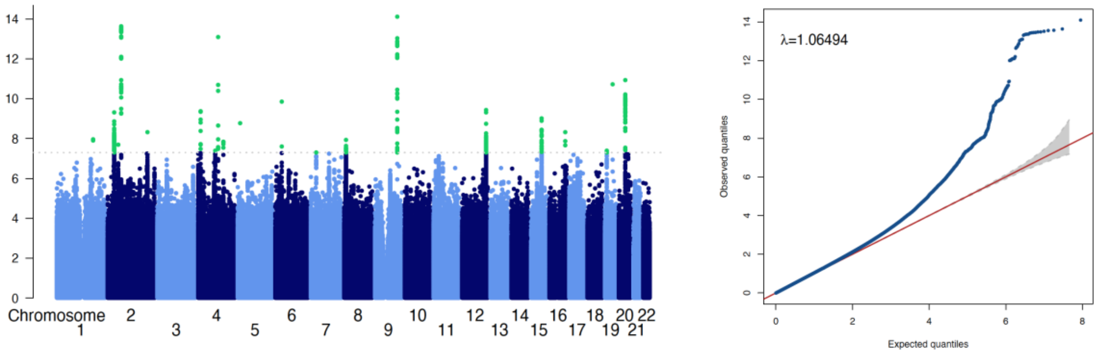

B

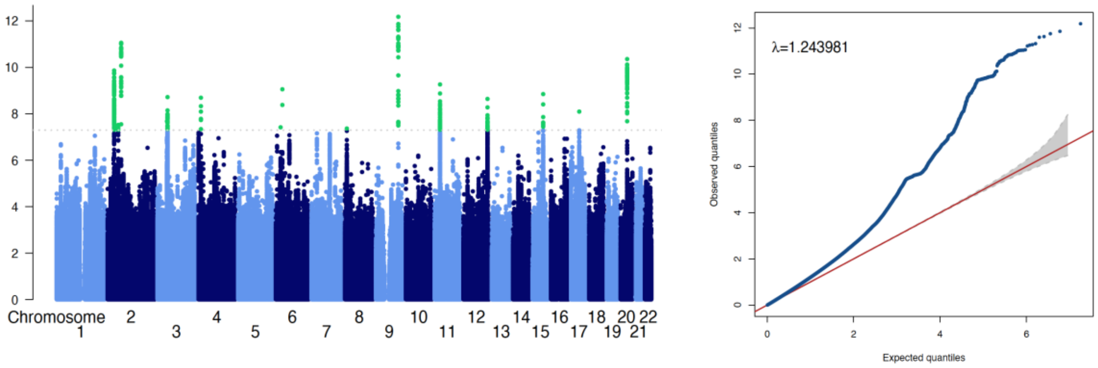

C

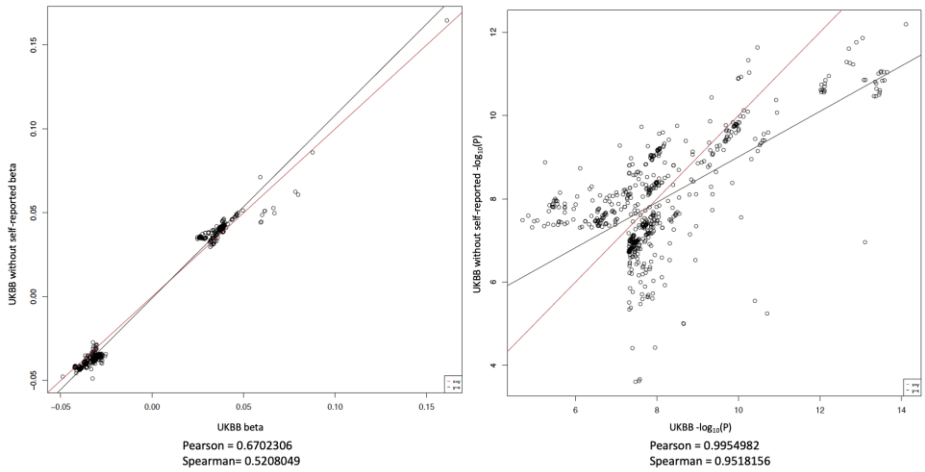

D

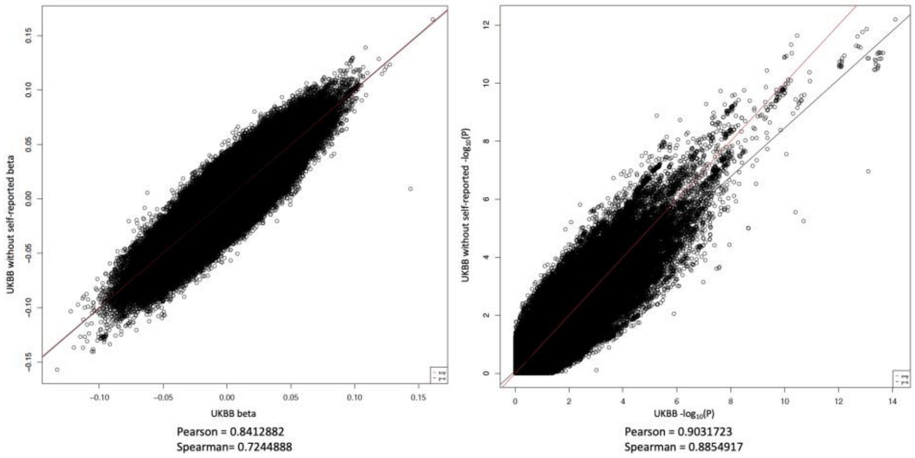

Manhattan and quantile-quantile (QQ) plot in UK Biobank for osteoarthritis at any site. The dashed grey line represents genome-wide significance (P value = 5 x 10^{-8}) (A) Main analysis, including all cases (n=82,420 individuals) (B) Excluding self-reported cases (n=19,885 individuals). The number of controls remained the same between the two analyses (n=323,032). GWAS correlation of beta and -$\log_{10}$(P values) for osteoarthritis at any site in UK Biobank between main analysis and without self-reported cases. (C) Includes variants that are genome-wide significant (P value < 5 x 10^{-8}) in either GWAS (n = 621). (D) Includes all overlapping variants (n = 7,638,131). The red correlation line indicates y=x and the black correlation line indicates y~x.

Supplementary Figure 7. Significant enrichment altered Transcription Factor binding Motifs.

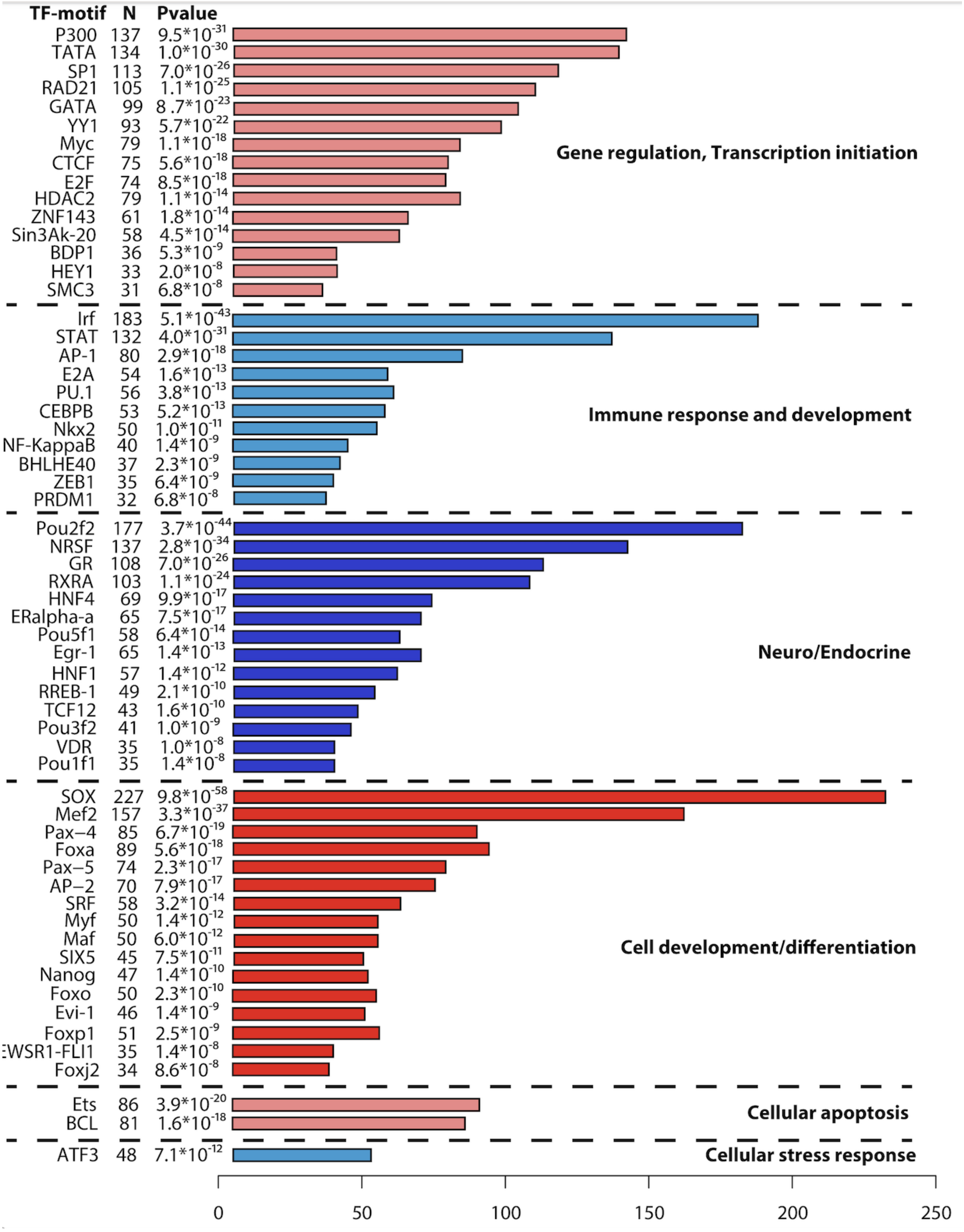

The bars indicate the number of times this TF-motif was found to be altered by one of the 95% credible set variants located in osteoblast/chondrogenic regulatory regions. The TF-motif are grouped based on the general function of the matching transcription factor. TF-Motif: significantly enriched altered transcription factor binding motif; N= the number of times this TF-motif was found to be altered by one of the 95% credible set variants located in osteoblast/chondrogenic regulatory regions. P-value: the P-value of the propensity test for enrichment.

Supplementary Figure 8. Bar plot illustrating the sensitivity of the 24 lines evidence for known osteoarthritis genes pre and post Boer, Hatzikotoulas, Southam *et al*. 2021.

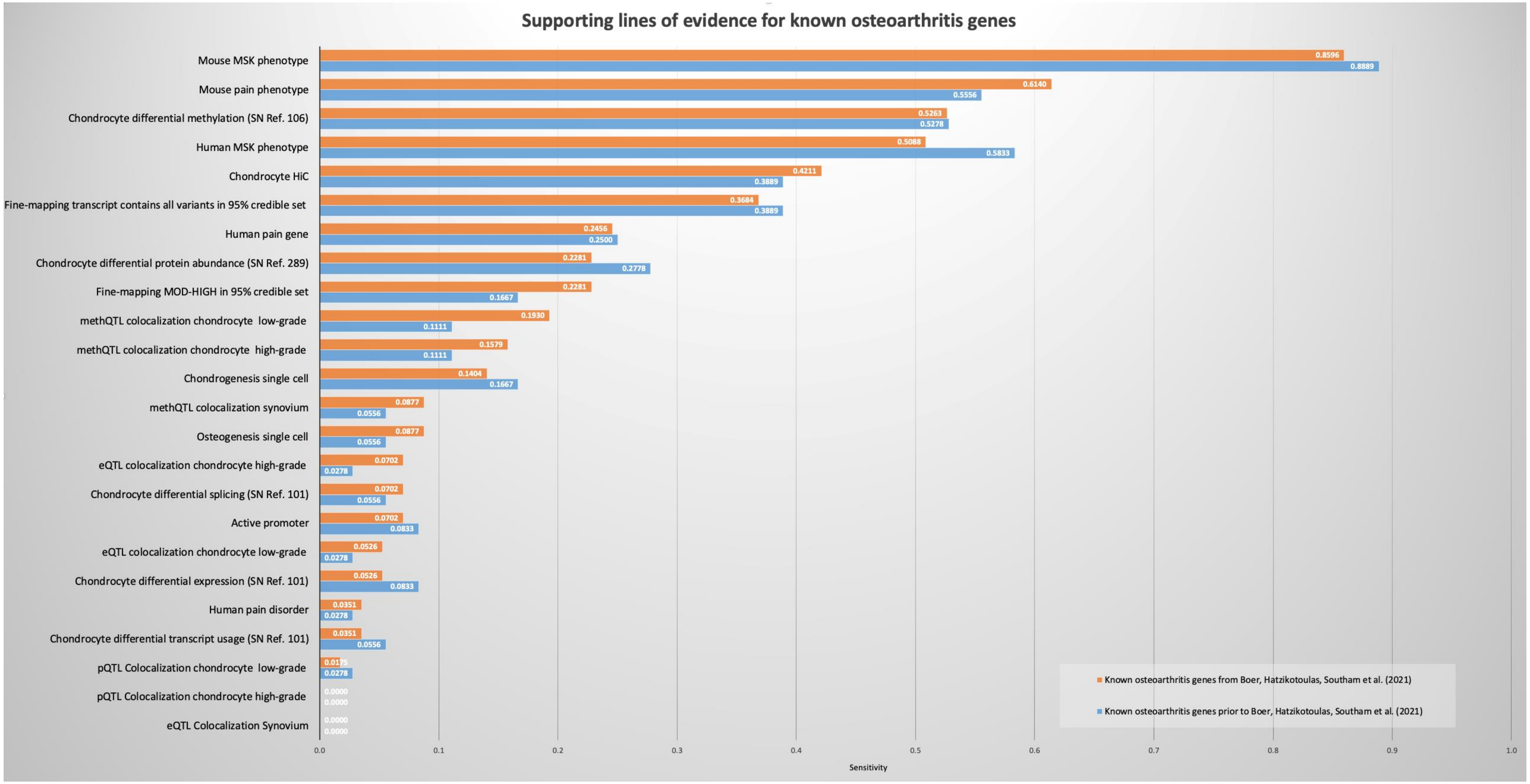

The x-axis indicates the sensitivity results. The y-axis indicate the supporting lines of evidence used for establishing effector genes for 57 known genes from Boer, Hatzikotoulas, Southam *et al.* (orange bars) and 36 know osteoarthritis genes prior to Boer, Hatzikotoulas, Southam *et al.* (blue bars).

Supplementary Figure 9. Genetic heritability for each of the 8 biological processes.

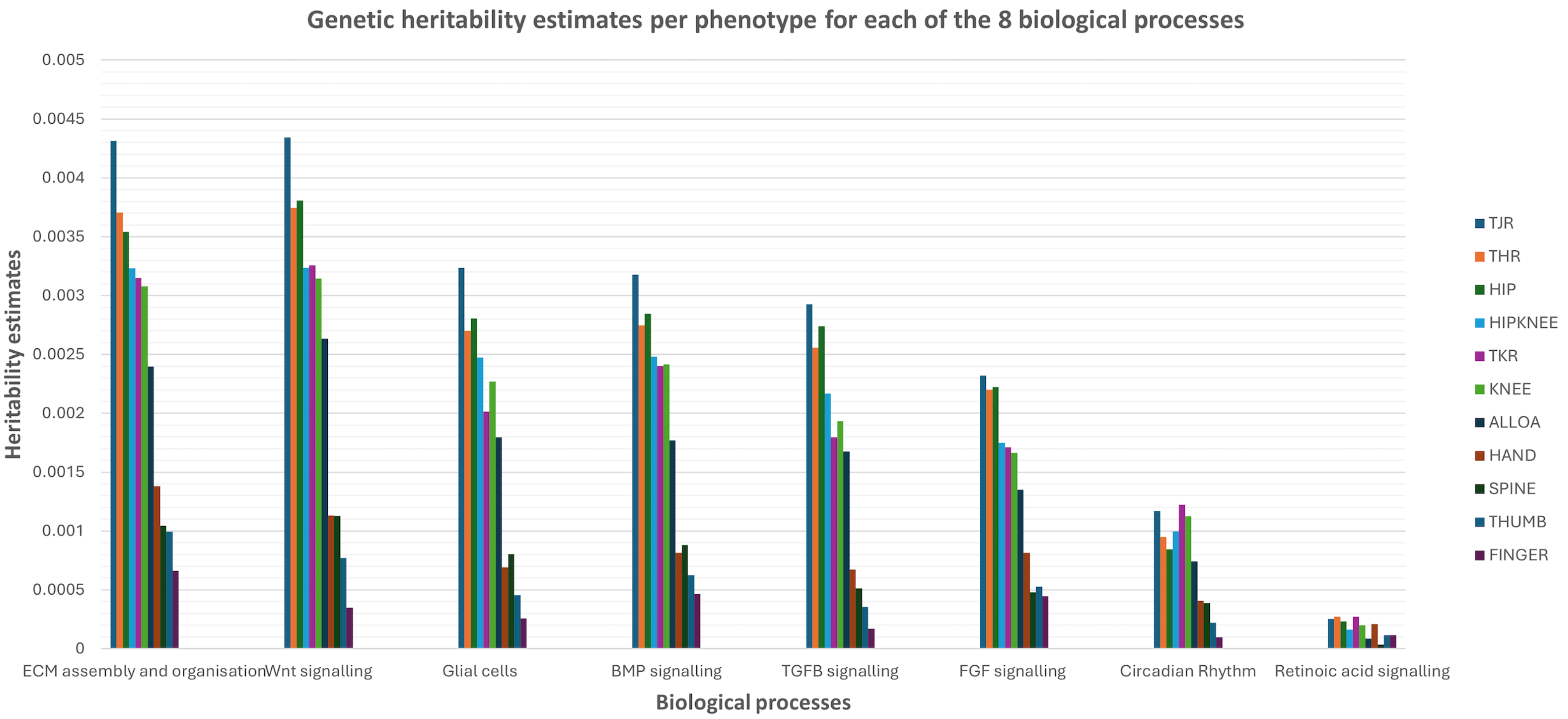

The bars depict the heritability estimates (y-axis) for various phenotypes across eight biological processes (x-axis). Each colored bar represents a specific phenotype. Osteoarthritis at any site (ALLOA), hip osteoarthritis (HIP), knee osteoarthritis (KNEE), hip and/or knee osteoarthritis (HIPKNEE), spine osteoarthritis (SPINE), hand osteoarthritis (HAND), finger osteoarthritis (FINGER), thumb osteoarthritis (TUMB), total hip replacement (THR), total knee replacement (TKR) and total hip and/or knee replacement (total joint replacement; TJR).

Supplementary Figure 10. Allelic imbalance in subchondral bone.

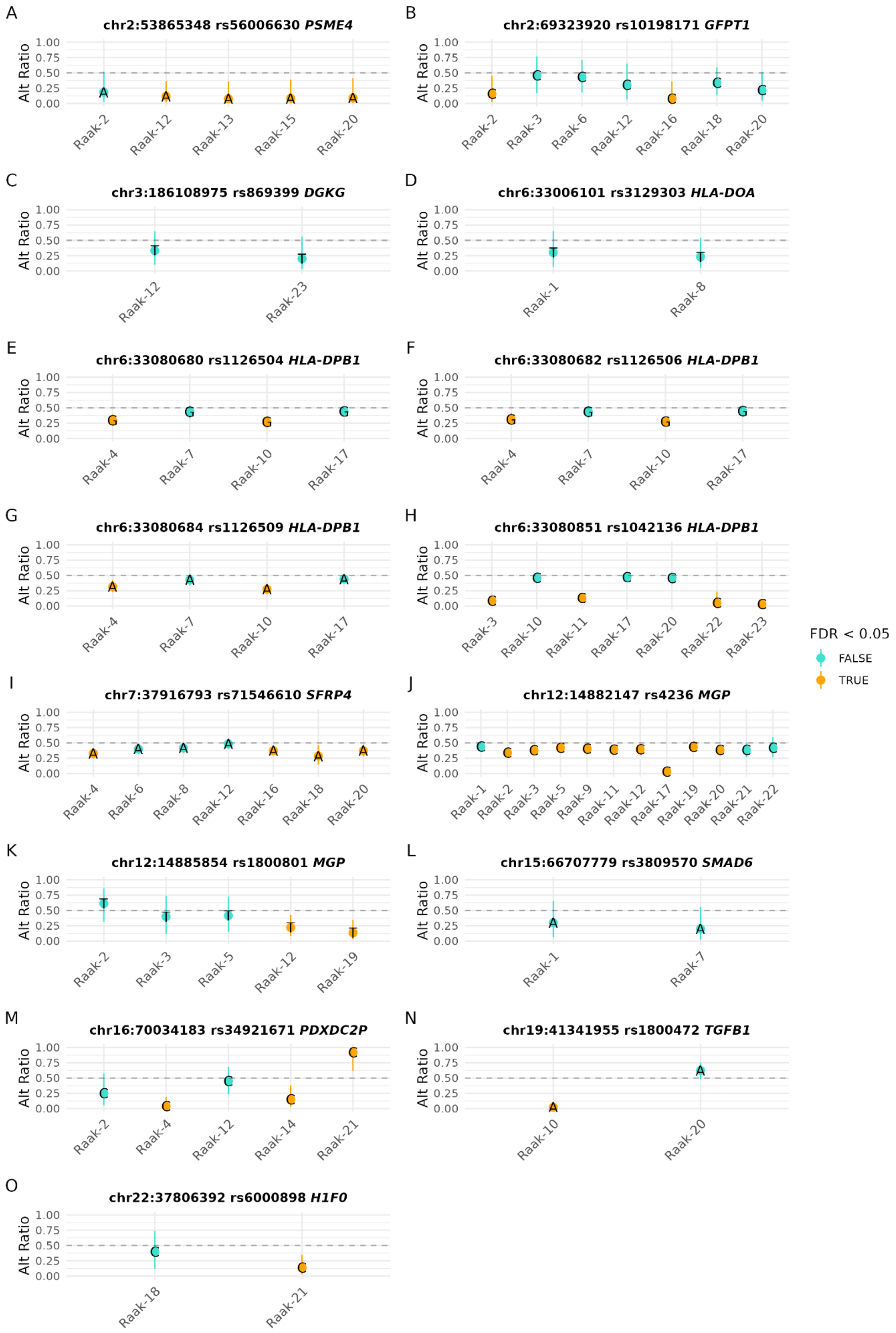

For each Allelic Imbalance variant mentioned in Supplementary Table 27 (A-O), the alternative allele (letter in point) ratio (Alt Ratio; y-axis) for each heterozygote (anonymized Raak-00; x-axis) in the RAAK in subchondral bone. The dotted line reflects the 1:1 allele ratio reference (Alt ratio = 0.5). Significant ratios (FDR < 0.05) are coloured orange, insignificant ratios blue.

Supplementary Figure 11. Additional pathways related to FGF signalling that have relevance to osteoarthritis.

A

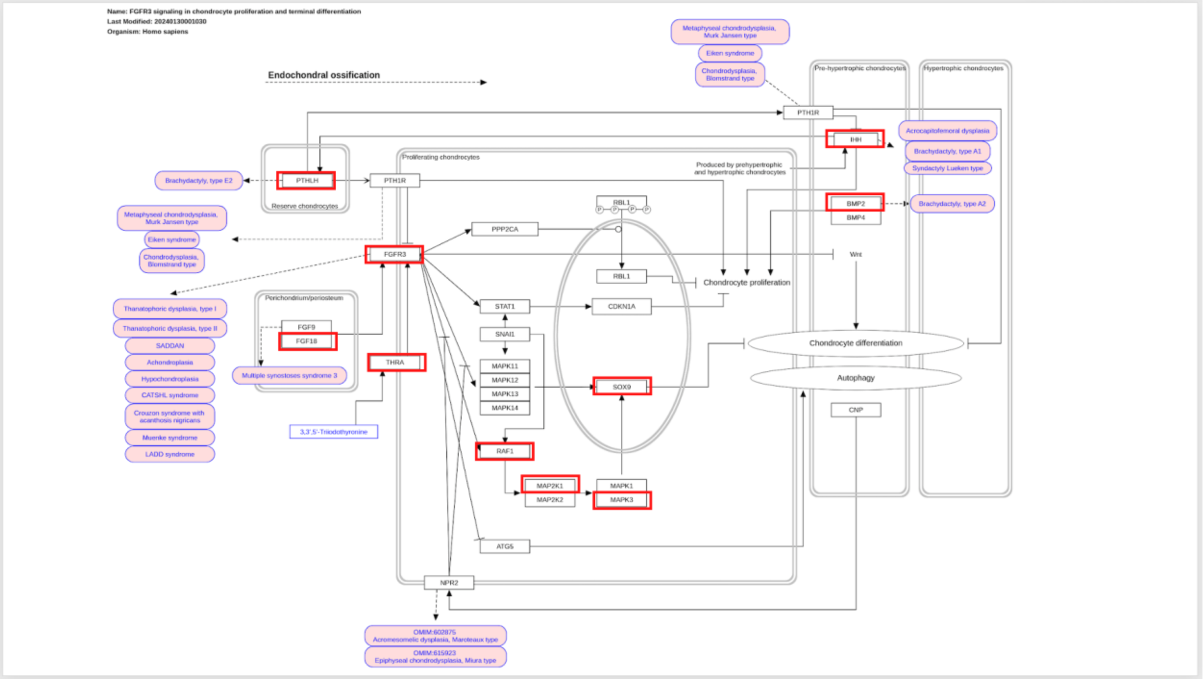

B

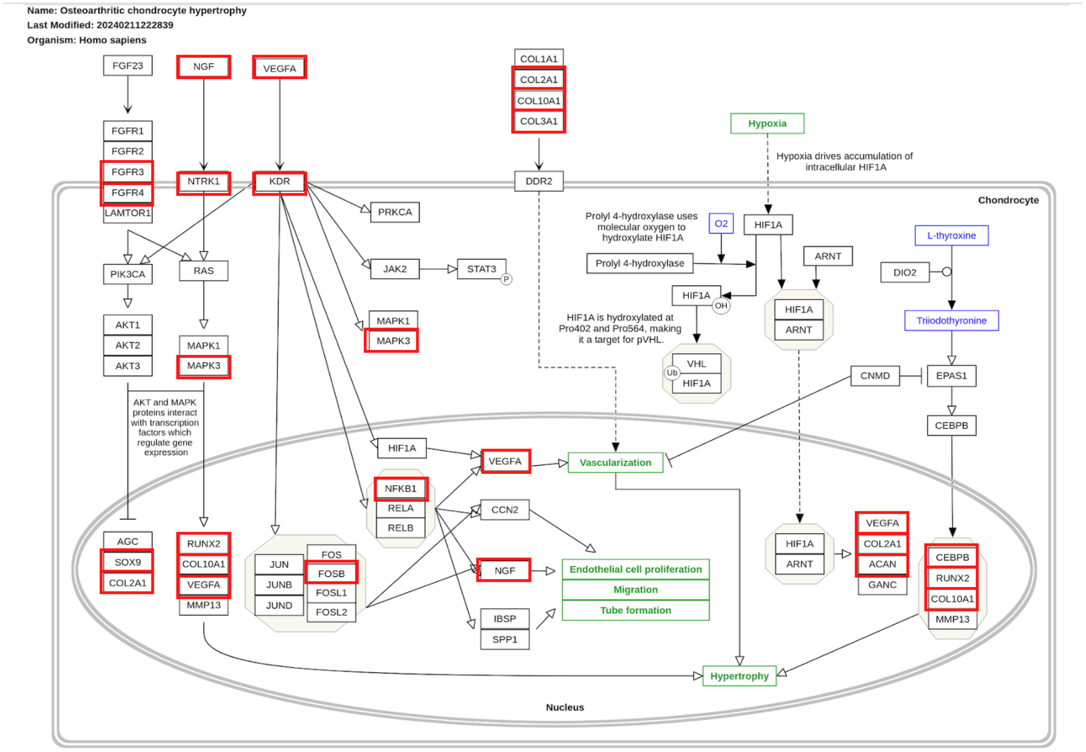

Additional pathways related to FGF signalling that have relevance to osteoarthritis that contain further effector genes not included directly in the FGF signalling pathway. A) Wikipathways FGFR3 signaling in chondrocyte proliferation and terminal differentiation (WP4767). 10 effector genes are highlighted in red. B) Osteoarthritic chondrocyte hypertrophy (WP5373), which is associated with dysregulation of FGF, hypoxia and angiogenesis [346], 16 effector genes are highlighted in red.

Supplementary Figure 12. Additional Wikipathways (WP5205) for genes involved in clock-controlled autophagy.

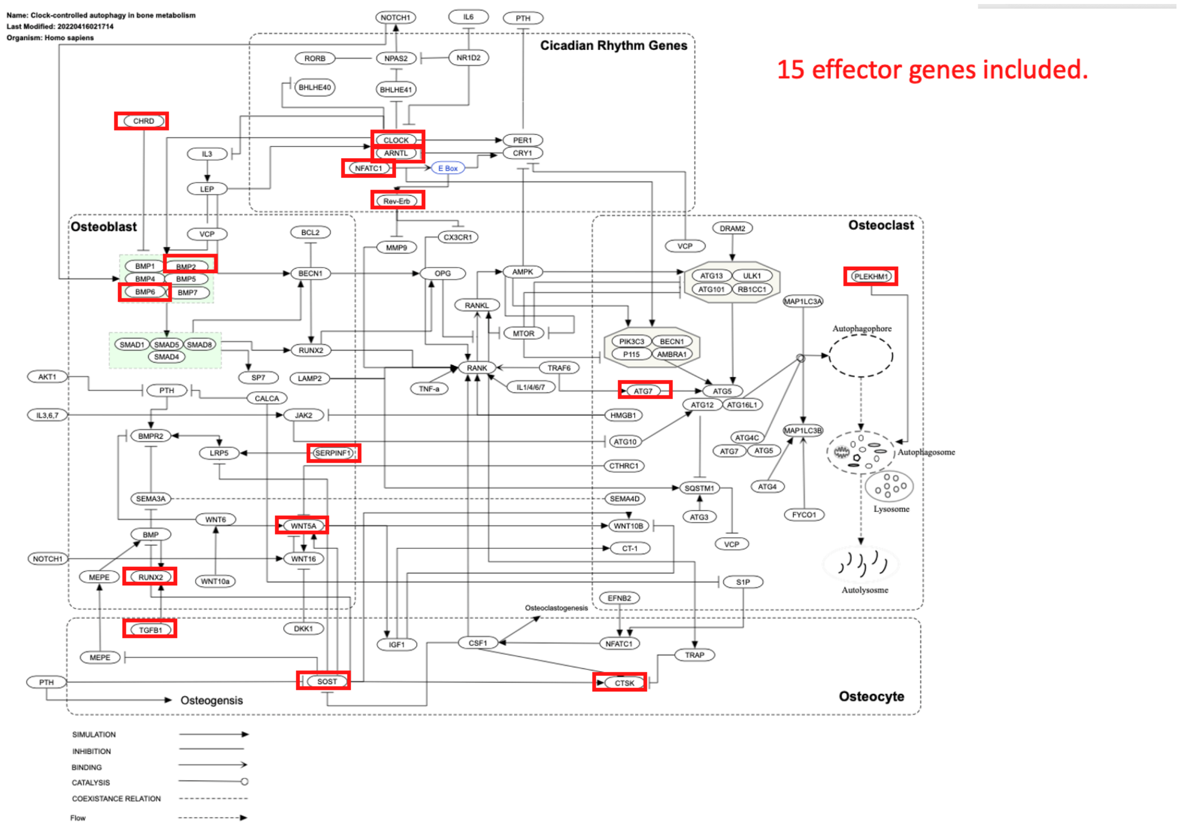

Fifteen effector genes are highlighted in red.

References

1 Baldwin, J. N. *et al.* Self-reported knee pain and disability among healthy individuals: reference data and factors associated with the Knee injury and Osteoarthritis Outcome Score (KOOS) and KOOS-Child. *Osteoarthritis Cartilage* **25**, 1282-1290 (2017).

2 Zengini, E. *et al.* Genome-wide analyses using UK Biobank data provide insights into the genetic architecture of osteoarthritis. *Nat Genet* **50**, 549-558 (2018).

3 Prieto-Alhambra, D. *et al.* An increased rate of falling leads to a rise in fracture risk in postmenopausal women with self-reported osteoarthritis: a prospective multinational cohort study (GLOW). *Ann Rheum Dis* **72**, 911-917 (2013).

4 Tschon, M., Contartese, D., Pagani, S., Borsari, V. & Fini, M. Gender and Sex Are Key Determinants in Osteoarthritis Not Only Confounding Variables. A Systematic Review of Clinical Data. *J Clin Med* **10**, 3178 (2021).

5 Srikanth, V. K. *et al.* A meta-analysis of sex differences prevalence, incidence and severity of osteoarthritis. *Osteoarthritis Cartilage* **13**, 769-781 (2005).

6 Jin, X. *et al.* Associations between endogenous sex hormones and MRI structural changes in patients with symptomatic knee osteoarthritis. *Osteoarthritis Cartilage* **25**, 1100-1106 (2017).

7 Bay-Jensen, A. C., Mobasheri, A., Thudium, C. S., Kraus, V. B. & Karsdal, M. A. Blood and urine biomarkers in osteoarthritis - an update on cartilage associated type II collagen and aggrecan markers. *Curr Opin Rheumatol* **34**, 54-60 (2022).

8 Masetti, R. *et al.* Chemotherapy-free treatment for acute promyelocytic leukemia: the pediatric view of a revolutionary tale. *Front Oncol* **13**, 1135350 (2023).

9 Szymański, Ł. *et al.* Retinoic Acid and Its Derivatives in Skin. *Cells* **9**, 2660 (2020).

10 Styrkarsdottir, U. *et al.* Severe osteoarthritis of the hand associates with common variants within the ALDH1A2 gene and with rare variants at 1p31. *Nat Genet* **46**, 498-502 (2014).

11 Shepherd, C. *et al.* Functional Characterization of the Osteoarthritis Genetic Risk Residing at ALDH1A2 Identifies rs12915901 as a Key Target Variant. *Arthritis Rheumatol* **70**, 1577-1587 (2018).

12 Zhu, L. *et al.* Variants in ALDH1A2 reveal an anti-inflammatory role for retinoic acid and a new class of disease-modifying drugs in osteoarthritis. *Sci Transl Med* **14**, eabm4054 (2022).

13 Krivospitskaya, O. *et al.* A CYP26B1 polymorphism enhances retinoic acid catabolism and may aggravate atherosclerosis. *Mol Med* **18**, 712-718 (2012).

14 Ward, L. D. & Kellis, M. HaploReg: a resource for exploring chromatin states, conservation, and regulatory motif alterations within sets of genetically linked variants. *Nucleic Acids Res* **40**, D930-934 (2012).

15 Nagpal, S. *et al.* Tazarotene-induced gene 2 (TIG2), a novel retinoid-responsive gene in skin. *J Invest Dermatol* **109**, 91-95 (1997).

16 Helfer, G. & Wu, Q. F. Chemerin: a multifaceted adipokine involved in metabolic disorders. *J Endocrinol* **238**, R79-R94 (2018).

17 Tan, L., Lu, X., Danser, A. H. J. & Verdonk, K. The Role of Chemerin in Metabolic and Cardiovascular Disease: A Literature Review of Its Physiology and Pathology from a Nutritional Perspective. *Nutrients* **15**, 2878 (2023).

18 Parlee, S. D., Ernst, M. C., Muruganandan, S., Sinal, C. J. & Goralski, K. B. Serum chemerin levels vary with time of day and are modified by obesity and tumor necrosis factor-{alpha}. *Endocrinology* **151**, 2590-2602 (2010).

19 Ma, J., Niu, D. S., Wan, N. J., Qin, Y. & Guo, C. J. Elevated chemerin levels in synovial fluid and synovial membrane from patients with knee osteoarthritis. *Int J Clin Exp Pathol* **8**, 13393-13398 (2015).
20 Zhao, L., Leung, L. L. & Morser, J. Chemerin Forms: Their Generation and Activity. *Biomedicines* **10**, 2018 (2022).
21 Xie, Y. & Liu, L. Role of Chemerin/ChemR23 axis as an emerging therapeutic perspective on obesity-related vascular dysfunction. *J Transl Med* **20**, 141 (2022).
22 Lima de Carvalho, J. R., Jr. *et al.* Effects of deficiency in the RLBP1-encoded visual cycle protein CRALBP on visual dysfunction in humans and mice. *J Biol Chem* **295**, 6767-6780 (2020).
23 Maw, M. A. *et al.* Mutation of the gene encoding cellular retinaldehyde-binding protein in autosomal recessive retinitis pigmentosa. *Nat Genet* **17**, 198-200 (1997).
24 Keenan, T. D., Goldacre, R. & Goldacre, M. J. ASSOCIATIONS BETWEEN AGE-RELATED MACULAR DEGENERATION, OSTEOARTHRITIS AND RHEUMATOID ARTHRITIS: RECORD LINKAGE STUDY. *Retina* **35**, 2613-2618 (2015).
25 Chiu, Y. H., Huang, J. Y., Huang, Y. P. & Pan, S. L. Osteoarthritis Is Associated With an Increased Risk of Age-Related Macular Degeneration: A Population-Based Longitudinal Follow-Up Study. *Front Med (Lausanne)* **9**, 854629 (2022).
26 Chong, E. W. *et al.* Age Related Macular Degeneration and Total Hip Replacement Due to Osteoarthritis or Fracture: Melbourne Collaborative Cohort Study. *PLoS One* **10**, e0137322 (2015).
27 Przyklenk, M. *et al.* LTBP1 promotes fibrillin incorporation into the extracellular matrix. *Matrix Biol* **110**, 60-75 (2022).
28 Rifkin, D. B. Latent transforming growth factor-beta (TGF-beta) binding proteins: orchestrators of TGF-beta availability. *J Biol Chem* **280**, 7409-7412 (2005).
29 Dubois, C. M., Laprise, M. H., Blanchette, F., Gentry, L. E. & Leduc, R. Processing of transforming growth factor beta 1 precursor by human furin convertase. *J Biol Chem* **270**, 10618-10624 (1995).
30 Robertson, I. B. & Rifkin, D. B. Regulation of the Bioavailability of TGF-β and TGF-β-Related Proteins. *Cold Spring Harb Perspect Biol* **8**, a021907 (2016).
31 Kuang, M. *et al.* Up-regulation of FUT8 inhibits TGF-β1-induced activation of hepatic stellate cells during liver fibrogenesis. *Glycoconj J* **38**, 77-87 (2021).
32 Tu, C. F., Wu, M. Y., Lin, Y. C., Kannagi, R. & Yang, R. B. FUT8 promotes breast cancer cell invasiveness by remodeling TGF-β receptor core fucosylation. *Breast Cancer Res* **19**, 111 (2017).
33 Lin, H. *et al.* Blocking core fucosylation of TGF-β1 receptors downregulates their functions and attenuates the epithelial-mesenchymal transition of renal tubular cells. *Am J Physiol Renal Physiol* **300**, F1017-1025 (2011).
34 Wang, X. *et al.* Dysregulation of TGF-beta1 receptor activation leads to abnormal lung development and emphysema-like phenotype in core fucose-deficient mice. *Proc Natl Acad Sci U S A* **102**, 15791-15796 (2005).
35 Yu, H. *et al.* Characterization of aberrant glycosylation associated with osteoarthritis based on integrated glycomics methods. *Arthritis Res Ther* **25**, 102 (2023).
36 Homan, K. *et al.* Articular cartilage corefucosylation regulates tissue resilience in osteoarthritis. **12**, RP92275 (2023).
37 Rapisarda, V. *et al.* Integrin Beta 3 Regulates Cellular Senescence by Activating the TGF-β Pathway. *Cell Rep* **18**, 2480-2493 (2017).
38 Luo, K. Ski and SnoN: negative regulators of TGF-beta signaling. *Curr Opin Genet Dev* **14**, 65-70 (2004).
39 Kim, K. O. *et al.* Ski inhibits TGF-β/phospho-Smad3 signaling and accelerates hypertrophic differentiation in chondrocytes. *J Cell Biochem* **113**, 2156-2166 (2012).

40 Imamura, T. *et al.* Smad6 inhibits signalling by the TGF-beta superfamily. *Nature* **389**, 622-626 (1997).
41 Hata, A., Lagna, G., Massagué, J. & Hemmati-Brivanlou, A. Smad6 inhibits BMP/Smad1 signaling by specifically competing with the Smad4 tumor suppressor. *Genes Dev* **12**, 186-197 (1998).
42 Estrada, K. D., Retting, K. N., Chin, A. M. & Lyons, K. M. Smad6 is essential to limit BMP signaling during cartilage development. *J Bone Miner Res* **26**, 2498-2510 (2011).
43 Liu, Y., Zhou, H. & Tang, X. STUB1/CHIP: New insights in cancer and immunity. *Biomed Pharmacother* **165**, 115190 (2023).
44 Mamun, M. M. A. *et al.* Stub1 maintains proteostasis of master transcription factors in embryonic stem cells. *Cell Rep* **39**, 110919 (2022).
45 Li, X. *et al.* CHIP promotes Runx2 degradation and negatively regulates osteoblast differentiation. *J Cell Biol* **181**, 959-972 (2008).
46 Wang, W. *et al.* CHIP regulates skeletal development and postnatal bone growth. *J Cell Physiol* **235**, 5378-5385 (2020).
47 Li, S. *et al.* Carboxyl terminus of Hsp70-interacting protein regulation of osteoclast formation in mice through promotion of tumor necrosis factor receptor-associated factor 6 protein degradation. *Arthritis Rheumatol* **66**, 1854-1863 (2014).
48 Li, L. *et al.* CHIP mediates degradation of Smad proteins and potentially regulates Smad-induced transcription. *Mol Cell Biol* **24**, 856-864 (2004).
49 Xin, H. *et al.* CHIP controls the sensitivity of transforming growth factor-beta signaling by modulating the basal level of Smad3 through ubiquitin-mediated degradation. *J Biol Chem* **280**, 20842-20850 (2005).
50 Stroschein, S. L., Wang, W., Zhou, S., Zhou, Q. & Luo, K. Negative feedback regulation of TGF-beta signaling by the SnoN oncoprotein. *Science* **286**, 771-774 (1999).
51 Matzuk, M. M. *et al.* Multiple defects and perinatal death in mice deficient in follistatin. *Nature* **374**, 360-363 (1995).
52 Abe, Y., Abe, T., Aida, Y., Hara, Y. & Maeda, K. Follistatin restricts bone morphogenetic protein (BMP)-2 action on the differentiation of osteoblasts in fetal rat mandibular cells. *J Bone Miner Res* **19**, 1302-1307 (2004).
53 Chang, C. Agonists and Antagonists of TGF-β Family Ligands. *Cold Spring Harb Perspect Biol* **8**, a021923 (2016).
54 Tang, R. *et al.* Gene therapy for follistatin mitigates systemic metabolic inflammation and post-traumatic arthritis in high-fat diet-induced obesity. *Sci Adv* **6**, eaaz7492 (2020).
55 Liu, Y., Hou, R., Yin, R. & Yin, W. Correlation of bone morphogenetic protein-2 levels in serum and synovial fluid with disease severity of knee osteoarthritis. *Med Sci Monit* **21**, 363-370 (2015).
56 Blaney Davidson, E. N. *et al.* Elevated extracellular matrix production and degradation upon bone morphogenetic protein-2 (BMP-2) stimulation point toward a role for BMP-2 in cartilage repair and remodeling. *Arthritis Res Ther* **9**, R102 (2007).
57 Pester, J. K. *et al.* BMP-2 shows characteristic extracellular patterns in osteoarthritic cartilage: a preliminary report. *GMS Interdiscip Plast Reconstr Surg DGPW* **2**, Doc09 (2013).
58 Whitty, C. *et al.* Sustained delivery of the bone morphogenetic proteins BMP-2 and BMP-7 for cartilage repair and regeneration in osteoarthritis. *Osteoarthr Cartil Open* **4**, 100240 (2022).
59 Gao, X. *et al.* A comparison of BMP2 delivery by coacervate and gene therapy for promoting human muscle-derived stem cell-mediated articular cartilage repair. *Stem Cell Res Ther* **10**, 346 (2019).

60 Lin, H. *et al.* Efficient in vivo bone formation by BMP-2 engineered human mesenchymal stem cells encapsulated in a projection stereolithographically fabricated hydrogel scaffold. *Stem Cell Res Ther* **10**, 254 (2019).
61 Sampath, T. K. & Vukicevic, S. Biology of bone morphogenetic protein in bone repair and regeneration: A role for autologous blood coagulum as carrier. *Bone* **141**, 115602 (2020).
62 Katagiri, T. & Watabe, T. Bone Morphogenetic Proteins. *Cold Spring Harb Perspect Biol* **8**, a021899 (2016).
63 Gamer, L. W. *et al.* The Role of Bmp2 in the Maturation and Maintenance of the Murine Knee Joint. *J Bone Miner Res* **33**, 1708-1717 (2018).
64 Ullah, A. *et al.* A novel homozygous variant in BMPR1B underlies acromesomelic dysplasia Hunter-Thompson type. *Ann Hum Genet* **82**, 129-134 (2018).
65 Aggarwal, B. B. Signalling pathways of the TNF superfamily: a double-edged sword. *Nat Rev Immunol* **3**, 745-756 (2003).
66 Zhao, B. TNF and Bone Remodeling. *Curr Osteoporos Rep* **15**, 126-134 (2017).
67 Xia, Y. *et al.* TGFβ reprograms TNF stimulation of macrophages towards a non-canonical pathway driving inflammatory osteoclastogenesis. *Nat Commun* **13**, 3920 (2022).
68 Liu, T., Zhang, L., Joo, D. & Sun, S. C. NF-κB signaling in inflammation. *Signal Transduct Target Ther* **2**, 17023 (2017).
69 Choi, M. C., Jo, J., Park, J., Kang, H. K. & Park, Y. NF-κB Signaling Pathways in Osteoarthritic Cartilage Destruction. *Cells* **8**, 734 (2019).
70 Freudlsperger, C. *et al.* TGF-β and NF-κB signal pathway cross-talk is mediated through TAK1 and SMAD7 in a subset of head and neck cancers. *Oncogene* **32**, 1549-1559 (2013).
71 Hapak, S. M., Rothlin, C. V. & Ghosh, S. aPKC in neuronal differentiation, maturation and function. *Neuronal Signal* **3**, NS20190019 (2019).
72 Ozdamar, B. *et al.* Regulation of the polarity protein Par6 by TGFbeta receptors controls epithelial cell plasticity. *Science* **307**, 1603-1609 (2005).
73 Hakanen, J., Ruiz-Reig, N. & Tissir, F. Linking Cell Polarity to Cortical Development and Malformations. *Front Cell Neurosci* **13**, 244 (2019).
74 Kaiser, J. *et al.* TGFβ1 Induces Axonal Outgrowth via ALK5/PKA/SMURF1-Mediated Degradation of RhoA and Stabilization of PAR6. *eNeuro* **7**, ENEURO.0104-0120.2020 (2020).
75 Rahmati, M., Nalesso, G., Mobasheri, A. & Mozafari, M. Aging and osteoarthritis: Central role of the extracellular matrix. *Ageing Res Rev* **40**, 20-30 (2017).
76 Verrecchia, F., Chu, M. L. & Mauviel, A. Identification of novel TGF-beta /Smad gene targets in dermal fibroblasts using a combined cDNA microarray/promoter transactivation approach. *J Biol Chem* **276**, 17058-17062 (2001).
77 Ni, G. X., Li, Z. & Zhou, Y. Z. The role of small leucine-rich proteoglycans in osteoarthritis pathogenesis. *Osteoarthritis Cartilage* **22**, 896-903 (2014).
78 Hildebrand, A. *et al.* Interaction of the small interstitial proteoglycans biglycan, decorin and fibromodulin with transforming growth factor beta. *Biochem J* **302 (Pt 2)**, 527-534 (1994).
79 Luu, H. H. *et al.* Distinct roles of bone morphogenetic proteins in osteogenic differentiation of mesenchymal stem cells. *J Orthop Res* **25**, 665-677 (2007).
80 Deng, Z. H., Li, Y. S., Gao, X., Lei, G. H. & Huard, J. Bone morphogenetic proteins for articular cartilage regeneration. *Osteoarthritis Cartilage* **26**, 1153-1161 (2018).
81 Moutsatsos, I. K. *et al.* Exogenously regulated stem cell-mediated gene therapy for bone regeneration. *Mol Ther* **3**, 449-461 (2001).

82 Mizrahi, O. *et al.* BMP-6 is more efficient in bone formation than BMP-2 when overexpressed in mesenchymal stem cells. *Gene Ther* **20**, 370-377 (2013).
83 Chiari, C. *et al.* Recombinant Human BMP6 Applied Within Autologous Blood Coagulum Accelerates Bone Healing: Randomized Controlled Trial in High Tibial Osteotomy Patients. *J Bone Miner Res* **35**, 1893-1903 (2020).
84 Erlacher, L. *et al.* Cartilage-derived morphogenetic proteins and osteogenic protein-1 differentially regulate osteogenesis. *J Bone Miner Res* **13**, 383-392 (1998).
85 Klammert, U. *et al.* GDF-5 can act as a context-dependent BMP-2 antagonist. *BMC Biol* **13**, 77 (2015).
86 Kania, K. *et al.* Regulation of Gdf5 expression in joint remodelling, repair and osteoarthritis. *Sci Rep* **10**, 157 (2020).
87 Deng, P., Yu, Y., Hong, C. & Wang, C. Y. Growth differentiation factor 6, a repressive target of EZH2, promotes the commitment of human embryonic stem cells to mesenchymal stem cells. *Bone Res* **8**, 39 (2020).
88 Clarke, L. E. *et al.* Growth differentiation factor 6 and transforming growth factor-beta differentially mediate mesenchymal stem cell differentiation, composition, and micromechanical properties of nucleus pulposus constructs. *Arthritis Res Ther* **16**, R67 (2014).
89 Correns, A., Zimmermann, L. A., Baldock, C. & Sengle, G. BMP antagonists in tissue development and disease. *Matrix Biol Plus* **11**, 100071 (2021).
90 Chen, H. *et al.* CHRDL2 promotes osteosarcoma cell proliferation and metastasis through the BMP-9/PI3K/AKT pathway. *Cell Biol Int* **45**, 623-632 (2021).
91 Chien, S. Y. *et al.* Noggin Inhibits IL-1β and BMP-2 Expression, and Attenuates Cartilage Degeneration and Subchondral Bone Destruction in Experimental Osteoarthritis. *Cells* **9**, 927 (2020).
92 Wen, L. *et al.* Noggin, an inhibitor of bone morphogenetic protein signaling, antagonizes TGF-β1 in a mouse model of osteoarthritis. *Biochem Biophys Res Commun* **570**, 199-205 (2021).
93 Mang, T. *et al.* BMPR1A is necessary for chondrogenesis and osteogenesis, whereas BMPR1B prevents hypertrophic differentiation. *J Cell Sci* **133**, jcs246934 (2020).
94 Schmidt, V., Subkhangulova, A. & Willnow, T. E. Sorting receptor SORLA: cellular mechanisms and implications for disease. *Cell Mol Life Sci* **74**, 1475-1483 (2017).
95 Whittle, A. J. *et al.* Soluble LR11/SorLA represses thermogenesis in adipose tissue and correlates with BMI in humans. *Nat Commun* **6**, 8951 (2015).
96 Liu, P. *et al.* Requirement for Wnt3 in vertebrate axis formation. *Nat Genet* **22**, 361-365 (1999).
97 Niemann, S. *et al.* Homozygous WNT3 mutation causes tetra-amelia in a large consanguineous family. *Am J Hum Genet* **74**, 558-563 (2004).
98 Kim, J. H. *et al.* Wnt signaling in bone formation and its therapeutic potential for bone diseases. *Ther Adv Musculoskelet Dis* **5**, 13-31 (2013).
99 Weivoda, M. M. *et al.* Wnt Signaling Inhibits Osteoclast Differentiation by Activating Canonical and Noncanonical cAMP/PKA Pathways. *J Bone Miner Res* **31**, 65-75 (2016).
100 Hill, T. P., Später, D., Taketo, M. M., Birchmeier, W. & Hartmann, C. Canonical Wnt/beta-catenin signaling prevents osteoblasts from differentiating into chondrocytes. *Dev Cell* **8**, 727-738 (2005).
101 Bradley, E. W. & Drissi, M. H. WNT5A regulates chondrocyte differentiation through differential use of the CaN/NFAT and IKK/NF-kappaB pathways. *Mol Endocrinol* **24**, 1581-1593 (2010).
102 Yang, Y., Topol, L., Lee, H. & Wu, J. Wnt5a and Wnt5b exhibit distinct activities in coordinating chondrocyte proliferation and differentiation. *Development* **130**, 1003-1015 (2003).

103 Huang, G., Chubinskaya, S., Liao, W. & Loeser, R. F. Wnt5a induces catabolic signaling and matrix metalloproteinase production in human articular chondrocytes. *Osteoarthritis Cartilage* **25**, 1505-1515 (2017).
104 Katsoula, G. *et al.* A molecular map of long non-coding RNA expression, isoform switching and alternative splicing in osteoarthritis. *Hum Mol Genet* **31**, 2090-2105 (2022).
105 Ashburner, M. *et al.* Gene ontology: tool for the unification of biology. The Gene Ontology Consortium. *Nat Genet* **25**, 25-29 (2000).
106 Gene Ontology Consortium *et al.* The Gene Ontology knowledgebase in 2023. *Genetics* **224**, iyad031 (2023).
107 Zhong, Q. *et al.* Cryo-EM structure of human Wntless in complex with Wnt3a. *Nat Commun* **12**, 4541 (2021).
108 Nygaard, R. *et al.* Structural Basis of WLS/Evi-Mediated Wnt Transport and Secretion. *Cell* **184**, 194-206.e114 (2021).
109 Kreitmaier, P. *et al.* An epigenome-wide view of osteoarthritis in primary tissues. *Am J Hum Genet* **109**, 1255-1271 (2022).
110 den Hollander, W. *et al.* Transcriptional associations of osteoarthritis-mediated loss of epigenetic control in articular cartilage. *Arthritis Rheumatol* **67**, 2108-2116 (2015).
111 Monteagudo, S. *et al.* DOT1L safeguards cartilage homeostasis and protects against osteoarthritis. *Nat Commun* **8**, 15889 (2017).
112 Castaño Betancourt, M. C. *et al.* Genome-wide association and functional studies identify the DOT1L gene to be involved in cartilage thickness and hip osteoarthritis. *Proc Natl Acad Sci U S A* **109**, 8218-8223 (2012).
113 Pawar, N. M. & Rao, P. Secreted frizzled related protein 4 (sFRP4) update: A brief review. *Cell Signal* **45**, 63-70 (2018).
114 Haraguchi, R. *et al.* sFRP4-dependent Wnt signal modulation is critical for bone remodeling during postnatal development and age-related bone loss. *Sci Rep* **6**, 25198 (2016).
115 Sowińska-Seidler, A. *et al.* The First Report of Biallelic Missense Mutations in the SFRP4 Gene Causing Pyle Disease in Two Siblings. *Front Genet* **11**, 593407 (2020).
116 van Bezooijen, R. L., ten Dijke, P., Papapoulos, S. E. & Löwik, C. W. SOST/sclerostin, an osteocyte-derived negative regulator of bone formation. *Cytokine Growth Factor Rev* **16**, 319-327 (2005).
117 Yu, S. *et al.* Drug discovery of sclerostin inhibitors. *Acta Pharm Sin B* **12**, 2150-2170 (2022).
118 Greenblatt, M. B. *et al.* NFATc1 and NFATc2 repress spontaneous osteoarthritis. *Proc Natl Acad Sci U S A* **110**, 19914-19919 (2013).
119 Winslow, M. M. *et al.* Calcineurin/NFAT signaling in osteoblasts regulates bone mass. *Dev Cell* **10**, 771-782 (2006).
120 Canalis, E., Schilling, L., Eller, T. & Yu, J. Nuclear factor of activated T cells 1 and 2 are required for vertebral homeostasis. *J Cell Physiol* **235**, 8520-8532 (2020).
121 Cesareo, R., Napolitano, C. & Iozzino, M. Strontium ranelate in postmenopausal osteoporosis treatment: a critical appraisal. *Int J Womens Health* **2**, 1-6 (2010).
122 Kołodziejska, B., Stępień, N. & Kolmas, J. The Influence of Strontium on Bone Tissue Metabolism and Its Application in Osteoporosis Treatment. *Int J Mol Sci* **22**, 6564 (2021).
123 Fromigué, O., Haÿ, E., Barbara, A. & Marie, P. J. Essential role of nuclear factor of activated T cells (NFAT)-mediated Wnt signaling in osteoblast differentiation induced by strontium ranelate. *J Biol Chem* **285**, 25251-25258 (2010).
124 Sluzalska, K. D. *et al.* Intracellular partners of fibroblast growth factors 1 and 2 - implications for functions. *Cytokine Growth Factor Rev* **57**, 93-111 (2021).

125 Yamashita, A. *et al.* Fibroblast growth factor-2 determines severity of joint disease in adjuvant-induced arthritis in rats. *J Immunol* **168**, 450-457 (2002).
126 Li, Y. X. *et al.* FGF1 reduces cartilage injury in osteoarthritis via regulating AMPK/Nrf2 pathway. *J Mol Histol* **54**, 427-438 (2023).
127 El-Seoudi, A. *et al.* Catabolic effects of FGF-1 on chondrocytes and its possible role in osteoarthritis. *J Cell Commun Signal* **11**, 255-263 (2017).
128 Arias-Gallo, J., Chamorro-Pons, M., Avendaño, C. & Giménez-Gallego, G. Influence of acidic fibroblast growth factor on bone regeneration in experimental cranial defects using spongostan and Bio-Oss as protein carriers. *J Craniofac Surg* **24**, 1507-1514 (2013).
129 Kisand, K., Tamm, A. E., Lintrop, M. & Tamm, A. O. New insights into the natural course of knee osteoarthritis: early regulation of cytokines and growth factors, with emphasis on sex-dependent angiogenesis and tissue remodeling. A pilot study. *Osteoarthritis Cartilage* **26**, 1045-1054 (2018).
130 Li, R. *et al.* Upregulation of fibroblast growth factor 1 in the synovial membranes of patients with late stage osteoarthritis. *Genet Mol Res* **14**, 11191-11199 (2015).
131 Yao, X. *et al.* Fibroblast growth factor 18 exerts anti-osteoarthritic effects through PI3K-AKT signaling and mitochondrial fusion and fission. *Pharmacol Res* **139**, 314-324 (2019).
132 Correa, D. *et al.* Sequential exposure to fibroblast growth factors (FGF) 2, 9 and 18 enhances hMSC chondrogenic differentiation. *Osteoarthritis Cartilage* **23**, 443-453 (2015).
133 DePhillipo, N. N. *et al.* Preclinical Use of FGF-18 Augmentation for Improving Cartilage Healing Following Surgical Repair: A Systematic Review. *Cartilage* **14**, 59-66 (2023).
134 Song, Z. *et al.* Sprifermin: Effects on Cartilage Homeostasis and Therapeutic Prospects in Cartilage-Related Diseases. *Front Cell Dev Biol* **9**, 786546 (2021).
135 Ohbayashi, N. *et al.* FGF18 is required for normal cell proliferation and differentiation during osteogenesis and chondrogenesis. *Genes Dev* **16**, 870-879 (2002).
136 Liu, Z., Lavine, K. J., Hung, I. H. & Ornitz, D. M. FGF18 is required for early chondrocyte proliferation, hypertrophy and vascular invasion of the growth plate. *Dev Biol* **302**, 80-91 (2007).
137 Sahni, M. *et al.* FGF signaling inhibits chondrocyte proliferation and regulates bone development through the STAT-1 pathway. *Genes Dev* **13**, 1361-1366 (1999).
138 Yan, D. *et al.* Fibroblast growth factor receptor 1 is principally responsible for fibroblast growth factor 2-induced catabolic activities in human articular chondrocytes. *Arthritis Res Ther* **13**, R130 (2011).
139 Kim, H. Y. & Ko, J. M. Clinical management and emerging therapies of FGFR3-related skeletal dysplasia in childhood. *Ann Pediatr Endocrinol Metab* **27**, 90-97 (2022).
140 Rozenblatt-Rosen, O. *et al.* Induction of chondrocyte growth arrest by FGF: transcriptional and cytoskeletal alterations. *J Cell Sci* **115**, 553-562 (2002).
141 Krejci, P. *et al.* STAT1 and STAT3 do not participate in FGF-mediated growth arrest in chondrocytes. *J Cell Sci* **121**, 272-281 (2008).
142 Wen, X. *et al.* Chondrocyte FGFR3 Regulates Bone Mass by Inhibiting Osteogenesis. *J Biol Chem* **291**, 24912-24921 (2016).
143 Weinstein, M., Xu, X., Ohyama, K. & Deng, C. X. FGFR-3 and FGFR-4 function cooperatively to direct alveogenesis in the murine lung. *Development* **125**, 3615-3623 (1998).
144 Cool, S., Jackson, R., Pincus, P., Dickinson, I. & Nurcombe, V. Fibroblast growth factor receptor 4 (FGFR4) expression in newborn murine calvaria and primary osteoblast cultures. *Int J Dev Biol* **46**, 519-523 (2002).

145 Trueb, B., Zhuang, L., Taeschler, S. & Wiedemann, M. Characterization of FGFRL1, a novel fibroblast growth factor (FGF) receptor preferentially expressed in skeletal tissues. *J Biol Chem* **278**, 33857-33865 (2003).
146 Silva, P. N., Altamentova, S. M., Kilkenny, D. M. & Rocheleau, J. V. Fibroblast growth factor receptor like-1 (FGFRL1) interacts with SHP-1 phosphatase at insulin secretory granules and induces beta-cell ERK1/2 protein activation. *J Biol Chem* **288**, 17859-17870 (2013).
147 Szybowska, P., Kostas, M., Wesche, J., Haugsten, E. M. & Wiedlocha, A. Negative Regulation of FGFR (Fibroblast Growth Factor Receptor) Signaling. *Cells* **10**, 1342 (2021).
148 Vincent, T. L. & Wann, A. K. T. Mechanoadaptation: articular cartilage through thick and thin. *J Physiol* **597**, 1271-1281 (2019).
149 Maldonado, M. & Nam, J. The role of changes in extracellular matrix of cartilage in the presence of inflammation on the pathology of osteoarthritis. *Biomed Res Int* **2013**, 284873 (2013).
150 Guilak, F., Nims, R. J., Dicks, A., Wu, C. L. & Meulenbelt, I. Osteoarthritis as a disease of the cartilage pericellular matrix. *Matrix Biol* **71-72**, 40-50 (2018).
151 Yengo, L. *et al.* A saturated map of common genetic variants associated with human height. *Nature* **610**, 704-712 (2022).
152 Cheng, J. *et al.* Accurate proteome-wide missense variant effect prediction with AlphaMissense. *Science* **381**, eadg7492 (2023).
153 Perera, R. S. *et al.* Variants of ACAN are associated with severity of lumbar disc herniation in patients with chronic low back pain. *PLoS One* **12**, e0181580 (2017).
154 Zheng, J., Luo, W. & Tanzer, M. L. Aggrecan synthesis and secretion. A paradigm for molecular and cellular coordination of multiglobular protein folding and intracellular trafficking. *J Biol Chem* **273**, 12999-13006 (1998).
155 Roughley, P. J. & Mort, J. S. The role of aggrecan in normal and osteoarthritic cartilage. *J Exp Orthop* **1**, 8 (2014).
156 Cho, H. *et al.* Regulation of circadian behaviour and metabolism by REV-ERB-α and REV-ERB-β. *Nature* **485**, 123-127 (2012).
157 Preitner, N. *et al.* The orphan nuclear receptor REV-ERBalpha controls circadian transcription within the positive limb of the mammalian circadian oscillator. *Cell* **110**, 251-260 (2002).
158 Dudek, M. *et al.* The chondrocyte clock gene Bmal1 controls cartilage homeostasis and integrity. *J Clin Invest* **126**, 365-376 (2016).
159 Yuan, G. *et al.* Clock mutant promotes osteoarthritis by inhibiting the acetylation of NFκB. *Osteoarthritis Cartilage* **27**, 922-931 (2019).
160 Akagi, R. *et al.* Dysregulated circadian rhythm pathway in human osteoarthritis: NR1D1 and BMAL1 suppression alters TGF-β signaling in chondrocytes. *Osteoarthritis Cartilage* **25**, 943-951 (2017).
161 So, A. Y., Bernal, T. U., Pillsbury, M. L., Yamamoto, K. R. & Feldman, B. J. Glucocorticoid regulation of the circadian clock modulates glucose homeostasis. *Proc Natl Acad Sci U S A* **106**, 17582-17587 (2009).
162 Torra, I. P. *et al.* Circadian and glucocorticoid regulation of Rev-erbalpha expression in liver. *Endocrinology* **141**, 3799-3806 (2000).
163 Cheon, S., Park, N., Cho, S. & Kim, K. Glucocorticoid-mediated Period2 induction delays the phase of circadian rhythm. *Nucleic Acids Res* **41**, 6161-6174 (2013).
164 Carey, K. T. *et al.* Nfil3 is a glucocorticoid-regulated gene required for glucocorticoid-induced apoptosis in male murine T cells. *Endocrinology* **154**, 1540-1552 (2013).

165 Jones, J. R., Chaturvedi, S., Granados-Fuentes, D. & Herzog, E. D. Circadian neurons in the paraventricular nucleus entrain and sustain daily rhythms in glucocorticoids. *Nat Commun* **12**, 5763 (2021).
166 Dickmeis, T. Glucocorticoids and the circadian clock. *J Endocrinol* **200**, 3-22 (2009).
167 Oster, H. *et al.* The Functional and Clinical Significance of the 24-Hour Rhythm of Circulating Glucocorticoids. *Endocr Rev* **38**, 3-45 (2017).
168 Rossetti, C., Cherix, A., Guiraud, L. F. & Cardinaux, J. R. New Insights Into the Pivotal Role of CREB-Regulated Transcription Coactivator 1 in Depression and Comorbid Obesity. *Front Mol Neurosci* **15**, 810641 (2022).
169 Sakamoto, K. *et al.* Clock and light regulation of the CREB coactivator CRTC1 in the suprachiasmatic circadian clock. *J Neurosci* **33**, 9021-9027 (2013).
170 Parra-Damas, A., Rubió-Ferrarons, L., Shen, J. & Saura, C. A. CRTC1 mediates preferential transcription at neuronal activity-regulated CRE/TATA promoters. *Sci Rep* **7**, 18004 (2017).
171 Jagannath, A. *et al.* The CRTC1-SIK1 pathway regulates entrainment of the circadian clock. *Cell* **154**, 1100-1111 (2013).
172 Kim, M. K. *et al.* Salt-inducible kinase 1 regulates bone anabolism via the CRTC1-CREB-Id1 axis. *Cell Death Dis* **10**, 826 (2019).
173 Paneque, A., Fortus, H., Zheng, J., Werlen, G. & Jacinto, E. The Hexosamine Biosynthesis Pathway: Regulation and Function. *Genes (Basel)* **14**, 933 (2023).
174 Liu, X., Cai, Y. D. & Chiu, J. C. Regulation of protein O-GlcNAcylation by circadian, metabolic, and cellular signals. *J Biol Chem* **300**, 105616 (2024).
175 Li, M. D. *et al.* O-GlcNAc signaling entrains the circadian clock by inhibiting BMAL1/CLOCK ubiquitination. *Cell Metab* **17**, 303-310 (2013).
176 Riegger, J., Baumert, J., Zaucke, F. & Brenner, R. E. The Hexosamine Biosynthetic Pathway as a Therapeutic Target after Cartilage Trauma: Modification of Chondrocyte Survival and Metabolism by Glucosamine Derivatives and PUGNAc in an Ex Vivo Model. *Int J Mol Sci* **22**, 7247 (2021).
177 McAlindon, T. E. *et al.* OARSI guidelines for the non-surgical management of knee osteoarthritis. *Osteoarthritis Cartilage* **22**, 363-388 (2014).
178 Mendoza, J. & Challet, E. Circadian insights into dopamine mechanisms. *Neuroscience* **282**, 230-242 (2014).
179 Korshunov, K. S., Blakemore, L. J. & Trombley, P. Q. Dopamine: A Modulator of Circadian Rhythms in the Central Nervous System. *Front Cell Neurosci* **11**, 91 (2017).
180 Morin, L. P. Serotonin and the regulation of mammalian circadian rhythmicity. *Ann Med* **31**, 12-33 (1999).
181 Goel, M. & Mangel, S. C. Dopamine-Mediated Circadian and Light/Dark-Adaptive Modulation of Chemical and Electrical Synapses in the Outer Retina. *Front Cell Neurosci* **15**, 647541 (2021).
182 Portas, C. M., Bjorvatn, B. & Ursin, R. Serotonin and the sleep/wake cycle: special emphasis on microdialysis studies. *Prog Neurobiol* **60**, 13-35 (2000).
183 Yoshitane, H. *et al.* JNK regulates the photic response of the mammalian circadian clock. *EMBO Rep* **13**, 455-461 (2012).
184 Goldsmith, C. S. & Bell-Pedersen, D. Diverse roles for MAPK signaling in circadian clocks. *Adv Genet* **84**, 1-39 (2013).
185 Oster, H. *et al.* cGMP-dependent protein kinase II modulates mPer1 and mPer2 gene induction and influences phase shifts of the circadian clock. *Curr Biol* **13**, 725-733 (2003).
186 Pitts, G. R., Ohta, H. & McMahon, D. G. Daily rhythmicity of large-conductance Ca2+ -activated K+ currents in suprachiasmatic nucleus neurons. *Brain Res* **1071**, 54-62 (2006).

187 Montgomery, J. R., Whitt, J. P., Wright, B. N., Lai, M. H. & Meredith, A. L. Mis-expression of the BK K(+) channel disrupts suprachiasmatic nucleus circuit rhythmicity and alters clock-controlled behavior. *Am J Physiol Cell Physiol* **304**, C299-311 (2013).
188 Meredith, A. L. *et al.* BK calcium-activated potassium channels regulate circadian behavioral rhythms and pacemaker output. *Nat Neurosci* **9**, 1041-1049 (2006).
189 Dinsdale, R. L., Roache, C. E. & Meredith, A. L. Disease-associated KCNMA1 variants decrease circadian clock robustness in channelopathy mouse models. *J Gen Physiol* **155**, e202313357 (2023).
190 Kent, J. & Meredith, A. L. BK channels regulate spontaneous action potential rhythmicity in the suprachiasmatic nucleus. *PLoS One* **3**, e3884 (2008).
191 Bouâouda, H. & Jha, P. K. Orexin and MCH neurons: regulators of sleep and metabolism. *Front Neurosci* **17**, 1230428 (2023).
192 Willie, J. T. *et al.* Distinct narcolepsy syndromes in Orexin receptor-2 and Orexin null mice: molecular genetic dissection of Non-REM and REM sleep regulatory processes. *Neuron* **38**, 715-730 (2003).
193 Popescu, C. Combined genotype of HCRTR2 and CLOCK variants in a large family of cluster headache with familial periodicity phenotype. *J Neurol* **270**, 5064-5070 (2023).
194 Schürks, M. *et al.* Cluster headache is associated with the G1246A polymorphism in the hypocretin receptor 2 gene. *Neurology* **66**, 1917-1919 (2006).
195 Ong, K. L., Lam, K. S. & Cheung, B. M. Urotensin II: its function in health and its role in disease. *Cardiovasc Drugs Ther* **19**, 65-75 (2005).
196 Huitron-Resendiz, S. *et al.* Urotensin II modulates rapid eye movement sleep through activation of brainstem cholinergic neurons. *J Neurosci* **25**, 5465-5474 (2005).
197 de Lecea, L. & Bourgin, P. Neuropeptide interactions and REM sleep: a role for Urotensin II? *Peptides* **29**, 845-851 (2008).
198 Nothacker, H. P. & Clark, S. From heart to mind. The urotensin II system and its evolving neurophysiological role. *FEBS J* **272**, 5694-5702 (2005).
199 De Fusco, M. *et al.* The nicotinic receptor beta 2 subunit is mutant in nocturnal frontal lobe epilepsy. *Nat Genet* **26**, 275-276 (2000).
200 Takeuchi, S., Shimizu, K., Fukada, Y. & Emoto, K. The circadian clock in the piriform cortex intrinsically tunes daily changes of odor-evoked neural activity. *Commun Biol* **6**, 332 (2023).
201 Toll, L., Bruchas, M. R., Calo, G., Cox, B. M. & Zaveri, N. T. Nociceptin/Orphanin FQ Receptor Structure, Signaling, Ligands, Functions, and Interactions with Opioid Systems. *Pharmacol Rev* **68**, 419-457 (2016).
202 Chen, C. Y. *et al.* Effects of aging on circadian patterns of gene expression in the human prefrontal cortex. *Proc Natl Acad Sci U S A* **113**, 206-211 (2016).
203 Morairty, S. R., Sun, Y., Toll, L., Bruchas, M. R. & Kilduff, T. S. Activation of the nociceptin/orphanin-FQ receptor promotes NREM sleep and EEG slow wave activity. *Proc Natl Acad Sci U S A* **120**, e2214171120 (2023).
204 Miyakawa, K. *et al.* ORL1 receptor-mediated down-regulation of mPER2 in the suprachiasmatic nucleus accelerates re-entrainment of the circadian clock following a shift in the environmental light/dark cycle. *Neuropharmacology* **52**, 1055-1064 (2007).
205 Seo, E. J., Efferth, T. & Panossian, A. Curcumin downregulates expression of opioid-related nociceptin receptor gene (OPRL1) in isolated neuroglia cells. *Phytomedicine* **50**, 285-299 (2018).

206 Bannuru, R. R., Osani, M. C., Al-Eid, F. & Wang, C. Efficacy of curcumin and Boswellia for knee osteoarthritis: Systematic review and meta-analysis. *Semin Arthritis Rheum* **48**, 416-429 (2018).
207 Black, B. L. & Olson, E. N. Transcriptional control of muscle development by myocyte enhancer factor-2 (MEF2) proteins. *Annu Rev Cell Dev Biol* **14**, 167-196 (1998).
208 Potthoff, M. J. & Olson, E. N. MEF2: a central regulator of diverse developmental programs. *Development* **134**, 4131-4140 (2007).
209 Wu, W., de Folter, S., Shen, X., Zhang, W. & Tao, S. Vertebrate paralogous MEF2 genes: origin, conservation, and evolution. *PLoS One* **6**, e17334 (2011).
210 Fernandez-Marcos, P. J. & Auwerx, J. Regulation of PGC-1α, a nodal regulator of mitochondrial biogenesis. *Am J Clin Nutr* **93**, 884s-890 (2011).
211 Czubryt, M. P., McAnally, J., Fishman, G. I. & Olson, E. N. Regulation of peroxisome proliferator-activated receptor gamma coactivator 1 alpha (PGC-1 alpha) and mitochondrial function by MEF2 and HDAC5. *Proc Natl Acad Sci U S A* **100**, 1711-1716 (2003).
212 Froy, O. & Garaulet, M. The Circadian Clock in White and Brown Adipose Tissue: Mechanistic, Endocrine, and Clinical Aspects. *Endocr Rev* **39**, 261-273 (2018).
213 Liu, C., Li, S., Liu, T., Borjigin, J. & Lin, J. D. Transcriptional coactivator PGC-1alpha integrates the mammalian clock and energy metabolism. *Nature* **447**, 477-481 (2007).
214 Blanchard, F. J. *et al.* The transcription factor Mef2 is required for normal circadian behavior in Drosophila. *J Neurosci* **30**, 5855-5865 (2010).
215 Mohawk, J. A. *et al.* Neuronal Myocyte-Specific Enhancer Factor 2D (MEF2D) Is Required for Normal Circadian and Sleep Behavior in Mice. *J Neurosci* **39**, 7958-7967 (2019).
216 Nakamura, K., Inoue, I., Takahashi, S., Komoda, T. & Katayama, S. Cryptochrome and Period Proteins Are Regulated by the CLOCK/BMAL1 Gene: Crosstalk between the PPARs/RXRalpha-Regulated and CLOCK/BMAL1-Regulated Systems. *PPAR Res* **2008**, 348610 (2008).
217 Chen, L. & Yang, G. PPARs Integrate the Mammalian Clock and Energy Metabolism. *PPAR Res* **2014**, 653017 (2014).
218 Yang, G. *et al.* Systemic PPARγ deletion impairs circadian rhythms of behavior and metabolism. *PLoS One* **7**, e38117 (2012).
219 Matagne, V. *et al.* Thyroid transcription factor 1, a homeodomain containing transcription factor, contributes to regulating periodic oscillations in GnRH gene expression. *J Neuroendocrinol* **24**, 916-929 (2012).
220 Son, Y. J. *et al.* Expression and role of TTF-1 in the rat suprachiasmatic nucleus. *Biochem Biophys Res Commun* **380**, 559-563 (2009).
221 Ertosun, M. G., Kocak, G. & Ozes, O. N. The regulation of circadian clock by tumor necrosis factor alpha. *Cytokine Growth Factor Rev* **46**, 10-16 (2019).
222 Li, C. X. *et al.* Altered melatonin secretion and circadian gene expression with increased proinflammatory cytokine expression in early-stage sepsis patients. *Mol Med Rep* **7**, 1117-1122 (2013).
223 Pontes, G. N., Cardoso, E. C., Carneiro-Sampaio, M. M. & Markus, R. P. Pineal melatonin and the innate immune response: the TNF-alpha increase after cesarean section suppresses nocturnal melatonin production. *J Pineal Res* **43**, 365-371 (2007).
224 Yoshida, K. *et al.* TNF-α induces expression of the circadian clock gene Bmal1 via dual calcium-dependent pathways in rheumatoid synovial cells. *Biochem Biophys Res Commun* **495**, 1675-1680 (2018).

225 Petrzilka, S., Taraborrelli, C., Cavadini, G., Fontana, A. & Birchler, T. Clock gene modulation by TNF-alpha depends on calcium and p38 MAP kinase signaling. *J Biol Rhythms* **24**, 283-294 (2009).
226 Lopez, M. *et al.* Tumor necrosis factor and transforming growth factor β regulate clock genes by controlling the expression of the cold inducible RNA-binding protein (CIRBP). *J Biol Chem* **289**, 2736-2744 (2014).
227 Chisari, E., Yaghmour, K. M. & Khan, W. S. The effects of TNF-alpha inhibition on cartilage: a systematic review of preclinical studies. *Osteoarthritis Cartilage* **28**, 708-718 (2020).
228 Toledo, M. *et al.* Autophagy Regulates the Liver Clock and Glucose Metabolism by Degrading CRY1. *Cell Metab* **28**, 268-281.e264 (2018).
229 Rabinovich-Nikitin, I., Kirshenbaum, E. & Kirshenbaum, L. A. Autophagy, Clock Genes, and Cardiovascular Disease. *Can J Cardiol* **39**, 1772-1780 (2023).
230 Liu, J., Yang, M., Kang, R., Klionsky, D. J. & Tang, D. Autophagic degradation of the circadian clock regulator promotes ferroptosis. *Autophagy* **15**, 2033-2035 (2019).
231 Zhang, S. *et al.* The Role Played by Ferroptosis in Osteoarthritis: Evidence Based on Iron Dyshomeostasis and Lipid Peroxidation. *Antioxidants (Basel)* **11**, 1668 (2022).
232 Baeza-Raja, B. *et al.* p75 neurotrophin receptor is a clock gene that regulates oscillatory components of circadian and metabolic networks. *J Neurosci* **33**, 10221-10234 (2013).
233 Iannone, F. *et al.* Increased expression of nerve growth factor (NGF) and high affinity NGF receptor (p140 TrkA) in human osteoarthritic chondrocytes. *Rheumatology (Oxford)* **41**, 1413-1418 (2002).
234 Denk, F., Bennett, D. L. & McMahon, S. B. Nerve Growth Factor and Pain Mechanisms. *Annu Rev Neurosci* **40**, 307-325 (2017).
235 Woolf, C. J., Safieh-Garabedian, B., Ma, Q. P., Crilly, P. & Winter, J. Nerve growth factor contributes to the generation of inflammatory sensory hypersensitivity. *Neuroscience* **62**, 327-331 (1994).
236 Downton, P. *et al.* Chronic inflammatory arthritis drives systemic changes in circadian energy metabolism. *Proc Natl Acad Sci U S A* **119**, e2112781119 (2022).
237 Domowicz, M. S., Sanders, T. A., Ragsdale, C. W. & Schwartz, N. B. Aggrecan is expressed by embryonic brain glia and regulates astrocyte development. *Dev Biol* **315**, 114-124 (2008).
238 Schröder, J. E. *et al.* Dystroglycan regulates structure, proliferation and differentiation of neuroepithelial cells in the developing vertebrate CNS. *Dev Biol* **307**, 62-78 (2007).
239 Tian, M. *et al.* Dystroglycan in the cerebellum is a laminin alpha 2-chain binding protein at the glial-vascular interface and is expressed in Purkinje cells. *Eur J Neurosci* **8**, 2739-2747 (1996).
240 Nickolls, A. R. & Bönnemann, C. G. The roles of dystroglycan in the nervous system: insights from animal models of muscular dystrophy. *Dis Model Mech* **11**, dmm035931 (2018).
241 Saito, F. *et al.* Unique role of dystroglycan in peripheral nerve myelination, nodal structure, and sodium channel stabilization. *Neuron* **38**, 747-758 (2003).
242 Ji-Wei, S. *et al.* CNTF induces Clcf1 in astrocytes to promote the differentiation of oligodendrocyte precursor cells. *Biochem Biophys Res Commun* **636**, 170-177 (2022).
243 Xu, X. *et al.* YAP prevents premature senescence of astrocytes and cognitive decline of Alzheimer's disease through regulating CDK6 signaling. *Aging Cell* **20**, e13465 (2021).
244 Sussel, L., Marin, O., Kimura, S. & Rubenstein, J. L. Loss of Nkx2.1 homeobox gene function results in a ventral to dorsal molecular respecification within the basal

telencephalon: evidence for a transformation of the pallidum into the striatum. *Development* **126**, 3359-3370 (1999).
245 Minocha, S. *et al.* Nkx2.1 regulates the generation of telencephalic astrocytes during embryonic development. *Sci Rep* **7**, 43093 (2017).
246 Skjoerringe, T., Lundvig, D. M., Jensen, P. H. & Moos, T. P25alpha/Tubulin polymerization promoting protein expression by myelinating oligodendrocytes of the developing rat brain. *J Neurochem* **99**, 333-342 (2006).
247 Lehotzky, A. *et al.* Tubulin polymerization-promoting protein (TPPP/p25) is critical for oligodendrocyte differentiation. *Glia* **58**, 157-168 (2010).
248 Socodato, R. *et al.* RhoA balances microglial reactivity and survival during neuroinflammation. *Cell Death Dis* **14**, 690 (2023).
249 Adães, S. *et al.* Glial activation in the collagenase model of nociception associated with osteoarthritis. *Mol Pain* **13**, 1744806916688219 (2017).
250 Saito, T. & Koshino, T. Distribution of neuropeptides in synovium of the knee with osteoarthritis. *Clin Orthop Relat Res* **376**, 172-182 (2000).
251 Saito, T. Neurogenic inflammation in osteoarthritis of the knee. *Mod Rheumatol* **13**, 301-304 (2003).
252 Bonnet, C. S. & Walsh, D. A. Osteoarthritis, angiogenesis and inflammation. *Rheumatology (Oxford)* **44**, 7-16 (2005).
253 Matsuda, M., Huh, Y. & Ji, R. R. Roles of inflammation, neurogenic inflammation, and neuroinflammation in pain. *J Anesth* **33**, 131-139 (2019).
254 Amodeo, G. *et al.* Osteoarthritis Pain in Old Mice Aggravates Neuroinflammation and Frailty: The Positive Effect of Morphine Treatment. *Biomedicines* **10**, 2847 (2022).
255 Bourassa, V., Deamond, H., Yousefpour, N., Fitzcharles, M. A. & Ribeiro-da-Silva, A. Pain-related behavior is associated with increased joint innervation, ipsilateral dorsal horn gliosis, and dorsal root ganglia activating transcription factor 3 expression in a rat ankle joint model of osteoarthritis. *Pain Rep* **5**, e846 (2020).
256 Martin Gil, C. *et al.* Myostatin and CXCL11 promote nervous tissue macrophages to maintain osteoarthritis pain. *Brain Behav Immun* **116**, 203-215 (2024).
257 Ohashi, Y., Uchida, K., Fukushima, K., Inoue, G. & Takaso, M. Mechanisms of Peripheral and Central Sensitization in Osteoarthritis Pain. *Cureus* **15**, e35331 (2023).
258 Sagar, D. R. *et al.* The contribution of spinal glial cells to chronic pain behaviour in the monosodium iodoacetate model of osteoarthritic pain. *Mol Pain* **7**, 88 (2011).
259 Ji, R. R., Berta, T. & Nedergaard, M. Glia and pain: is chronic pain a gliopathy? *Pain* **154 Suppl 1**, S10-s28 (2013).
260 Neogi, T. *et al.* Association between radiographic features of knee osteoarthritis and pain: results from two cohort studies. *BMJ* **339**, b2844 (2009).
261 Steenkamp, W., Rachuene, P. A., Dey, R., Mzayiya, N. L. & Ramasuvha, B. E. The correlation between clinical and radiological severity of osteoarthritis of the knee. *SICOT J* **8**, 14 (2022).
262 Dainese, P. *et al.* Association between knee inflammation and knee pain in patients with knee osteoarthritis: a systematic review. *Osteoarthritis Cartilage* **30**, 516-534 (2022).
263 Feldman, R. G. & Ridgway, G. L. Database handling for infection control and hospital epidemiology. *J Hosp Infect* **11 Suppl A**, 37-42 (1988).
264 Iuamoto, L. R. *et al.* Effects of neuroplasticity in people with knee osteoarthritis: A systematic review of the literature. *Medicine (Baltimore)* **101**, e28616 (2022).
265 Gupta, D. P. *et al.* Knee osteoarthritis accelerates amyloid beta deposition and neurodegeneration in a mouse model of Alzheimer's disease. *Mol Brain* **16**, 1 (2023).

266 Ikram, M., Innes, K. & Sambamoorthi, U. Association of osteoarthritis and pain with Alzheimer's Diseases and Related Dementias among older adults in the United States. *Osteoarthritis Cartilage* **27**, 1470-1480 (2019).
267 Nazarinasab, M., Motamedfar, A. & Moqadam, A. E. Investigating mental health in patients with osteoarthritis and its relationship with some clinical and demographic factors. *Reumatologia* **55**, 183-188 (2017).
268 Wang, C., Wang, F., Lin, F., Duan, X. & Bi, B. Naproxen attenuates osteoarthritis progression through inhibiting the expression of prostaglandinl-endoperoxide synthase 1. *J Cell Physiol* **234**, 12771-12785 (2019).
269 Pulkkinen, H. H. *et al.* BMP6/TAZ-Hippo signaling modulates angiogenesis and endothelial cell response to VEGF. *Angiogenesis* **24**, 129-144 (2021).
270 Winkler, D. G. *et al.* Osteocyte control of bone formation via sclerostin, a novel BMP antagonist. *EMBO J* **22**, 6267-6276 (2003).
271 Wu, D., Li, L., Wen, Z. & Wang, G. Romosozumab in osteoporosis: yesterday, today and tomorrow. *J Transl Med* **21**, 668 (2023).
272 Alnaqbi, K. A., Al Zeyoudi, J. & Aljaberi, A. K. Cardiac Arrhythmia and Heart Failure Shortly After Starting Romosozumab for Osteoporosis: A Case-Based Review. *Cureus* **15**, e50303 (2023).
273 Fusaro, M. & Tripepi, G. Romosozumab Use and Cardiovascular Events. *J Bone Miner Res* **38**, 452-453 (2023).
274 1000 Genomes Project Consortium *et al.* A global reference for human genetic variation. *Nature* **526**, 68-74 (2015).
275 Das, S. *et al.* Next-generation genotype imputation service and methods. *Nat Genet* **48**, 1284-1287 (2016).
276 Taliun, D. *et al.* Sequencing of 53,831 diverse genomes from the NHLBI TOPMed Program. *Nature* **590**, 290-299 (2021).
277 Cook, J. P., Mahajan, A. & Morris, A. P. Guidance for the utility of linear models in meta-analysis of genetic association studies of binary phenotypes. *Eur J Hum Genet* **25**, 240-245 (2017).
278 Danecek, P. *et al.* Twelve years of SAMtools and BCFtools. *Gigascience* **10**, giab008 (2021).
279 Zhao, H. *et al.* CrossMap: a versatile tool for coordinate conversion between genome assemblies. *Bioinformatics* **30**, 1006-1007 (2014).
280 Winkler, T. W. *et al.* Quality control and conduct of genome-wide association meta-analyses. *Nat Protoc* **9**, 1192-1212 (2014).
281 Purcell, S. *et al.* PLINK: a tool set for whole-genome association and population-based linkage analyses. *Am J Hum Genet* **81**, 559-575 (2007).
282 Willer, C. J., Li, Y. & Abecasis, G. R. METAL: fast and efficient meta-analysis of genomewide association scans. *Bioinformatics* **26**, 2190-2191 (2010).
283 Lee, B. T. *et al.* The UCSC Genome Browser database: 2022 update. *Nucleic Acids Res* **50**, D1115-D1122 (2022).
284 McLaren, W. *et al.* The Ensembl Variant Effect Predictor. *Genome Biol* **17**, 122 (2016).
285 Ward, L. D. & Kellis, M. HaploReg v4: systematic mining of putative causal variants, cell types, regulators and target genes for human complex traits and disease. *Nucleic Acids Res* **44**, D877-881 (2016).
286 Bittner, N. *et al.* Primary osteoarthritis chondrocyte map of chromatin conformation reveals novel candidate effector genes. *Ann Rheum Dis*, doi: 10.1136/ard-2023-224945 (2024).
287 Roadmap Epigenomics Consortium *et al.* Integrative analysis of 111 reference human epigenomes. *Nature* **518**, 317-330 (2015).

288 Wang, X. & Yue, F. HiCLift: a fast and efficient tool for converting chromatin interaction data between genome assemblies. *Bioinformatics* **39**, btad389 (2023).
289 Cunningham, F. *et al.* Ensembl 2022. *Nucleic Acids Res* **50**, D988-D995 (2022).
290 Bravo González-Blas, C. *et al.* SCENIC+: single-cell multiomic inference of enhancers and gene regulatory networks. *Nat Methods* **20**, 1355-1367 (2023).
291 Kent, W. J. *et al.* The human genome browser at UCSC. *Genome Res* **12**, 996-1006 (2002).
292 Steinberg, J. *et al.* A molecular quantitative trait locus map for osteoarthritis. *Nat Commun* **12**, 1309 (2021).
293 Yang, J. *et al.* Conditional and joint multiple-SNP analysis of GWAS summary statistics identifies additional variants influencing complex traits. *Nat Genet* **44**, 369-375, s361-363 (2012).
294 Giambartolomei, C. *et al.* Bayesian test for colocalisation between pairs of genetic association studies using summary statistics. *PLoS Genet* **10**, e1004383 (2014).
295 Unger, S. *et al.* Nosology of genetic skeletal disorders: 2023 revision. *Am J Med Genet A* **191**, 1164-1209 (2023).
296 Butterfield, N. C. *et al.* Accelerating functional gene discovery in osteoarthritis. *Nat Commun* **12**, 467 (2021).
297 Boer, C. G. *et al.* Deciphering osteoarthritis genetics across 826,690 individuals from 9 populations. *Cell* **184**, 4784-4818.e4717 (2021).
298 Speed, D., Holmes, J. & Balding, D. J. Evaluating and improving heritability models using summary statistics. *Nat Genet* **52**, 458-462 (2020).
299 Wu, T. *et al.* clusterProfiler 4.0: A universal enrichment tool for interpreting omics data. *Innovation (Camb)* **2**, 100141 (2021).
300 Tuerlings, M. *et al.* RNA Sequencing Reveals Interacting Key Determinants of Osteoarthritis Acting in Subchondral Bone and Articular Cartilage: Identification of IL11 and CHADL as Attractive Treatment Targets. *Arthritis Rheumatol* **73**, 789-799 (2021).
301 Coutinho de Almeida, R. *et al.* Allelic expression imbalance in articular cartilage and subchondral bone refined genome-wide association signals in osteoarthritis. *Rheumatology (Oxford)* **62**, 1669-1676 (2023).
302 den Hollander, W. *et al.* Annotating Transcriptional Effects of Genetic Variants in Disease-Relevant Tissue: Transcriptome-Wide Allelic Imbalance in Osteoarthritic Cartilage. *Arthritis Rheumatol* **71**, 561-570 (2019).
303 Band, G. & Marchini, J. BGEN: a binary file format for imputed genotype and haplotype data. *bioRxiv*, 308296 (2018).
304 R Core Team. R: A language and environment for statistical computing. (R Foundation for Statistical Computing, Vienna, Austria, 2021). https://www.R-project.org/.
305 Trynka, G. *et al.* Chromatin marks identify critical cell types for fine mapping complex trait variants. *Nat Genet* **45**, 124-130 (2013).
306 Chen, E. Y. *et al.* Enrichr: interactive and collaborative HTML5 gene list enrichment analysis tool. *BMC Bioinformatics* **14**, 128 (2013).
307 Kuleshov, M. V. *et al.* Enrichr: a comprehensive gene set enrichment analysis web server 2016 update. *Nucleic Acids Res* **44**, W90-97 (2016).
308 Milacic, M. *et al.* The Reactome Pathway Knowledgebase 2024. *Nucleic Acids Res* **52**, D672-D678 (2024).
309 Agrawal, A. *et al.* WikiPathways 2024: next generation pathway database. *Nucleic Acids Res* **52**, D679-D689 (2024).
310 Styrkarsdottir, U. *et al.* Meta-analysis of Icelandic and UK data sets identifies missense variants in SMO, IL11, COL11A1 and 13 more new loci associated with osteoarthritis. *Nat Genet* **50**, 1681-1687 (2018).

311 Laursen, I. H. *et al.* Cohort profile: Copenhagen Hospital Biobank - Cardiovascular Disease Cohort (CHB-CVDC): Construction of a large-scale genetic cohort to facilitate a better understanding of heart diseases. *BMJ Open* **11**, e049709 (2021).
312 Erikstrup, C. *et al.* Cohort Profile: The Danish Blood Donor Study. *Int J Epidemiol* **52**, e162-e171 (2023).
313 Stanaway, I. B. *et al.* The eMERGE genotype set of 83,717 subjects imputed to ~40 million variants genome wide and association with the herpes zoster medical record phenotype. *Genet Epidemiol* **43**, 63-81 (2019).
314 Leitsalu, L., Alavere, H., Tammesoo, M. L., Leego, E. & Metspalu, A. Linking a population biobank with national health registries-the estonian experience. *J Pers Med* **5**, 96-106 (2015).
315 Carey, D. J. *et al.* The Geisinger MyCode community health initiative: an electronic health record-linked biobank for precision medicine research. *Genet Med* **18**, 906-913 (2016).
316 de Schepper, E. I. *et al.* The association between lumbar disc degeneration and low back pain: the influence of age, gender, and individual radiographic features. *Spine (Phila Pa 1976)* **35**, 531-536 (2010).
317 Määttä, J. H., Karppinen, J. I., Luk, K. D., Cheung, K. M. & Samartzis, D. Phenotype profiling of Modic changes of the lumbar spine and its association with other MRI phenotypes: a large-scale population-based study. *Spine J* **15**, 1933-1942 (2015).
318 Cheung, K. M. *et al.* Prevalence and pattern of lumbar magnetic resonance imaging changes in a population study of one thousand forty-three individuals. *Spine (Phila Pa 1976)* **34**, 934-940 (2009).
319 Teraguchi, M. *et al.* Lumbar high-intensity zones on MRI: imaging biomarkers for severe, prolonged low back pain and sciatica in a population-based cohort. *Spine J* **20**, 1025-1034 (2020).
320 Schneiderman, G. *et al.* Magnetic resonance imaging in the diagnosis of disc degeneration: correlation with discography. *Spine (Phila Pa 1976)* **12**, 276-281 (1987).
321 Anderson, C. A. *et al.* Data quality control in genetic case-control association studies. *Nat Protoc* **5**, 1564-1573 (2010).
322 McCarthy, S. *et al.* A reference panel of 64,976 haplotypes for genotype imputation. *Nat Genet* **48**, 1279-1283 (2016).
323 Krokstad, S. *et al.* Cohort Profile: the HUNT Study, Norway. *Int J Epidemiol* **42**, 968-977 (2013).
324 Jun, G. *et al.* Detecting and estimating contamination of human DNA samples in sequencing and array-based genotype data. *Am J Hum Genet* **91**, 839-848 (2012).
325 Guo, Y. *et al.* Illumina human exome genotyping array clustering and quality control. *Nat Protoc* **9**, 2643-2662 (2014).
326 ENCODE Project Consortium. An integrated encyclopedia of DNA elements in the human genome. *Nature* **489**, 57-74 (2012).
327 MacInnes, S. J. *et al.* The 2018 Otto Aufranc Award: How Does Genome-wide Variation Affect Osteolysis Risk After THA? *Clin Orthop Relat Res* **477**, 297-309 (2019).
328 Lester, G. The Osteoarthritis Initiative: A NIH Public-Private Partnership. *Hss j* **8**, 62-63 (2012).
329 Yau, M. S. *et al.* Genome-Wide Association Study of Radiographic Knee Osteoarthritis in North American Caucasians. *Arthritis Rheumatol* **69**, 343-351 (2017).
330 Liu, Y. *et al.* Genetic Determinants of Radiographic Knee Osteoarthritis in African Americans. *J Rheumatol* **44**, 1652-1658 (2017).
331 Lupton, M. K. *et al.* A prospective cohort study of prodromal Alzheimer's disease: Prospective Imaging Study of Ageing: Genes, Brain and Behaviour (PISA). *Neuroimage Clin* **29**, 102527 (2021).

332 Ramos, Y. F. *et al.* Genes involved in the osteoarthritis process identified through genome wide expression analysis in articular cartilage; the RAAK study. *PLoS One* **9**, e103056 (2014).
333 Coutinho de Almeida, R. *et al.* RNA sequencing data integration reveals an miRNA interactome of osteoarthritis cartilage. *Ann Rheum Dis* **78**, 270-277 (2019).
334 van Hoolwerff, M. *et al.* Elucidating Epigenetic Regulation by Identifying Functional cis-Acting Long Noncoding RNAs and Their Targets in Osteoarthritic Articular Cartilage. *Arthritis Rheumatol* **72**, 1845-1854 (2020).
335 Nakajima, M. *et al.* New sequence variants in HLA class II/III region associated with susceptibility to knee osteoarthritis identified by genome-wide association study. *PLoS One* **5**, e9723 (2010).
336 Völzke, H. *et al.* Cohort profile: the study of health in Pomerania. *Int J Epidemiol* **40**, 294-307 (2011).
337 Völzke, H. *et al.* Cohort Profile Update: The Study of Health in Pomerania (SHIP). *Int J Epidemiol* **51**, e372-e383 (2022).
338 Kuriyama, S. *et al.* The Tohoku Medical Megabank Project: Design and Mission. *J Epidemiol* **26**, 493-511 (2016).
339 Hozawa, A. *et al.* Study Profile of the Tohoku Medical Megabank Community-Based Cohort Study. *J Epidemiol* **31**, 65-76 (2021).
340 Hysi, P. G. *et al.* Genome-wide association meta-analysis of individuals of European ancestry identifies new loci explaining a substantial fraction of hair color variation and heritability. *Nat Genet* **50**, 652-656 (2018).
341 Sudlow, C. *et al.* UK biobank: an open access resource for identifying the causes of a wide range of complex diseases of middle and old age. *PLoS Med* **12**, e1001779 (2015).
342 O'Connell, J. *et al.* Haplotype estimation for biobank-scale data sets. *Nat Genet* **48**, 817-820 (2016).
343 Bycroft, C. *et al.* The UK Biobank resource with deep phenotyping and genomic data. *Nature* **562**, 203-209 (2018).
344 Huang, J. *et al.* Improved imputation of low-frequency and rare variants using the UK10K haplotype reference panel. *Nat Commun* **6**, 8111 (2015).
345 King, T., Butcher, S. & Zalewski, L. Apocrita - High Performance Computing Cluster for Queen Mary University of London. *Zenodo*, https://doi.org/10.5281/zenodo.438045 (2017).
346 Ripmeester, E. G. J., Timur, U. T., Caron, M. M. J. & Welting, T. J. M. Recent Insights into the Contribution of the Changing Hypertrophic Chondrocyte Phenotype in the Development and Progression of Osteoarthritis. *Front Bioeng Biotechnol* **6**, 18 (2018).
