# Supplementary material for: Translational genomics of osteoarthritis in 1,962,069 individuals
Source: Nature. 2025 Apr 9;641(8065):1217–24. doi: 10.1038/s41586-025-08771-z (PMC12119359; doi:10.1038/s41586-025-08771-z)
Supplement: Supplementary file 2 — Reporting Summary [file 41586_2025_8771_MOESM2_ESM.pdf]

nature portfolio

Corresponding author(s): Eleftheria Zeggini

Last updated by author(s): Jan 16, 2025

Reporting Summary

Nature Portfolio wishes to improve the reproducibility of the work that we publish. This form provides structure for consistency and transparency in reporting. For further information on Nature Portfolio policies, see our Editorial Policies and the Editorial Policy Checklist.

Statistics

For all statistical analyses, confirm that the following items are present in the figure legend, table legend, main text, or Methods section.

n/a	Confirmed	
☐	☒	The exact sample size (*n*) for each experimental group/condition, given as a discrete number and unit of measurement
☒	☐	A statement on whether measurements were taken from distinct samples or whether the same sample was measured repeatedly
☐	☒	The statistical test(s) used AND whether they are one- or two-sided<br>*Only common tests should be described solely by name; describe more complex techniques in the Methods section.*
☐	☒	A description of all covariates tested
☐	☒	A description of any assumptions or corrections, such as tests of normality and adjustment for multiple comparisons
☐	☒	A full description of the statistical parameters including central tendency (e.g. means) or other basic estimates (e.g. regression coefficient) AND variation (e.g. standard deviation) or associated estimates of uncertainty (e.g. confidence intervals)
☐	☒	For null hypothesis testing, the test statistic (e.g. *F*, *t*, *r*) with confidence intervals, effect sizes, degrees of freedom and *P* value noted<br>*Give P values as exact values whenever suitable.*
☐	☒	For Bayesian analysis, information on the choice of priors and Markov chain Monte Carlo settings
☒	☐	For hierarchical and complex designs, identification of the appropriate level for tests and full reporting of outcomes
☐	☒	Estimates of effect sizes (e.g. Cohen's *d*, Pearson's *r*), indicating how they were calculated

Our web collection on statistics for biologists contains articles on many of the points above.

Software and code

Policy information about availability of computer code

Data collection	No software was used for the collection of data
Data analysis	Analyses were performed using the following publicly available software: BCFtools version 1.13 (https://samtools.github.io/bcftools/bcftools.html, CrossMap version 0.5.4 (https://crossmap.readthedocs.io/en/latest/), EasyQC version 23.8, 5th June 2020 (https://www.uni-regensburg.de/medizin/epidemiologie-praeventivmedizin/genetische-epidemiologie/software), GWAMA version 2.2.2 (https://genomics.ut.ee/en/tools), METAL version released on 2011-03-25 (https://genome.sph.umich.edu/wiki/METAL_Documentation), METASOFT version 2.0.0 (2012-02-15 (http://genetics.cs.ucla.edu/meta_jemdoc/), PLINKv1.9 (https://www.cog-genomics.org/plink/1.9/), PLINKv2.09 (https://www.cog-genomics.org/plink/2.0/), R (https://www.R-project.org/), COJO in GCTA version 1.93.0beta (https://yanglab.westlake.edu.cn/software/gcta/#COJO), Functional GWAS analysis (https://github.com/natsuhiko/PHM), BGEN bgenix version: 1.1.7, revision (https://www.biorxiv.org/content/10.1101/308296v2), HiCLift (https://github.com/XiaoTaoWang/HiCLift), GRNBoost2 algorithm in Scenic+ software (https://github.com/aertslab/scenicplus), dbNFSFP version 4.1c (https://www.dbnsfp.org/home), CADD (https://cadd.gs.washington.edu/), LDAK version 6 (https://www.ldak.org). Analysis also included the following R packages: coloc version 5.2.2 (https://rdrr.io/cran/coloc/man/coloc.abf.html), ClusterProfiler64 (version 4.8.2), (https://bioconductor.org/packages/release/bioc/html/clusterProfiler.html), susieR package62 v. 0.12.27, R version: 4.2.163 (https://cran.r-project.org/web/packages/susieR/index.html). A collection of scripts that were used in this study are available at https://github.com/hmgu-itg/Genetics-of-Osteoarthritis-2.0.

For manuscripts utilizing custom algorithms or software that are central to the research but not yet described in published literature, software must be made available to editors and reviewers. We strongly encourage code deposition in a community repository (e.g. GitHub). See the Nature Portfolio guidelines for submitting code & software for further information.

Data

Policy information about availability of data

All manuscripts must include a data availability statement. This statement should provide the following information, where applicable:

- Accession codes, unique identifiers, or web links for publicly available datasets
- A description of any restrictions on data availability
- For clinical datasets or third party data, please ensure that the statement adheres to our policy

The data from the genome-wide summary statistics for each meta-analysis generated during this study have been deposited at the 'Downloads' page of the Musculoskeletal Knowledge Portal (https://msk.hugeamp.org/downloads.html) and are publicly available now.

Online resources, databases, datasets used:
Ensembl BioMart (http://grch37.ensembl.org/biomart/martview/, release 110, GRCh37)
UCSC liftOver tool (https://genome.ucsc.edu/cgi-bin/hgLiftOver)
ROADMAP ChromHMM (https://egg2.wustl.edu/roadmap/web_portal/index.html, https://egg2.wustl.edu/roadmap/web_portal/chr_state_learning.html)
Ensembl REST API (http://grch37.rest.ensembl.org)
University of California Santa Cruz (UCSC) Genome Browser (https://genome.ucsc.edu)
Ensembl (http://grch37.ensembl.org/Tools/VEP)
HaploReg (https://pubs.broadinstitute.org/mammals/haploreg/haploreg.php, version 4.2)
Mouse Genome Informatics database (www.informatics.jax.org)
International Mouse Phenotyping Consortium (http://www.mousephenotype.org/)
Online Mendelian Inheritance in Man (OMIM) database (https://www.omim.org/)
Human Pain Genetics Database (https://humanpaingeneticsdb.ca/hpgdb/)
Enrichr (https://maayanlab.cloud/Enrichr/)
Gene Ontology (https://geneontology.org/)
Reactome (https://reactome.org/)
Wikipathways (https://www.wikipathways.org/)
Open Targets (https://platform.opentargets.org/downloads, version: 23.09)
BioRender (https://www.biorender.com/)

Research involving human participants, their data, or biological material

Policy information about studies with human participants or human data. See also policy information about sex, gender (identity/presentation), and sexual orientation and race, ethnicity and racism.

Reporting on sex and gender	The numbers of males and females for each contributing cohort are included in Supplementary Table 2. Sex-stratified analyses were performed in a subset of contributing cohorts.
Reporting on race, ethnicity, or other socially relevant groupings	Ancestral groups are reported according to 1000 Genomes major sub-groups.
Population characteristics	Population characteristics for each contributing study are included in Supplementary Table 2.
Recruitment	Ascertainment for osteoarthritis cases and controls for each contributing study are presented in Supplementary Table 2.
Ethics oversight	All participants provided written informed consent. The ethics statements from each contributing study are provided in the Supplementary Note. All human research was approved within each contributing study by the relevant institutional review boards and conducted according to the Declaration of Helsinki.

Note that full information on the approval of the study protocol must also be provided in the manuscript.

Field-specific reporting

Please select the one below that is the best fit for your research. If you are not sure, read the appropriate sections before making your selection.

☒ Life sciences ☐ Behavioural & social sciences ☐ Ecological, evolutionary & environmental sciences

For a reference copy of the document with all sections, see nature.com/documents/nr-reporting-summary-flat.pdf

Life sciences study design

All studies must disclose on these points even when the disclosure is negative.

Sample size	We have performed genome-wide association study meta-analysis for osteoarthritis, across 1,962,069 individuals, including 489,975 osteoarthritis cases. This is the largest GWAS meta-analysis for osteoarthritis to date. In order to reach this sample size, we included as many global GWAS summary statistics as possible that contained the relevant phenotypes. With our sample size of 489,975 osteoarthritis cases and

	1,472,094 controls, at a study-wide significance threshold (p≤1.3x10-8), under an additive genetic model of homogeneous effects across ancestry groups, we had ≥80% power to detect association of variants with MAF ≥5% and OR ≥1.0355 or MAF ≥1% and OR ≥1.078.
Data exclusions	Within each contributing study, individuals and variants were excluded based on well-established individual and variant quality control procedures to eliminate poor quality genotypes, samples, and variants. The procedures and thresholds for each study are detailed in Supplementary Table 2.
Replication	For each osteoarthritis associated signal that reached study-wide significance (P<1.3x10-8) we required that the signal was internally replicated: nominally significant (P<0.05) in at least 2 contributing studies with the same direction of effect.
Randomization	Randomization was not performed. Within each study, covariates were included to account for potential confounding. The covariate adjustments are reported in Supplementary Table 2.
Blinding	Group allocation was not relevant to this study, so blinding was not necessary.

Reporting for specific materials, systems and methods

We require information from authors about some types of materials, experimental systems and methods used in many studies. Here, indicate whether each material, system or method listed is relevant to your study. If you are not sure if a list item applies to your research, read the appropriate section before selecting a response.

Materials & experimental systems

n/a	Involved in the study
☒	☐ Antibodies
☒	☐ Eukaryotic cell lines
☒	☐ Palaeontology and archaeology
☒	☐ Animals and other organisms
☒	☐ Clinical data
☒	☐ Dual use research of concern
☒	☐ Plants

Methods

n/a	Involved in the study
☒	☐ ChIP-seq
☒	☐ Flow cytometry
☒	☐ MRI-based neuroimaging

Plants

Seed stocks	Not applicable
Novel plant genotypes	Not applicable
Authentication	Not applicable
